# ShenQi DiHuang Decoction (SQDHD) Ameliorates Neuroinflammation and Neuropsychiatric Manifestations in Pristane Induced Lupus Mice via Blocking JAK1‐STAT3 Pathway

**DOI:** 10.1002/cns.70814

**Published:** 2026-03-07

**Authors:** Jie Chen, Chumiao Cui, Fei Xu, Jiayan Feng, Jingyu Chen, Xueru Wang, Hui Yuan, Chenye Jin, Yutian Li, Yang Yun

**Affiliations:** ^1^ Department of Cardiology Shengjing Hospital of China Medical University Shenyang China; ^2^ Department of Nephrology Shengjing Hospital of China Medical University Shenyang China; ^3^ Department of Nephrology Dalian Municipal Central Hospital Dalian China; ^4^ Dalian Key Laboratory of Intelligent Blood Purification Dalian Municipal Central Hospital Dalian China; ^5^ Department of Physiology China Medical University Shenyang China; ^6^ Department of Rheumatology and Immunology First Affiliated Hospital of China Medical University Shenyang China

**Keywords:** cerebrovascular endothelial cells, Janus kinase‐signal transducer and activator of transcription signaling pathway, neuroinflammation, neuropsychiatric systemic lupus erythematosus, ShenQi DiHuang decoction, traditional Chinese medicine

## Abstract

**Aims:**

Neuroinflammation is widely acknowledged as a crucial pathogenic factor in neuropsychiatric systemic lupus erythematosus (NPSLE). However, specific clinical treatments to mitigate neuroinflammation associated with NPSLE are currently lacking. While ShenQi DiHuang decoction (SQDHD) has demonstrated significant anti‐inflammatory effects in lupus nephritis, its efficacy in NPSLE has yet to be investigated. This study aims to explore the neuroprotective effects of SQDHD in NPSLE and to elucidate the underlying mechanisms.

**Methods:**

In vivo, the effects of SQDHD were studied in pristane‐induced lupus (PIL) mice using behavioral tests, intravital microscopy, blood–brain barrier (BBB) permeability assessment, cytokine quantification, and brain histopathological analysis. The active compounds and the underlying mechanism of SQDHD action against NPSLE were examined using ultra performance liquid chromatography–tandem mass spectrometry (UPLC‐MS/MS), network pharmacology, molecular docking, cellular thermal shift assays (CETSAs), and drug affinity responsive target stability (DARTS) assays. In vitro and in vivo experiments were performed to validate the proposed mechanism.

**Results:**

SQDHD significantly ameliorated olfactory dysfunction, anxiety, and depression in PIL mice. Additionally, adhesion molecule upregulation, leukocyte recruitment, BBB leakage, and brain pathophysiological alterations, including cytokine overexpression, immunoglobulin G deposition, and lipofuscin accumulation were markedly reduced. By integrating UPLC‐MS/MS, network pharmacology, and molecular docking, we predicted the therapeutic mechanism of SQDHD against NPSLE to involve Janus kinase‐signal transducer and activator of transcription (JAK–STAT) signaling. Five primary active compounds of SQDHD, Alisol B acetate, Hederagenin, Ellagic acid, Wogonin, and Quercetin, exhibited strong binding affinities to JAK1 and other JAK–STAT pathway components, surpassing the binding affinities of Upadacitinib, a selective JAK1 inhibitor. CETSAs and DARTS assays confirmed the direct interactions between these compounds and JAK1. Alisol B acetate and Hederagenin inhibited the JAK1‐STAT3 pathway and its downstream effectors in cerebrovascular endothelial cells (CVECs). In vitro studies in lupus serum‐induced CVECs and in vivo studies in PIL mice further corroborated SQDHD downregulation of elevated levels of adhesion molecules, potentially through inhibition of JAK1‐STAT3 signaling.

**Conclusions:**

SQDHD may exert neuroprotective effects in NPSLE by inhibiting the activation of CVECs through blocking the JAK1‐STAT3 signaling pathway, thereby suggesting its potential as a promising therapeutic strategy for NPSLE.

## Graphical abstract

SQDHD exerts neuroprotective effects and alleviates behavioral deficits in PIL mice, likely by inhibiting the JAK1‐STAT3 pathway in activated CVECs. These findings underscore the therapeutic potential of SQDHD and establish it as a promising candidate for the treatment of NPSLE.

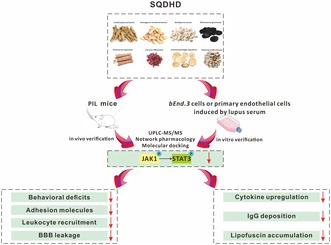

**Abbreviations**
2D: 2‐dimensional
AMPK: adenosine monophosphate‐activated protein kinase
ANOVA: one‐way analysis of variance
BBB: blood–brain barrier
BP: biological processes
CC: cellular components
CNS: central nervous system
CT: compound‐target
Ctrl: control
CVECs: cerebrovascular endothelial cells
DARTS: drug affinity responsive target stability
DAVID: Database for Annotation, Visualization, and Integrated Discovery
DMEM: Dulbecco's modified Eagle's medium
DMSO: dimethyl sulfoxide
ERK: extracellular‐signal‐regulated kinase
ESI‐QTRAP: electrospray ionization triple‐quadrupole linear ion trap
FBS: fetal bovine serum
GO: Gene Ontology
H‐SQDHD: high‐dose SQDHD
IgG: immunoglobulin G
IL‐6: interleukin 6
JAKi: JAK inhibitors
JAK–STAT: Janus kinase‐signal transducer and activator of transcription
KEGG: Kyoto encyclopedia of genes and genomes
LN: lupus nephritis
MAPK: mitogen‐activated protein kinase
MF: molecular functions
MFI: Mean fluorescence intensity
MRM: multiple reaction monitoring
M‐SQDHD: medium‐dose SQDHD
mTOR: mammalian target of rapamycin
NF‐κB: nuclear factor‐kappa B
NPSLE: neuropsychiatric systemic lupus erythematosus
PBS: phosphate buffer saline
PDB: Protein Data Bank
PIL: pristane‐induced lupus
PPI: protein–protein interaction
qRT‐PCR: Quantitative real‐time polymerase chain reaction
RCSB: Research Collaboratory for Structural Bioinformatics
SEM: standard error of the mean
SLE: Systemic lupus erythematosus
SQDHD: ShenQi DiHuang decoction
TCM: traditional Chinese medicine
TICs: total ion chromatograms
TNF‐α: tumor necrosis factor‐α
UPLC‐MS/MS: ultra‐performance liquid chromatography–tandem mass spectrometry
VCAM‐1: vascular cell adhesion molecule‐1

## 1 Introduction

Systemic lupus erythematosus (SLE) is a multifactorial autoimmune disease characterized by multi‐organ damage, notably affecting the skin, kidneys, and brain [1]. Neuropsychiatric SLE (NPSLE), which affects the central nervous system (CNS), manifests with a variety of symptoms, including mood disorders, sensory impairments, and cognitive dysfunction [2, 3]. Despite advancements in the survival rates and prognosis of SLE in recent decades, NPSLE remains a leading cause of disease‐related morbidity and mortality, ranking second only to lupus nephritis (LN) [4]. Therefore, there is an urgent need to elucidate the pathogenesis of NPSLE and to develop more effective treatment strategies.

The pathogenesis of NPSLE is widely understood to begin with inflammatory mediators from the peripheral circulation disrupting the blood–brain barrier (BBB). This breach, in turn, triggers CNS inflammation that propagates a deleterious cascade of glial activation, neuronal damage, and ultimately, behavioral deficits [5]. Cerebrovascular endothelial cells (CVECs) are essential for maintaining BBB integrity [6]. When exposed to chronic inflammatory stimuli associated with SLE, CVECs activate intracellular signaling pathways, resulting in the expression of adhesion molecules and additional pro‐inflammatory mediators [7]. The Janus kinase‐signal transducer and activator of transcription (JAK–STAT) pathway is a critical pro‐inflammatory signaling cascade [8]. Ligands such as interleukin 6 (IL‐6) or interferon initiate JAK phosphorylation, which subsequently activates STATs [8]. Notably, the JAK–STAT signaling pathway is implicated in endothelial activation, leading to the upregulation of adhesion molecule expression [9, 10]. Inhibition of this pathway effectively attenuates endothelial activation, thereby reducing leukocyte‐endothelial interactions [10]. Animal studies suggest that JAK inhibitors (JAKi) may alleviate behavioral abnormalities by targeting brain cells in regions with compromised BBB [11]. Hence, targeting JAK–STAT signaling in CVECs may present a promising therapeutic approach for NPSLE.

Current treatment options for NPSLE are limited, with most patients depending on empirical therapies such as immunosuppressive agents, corticosteroids, or symptomatic treatments [12]. However, the adverse effects of these therapies, including an increased risk of infection and steroid‐induced psychosis, impose a significant burden on NPSLE patients. In recent years, traditional Chinese medicine (TCM) has gained increasing recognition for its multi‐target and multi‐functional approach to treating complex and refractory diseases, particularly autoimmune disorders [13]. Several TCMs, such as *tripterygium wilfordii*, *red peony root*, and *sweet wormwood herb*, have been validated for their anti‐inflammatory and immunoregulatory properties in LN [14]. ShenQi DiHuang decoction (SQDHD), a formulation comprising eight traditional Chinese herbs, was first documented in the Qing Dynasty medical classic “Shen Shi Zun Sheng Shu” (1773 A.D.) and embodies Yiqi and Huoxue therapy [15]. SQDHD is primarily utilized in TCM for the management of diverse kidney conditions, fever, and other symptoms, notably reducing infection duration and slowing disease progression [16]. Furthermore, key components of SQDHD have been shown to elicit potent anti‐inflammatory effects by inhibiting the JAK–STAT signaling pathway, such as the modulation of microglia polarization [17, 18], the mitigation of intestinal inflammation [19], and the suppression of renal NOD‐like receptor protein 3 inflammasome activation [20]. Nevertheless, the therapeutic efficacy and mechanisms of SQDHD in NPSLE remain inadequately explored.

To examine the impact of SQDHD on neuropsychiatric manifestations and its underlying mechanisms, we utilized the pristane‐induced lupus (PIL) mouse model, which is known for mirroring the neurological and behavioral abnormalities observed in NPSLE patients [21]. Through a combination of ultra‐performance liquid chromatography–tandem mass spectrometry (UPLC‐MS/MS), network pharmacology, molecular docking, cellular thermal shift assay (CETSA), drug affinity responsive target stability (DARTS) assay, and experimental validation, we investigated the efficacy and mechanisms of SQDHD in the treatment of NPSLE. Our results provide robust experimental evidence that supports the application of TCM‐based strategies for NPSLE in clinical practice.

## 2 Materials and Methods

### 2.1 Animal Experimental Design

Female BALB/c mice were randomly assigned to six groups (*n* = 12 per group): control (Ctrl), NPSLE model (PIL), low‐dose SQDHD (L‐SQDHD), medium‐dose SQDHD (M‐SQDHD), high‐dose SQDHD (H‐SQDHD), and positive drug (JAKi) groups. At the onset of the study, mice received a single intraperitoneal injection of 0.5 mL of phosphate buffer saline (PBS) or pristane (Sigma‐Aldrich, St. Louis, MO, USA), respectively. Subsequently, mice received PBS, varying concentrations of SQDHD, or JAKi (Upadacitinib, 6 mg/kg/day, MedChemExpress, NJ, USA) in their daily drinking water for 4 months. Following behavioral assessments and intravital microscopy recordings, mice were euthanized. Blood samples were obtained via retro‐orbital puncture for in vitro analyses, and brain tissues were collected for further investigations.

### 2.2 Animals

Specific pathogen‐free female BALB/c mice (8 weeks old) were purchased from Vital River Laboratory (Beijing, CHN). Mice were housed in standard cages under controlled environmental conditions (12/12 h light/dark cycle, 22°C ± 2°C, 40%–80% humidity) with *ad libitum* access to food and water. All experimental procedures adhered to the *Guide for the Care and Use of Laboratory Animals* (National Institutes of Health) and received approval from the Animal Care and Use Committee of Shengjing Hospital affiliated with China Medical University (No. 2024PS589K). Measures were implemented to minimize animal distress throughout the study.

### 2.3 Composition and Preparation of SQDHD

SQDHD was prepared using eight raw herbs sourced from the pharmacy department of Shengjing Hospital affiliated with China Medical University (Shenyang, CHN). The herbal mixture, composed of *Codonopsis pilosula* (Franch.) Nannf, *Astragalus membranaceus* (Fisch.) Bunge, *Wolfiporia cocos* (F.A. Wolf) Ryvarden & Gilb, *Rehmannia glutinosa* (Gaertn.) DC, *Dioscorea opposita* Thunb, *Cornus officinalis* Siebold and Zucc, *Alisma plantago‐aquatica* L. subsp. *orientale* (Sam.) Sam, and *Paeonia suffruticosa* Andrews in a weight ratio of 3:3:2:2:2:2:2:1, was decocted twice in distilled water (1:8, w/v) for 1.5 h per cycle. The supernatant was concentrated to 200 mL using a rotary evaporator, frozen at −80°C overnight, and subsequently lyophilized to yield 45 g of powder. Employing a human‐mouse dose conversion coefficient of 9.1, SQDHD was administered at three concentrations: 2.93 g/kg (L‐SQDHD), 5.85 g/kg (M‐SQDHD), and 11.7 g/kg (H‐SQDHD). The powder was dissolved in drinking water, with concentrations adjusted based on daily water consumption to ensure precise dosing. Administration of SQDHD via drinking water was chosen to minimize the chronic stress associated with daily oral gavage, a critical consideration for valid assessment of neuropsychiatric behaviors [22, 23, 24]. The suitability and efficacy of this administration route are corroborated by previous neuropsychiatric disease literature [25, 26, 27, 28, 29]. Furthermore, our pilot studies confirmed that daily gavage itself induces abnormal behaviors in mice. Therefore, administering SQDHD via drinking water represents a viable and methodologically superior approach for the behavioral and neuroimmunological research conducted in this study.

### 2.4 UPLC‐MS/MS Analysis

For UPLC‐MS/MS analysis, 50 mg of SQDHD powder was immersed in 70% methanol containing the internal standard extraction solution, followed by centrifugation for 3 min to isolate the supernatant. The supernatant was then filtered through a microporous membrane and stored in an injection vial. The analysis utilized an Agilent SB‐C18 column (1.8 μm, 2.1 × 100 mm) with a mobile phase composed of solvent A (0.1% formic acid in pure water) and solvent B (0.1% formic acid in acetonitrile). A gradient program was implemented, beginning with 95% A and 5% B, transitioning linearly to 5% A and 95% B over 9 min, maintaining this composition for 1 min, and returning to the initial conditions (95% A and 5% B) within 1.1 min. The flow rate was set at 0.35 mL/min with the column oven maintained at 40°C. The effluent was analyzed using an electrospray ionization triple‐quadrupole linear ion trap (ESI‐QTRAP) mass spectrometer with the following parameters: source temperature, 500°C; ion spray voltage, 5500 V (positive) or −4500 V (negative); gas I, 50 psi; gas II, 60 psi; curtain gas, 25 psi. Specific metabolites were monitored via multiple reaction monitoring (MRM) transitions, with declustering potential and collision energy optimized for each transition.

### 2.5 Behavioral Tests

#### 2.5.1 Open Field Test

Mice were placed in an open field chamber (40 × 28 × 40 cm) with a central zone (20 × 14 cm) and allowed to explore freely for 30 min. A custom‐built program was utilized to digitally track and analyze the total distance traveled (km) and the time spent in the center (s).

#### 2.5.2 Olfactory Sensitivity Test

Mice were exposed to odorant‐infused filter paper for 2 min, followed by a 1‐min rest, repeated three times. Sniffing activity within 0.5 cm of the odorant source was recorded, and the total sniffing time was calculated.

#### 2.5.3 Forced Swim Test

Mice were placed into a glass beaker filled with water (3000 mL), maintained at approximately 24°C ± 1°C. After habituation to swimming in the beaker for 2 min, a 4‐min test session was digitally recorded. Immobility time (s), indicative of depression‐like behavior, was measured.

### 2.6 Intravital Microscopy

To fluorescently label leukocytes, mice received an intravenous injection of rhodamine 6G (0.5 mg/kg, Sigma‐Aldrich). Following immobilization of the mice in a stereotaxic apparatus, the skull was thinned and treated with 10% EDTA disodium (Sigma‐Aldrich) for 5–10 min to establish an optical clearing window. Rolling leukocytes were identified as cells moving slower than erythrocytes, while adherent leukocytes were defined as cells remaining stationary for at least 30 s.

### 2.7 BBB Permeability Analysis

The analysis of BBB permeability was performed by measuring the extravasation of Evans blue. Evans blue dye (2%, 4 mL/kg, Beyotime Biotechnology, Shanghai, CHN) was injected intravenously and circulated for 30 min. After cardiac perfusion, brain tissues used for BBB permeability analysis were homogenized and incubated in formamide (24 h, 55°C). BBB permeability was assessed by measuring the absorbance of the supernatant at 620 nm for each sample. Evans blue extravasation was quantified and expressed as micrograms per gram (μg/g) of wet tissue.

### 2.8 Immunofluorescence Staining

Brain tissues for immunofluorescence staining were fixed in 4% paraformaldehyde for 24 h at 4°C, followed by immersion in 30% sucrose solution, and then sectioned at a thickness of 10 μm. The sections were blocked with 10% goat serum and incubated with primary antibodies against CD31 (1:200; ABclonal, Wuhan, CHN) and against either vascular cell adhesion molecule‐1 (VCAM‐1, 1:200; Abcam, Cambridge, UK) or P‐selectin (1:80; Santa Cruz, CA, USA), respectively. Detection was performed using Alexa Fluor 488‐conjugated goat anti‐mouse and Alexa Fluor 594‐conjugated goat anti‐rabbit secondary antibodies (1:200; Proteintech, Wuhan, China). For immunoglobulin G (IgG) staining, Alexa Fluor 488‐conjugated goat anti‐mouse IgG (1:200; Proteintech) was used. Autofluorescent lipofuscin was assessed by capturing specific regions of interest using excitation light at 480 nm. Cell nuclei were visualized using DAPI staining (Beyotime Biotechnology). Mean fluorescence intensity (MFI) was quantified using Image J software.

### 2.9 ELISA Detection

Brain tissues were homogenized in 0.01 M PBS (*pH* 7.4) with a protease inhibitor cocktail (1:100 (v/v); MCE) using a FastPrep‐96 high‐throughput homogenizer (MP Biomedicals, CA, USA). The homogenates were centrifuged at 12,000 × *g* for 15 min at 4°C to eliminate cellular debris. Supernatants were collected, and the total protein concentration was determined using a BCA protein assay kit (Beyotime Biotechnology). Cytokine levels were assessed using ELISA kits (CUSABIO, Wuhan, CHN) for mouse IL‐1β, IL‐6, tumor necrosis factor‐α (TNF‐α), and IL‐10. In brief, equal amounts of protein samples were added to antibody‐precoated 96‐well plates and incubated for 2 h at room temperature. Subsequently, horseradish peroxidase‐conjugated streptavidin was added and incubated for 1 h at 37°C. The enzymatic reaction was developed using tetramethylbenzidine substrate solution for 15 min in the dark and subsequently stopped with 2 M sulfuric acid. Absorbance was measured at 450 nm using a microplate reader. Cytokine levels in the samples were calculated as relative titers based on the standard curve.

### 2.10 Cell Culture and Viability Analysis

The mouse brain endothelial cell line (*bEnd.3* cells, Procell Life Science & Technology Co. Ltd., Wuhan, CHN) and primary endothelial cells (Procell Life Science & Technology Co.) were used in this study. The *bEnd.3* cells were passaged for no more than 20 generations, while the primary endothelial cells were used at the second generation for the experiment. Cells were cultured in Dulbecco's modified Eagle's medium (DMEM) supplemented with 10% fetal bovine serum (FBS) at 37°C with 5% CO_2_. The culture medium was replenished every 2 to 3 days. Upon reaching 80% confluence, cells were harvested and seeded onto multi‐well plates for subsequent analyses.

To determine the optimal working concentrations, cell viability assessments were conducted on *bEnd.3* and primary endothelial cells under various treatment conditions. The cells were exposed to a dilution series of lupus serum (0%, 2.5%, 5%, 10%, 20%) and, in separate experiments, to a range of SQDHD concentrations (0, 50, 100, 200, 400, 800 μg/mL) under normal conditions. For treatments involving Alisol B Acetate (MCE) and Hederagenin (MCE), *bEnd.3* and primary endothelial cells received six concentrations of either Alisol B Acetate (0, 5, 10, 20, 40, 80 μM) or Hederagenin (0, 10, 20, 40, 80, 160 μM). Following treatment, 10 μL of CCK‐8 reagent was dispensed into each well, and the cells were incubated for 2 h in the dark. Cell viability was assessed by measuring the absorbance at 450 nm. Subsequently, the cells were pretreated with serum‐free culture medium, SQDHD, Upadacitinib (1 μM), SQDHD + Colivelin (1 μM, MCE), Alisol B Acetate or Hederagenin for 2 h. After pretreatment, serum from either control or PIL mice was introduced, and the cells were harvested 24 h later.

### 2.11 Western Blot

Total proteins from brain samples were lysed using RIPA Lysis Buffer, then extracted with the T‐PER protein extraction kit (WLA019, wanleibio, SY, CHN) according to the manufacturer's instructions. The cells were homogenized and centrifuged to obtain supernatants. Protein concentrations were assessed using a BCA protein assay kit. Equal amounts of protein samples were separated on either 10% or 12.5% SDS‐PAGE gels and subsequently transferred to PVDF membranes. The membranes were blocked with 5% non‐fat milk for 2 h and incubated overnight at 4°C with the following primary antibodies: VCAM‐1 (1:2000), P‐selectin (1:500), JAK1 (1:1000; Cell Signaling Technology, Boston, USA), p‐JAK1 (1:1000; Cell Signaling Technology), STAT3 (1:2000, Cell Signaling Technology) and p‐STAT3 (1:2000, Cell Signaling Technology). Following this, the membranes were incubated with a horseradish peroxidase‐conjugated IgG secondary antibody (1:10000; Proteintech, Wuhan, CHN) at room temperature for 2 h. Protein bands were visualized using an ECL chemiluminescence kit (absin, Shanghai, CHN) and then imaged with a luminescent image analyzer (cytiva, Tokyo, JPN). Protein expression levels were quantified using ImageJ software and normalized to β‐actin.

### 2.12 Quantitative Real‐Time Polymerase Chain Reaction (qRT‐PCR)

The expression levels of VCAM‐1 and P‐selectin in brain tissue were assessed by qRT‐PCR. Total RNA was extracted from homogenized brain tissue using the TRI pure Reagent assay kit (RP1001, BioTeke, Beijing, CHN) in accordance with the manufacturer's instructions. RNA from each group was reverse‐transcribed with a reverse transcription kit (PR6502, BioTeke, Beijing, CHN) to generate the corresponding cDNA. Gene expression analysis was conducted on an Exicycler 96 Real‐Time PCR System (BIONEER, Daejeon, ROK) using a SYBR Green kit (SY1020, Solarbio, Beijing, CHN). mRNA levels were normalized to the control (β‐actin). Each sample was analyzed in triplicate, and the average value was recorded. The sequences of the primers utilized for qRT‐PCR are listed in Table 1.

**TABLE 1:** Primers of qRT–PCR analysis (5′ → 3′).

Gene	Forward primer	Reverse primer
VCAM‐1	5′‐GTGGAAATGTGCCCGAAAC‐3′	5′‐GCCTGGCGGATGGTGTA‐3′
P‐selectin	5′‐GGTCCGTCTGTCCCGTAA‐3′	5′‐CCCACTGGGCTCTGTCTTC‐3′
β‐Actin	5′‐GTGCTATGTTGCTCTAGACTTCG‐3′	5′‐ATGCCACAGGATTCCATACC‐3′

### 2.13 Network Pharmacology Analysis

Potential target genes associated with NPSLE were retrieved from multiple online databases, including GeneCards (https://www.genecards.org/), Disease Gene Network (https://www.disgenet.org/), and Comparative Toxicogenomics Database (https://ctdbase.org/). Human gene entries were screened, standardized, and deduplicated using the Uniprot Database (https://www.uniprot.org). The active components of SQDHD were identified by integrating UPLC‐MS/MS analysis with a comprehensive relevant review. Potential target genes of these components were predicted using the STITCH database (http://stitch.embl.de/) and Swiss Target Prediction database (http://www.swisstargetprediction.ch/), with gene nomenclature validated and standardized via the UniProt database. To investigate the mechanism of SQDHD in treating NPSLE, a Venn diagram was utilized to identify overlapping targets between NPSLE‐related genes and the predicted targets of SQDHD's active components. The overlapping targets were then analyzed via the STRING database (https://cn.string‐db.org/) to construct a protein–protein interaction (PPI) network, applying a minimum interaction score threshold of 0.70. The PPI network was visualized using Cytoscape 3.7.1 software. Within Cytoscape, the cytoHubba plugin was utilized to rank the targets according to their degree centrality, from which the top 15 targets were selected for further analysis. Functional enrichment analysis for these top 15 targets was conducted using the Database for Annotation, Visualization, and Integrated Discovery (DAVID) database (https://david.ncifcrf.gov/). Gene Ontology (GO) and Kyoto encyclopedia of genes and genomes (KEGG) pathway analyses were performed to identify significantly enriched biological processes (BP), cellular components (CC), molecular functions (MF), and signaling pathways. The results from the GO and KEGG analyses were visualized using an online platform (https://www.bioinformatics.com.cn/). To construct a compound‐target (CT) network, data on the active components of SQDHD and the overlapping targets of SQDHD against NPSLE were integrated using Cytoscape. The primary active components of SQDHD against NPSLE were identified as those exhibiting the highest degree values connected to the top 15 targets.

### 2.14 Molecular Docking

The 2‐dimensional (2D) structures of the primary active components were retrieved from the PubChem database (https://pubchem.ncbi.nlm.nih.gov/). The 3D crystal structures of target proteins, including JAK1 (PDB code: 4FK6), AKT1 (PDB code: 3O96), EGFR (PDB code: 3LZB), CSF2 (PDB code: 2GMF), IL‐6 (PDB code: 1ALU), IL‐4 (PDB code: 1HZI), IL‐2 (PDB code: 1 M47), IL‐10 (PDB code: 1INR), and STAT3 (PDB code: 6TLC), were retrieved from the Research Collaboratory for Structural Bioinformatics (RCSB) Protein Data Bank (PDB) database (https://www.rcsb.org/). Using PyMOL 2.6.0, irrelevant ligands and water molecules were removed from the crystal structures to prepare the protein receptors for molecular docking. Subsequently, AutoDock Tools 1.5.6 was employed to add hydrogen atoms, thereby optimizing the docking environment. Molecular docking was performed using AutoDock Vina 1.1.2 to calculate the binding affinities (docking scores), where lower scores represented more stable and favorable interactions. The docking results were visualized using Origin 2024 software. From these, the JAK1‐ligand complex that occupied the same binding site as, or a site proximal to, that of Upadacitinib was selected for visualization in PyMOL.

### 2.15 CETSA

*bEnd.3* cells were treated with RIPA lysis buffer on ice for 15 min to induce cell lysis. After centrifugation, the supernatant was collected. The supernatant was then incubated with Alisol B Acetate, Hederagenin, Ellagic acid (MCE), Wogonin (MCE), Quercetin (MCE), or dimethyl sulfoxide (DMSO) for 2 h at room temperature. The samples were subjected to heating across a temperature ranging from 40°C to 65°C for 3 min. Following the thermal challenge, the samples were combined with 5× loading buffer and denatured by boiling at 95°C for 10 min. Finally, the remaining soluble JAK1 protein in each sample was detected by Western blot analysis.

### 2.16 DARTS Assay

The supernatant of the *bEnd.3* cell lysate was collected according to the CETSA protocol. The supernatant was then incubated at 4°C for 2 h with either 0.5% DMSO or a range of concentrations of the indicated compounds: Alisol B Acetate (2.5, 5, 10 μM), Hederagenin (5, 10, 20 μM), Ellagic acid (2.5, 5, 10 μM), Wogonin (5, 10, 20 μM), and Quercetin (5, 10, 20 μM). Following this, the samples were either digested with pronase (at a 1:100 ratio of pronase to protein) or left undigested for 10 min at room temperature. The reactions were immediately terminated by the addition of loading buffer, and the samples were boiled for 5 min to denature proteins prior to Western blot analysis.

### 2.17 Statistical Analysis

Statistical analyses were performed using GraphPad Prism V8 software (La Jolla, CA, USA). Prior to applying parametric statistical tests, the normality of the data were assessed using the *D'Agostino‐Pearson omnibus normality test*. Data are presented as mean ± standard error of the mean (SEM). Statistical significance was assessed using *one‐way analysis of variance (ANOVA)* followed by *Tukey's post hoc test* for multiple comparisons or *multiple t‐tests with the Holm‐Sidak correction* for pairwise comparisons. *p* < 0.05 was considered statistically significant.

## 3 Results

### 3.1 SQDHD Ameliorates Behavioral Deficits in PIL Mice

**FIGURE 1:**
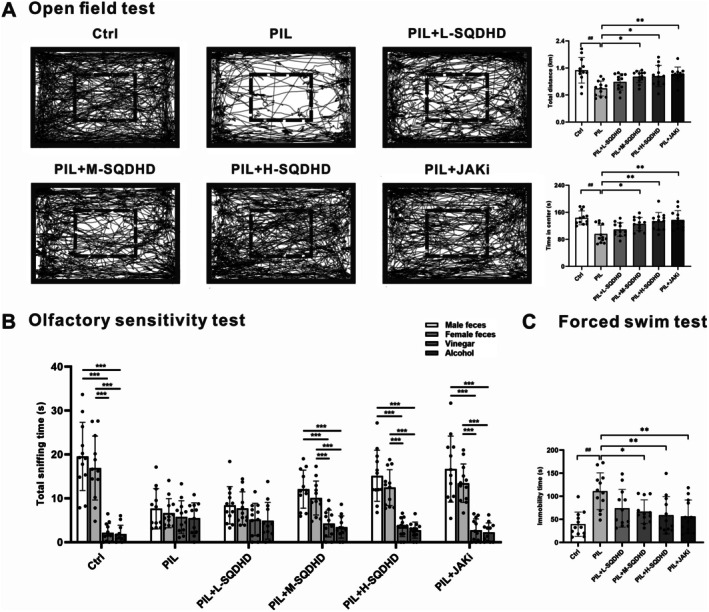
Effects of SQDHD on behavioral deficits in PIL mice. (A) Open field test. Left panel: Representative images of the traveled path. Right panel: Quantitative analysis of total distance (km) and time spent in the center (s). (B) Olfactory sensitivity test. Quantitative analysis of total sniffing time for male feces, female feces, vinegar or alcohol. (C) Forced swim test. Quantitative analysis of immobility time (s). Data are expressed as mean ± SEM (*n* = 12 per group). Statistical analysis was conducted using *One‐way ANOVA* followed by *Tukey's post hoc test*: ##*p* < 0.01 vs. Ctrl group, *p < 0.05 or **p < 0.01 vs. PIL group. Multiple *t*‐tests followed by *Holm‐Sidak correction*: ***p < 0.01 for comparisons between two groups.

To assess the therapeutic impact of SQDHD on behavioral deficits in PIL mice, we administered a battery of behavioral tests. In the open field test, PIL mice displayed anxiety‐like behavior, as indicated by a significantly reduced total distance traveled and in time spent in the center compared to control mice (Figure 1A). Treatment with M‐SQDHD, H‐SQDHD, or JAKi significantly mitigated the anxiety‐like behavior in PIL mice (Figure 1A). The olfactory sensitivity test revealed that control mice exhibited a strong preference for both female and male fecal odors over vinegar and alcohol, spending significantly more time investigating the fecal scents (Figure 1B). However, 4 months after model establishment, PIL mice failed to display this preference for fecal odors (Figure 1B). Remarkably, treatment with M‐SQDHD, H‐SQDHD, or JAKi restored this preference for fecal odors, suggesting an improvement in olfactory function (Figure 1B). Furthermore, in the forced swim test, PIL mice showed a significantly prolonged immobility time compared to controls, suggestive of depression‐like behavior (Figure 1C). Treatment with M‐SQDHD, H‐SQDHD, or JAKi resulted in a significant reduction in the immobility duration of PIL mice in the forced swim test (Figure 1C). Collectively, these findings suggest that SQDHD, particularly at its medium and high doses, exerts therapeutic effects comparable to JAKi in ameliorating olfactory dysfunction, anxiety‐like, and depression‐like behavioral deficits in PIL mice.

### 3.2 SQDHD Suppresses Adhesion Molecule Expression, Leukocyte Recruitment, and BBB Disruption in PIL Mice

**FIGURE 2:**
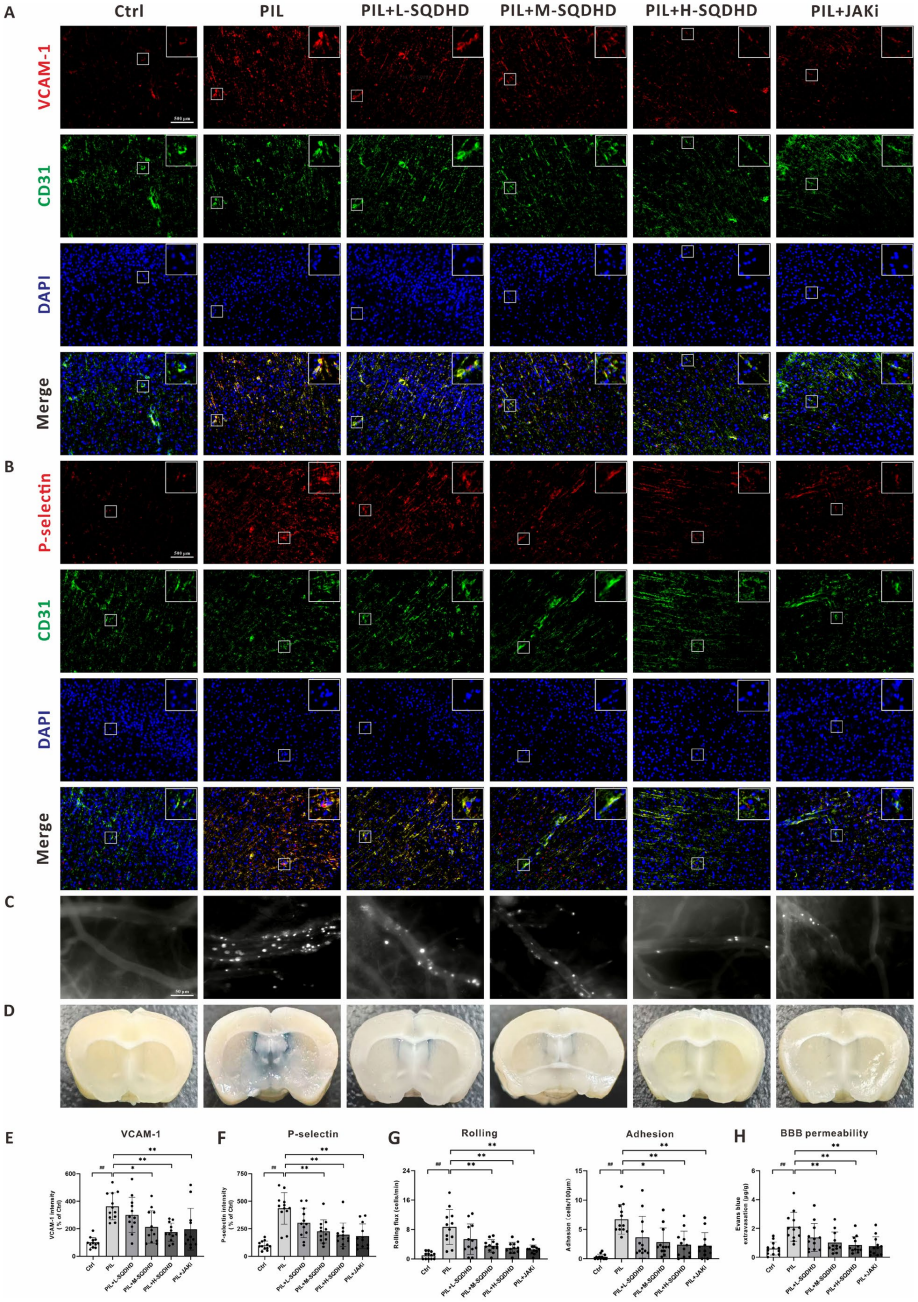
Effects of SQDHD on adhesion molecules, leukocyte recruitment and BBB leakage. (A, B) Representative images of VCAM‐1 and P‐selectin expression. White square showing the co‐localization of enlarged VCAM‐1 or P‐selectin (red) with CD31 (green). DAPI staining for nuclei (blue). (C) Representative images of leukocyte rolling and adhesion. (D) Representative images of Evans blue dye extravasation across the BBB. (E, F) Quantitative analysis of VCAM‐1 and P‐selectin expression, measured by MFI and normalized to the Ctrl group. (G) Quantitative analysis of leukocyte rolling and adhesion. (H) Quantitative analysis of Evans blue dye extravasation. Data are expressed as mean ± SEM (*n* = 12 per group). Statistical analysis was conducted using *One‐way ANOVA* followed by *Tukey's post hoc test*: ##*p* < 0.01 vs. Ctrl group, *p < 0.05 or **p < 0.01 vs. PIL group.

The activation of the cerebral endothelium, characterized by the upregulation of adhesion molecules, is a critical initial driver of neuroinflammation, facilitating the recruitment of circulating leukocytes and the disruption of the BBB [30]. To investigate the effect of SQDHD on endothelial activation, we assessed the expression of adhesion molecules VCAM‐1 and P‐selectin in the cerebral cortex by immunofluorescence staining. For a comprehensive visualization of the vascular architecture, we conducted co‐staining of VCAM‐1 or P‐selectin with the endothelial marker CD31 (Figure 2A,B). DAPI staining was employed to indicate their specific locations within the cortex and to elucidate the anatomical context (Figure 2A,B). As shown in Figure 2A,B and Figure 2E,F, PIL mice exhibited a significant upregulation of these markers compared to controls. Importantly, treatment with M‐SQDHD, H‐SQDHD or JAKi effectively rescued the disease‐induced elevation in VCAM‐1 and P‐selectin (Figure 2A,B,E,F). To directly visualize leukocyte‐endothelial interactions within cerebral vessels, we performed intravital microscopy [31]. Few rolling or adherent leukocytes were observed in the cerebral vessels of control mice, whereas PIL mice exhibited a marked increase in these cells (Figure 2C,G). Treatment with M‐SQDHD, H‐SQDHD, or JAKi significantly attenuated leukocyte rolling and adhesion in PIL mice (Figure 2C,G). Additionally, PIL mice showed significant BBB leakage, as indicated by elevated Evans blue content in brain tissues (Figure 2D,H). Following treatment with M‐SQDHD, H‐SQDHD, or JAKi, we observed a significant decrease in Evans blue extravasation (Figure 2D,H).

### 3.3 SQDHD Decreases Cytokine Overexpression, IgG Deposition and Lipofuscin Accumulation in PIL Mice

**FIGURE 3:**
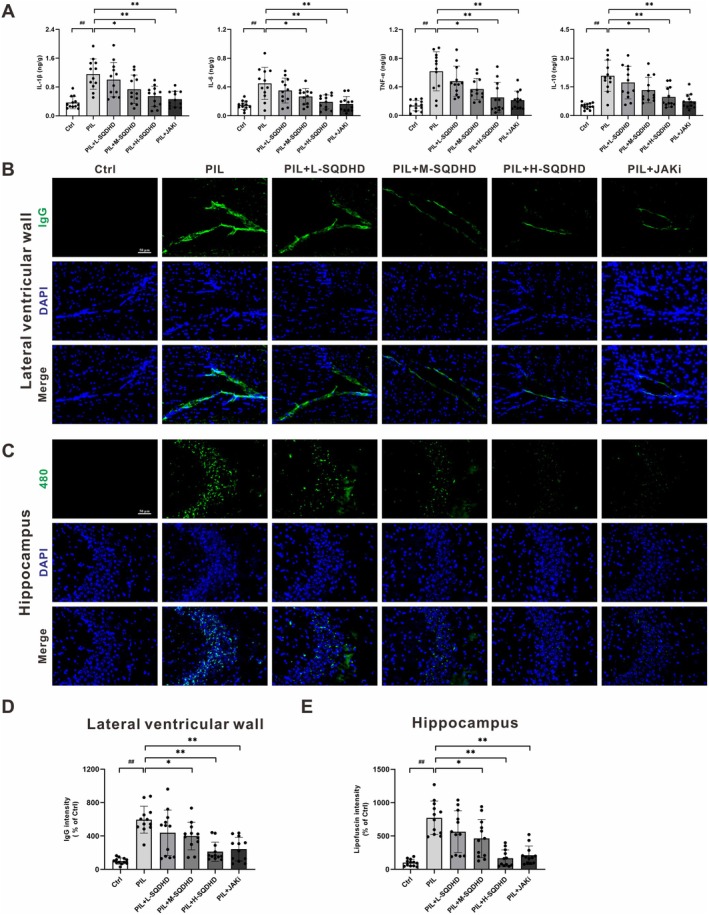
Effects of SQDHD on cytokine expression, IgG deposition and lipofuscin accumulation. (A) Quantitative analysis of brain cytokine expression (IL‐1β, IL‐6, TNF‐α, and IL‐10). (B) Representative images of IgG deposition (green) in the lateral ventricular wall. DAPI staining (blue) for nuclei. (C) Representative images of autofluorescent lipofuscin (excitation at 480 nm, green) in the hippocampus. DAPI staining (blue) for nuclei. (D) Quantitative analysis of IgG deposition in the lateral ventricular wall (measured by MFI and normalized to the Ctrl group). (E) Quantitative analysis of lipofuscin foci in the hippocampus (measured by MFI and normalized to the Ctrl group). Data are expressed as mean ± SEM (*n* = 12 per group). Statistical analysis was conducted using *One‐way ANOVA* followed by *Tukey's post hoc test*: ^##^ *p* < 0.01 vs. Ctrl group, *p < 0.05 or **p < 0.01 vs. PIL group.

To evaluate the impact of SQDHD on brain pathophysiological alterations in PIL mice, we first assessed cytokine expression in brain tissue. PIL mice exhibited significantly elevated levels of IL‐1β, IL‐6, TNF‐α, and IL‐10 compared to control mice (Figure 3A). SQDHD treatment induced a dose‐dependent reduction in these cytokine levels, with H‐SQDHD demonstrating an inhibitory effect comparable to that of JAKi (Figure 3A). Immunofluorescence staining revealed significant IgG deposition in the lateral ventricular wall of PIL mice, which was significantly attenuated by treatment with M‐SQDHD, H‐SQDHD, or JAKi (Figure 3B,D). Additionally, PIL mice exhibited a notable accumulation of lipofuscin in the hippocampus, which was significantly diminished by treatment with M‐SQDHD, H‐SQDHD, or JAKi (Figure 3C,E). Collectively, these findings indicate that SQDHD treatment effectively mitigates inflammatory neuropathological changes in PIL mice.

### 3.4 The JAK–STAT Pathway is Predicted as the Critical Mechanism of SQDHD Against NPSLE

**FIGURE 4:**
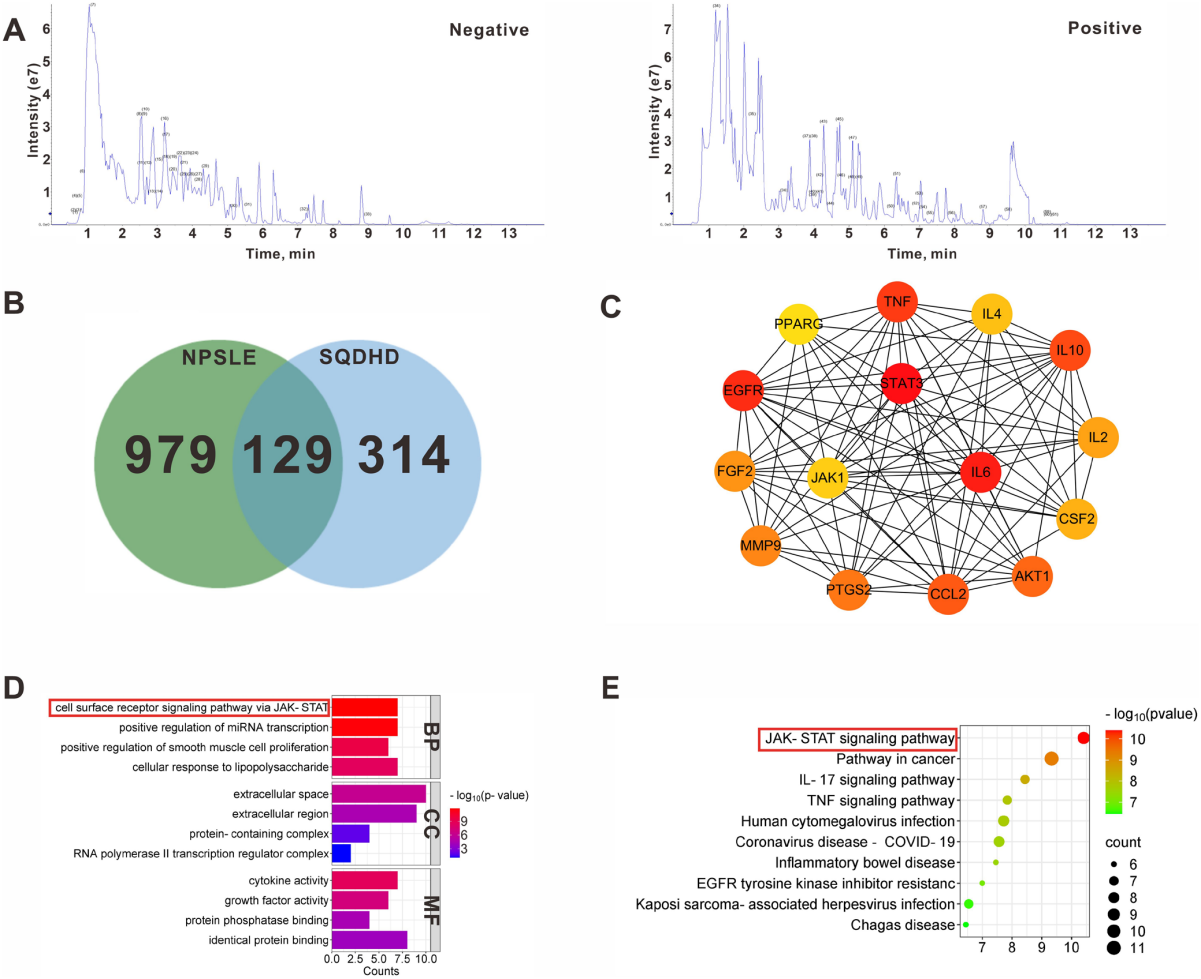
Analysis of the therapeutic mechanism of SQDHD against NPSLE. (A) Identification of chemical components in SQDHD, represented by TICs in both negative and positive ion modes. The numbers indicate the locations of the 61 active components. (B) Venn diagram. Identification of overlapping targets between SQDHD and NPSLE. (C) PPI network. Top 15 targets of SQDHD against NPSLE. (D, E) GO and KEGG pathway analyses. Functional and pathway enrichment of the top 15 targets.

To identify the specific therapeutic components of SQDHD, we conducted a comprehensive analysis utilizing UPLC‐MS/MS. The total ion chromatograms (TICs) of the SQDHD sample, examined in both negative and positive modes, revealed a vast array of components (Figure 4A). Based on the UPLC‐MS/MS data and relevant literature, we selected 61 active compounds in SQDHD for network pharmacology analysis (Tables S1 and S2). Following database predictions and the removal of duplicate values, we identified 1108 targets associated with NPSLE and 443 targets related to the active compounds (Tables S3 and S4). By integrating these two target sets, we obtained a total of 129 overlapping targets (Figure 4B and Table S5). Analysis of the PPI network constructed from these 129 overlapping targets revealed the top 15 targets, including EGFR, STAT3, IL‐6, TNF, IL‐10, CCL2, PTGS2, MMP9, AKT1, FGF2, IL‐2, CSF2, IL‐4, JAK1, and PPARG, as potentially critical effector targets of SQDHD against NPSLE (Figure 4C). To further elucidate the biological functions of these targets, we performed GO and KEGG pathway analyses using the DAVID database. The results of the GO and KEGG analyses underscored the significant involvement of cytokine activity and the JAK–STAT signaling pathway (Figure 4D,E and Table S6). Based on the PPI network and GO/KEGG analyses, we speculate that the JAK1‐STAT3 signaling pathway may serve as the critical mechanism underlying the therapeutic effects of SQDHD in NPSLE.

### 3.5 Analysis of the Primary Active Components in SQDHD Against NPSLE

**FIGURE 5:**
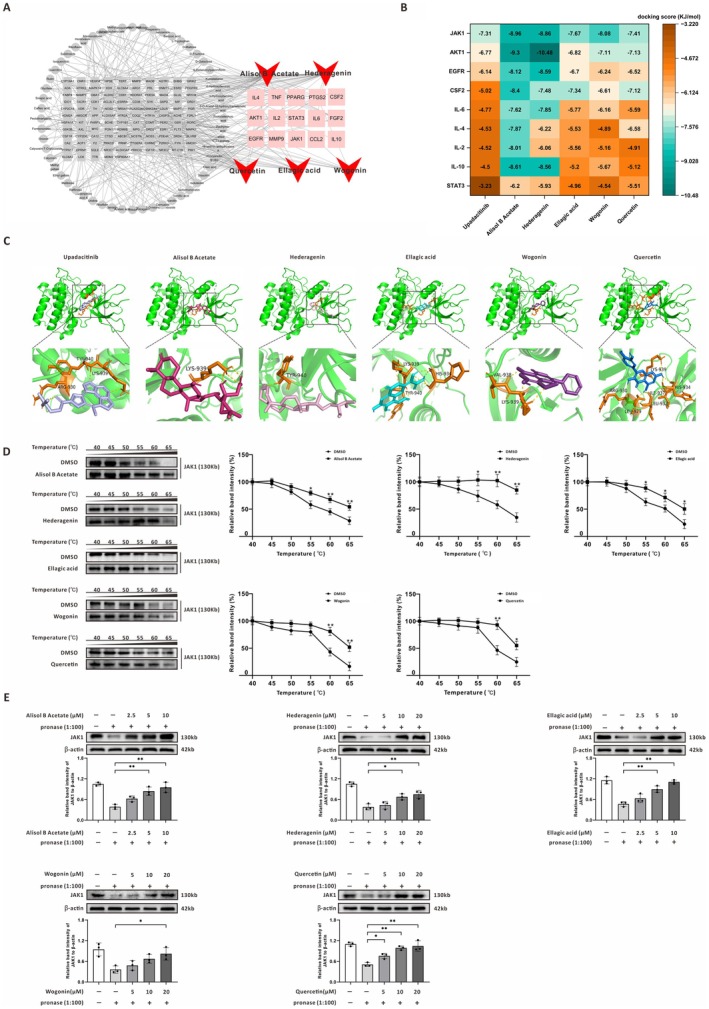
Analysis and identification of the primary active compounds of SQDHD against NPSLE. (A) CT network. Red denotes the five primary active compounds of SQDHD (Alisol B Acetate, Hederagenin, Ellagic acid, Wogonin, and Quercetin) with the highest degree values. Pink denotes the top 15 targets. Gray denotes compounds with lower degree values with their associated targets. (B) Heatmap of docking scores. Identification of the binding affinities between the five primary active compounds, Upadacitinib, and the nine targets enriched in the JAK–STAT pathway. (C) Representative images of docking complexes. The 3D structures of JAK1 in complex with the five primary active compounds and Upadacitinib at the same or proximal binding sites. (D) Representative western blot bands from CETSA. Quantitative analysis of JAK1. (E) Representative western blot bands from DARTS assay. Quantitative analysis of JAK1. Data are expressed as mean ± SEM (*n* = 3 per group). Statistical analysis was conducted using *One‐way ANOVA followed by Tukey's post hoc* test. *p < 0.05 or **p < 0.01.

To elucidate the intricate relationship between compounds and their targets, we constructed a network topology analysis. This analysis revealed five primary active components in SQDHD, including Alisol B Acetate, Hederagenin, Ellagic acid, Wogonin, and Quercetin, all of which exhibited the highest degree values associated with the top 15 targets within the CT network (Figure 5A and Table S7). Among these targets, nine targets (JAK1, AKT1, EGFR, CSF2, IL‐6, IL‐4, IL‐2, IL‐10, and STAT3) were enriched in the JAK–STAT signaling pathway (Table S6). We further employed molecular docking to evaluate the potential interactions between the five primary active components and Upadacitinib with the nine targets. The targets were ranked according to their docking scores with Upadacitinib. Notably, the five primary active components of SQDHD exhibited even stronger binding affinities to JAK1, with docking scores lower than −7.31 (Figure 5B), and they occupied the same or proximal binding sites as Upadacitinib on JAK1 (Figure 5C). Furthermore, the five primary active components, particularly Alisol B Acetate and Hederagenin, exhibited significant binding affinities to other targets enriched in the JAK–STAT signaling pathway. Thus, in contrast to Upadacitinib's single‐target inhibition, these active components may exert anti‐inflammatory effects by targeting multiple targets within the JAK–STAT signaling pathway. To investigate the potential interaction between the five primary active compounds and JAK1, we initially employed the CETSA. The results indicated that compound‐treated JAK1 exhibited significantly enhanced thermal stability compared to the control group (Figure 5D), suggesting direct binding. This finding was further corroborated by a DARTS experiment, which operates on the principle that target proteins become more resistant to proteolysis upon ligand binding. Indeed, JAK1 protein from the compound‐treated group displayed markedly greater resistance to pronase digestion than that of the control (Figure 5E). Collectively, these findings from two independent experimental approaches provide direct evidence of a physical interaction between the five active compounds of SQDHD and the JAK1 protein.

### 3.6 SQDHD Inhibits CVEC Activation by Blocking the JAK1‐STAT3 Pathway

**FIGURE 6:**
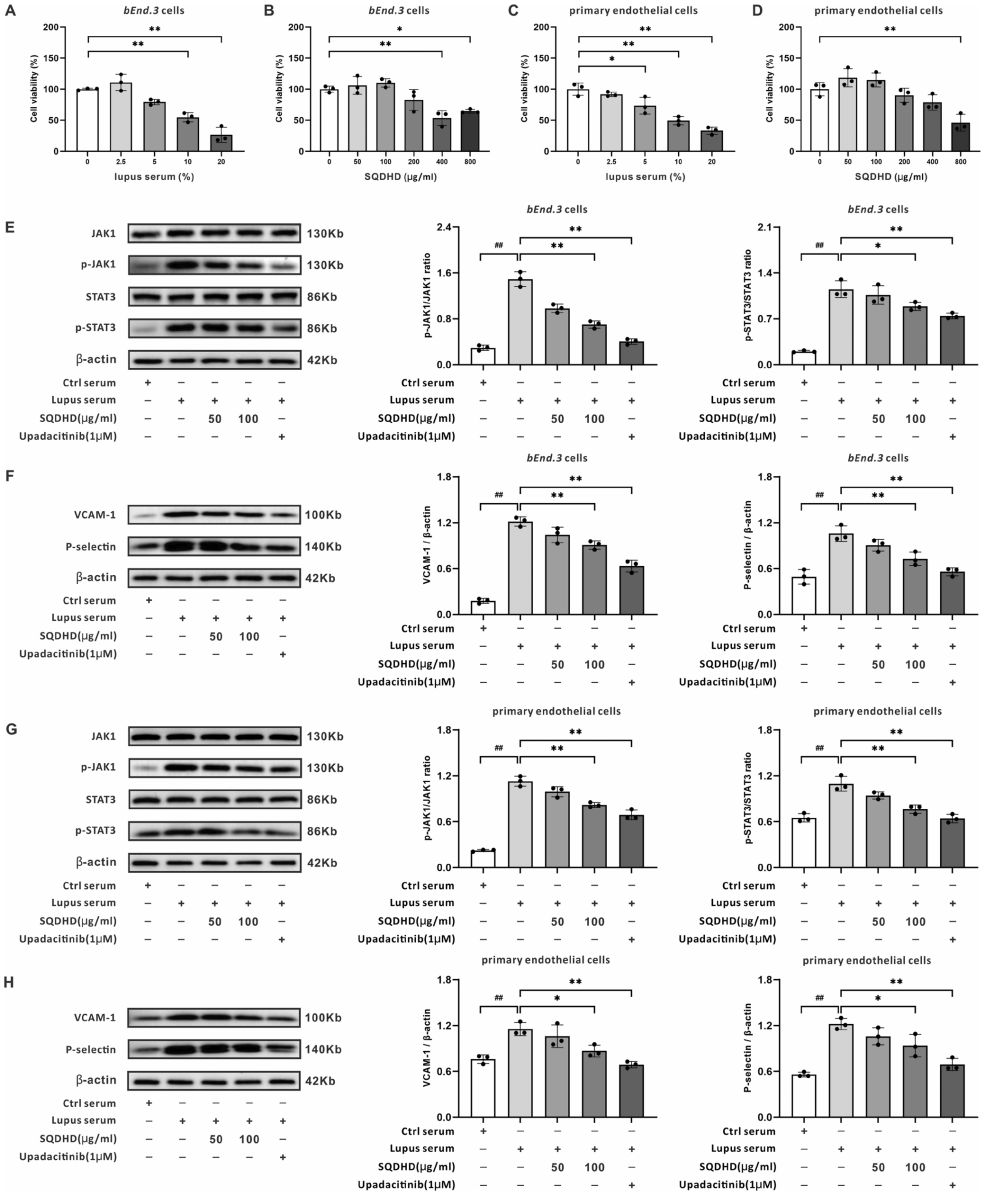
Effects and mechanisms of SQDHD in *bEnd.3* cells and primary endothelial cells induced by lupus serum. (A–D) Cell viability assessment. Quantitative analysis of varying concentrations of lupus serum or SQDHD in *bEnd.3* cells or primary endothelial cells. (E) Representative western blot bands for JAK1, p‐JAK1, STAT3, and p‐STAT3 in *bEnd.3* cells. Quantitative analysis of the ratios of p‐JAK1/JAK1 and p‐STAT3/STAT3 in *bEnd.3* cells. (F) Representative western blot bands and quantitative analysis of VCAM‐1 and P‐selectin in *bEnd.3* cells. (G) Representative western blot bands for JAK1, p‐JAK1, STAT3, and p‐STAT3 in primary endothelial cells. Quantitative analysis of the ratios of p‐JAK1/JAK1 and p‐STAT3/STAT3 in primary endothelial cells. (H) Representative western blot bands and quantitative analysis of VCAM‐1 and P‐selectin in primary endothelial cells. Data are expressed as mean ± SEM (*n* = 3 per group). Statistical analysis was conducted using *One‐way ANOVA* followed by *Tukey's post hoc* test. Cell viability assessment: *p < 0.05 or **p < 0.01 vs. 0% lupus serum or 0 μg/mL SQDHD. Western blot test: ##*p* < 0.01 vs. Ctrl serum group, *p < 0.05 or **p < 0.01 vs. lupus serum group.

We assessed the inhibitory effect of SQDHD on the JAK1‐STAT3 signaling pathway in CVECs upon lupus serum stimulation. Initially, CCK8 assays indicated that 10% lupus serum was optimal for cell stimulation (Figure 6A,C), and concentrations of SQDHD at 50 μg/mL and 100 μg/mL were appropriate for in vitro treatment (Figure 6B,D). Subsequently, we assessed the effects of SQDHD on the phosphorylation levels of the JAK1‐STAT3 signaling pathway, as well as the expression of VCAM‐1 and P‐selectin in *bEnd.3* cells. Lupus serum significantly increased the phosphorylation levels of JAK1 and STAT3 compared to control serum, while pre‐treatment with SQDHD (100 μg/mL) or Upadacitinib (1 μM) effectively inhibited the phosphorylation of JAK1 and STAT3 (Figure 6E). Furthermore, treatment with lupus serum significantly upregulated the expression of VCAM‐1 and P‐selectin compared to control serum, demonstrating that *bEnd.3* cells can be activated by lupus serum (Figure 6F). This upregulation was significantly attenuated by pre‐treatment with SQDHD (100 μg/mL) or Upadacitinib (Figure 6F). This inhibitory profile of SQDHD was consistently observed in primary endothelial cells, confirming that its effects on the JAK1‐STAT3 pathway and downstream adhesion molecules are reproducible across different cell types (Figure 6G,H).

**FIGURE 7:**
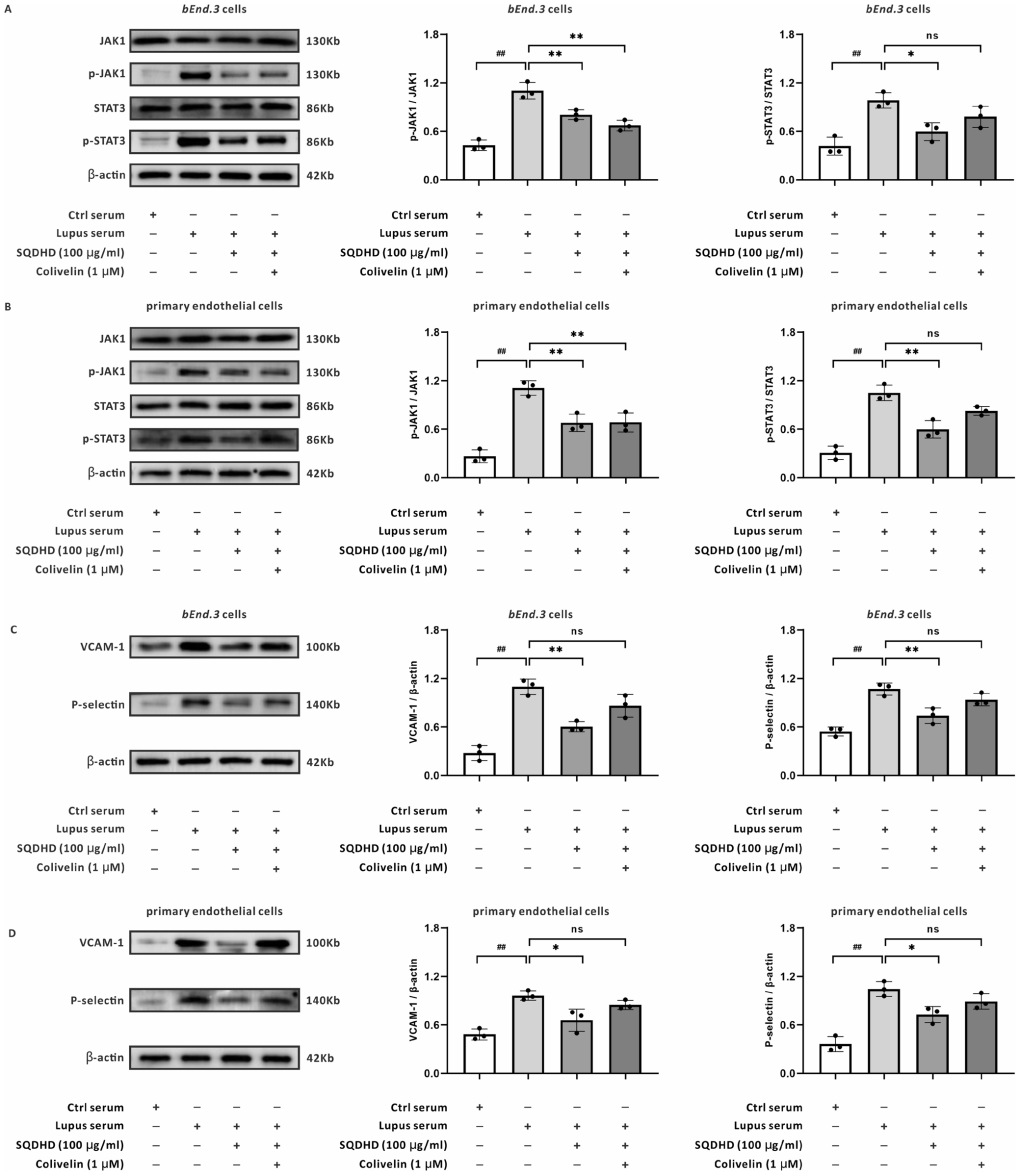
Validation of JAK1‐STAT3 pathway dependency in SQDHD's anti‐neuroinflammatory mechanism. (A, B) Representative western blot bands for JAK1, p‐JAK1, STAT3, and p‐STAT3 in *bEnd.3* cells or primary endothelial cells. Quantitative analysis of the ratios of p‐JAK1/JAK1 and p‐STAT3/STAT3. (C, D) Representative western blot bands and quantitative analysis of VCAM‐1 and P‐selectin in *bEnd.3* cells or primary endothelial cells. Data are expressed as mean ± SEM (*n* = 3 per group). Statistical analysis was conducted using *One‐way ANOVA* followed by *Tukey's post hoc* test. ##*p* < 0.01 vs. Ctrl serum group, *p < 0.05 or **p < 0.01 vs. lupus serum group.

To directly assess whether the anti‐neuroinflammatory efficacy of SQDHD is contingent upon the JAK1‐STAT3 signaling pathway, we conducted a rescue experiment in CVECs. Consistent with prior findings, SQDHD significantly inhibited the activation of the JAK1‐STAT3 pathway (Figure 7A,B) and the upregulation of adhesion molecules induced by lupus serum in CVECs (Figure 7C,D). The rescue experiment provided critical evidence for pathway dependency. Notably, co‐administration of the STAT3 activator Colivelin not only reversed SQDHD's inhibition of STAT3 phosphorylation (Figure 7A,B) but also restored the expression of VCAM‐1 and P‐selectin (Figure 7C,D).

### 3.7 Alisol B Acetate and Hederagenin Attenuate CVEC Activation via JAK1‐STAT3 Suppression

**FIGURE 8:**
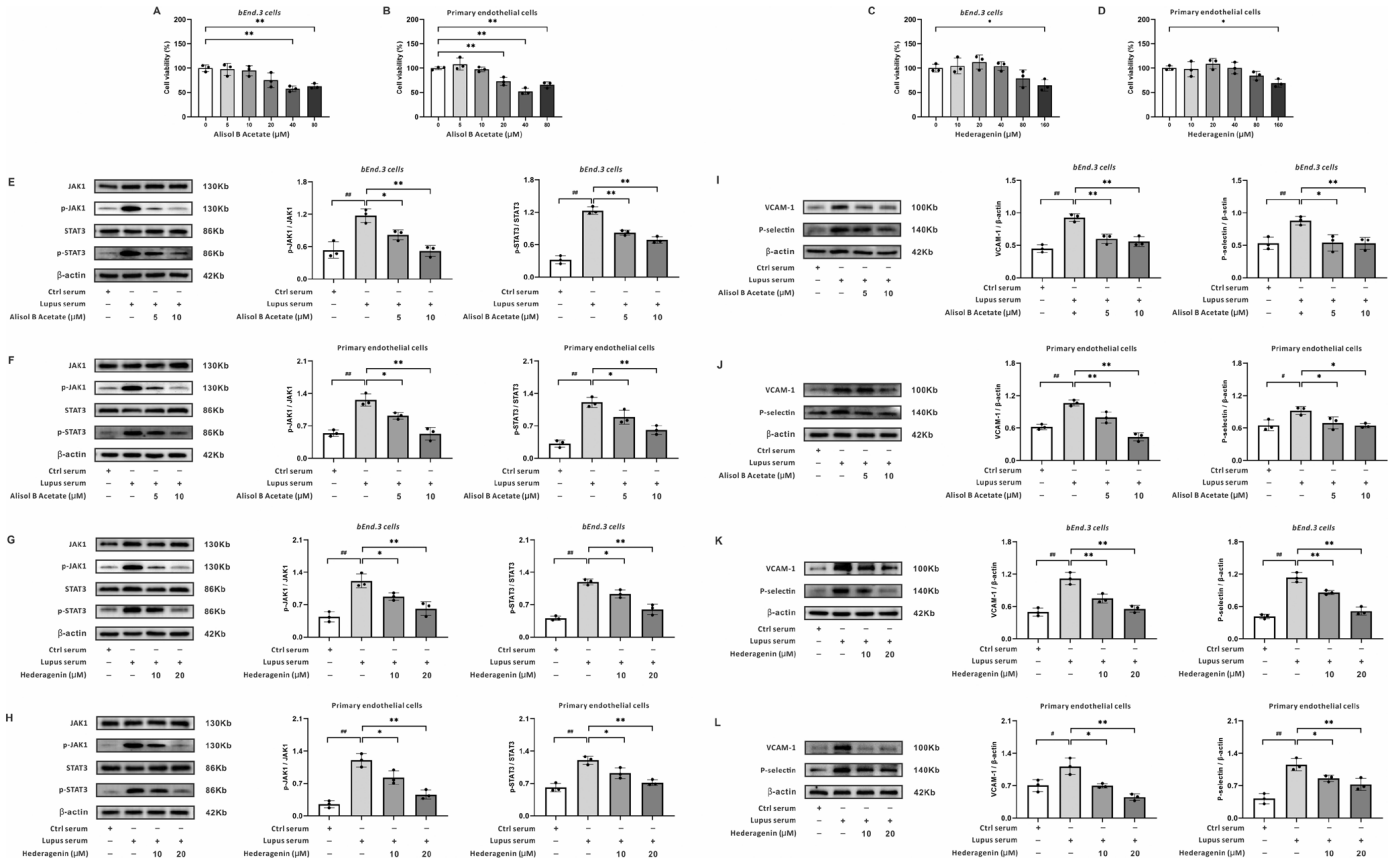
Effects and mechanisms of Alisol B Acetate or Hederagenin on *bEnd.3* cells and primary endothelial cells induced by lupus serum. (A–D) Cell viability assessment. Quantitative analysis of varying concentrations of Alisol B Acetate or Hederagenin in both *bEnd.3* cells and primary endothelial cells. (E–H) Representative western blot bands of JAK1, p‐JAK1, STAT3, and p‐STAT3 in *bEnd.3* cells or primary endothelial cells treated with Alisol B Acetate or Hederagenin. Quantitative analysis of the ratios of p‐JAK1/JAK1 and p‐STAT3/STAT3. (I–L) Representative western blot bands of VCAM‐1 and P‐selectin in *bEnd.3* cells or primary endothelial cells treated with Alisol B Acetate or Hederagenin. Data are expressed as mean ± SEM (*n* = 3 per group). Statistical analysis was conducted using *One‐way ANOVA* followed by *Tukey's post hoc* test. Cell viability assessment: *p < 0.05 or **p < 0.01 vs. 0 μM Alisol B Acetate or 0 μM Hederagenin. Western blot test: ##*p* < 0.01 vs. Ctrl serum group, *p < 0.05 or **p < 0.01 vs. lupus serum group.

To assess whether the top‐scoring docking compounds, Alisol B Acetate and Hederagenin, could replicate the full effects of SQDHD, we evaluated their activity in cellular models of CVEC activation. We first determined non‐cytotoxic concentrations for Alisol B Acetate and Hederagenin using CCK‐8 assays (Figure 8A–D). Subsequent experiments in both *bEnd.3* cells and primary endothelial cells demonstrated that pre‐treatment with either Alisol B Acetate or Hederagenin significantly inhibited lupus serum‐induced phosphorylation of JAK1 and STAT3 (Figure 8E–H). Furthermore, both compounds markedly attenuated the upregulation of the adhesion molecules VCAM‐1 and P‐selectin (Figure 8I–L). Collectively, these results indicate that either Alisol B Acetate or Hederagenin can mimic SQDHD by attenuating CVEC activation through suppression of the JAK1‐STAT3 pathway.

### 3.8 SQDHD Inhibits the JAK1‐STAT3 Pathway in the Brain of PIL Mice

**FIGURE 9:**
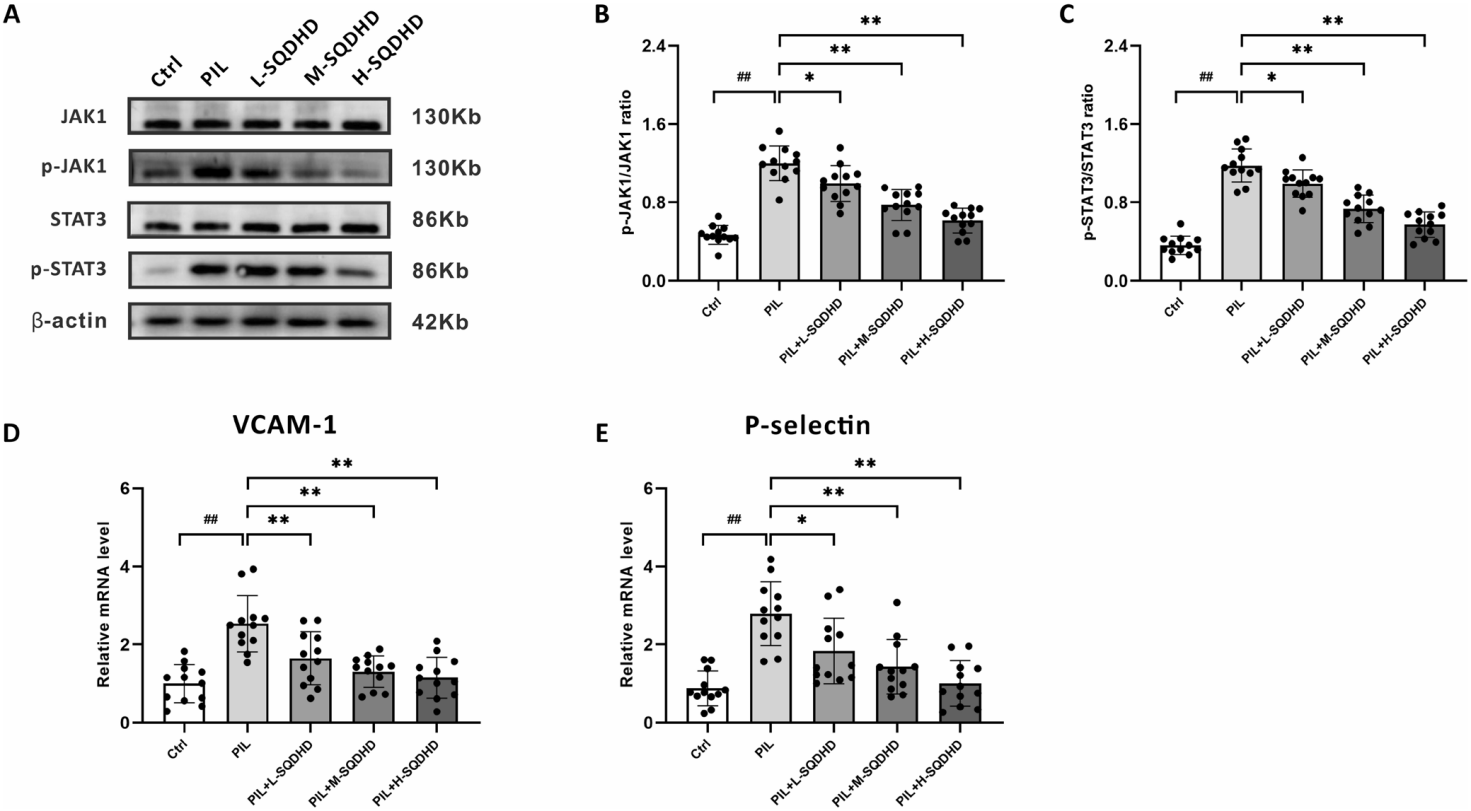
Effects and mechanisms of SQDHD in brain tissue of PIL mice. (A) Representative western blot bands of JAK1, p‐JAK1, STAT3, and p‐STAT3. (B, C) Quantitative analysis of the ratios of p‐JAK1/JAK1 and p‐STAT3/STAT3. (D, E) Quantitative analysis of the mRNA expression of VCAM‐1 and P‐selectin. Data are expressed as mean ± SEM (*n* = 3 per group). Statistical analysis was conducted using *One‐way ANOVA* followed by *Tukey's post hoc* test. ##*p* < 0.01 vs. Ctrl group, *p < 0.05 or **p < 0.01 vs. PIL group.

We assessed the effects of SQDHD on both the phosphorylation levels of JAK1 and STAT3 and the mRNA expression of VCAM‐1 and P‐selectin in the brain tissues of PIL mice. The results indicated that PIL mice exhibited significantly elevated phosphorylation levels of JAK1 and STAT3 compared to control mice, and SQDHD treatment effectively suppressed these increases in a dose‐dependent manner (Figure 9A–C). Moreover, the mRNA expression levels of VCAM‐1 and P‐selectin, key downstream effectors of the JAK–STAT pathway, were significantly upregulated in PIL mice relative to controls, and this upregulation was markedly attenuated by SQDHD treatment (Figure 9D,E). Collectively, these findings suggest that SQDHD exerts its protective effects against NPSLE by inhibiting the phosphorylation of the JAK1‐STAT3 signaling pathway and downregulating its key downstream effector molecules.

## 4 Discussion

**FIGURE 10:**
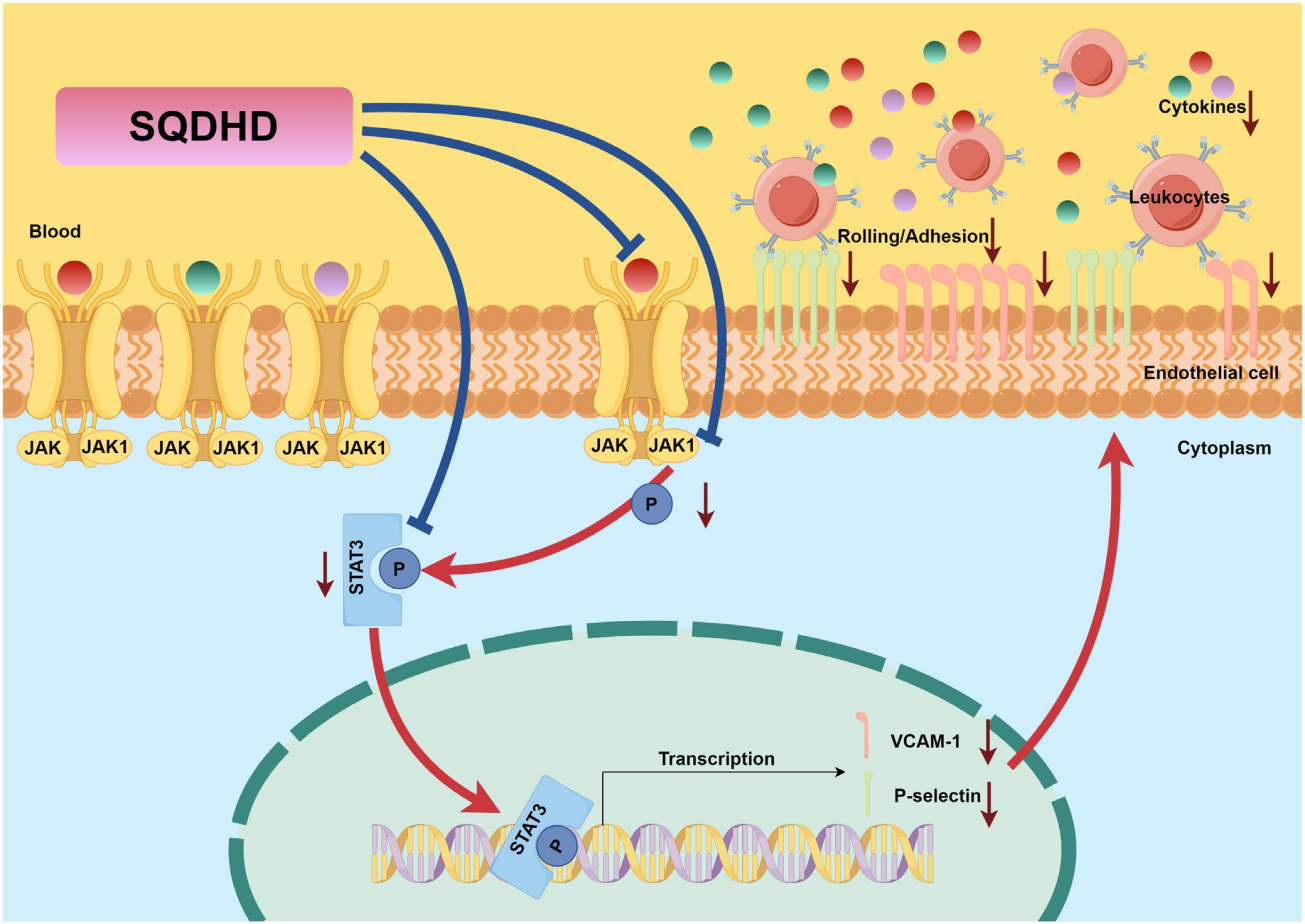
Potential therapeutic mechanism of SQDHD against NPSLE.

This study presents the first demonstration of the therapeutic effects of SQDHD in NPSLE, as evidenced by: (a) significant improvements in olfactory dysfunction, as well as anxiety‐ and depression‐like behaviors in PIL mice; (b) a reduction in the elevation of adhesion molecules (VCAM‐1 and P‐selectin), leukocyte recruitment, and BBB leakage in PIL mice; (c) decreased cytokine overexpression in the brain, IgG deposition in the lateral ventricular wall, and lipofuscin accumulation in the hippocampus of PIL mice; and (d) downregulation of adhesion molecule expression through the inhibition of the JAK1‐STAT3 signaling pathway in both lupus serum‐induced CVECs and PIL mice (Figure 10). Collectively, our findings provide a novel clinical application strategy for SQDHD in the treatment of NPSLE.

TCMs have recently been proposed as complementary and alternative therapies for addressing affective deficits, particularly depression and anxiety, which are prevalent neuropsychiatric disturbances that significantly impair the quality of life in NPSLE patients [32, 33]. For the first time, we demonstrated that SQDHD effectively alleviates anxiety‐ and depression‐like behaviors in PIL mice (Figure 1A,C). Previous studies have indicated that the primary components of SQDHD are associated with improvements in these behaviors [34]. For instance, *Poria cocos* water‐soluble polysaccharide has been shown to ameliorate anxiety‐like behavior by regulating gut dysbiosis, metabolic disorders, and the TNF‐α/nuclear factor‐kappa B (NF‐κB) signaling pathway [35]. Morroniside, derived from *Cornus officinalis* , has exhibited potential in alleviating depressive symptoms via the brain‐derived neurotrophic factor signaling pathway [36]. These findings offer a plausible explanation for the therapeutic benefits of SQDHD on affective deficits in NPSLE. Furthermore, olfactory dysfunction, a common characteristic in both NPSLE patients and animal models [37, 38, 39], was significantly improved by SQDHD treatment in PIL mice (Figure 1B). Previous research has established that olfactory dysfunction is closely associated with affective impairments in NPSLE, likely due to autoantibody deposition in both limbic and olfactory regions [40]. Notably, antidepressant treatment has been shown to alleviate both depressive‐like behaviors and olfactory dysfunction in anti‐ribosomal P‐injected NPSLE mice [37]. Our results reinforce this correlation and underscore the therapeutic potential of SQDHD in addressing behavioral deficits in NPSLE.

One of the key pathogenic mechanisms of NPSLE involves disrupting the BBB through the infiltration of proinflammatory factors and cells, leading to vascular and neuronal damage, ultimately impairing normal brain function [7]. CVECs are essential for maintaining BBB integrity and brain homeostasis. Inflammatory stimuli activate intracellular signaling pathways in endothelial cells, resulting in the production of adhesion molecules, such as VCAM‐1 and P‐selectin, along with proinflammatory mediators [41]. These factors facilitate leukocyte recruitment and BBB leakage [42]. Consequently, targeting CVECs has emerged as a crucial therapeutic approach for NPSLE [7, 43]. Our study demonstrated that SQDHD downregulates the expression of adhesion molecules, reduces leukocyte recruitment, and improves BBB integrity in PIL mice (Figure 2). These beneficial effects may be mediated by the anti‐inflammatory properties of SQDHD, which suppress CVEC activation.

Cytokine dysregulation has been demonstrated to play a pivotal role in the emergence of neuropsychiatric symptoms in both NPSLE animal models and affected patients [44, 45, 46]. In this context, a coordinated increase in pro‐inflammatory cytokines (IL‐1β, TNF‐α, IL‐6) alongside the anti‐inflammatory cytokine IL‐10 was observed in the brains of PIL mice (Figure 3A). This pattern indicates a host‐derived compensatory anti‐inflammatory response [47], where the upregulation of IL‐10 serves as a feedback mechanism aimed at suppressing inflammation and alleviating neuronal injury [48, 49]. However, in NPSLE, this compensatory mechanism becomes overwhelmed and proves inadequate to regulate the ongoing inflammatory cascade [50]. Treatment with SQDHD restores balance to this dysregulated cytokine network by targeting key inflammatory mediators (Figure 3A). Consequently, the concomitant reduction of IL‐10 signifies the normalization of the pathological state that initially triggered the compensatory response, rather than the suppression of a beneficial anti‐inflammatory mechanism [51, 52, 53]. IgG deposition, which exacerbates endothelial injury, microglial activation, and the synthesis of inflammatory mediators [54, 55], has been observed in the hippocampus of PIL mice [56]. This deposition may result from dysfunction of the BBB and blood‐ventricular barrier, triggered by cytokine dysregulation. Treatment with SQDHD reduced IgG deposition in the lateral ventricular wall (Figure 3B,D), likely by modulating cytokine dysregulation to enhance vascular barrier function. Lipofuscin, an autofluorescent pigment that accumulates in neurons subjected to sustained oxidative stress [57]. The pro‐inflammatory microenvironment in NPSLE induces chronic oxidative stress in neurons [58], triggering lipofuscin accumulation and cellular senescence. These processes mutually reinforce oxidative damage and pro‐inflammatory signaling, thereby establishing a self‐sustaining cycle of neuronal injury [59]. The mechanistic connection between lipofuscin‐driven pathology and NPSLE progression is supported by evidence from other chronic inflammatory and neurodegenerative diseases [60, 61]. Additionally, studies have demonstrated that senescent neural cells contribute to NPSLE pathogenesis in lupus‐prone mice exhibiting depressive‐like behaviors [62]. In this study, both SQDHD and a JAKi significantly reduce hippocampal lipofuscin accumulation in PIL mice (Figure 3C,E). This reduction appears to be mediated by SQDHD‐induced downregulation of brain cytokine expression, thereby alleviating the upstream oxidative stress that drives lipofuscin formation.

Using UPLC‐MS/MS and network pharmacology, we identified the active compounds in SQDHD and their potential targets (Figure 4A and Tables S1–, S5). Topological analysis of the overlapping targets revealed the top 15 targets of SQDHD against NPSLE, which are predominantly enriched in the JAK–STAT signaling pathway (Figure 4B–E and Table S6). Dysregulation of the JAK–STAT signaling pathway is associated with memory deficits in NPSLE [63], and inhibition of this pathway is essential for reducing inflammation, maintaining immune tolerance, and reinforcing barrier functions in SLE [64]. For instance, C‐28 methyl ester of 2‐cyano‐3, 12‐dioxoolean‐1, 9‐dien‐28‐oic acid (CDDO‐Me, a selective JAK1/STAT3 inhibitor), has demonstrated efficacy in alleviating LN in both preventive contexts (B6‐*Sle1.Sle3* mice and MRL/lpr mice) and established setting (NZM2410 mice) [65]. TCMs, characterized by their multi‐component, multi‐target and multi‐pathway properties, offer significant advantages and promising prospects for treating complex diseases such as SLE [14, 66, 67]. Our investigation into SQDHD illustrates these advantages. In contrast to single‐target inhibitors such as JAKi, SQDHD concurrently engages multiple targets within the JAK–STAT network and associated inflammatory cascades, potentially enabling more balanced and sustained immunomodulation (Figure 4C–E). Additionally, our KEGG pathway analysis indicated that the top 15 targets of SQDHD against NPSLE are also significantly enriched in other critical inflammatory pathways, including the IL‐17 and TNF signaling pathways (Figure 4E). These findings suggest that SQDHD's broad anti‐inflammatory effects are achieved through a synergistic, multi‐target mechanism that extends beyond JAK–STAT inhibition. Therefore, this TCM‐based, systems‐level therapeutic approach using SQDHD represents a promising strategy for addressing NPSLE.

Upadacitinib, a selective JAK1i, has demonstrated efficacy and an acceptable safety profile in SLE and other immune‐mediated conditions [68, 69]. Molecular docking analysis revealed that the primary active components of SQDHD, identified through the CT network (Figure 5A and Table S7), exhibit stronger binding affinities to JAK1 and other hub targets enriched in the JAK–STAT pathway than Upadacitinib (Figure 5B,C). These findings suggest that SQDHD may exert therapeutic effects comparable to those of Upadacitinib in NPSLE by targeting the JAK1‐STAT3 signaling pathway. To test this possibility, we employed CETSAs and DARTS assays, which confirmed that all five active compounds exhibit direct binding to JAK1, thereby inhibiting the JAK1‐STAT3 signaling pathway (Figure 5D,E). Furthermore, Alisol B Acetate and Hederagenin, the primary active components of SQDHD, have been shown to modulate phosphorylation in various signaling pathways, including P38 mitogen‐activated protein kinase (MAPK), extracellular‐signal‐regulated kinase (ERK), and adenosine monophosphate‐activated protein kinase (AMPK)‐mammalian target of rapamycin (mTOR) [70, 71], suggesting their potential role in regulating the JAK1‐STAT3 pathway. Consistent with this, we demonstrated that both Alisol B Acetate and Hederagenin inhibit the JAK1‐STAT3 pathway and downregulate its downstream effector molecules in CVECs induced by lupus serum (Figure 8). This investigation was based on the extensively documented involvement of JAK–STAT signaling in endothelial activation. First, JAK–STAT signaling proteins are specifically localized within endothelial caveolae, which are regulated in an agonist‐dependent manner [72]. Second, inhibiting this pathway decreases TNF‐α‐induced endothelial cell activation, resulting in markedly reduced secretion of P‐selectin, von Willebrand factor, and IL‐6 [10]. Third, *Astragalus extract*, a key active component of SQDHD, has been shown to significantly attenuate inflammatory responses in human umbilical vein endothelial cells through specific inhibition of JAK2 and STAT3 phosphorylation [73]. These established roles of JAK–STAT signaling led us to hypothesize that SQDHD may act through this pathway**—**a possibility subsequently confirmed by our experimental data. Our findings indicated that SQDHD inhibits the JAK1‐STAT3 pathway in both PIL mice and lupus serum‐induced CVECs (Figures 6 and 9). Importantly, the rescue experiment in CVECs provided conclusive evidence that the anti‐neuroinflammatory efficacy of SQDHD relies on the specific multi‐target inhibition of this pathway (Figure 7). These findings strongly indicate that SQDHD's therapeutic effectiveness in NPSLE primarily stems from blocking this critical signaling pathway.

This study presents evidence that supports the therapeutic potential of SQDHD for NPSLE; however, several limitations must be acknowledged. First, the translational relevance of our preclinical findings to humans necessitates further validation, particularly due to the known species differences in the metabolism of TCMs. Second, the lack of long‐term safety data for SQDHD warrants future investigation before clinical advancement. Finally, given the potential off‐target effects and complex interactions in multi‐component herbal formulas [15, 74], the inhibition of JAK1‐STAT3 was identified as a core therapeutic mechanism of SQDHD against NPSLE. This multi‐target profile, rather than signifying nonspecific off‐target effects, typically reflects a deliberate synergistic strategy characteristic of herbal polypharmacology [75, 76]. For instance, Zhenwu Decoction has been demonstrated to operate via synergistic regulatory mechanisms, evidenced by its 59 components targeting 37 targets to synergistically improve thyroid function and histopathology without the off‐target side effects of standard therapies [77]. Similarly, the 17‐herb Jinlida granule ameliorates glomerulosclerosis through a dual synergistic mechanism, which involves reshaping gut microbiota to generate the protective metabolite pyridoxamine and targeting fibrotic pathways with multi‐components, effectively preventing off‐target effects [78]. Thus, the “multi‐component, multi‐target” nature of SQDHD is more consistent with a deliberate multi‐target synergy than with nonspecific off‐target effects. Future systems‐level pharmacological investigations will be essential for comprehensively delineating the complete interaction network of SQDHD.

## 5 Conclusion

In summary, we provide the first evidence that SQDHD may exert an anti‐inflammatory effect in activated CVECs by inhibiting the JAK1‐STAT3 signaling pathway in NPSLE. Our results underscore the importance of SQDHD in mitigating behavioral deficits and reversing neuroinflammation in NPSLE, suggesting its potential as a therapeutic strategy for NPSLE.

## Author Contributions

Y.Y. supervised the project. J.C. designed the project, wrote the manuscript and performed the statistical analysis and revised the manuscript. C.C., X.W., H.Y., Jie C., J.F., and C.J. were involved in laboratory works and experimental design of the work. Jie C., Jingyu C., X.W., Y.L., and F.X. were involved in data collection and lab assessments, and study designing. All authors read and approved the final manuscript.

## Funding

This work was supported by the Collaborative Science and Technology Project of Liaoning Province, 2024‐BSLH‐308. The department of Education of Liaoning Province, LJKMZ20221145.

## Ethics Statement

The experimental scheme was approved by the Animal Care and Use Committee of Shengjing Hospital affiliated China Medical University (No. 2024PS589K).

## Conflicts of Interest

The authors declare no conflicts of interest.

## Supporting information


**Table S1:** A total of 61 components identified by UPLC‐MS/MS and relevant literature.

**Table S2:** The chemical fingerprint of 61 components in SQDHD.

**Table S3:** Collection of NPSLE associated targets.

**Table S4:** Collection of 61 active compounds associated targets.

**Table S5:** Collection of overlapping target genes between SQDHD and NPSLE.

**Table S6:** GO and KEGG analyses of the top 15 targets.

**Table S7:** Degree values between the active compounds and the top 15 targets.

## Data Availability

The data that support the findings of this study are available from the corresponding author upon reasonable request.
