# Supplementary material for: ShenQi DiHuang Decoction (SQDHD) Ameliorates Neuroinflammation and Neuropsychiatric Manifestations in Pristane Induced Lupus Mice via Blocking JAK1‐STAT3 Pathway
Source: CNS Neurosci Ther. 2026 Mar 7;32(3):e70814. doi: 10.1002/cns.70814 (PMC12967629; doi:10.1002/cns.70814)
Supplement: Supplementary file 1 — Table S1: A total of 61 components identified by UPLC‐MS/MS and relevant literature. [file CNS-32-e70814-s001.docx]

Table S1. A total of 61 components identified by UPLC-MS/MS and relevant literature.

| **No** | **Ionization model** | **Compounds** | **RT** | **Class I** | **Class II** | **CAS** | **Level** |
| --- | --- | --- | --- | --- | --- | --- | --- |
| 1 | [M-H]- | Melibiose | 0.7 | Others | Saccharides | 585-99-9 | 3 |
| 2 | [M-H]- | D-Fructose* | 0.76 | Others | Saccharides | 57-48-7 | 1 |
| 3 | [M-H]- | Raffinose* | 0.76 | Others | Saccharides | 512-69-6 | 1 |
| 4 | [M-H]- | D-Maltose* | 0.8 | Others | Saccharides | 69-79-4 | 1 |
| 5 | [M-H]- | Manninotriose | 0.8 | Others | Saccharides | 13382-86-0 | 1 |
| 6 | [M-H]- | D-Galactose* | 0.9 | Others | Saccharides | 59-23-4 | 1 |
| 7 | [M-H]- | Uridine | 1.2 | Nucleotides and derivatives | Nucleotides and derivatives | 58-96-8 | 1 |
| 8 | [M-H]- | 3,4-Dihydroxybenzoic acid (Protocatechuic acid)* | 2.5 | Phenolic acids | Phenolic acids | 99-50-3 | 1 |
| 9 | [M-H]- | L-Tryptophan | 2.5 | Amino acids and derivatives | Amino acids and derivatives | 73-22-3 | 1 |
| 10 | [M-H]- | Salidroside | 2.6 | Others | Alcohol compounds | 10338-51-9 | 3 |
| 11 | [M-H]- | Hydroquinone | 2.7 | Others | Others | 123-31-9 | 3 |
| 12 | [M-H]- | Procyanidin B1 | 2.7 | Tannins | Proanthocyanidins | 20315-25-7 | 2 |
| 13 | [M-H]- | 4-Hydroxybenzoic acid | 3 | Phenolic acids | Phenolic acids | 99-96-7 | 1 |
| 14 | [M-H]- | Catechin | 3 | Flavonoids | Flavanols | 154-23-4 | 1 |
| 15 | [M-H]- | Homovanillic acid; 4-Hydroxy-3-methoxyphenylacetic acid | 3.1 | Phenolic acids | Phenolic acids | 306-08-1 | 3 |
| 16 | [M-H]- | Methyl gallate* | 3.2 | Phenolic acids | Phenolic acids | 99-24-1 | 1 |
| 17 | [M-H]- | Vanillic acid | 3.3 | Phenolic acids | Phenolic acids | 121-34-6 | 1 |
| 18 | [M-H]- | Syringic acid | 3.4 | Phenolic acids | Phenolic acids | 530-57-4 | 1 |
| 19 | [M-H]- | Caffeic acid | 3.4 | Phenolic acids | Phenolic acids | 331-39-5 | 1 |
| 20 | [M-H]- | Quercetin-3-O-rutinoside (Rutin) | 3.7 | Flavonoids | Flavonols | 153-18-4 | 3 |
| 21 | [M-H]- | Ellagic acid | 3.8 | Tannins | Tannin | 476-66-4 | 1 |
| 22 | [M-H]- | Methyl 4-hydroxybenzoate | 3.9 | Phenolic acids | Phenolic acids | 99-76-3 | 1 |
| 23 | [M-H]- | Gallic Acid Ethyl Ester; Ethyl gallate | 3.9 | Phenolic acids | Phenolic acids | 831-61-8 | 1 |
| 24 | [M-H]- | Genistein-7-O-Glucoside (Genistin) | 3.9 | Flavonoids | Isoflavones | 529-59-9 | 2 |
| 25 | [M-H]- | Sinapic acid | 4 | Phenolic acids | Phenolic acids | 530-59-6 | 1 |
| 26 | [M-H]- | Kaempferol-3-O-galactoside (Trifolin)* | 4 | Flavonoids | Flavonols | 23627-87-4 | 2 |
| 27 | [M-H]- | Vanillin; 4-Hydroxy-3-Methoxybenzaldehyde | 4 | Others | Aldehyde compounds | 121-33-5 | 1 |
| 28 | [M-H]- | 3-Hydroxybenzoic acid | 4.2 | Phenolic acids | Phenolic acids | - | 1 |
| 29 | [M-H]- | Azelaic acid | 4.4 | Lipids | Free fatty acids | 123-99-9 | 1 |
| 30 | [M-H]- | Glycitein | 5.2 | Flavonoids | Isoflavones | 40957-83-3 | 1 |
| 31 | [M-H]- | Hesperetin | 5.6 | Flavonoids | Flavanones | 520-33-2 | 3 |
| 32 | [M-H]- | 5,4'-Dihydroxy-3,7-dimethoxyflavone(Kumatakenin)* | 7.2 | Flavonoids | Flavonols | 3301-49-3 | 2 |
| 33 | [M-H]- | 3,23-Dihydroxyolean-12-en-28-oic acid (Hederagenin)* | 8.97 | Terpenoids | Triterpene | 465-99-6 | 3 |
| 34 | [M+H]+ | 8-Debenzoylpaeoniflorin | 1.3 | Terpenoids | Monoterpenoids | 23532-11-8 | 2 |
| 35 | [M+H]+ | Benzamide | 2.3 | Alkaloids | Alkaloids | 55-21-0 | 3 |
| 36 | [M+H]+ | Riboflavin (Vitamin B2) | 3.2 | Others | Vitamin | 83-88-5 | 1 |
| 37 | [M+H]+ | Calycosin-7-O-glucoside | 3.9 | Flavonoids | Isoflavones | 20633-67-4 | 1 |
| 38 | [M+H]+ | Kaempferol-3-O-neohesperidoside* | 3.9 | Flavonoids | Flavonols | 32602-81-6 | 2 |
| 39 | [M+H]+ | Quercetin-3-O-glucoside (Isoquercitrin)* | 4 | Flavonoids | Flavonols | 482-35-9 | 2 |
| 40 | [M+H]+ | Apigenin-7-O-neohesperidoside (Rhoifolin)* | 4.1 | Flavonoids | Flavones | 17306-46-6 | 2 |
| 41 | [M+H]+ | Cornuside | 4.1 | Terpenoids | Monoterpenoids | 131189-57-6 | 3 |
| 42 | [M+H]+ | Kaempferol-3-O-glucoside (Astragalin)* | 4.2 | Flavonoids | Flavonols | 480-10-4 | 3 |
| 43 | [M+H]+ | Quercetin-3-O-rhamnoside(Quercitrin) | 4.3 | Flavonoids | Flavonols | 522-12-3 | 3 |
| 44 | [M+H]+ | Benzoic acid | 4.5 | Phenolic acids | Phenolic acids | 65-85-0 | 3 |
| 45 | [M+H]+ | Formononetin-7-O-glucoside (Ononin) | 4.7 | Flavonoids | Isoflavones | 486-62-4 | 1 |
| 46 | [M+H]+ | Isoluteolin (Orobol)(5,7,3',4'-tetrahydroxyisoflavone) | 4.8 | Flavonoids | Isoflavones | 480-23-9 | 2 |
| 47 | [M+H]+ | Quercetin | 5.1 | Flavonoids | Flavonols | 117-39-5 | 3 |
| 48 | [M+H]+ | Calycosin | 5.2 | Flavonoids | Isoflavones | 20575-57-9 | 1 |
| 49 | [M+H]+ | Biochanin A-7-O-glucoside (Sissotrin)* | 5.2 | Flavonoids | Isoflavones | 5928-26-7 | 1 |
| 50 | [M+H]+ | Isoformononetin | 6.2 | Flavonoids | Isoflavones | 486-63-5 | 1 |
| 51 | [M+H]+ | Formononetin (7-Hydroxy-4'-methoxyisoflavone) | 6.3 | Flavonoids | Isoflavones | 485-72-3 | 1 |
| 52 | [M+H]+ | Wogonin (5,7-Dihydroxy-8-Methoxyflavone) | 6.9 | Flavonoids | Flavones | 632-85-9 | 3 |
| 53 | [M+H]+ | Pectolinarigenin | 7 | Flavonoids | Flavones | 520-12-7 | 2 |
| 54 | [M+H]+ | Nobiletin (5,6,7,8,3',4'-Hexamethoxyflavone)* | 7.1 | Flavonoids | Flavones | 478-01-3 | 1 |
| 55 | [M+H]+ | 16-oxo-11-anhydroalisol A | 7.3 | Terpenoids | Triterpene | - | 1 |
| 56 | [M+H]+ | alisol C,23-acetate* | 7.9 | Terpenoids | Triterpene | - | 2 |
| 57 | [M+H]+ | Mudanpinoic acid A | 8.8 | Terpenoids | Triterpene | 203511-36-8 | 3 |
| 58 | [M+H]+ | 3-(Acetyloxy)-16-hydroxy-24-methylenelanost-8-en-21-oic acid (Pachymic acid) | 9.5 | Terpenoids | Triterpene | 29070-92-6 | 3 |
| 59 | [M+H]+ | 3-O-Acetyl-16-hydroxytrametenolic acid | 10.6 | Terpenoids | Triterpene | 168293-13-8 | 2 |
| 60 | [M+H]+ | Oleic acid | 10.7 | Lipids | Free fatty acids | 112-80-1 | 3 |
| 61 | [M+H]+ | Alisol B Acetate | 10.7 | Terpenoids | Triterpene | 26575-95-1 | 1 |

*calibrated with the standards.
