# Supplementary material for: ShenQi DiHuang Decoction (SQDHD) Ameliorates Neuroinflammation and Neuropsychiatric Manifestations in Pristane Induced Lupus Mice via Blocking JAK1‐STAT3 Pathway
Source: CNS Neurosci Ther. 2026 Mar 7;32(3):e70814. doi: 10.1002/cns.70814 (PMC12967629; doi:10.1002/cns.70814)
Supplement: Supplementary file 2 — Table S2: The chemical fingerprint of 61 components in SQDHD. [file CNS-32-e70814-s002.docx]

Table S2. The chemical fingerprint of 61 components in SQDHD,

| **No** | **Compounds** | **chemical fingerprint (MS)** |
| --- | --- | --- |
| 1 | Melibiose | 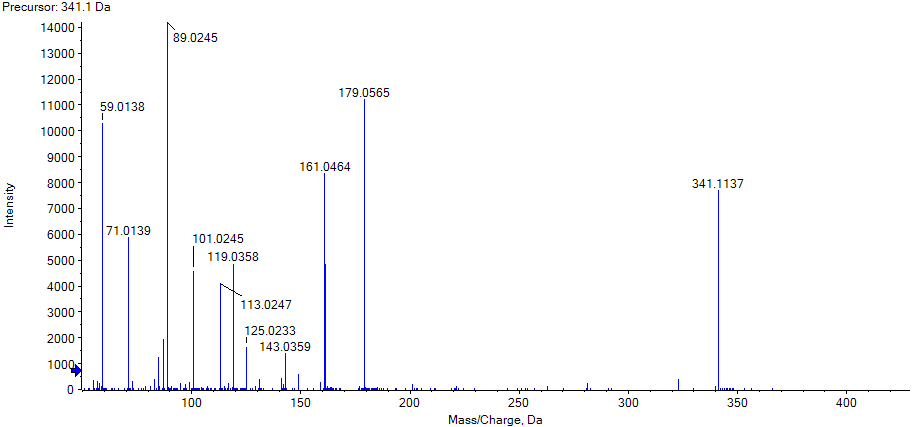 |
| 2 | D-Fructose* | 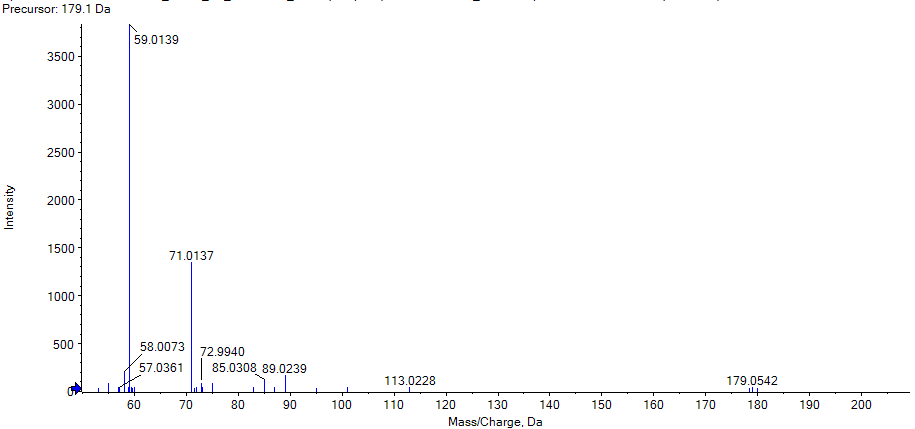 |
| 3 | Raffinose* | 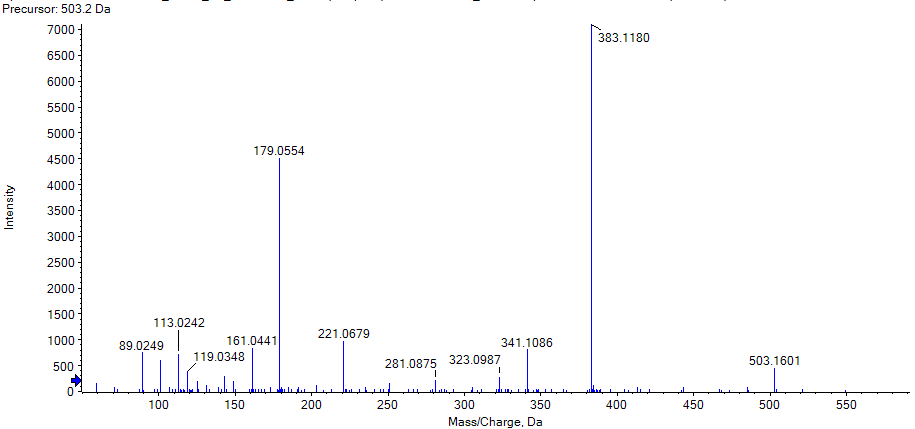 |
| 4 | D-Maltose* | 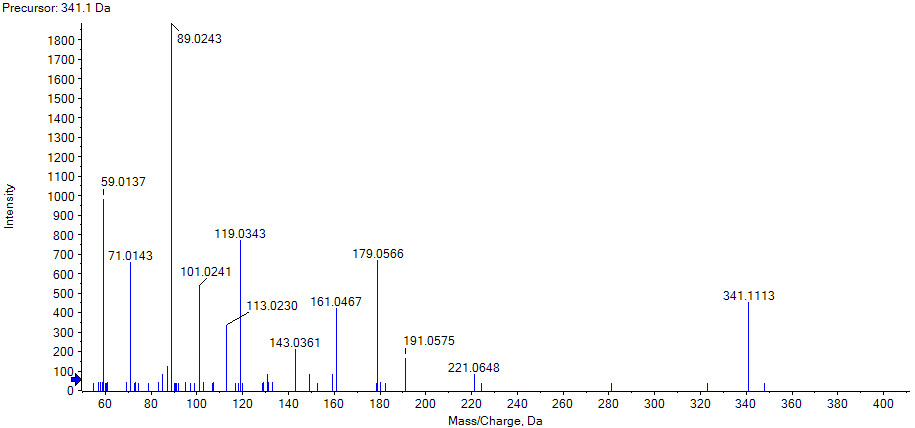 |
| 5 | Manninotriose | 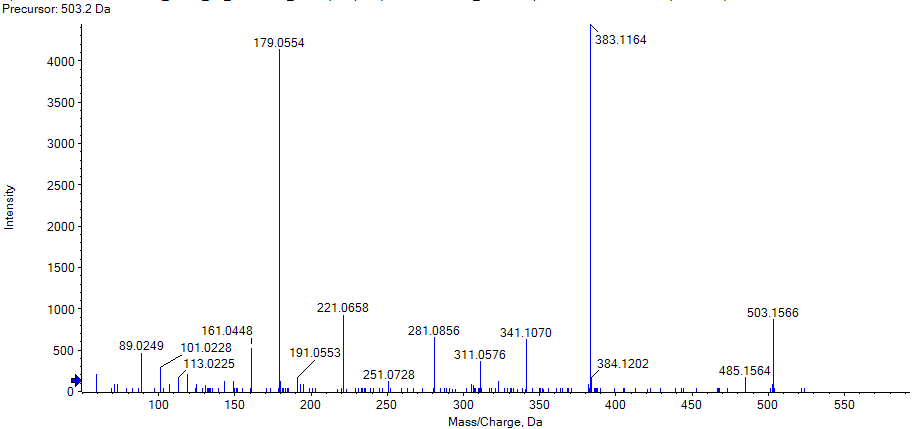 |
| 6 | D-Galactose* | 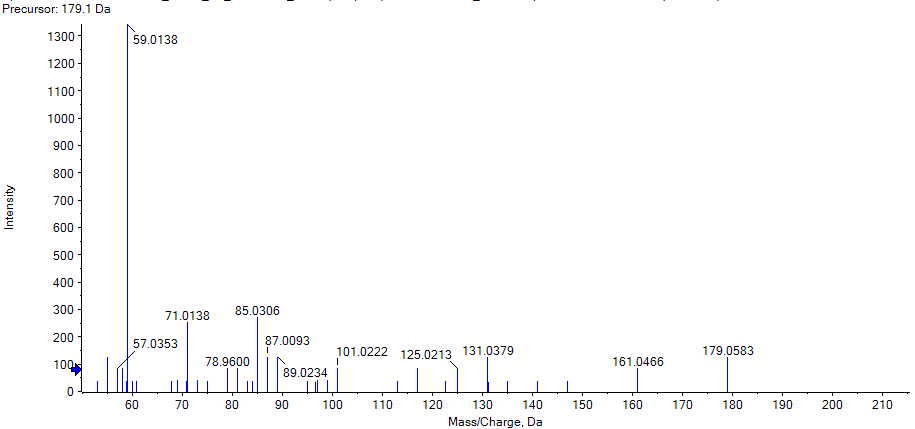 |
| 7 | Uridine | 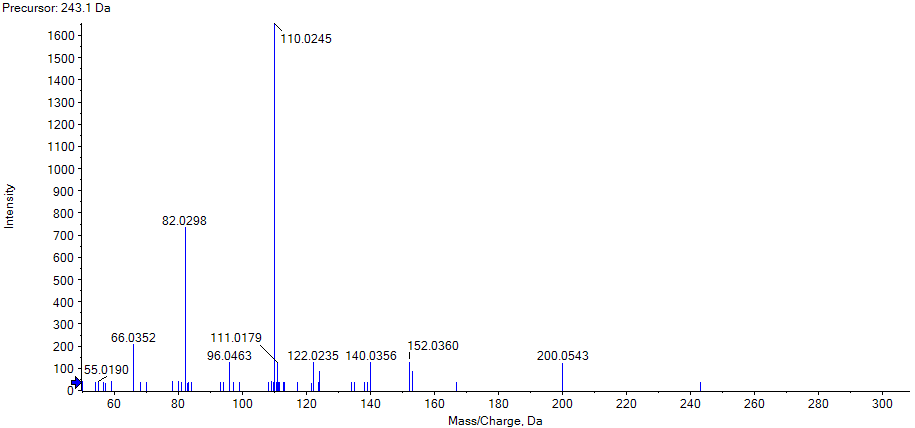 |
| 8 | 3,4-Dihydroxybenzoic acid (Protocatechuic acid)* | 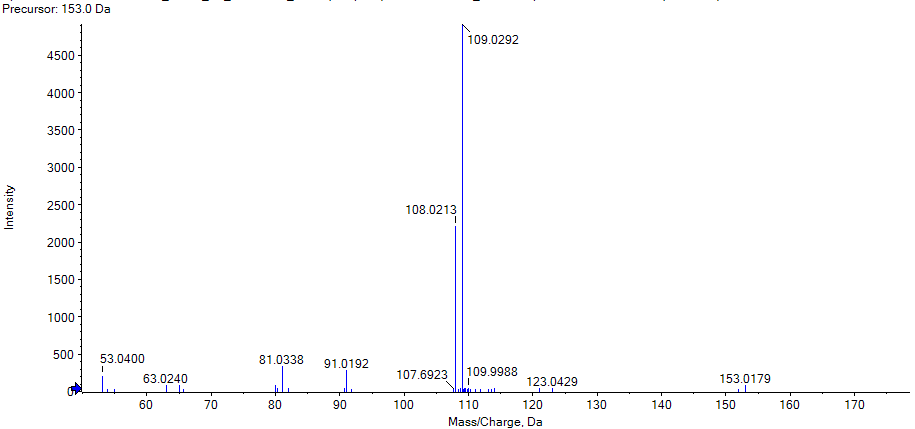 |
| 9 | L-Tryptophan | 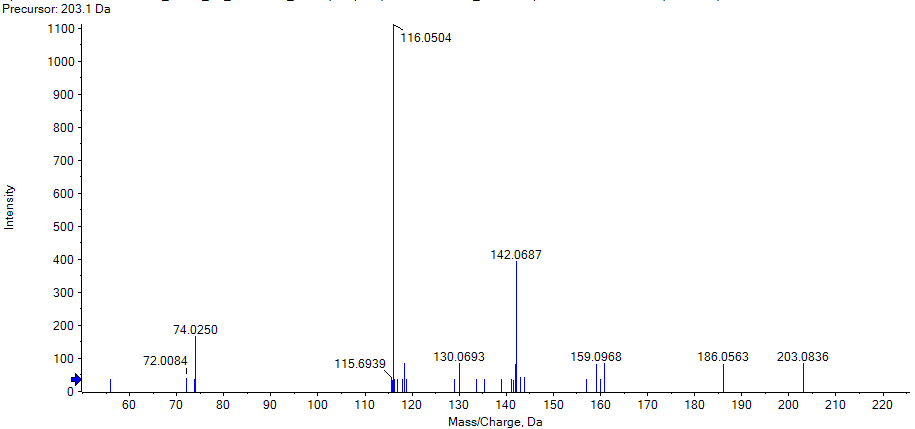 |
| 10 | Salidroside | 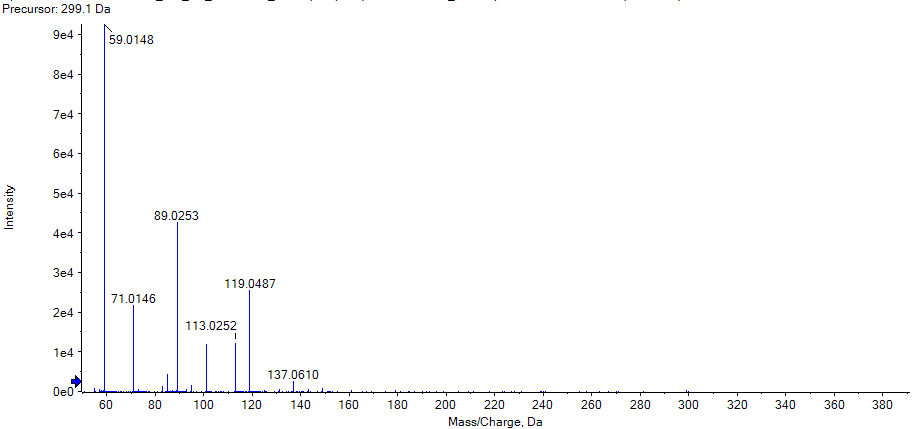 |
| 11 | Hydroquinone | 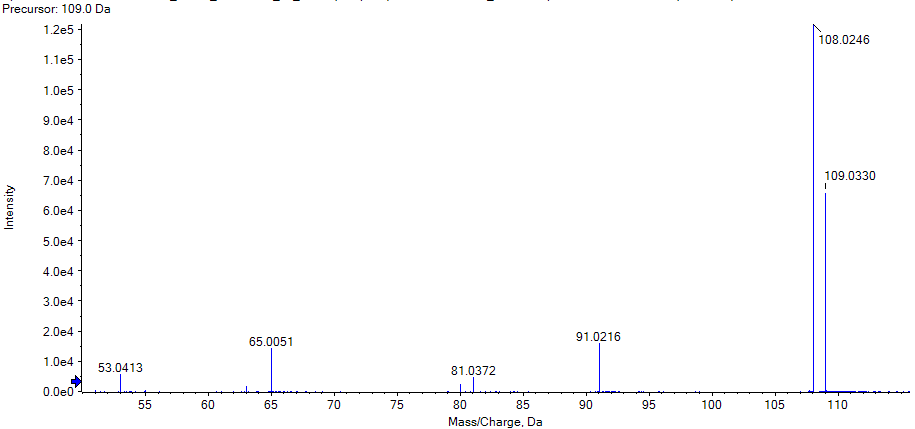 |
| 12 | Procyanidin B1 | 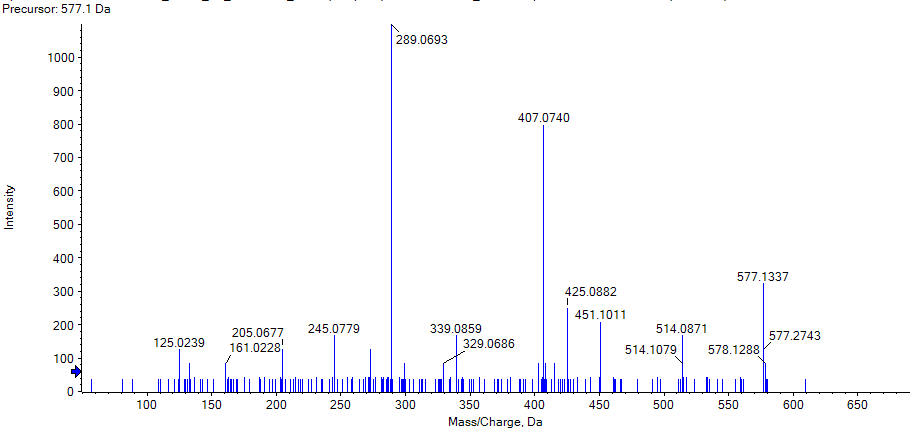 |
| 13 | 4-Hydroxybenzoic acid | 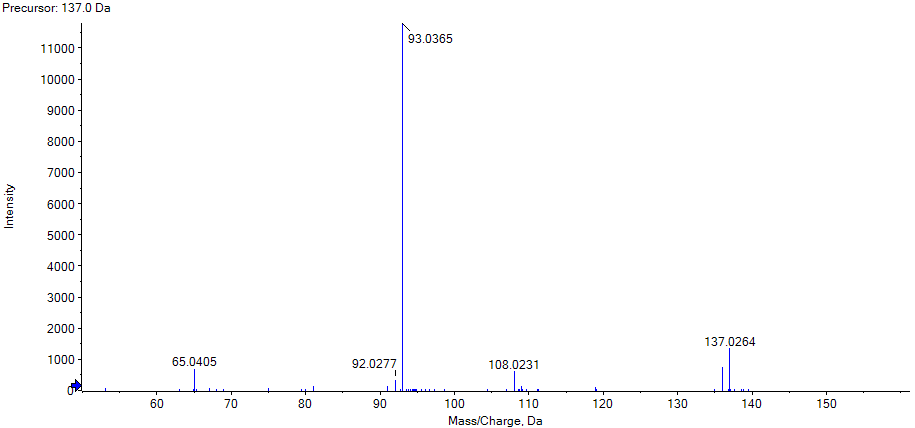 |
| 14 | Catechin | 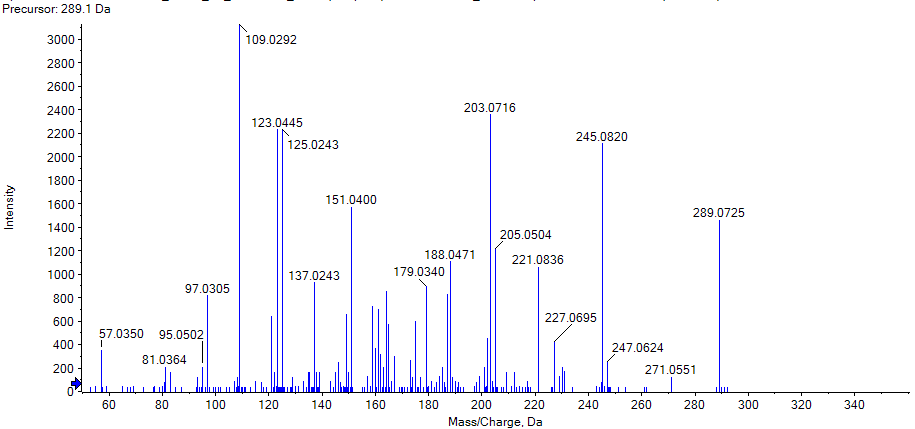 |
| 15 | Homovanillic acid; 4-Hydroxy-3-methoxyphenylacetic acid | 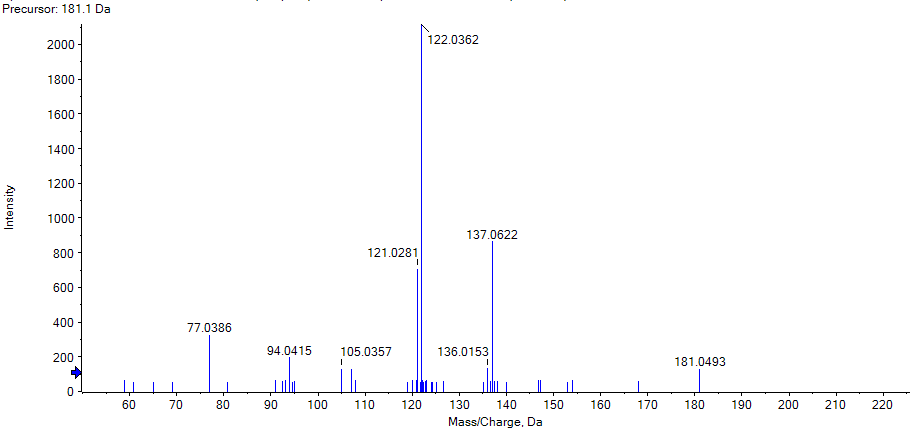 |
| 16 | Methyl gallate* | 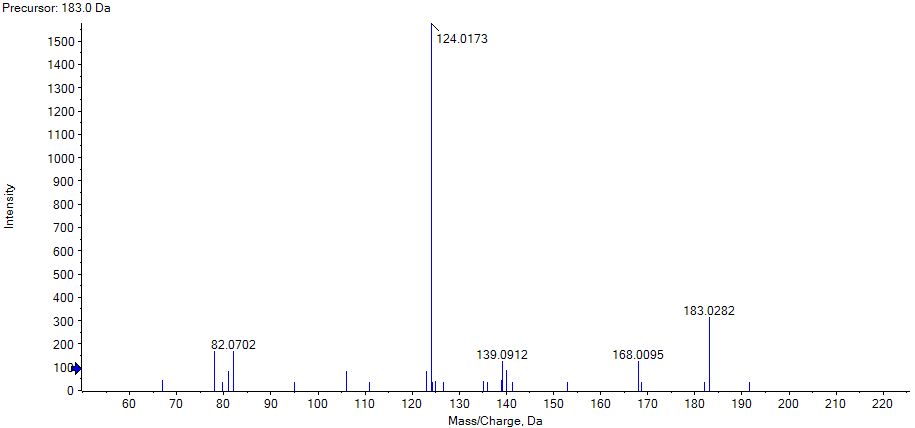 |
| 17 | Vanillic acid | 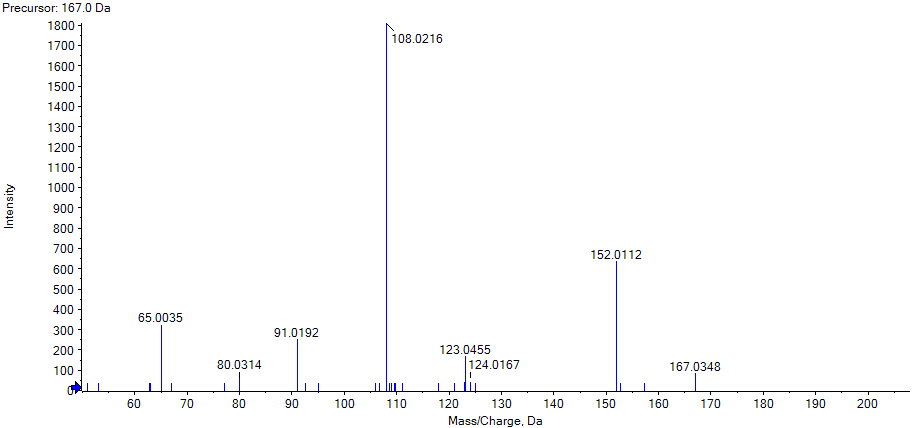 |
| 18 | Syringic acid | 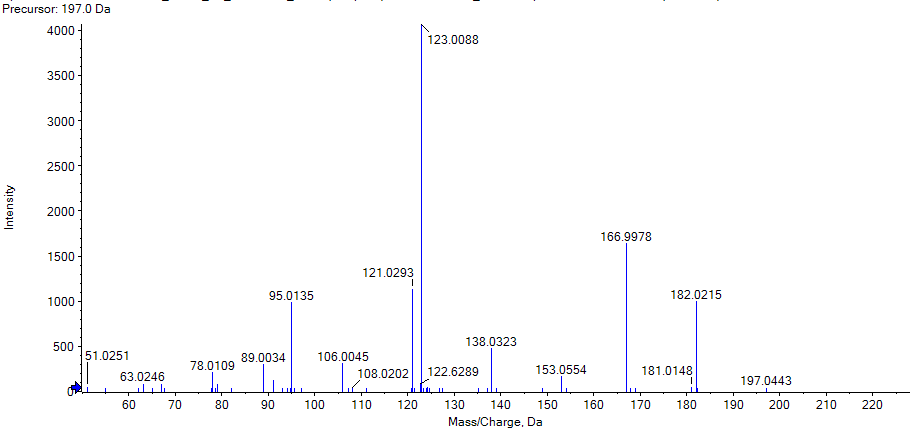 |
| 19 | Caffeic acid | 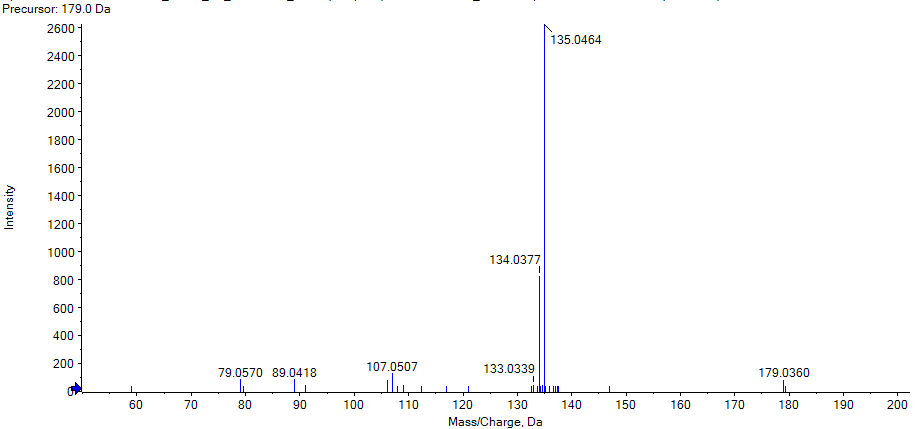 |
| 20 | Quercetin-3-O-rutinoside (Rutin) | 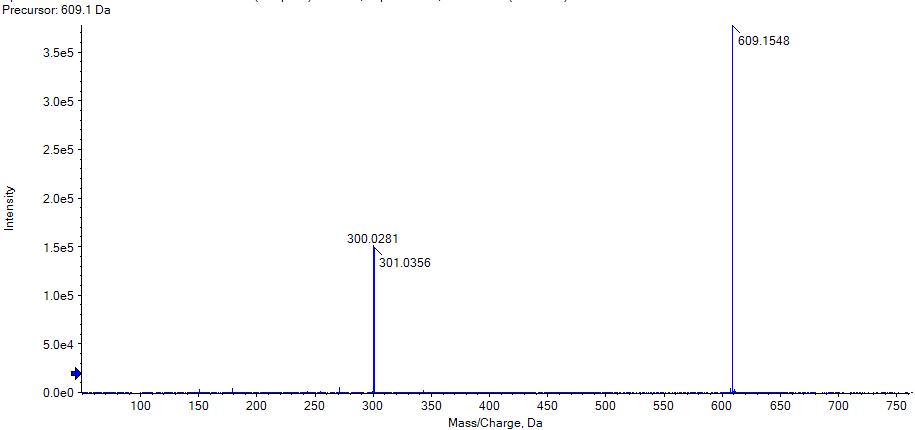 |
| 21 | Ellagic acid | 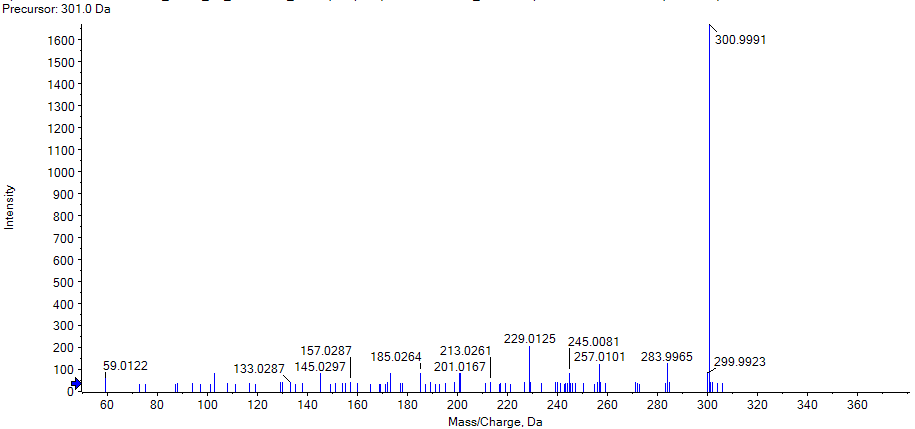 |
| 22 | Methyl 4-hydroxybenzoate | 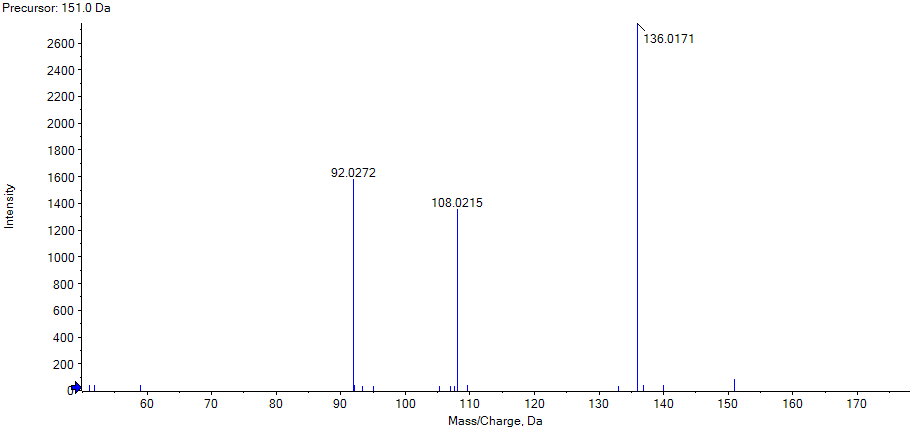 |
| 23 | Gallic Acid Ethyl Ester; Ethyl gallate | 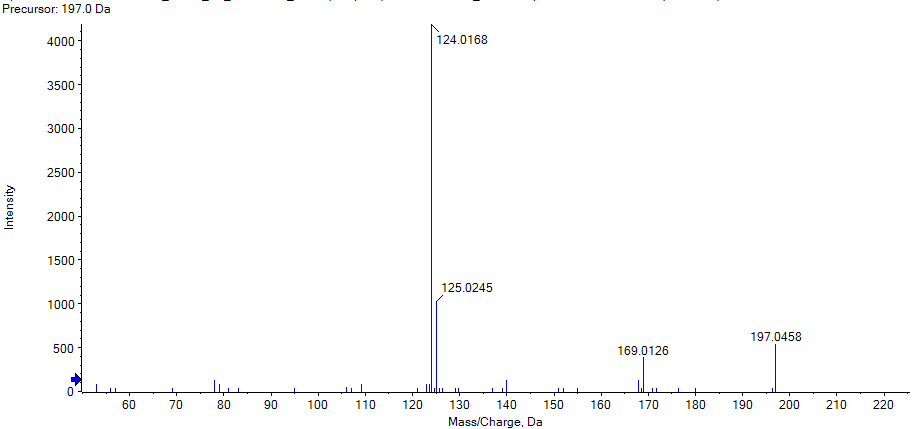 |
| 24 | Genistein-7-O-Glucoside (Genistin) | 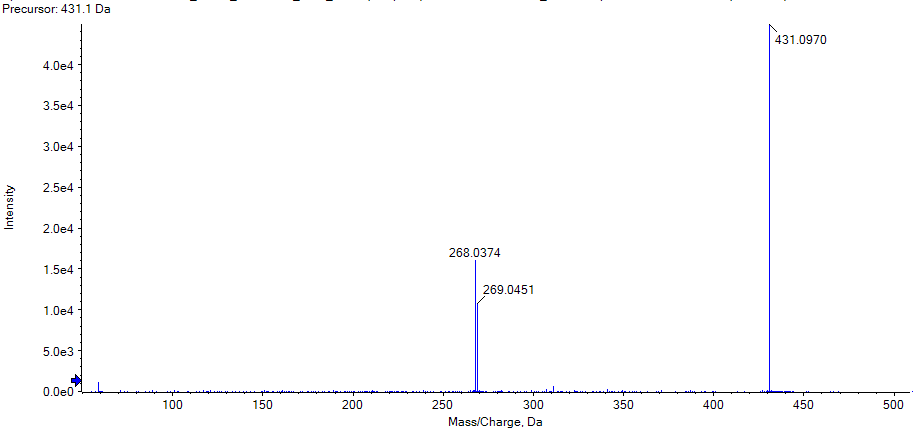 |
| 25 | Sinapic acid | 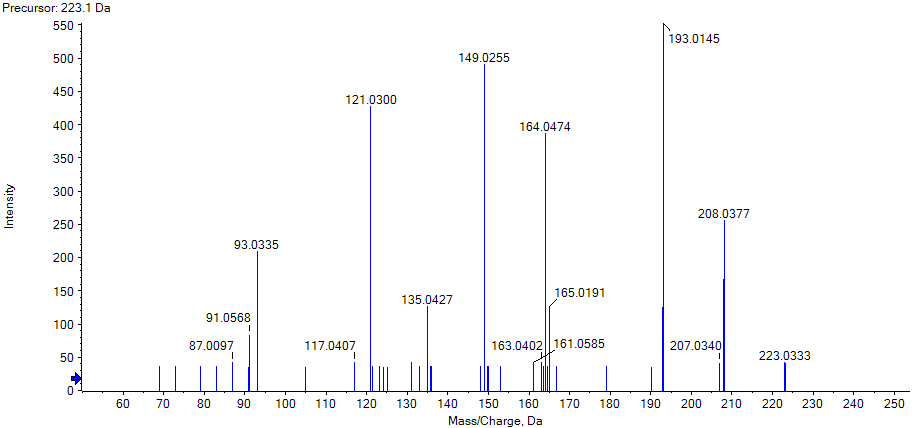 |
| 26 | Kaempferol-3-O-galactoside (Trifolin)* | 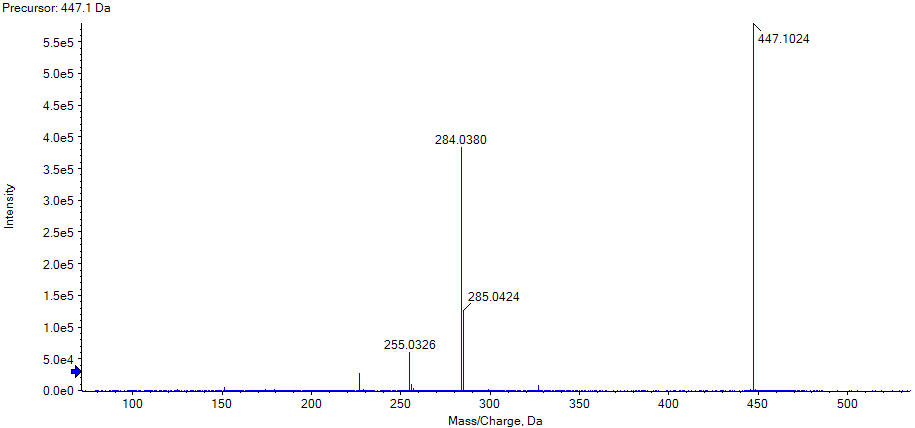 |
| 27 | Vanillin; 4-Hydroxy-3-Methoxybenzaldehyde | 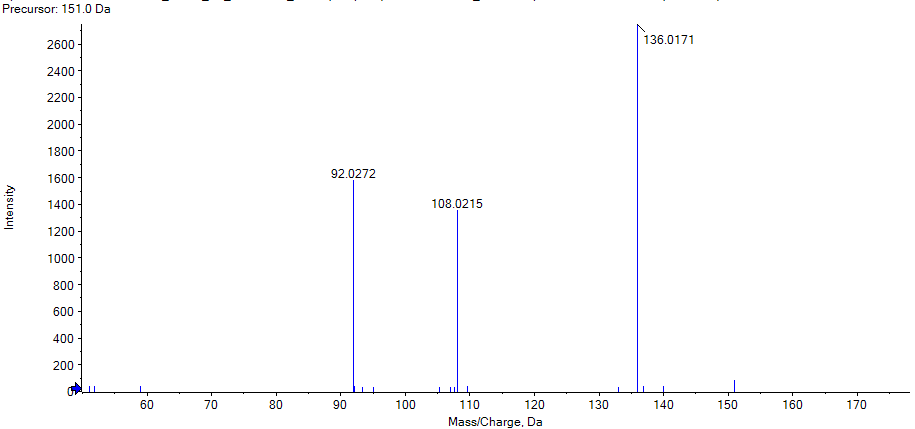 |
| 28 | 3-Hydroxybenzoic acid | 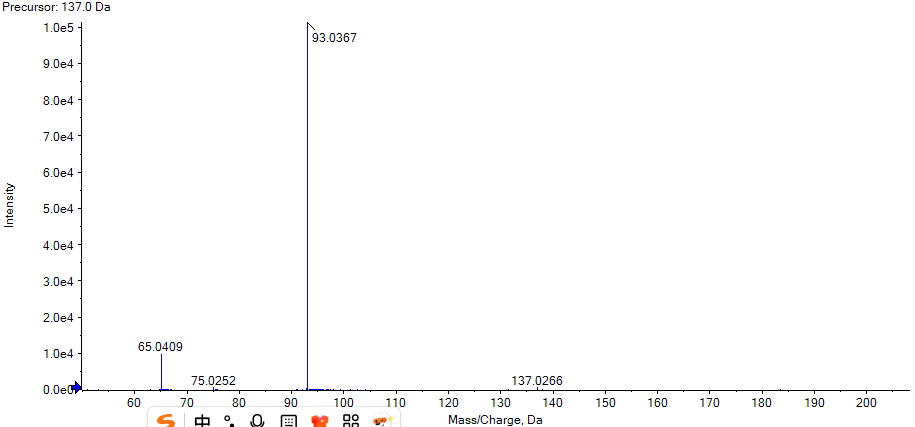 |
| 29 | Azelaic acid | 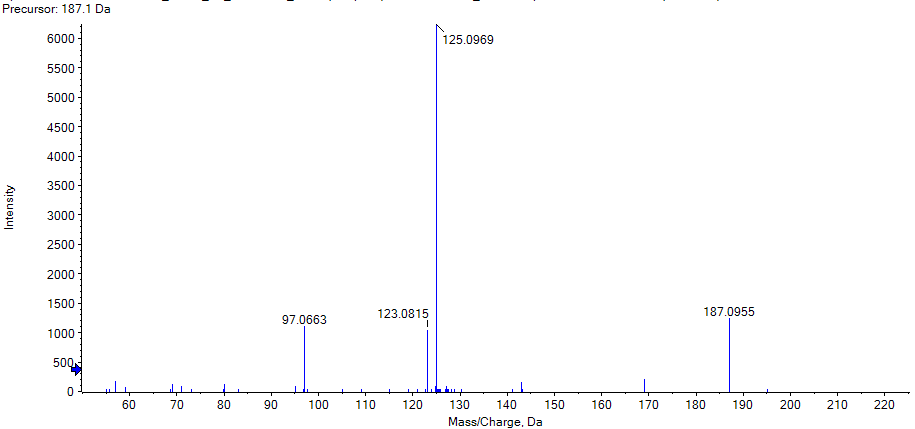 |
| 30 | Glycitein | 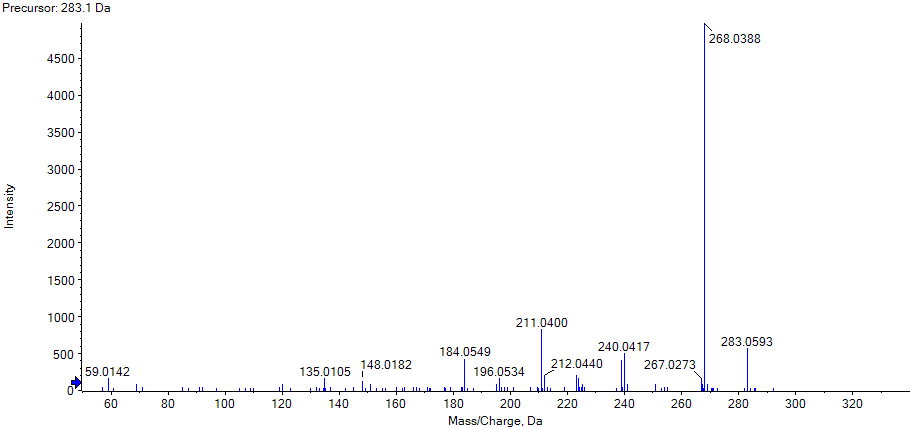 |
| 31 | Hesperetin | 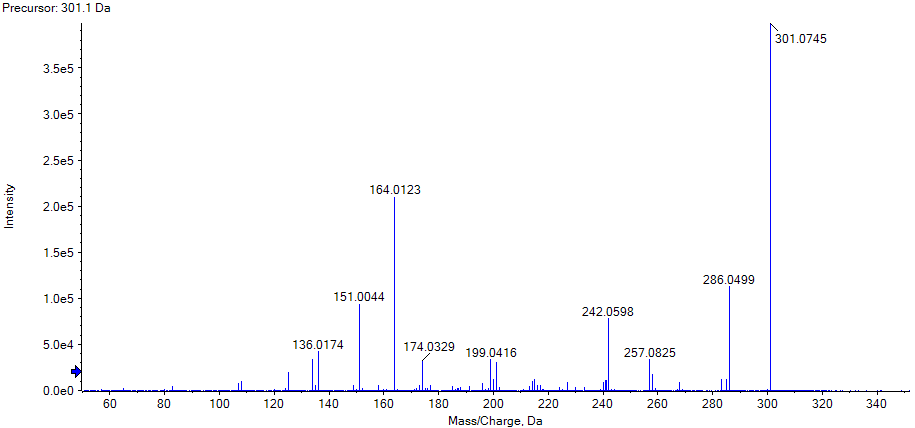 |
| 32 | 5,4'-Dihydroxy-3,7-dimethoxyflavone(Kumatakenin)* | 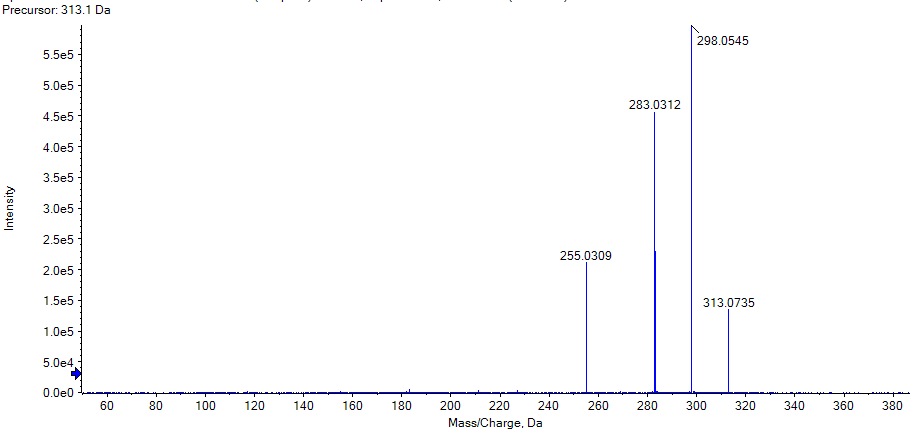 |
| 33 | 3,23-Dihydroxyolean-12-en-28-oic acid (Hederagenin)* | 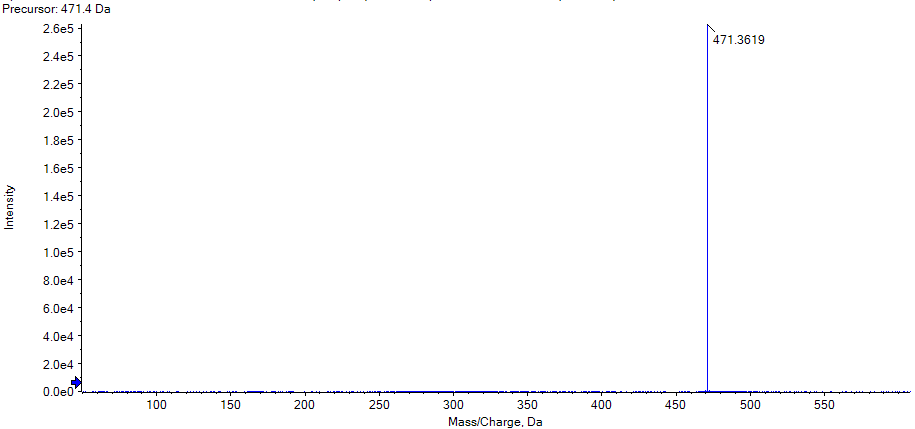 |
| 34 | 8-Debenzoylpaeoniflorin | 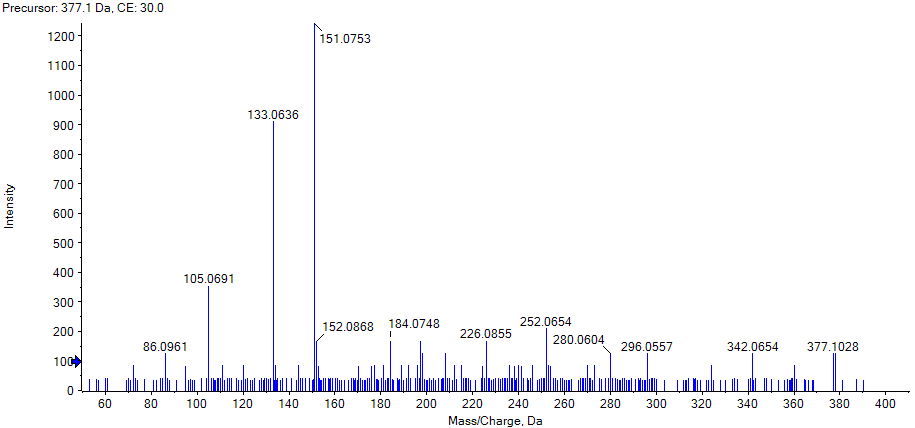 |
| 35 | Benzamide | 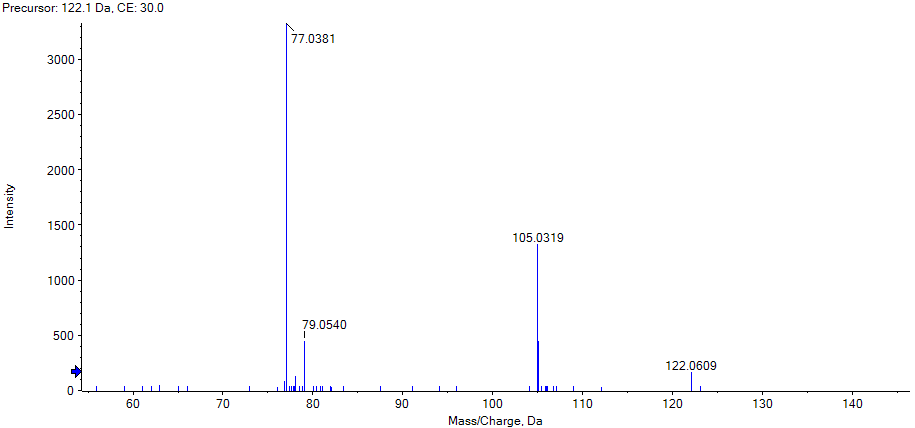 |
| 36 | Riboflavin (Vitamin B2) | 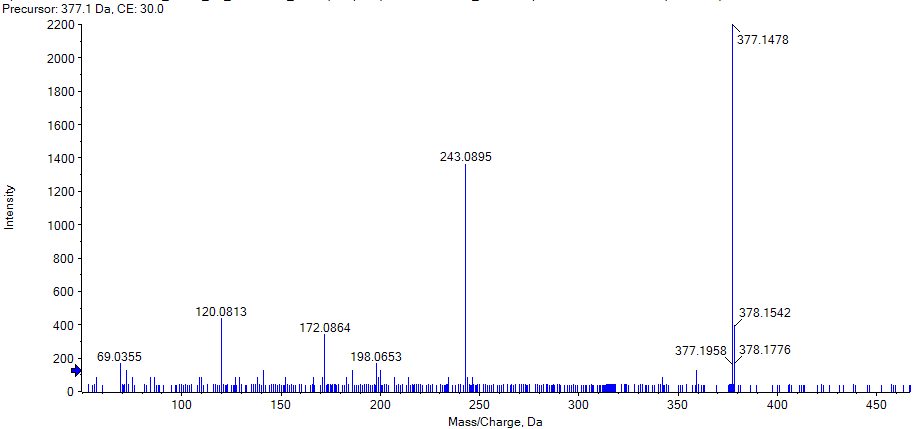 |
| 37 | Calycosin-7-O-glucoside | 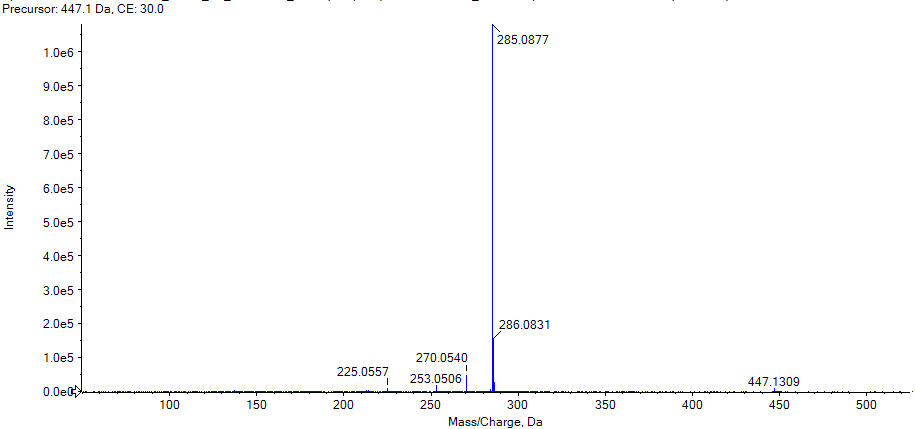 |
| 38 | Kaempferol-3-O-neohesperidoside* | 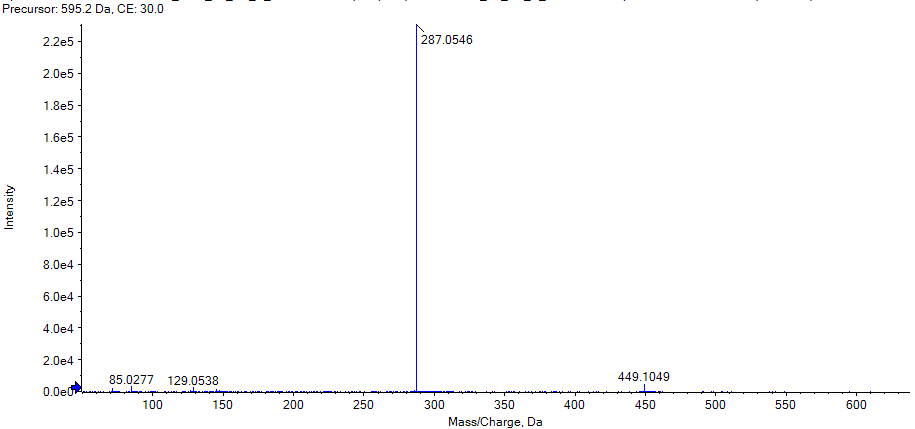 |
| 39 | Quercetin-3-O-glucoside (Isoquercitrin)* | 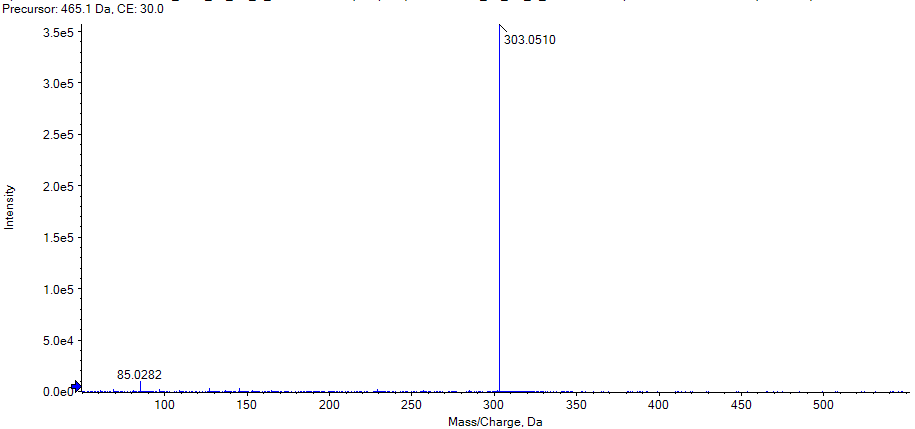 |
| 40 | Apigenin-7-O-neohesperidoside (Rhoifolin)* | 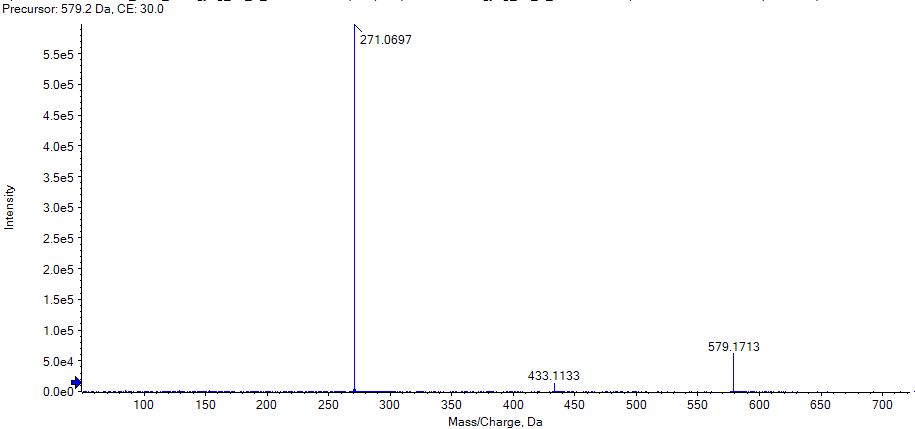 |
| 41 | Cornuside | 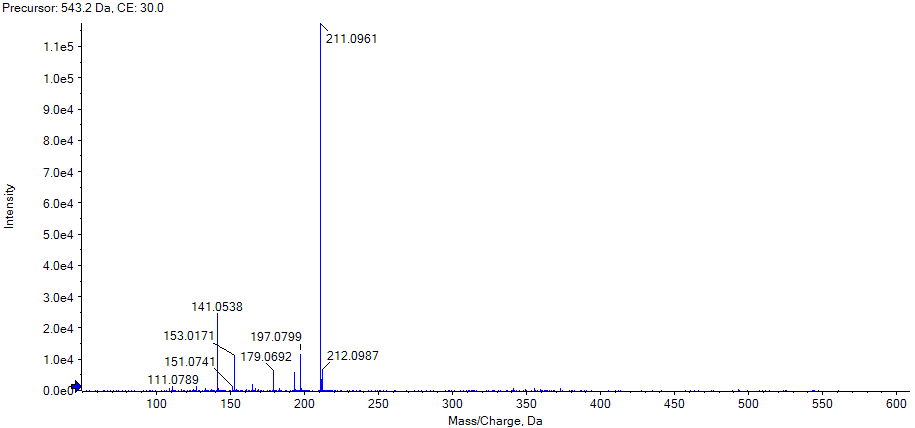 |
| 42 | Kaempferol-3-O-glucoside (Astragalin)* | 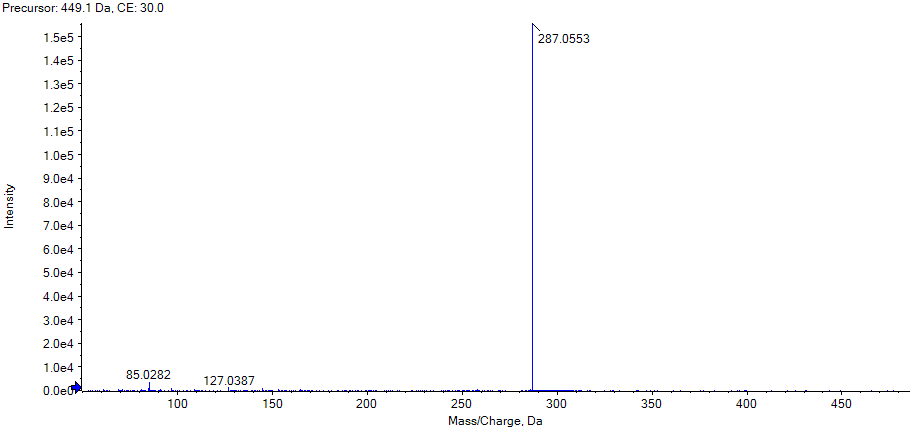 |
| 43 | Quercetin-3-O-rhamnoside(Quercitrin) | 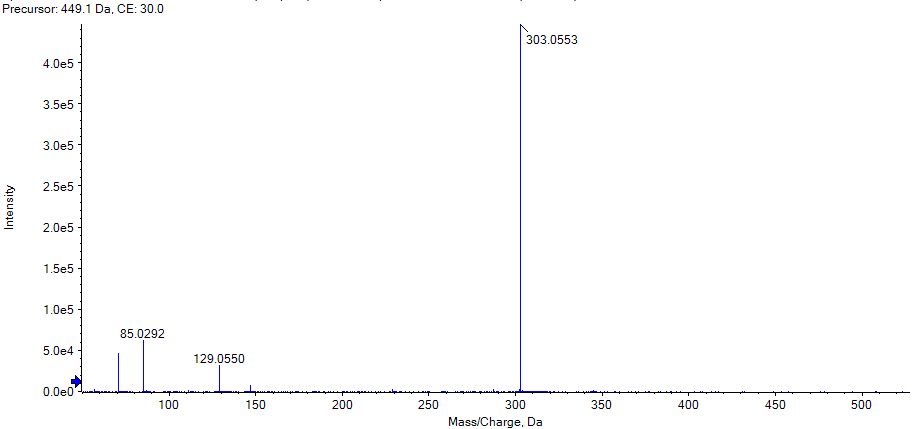 |
| 44 | Benzoic acid | 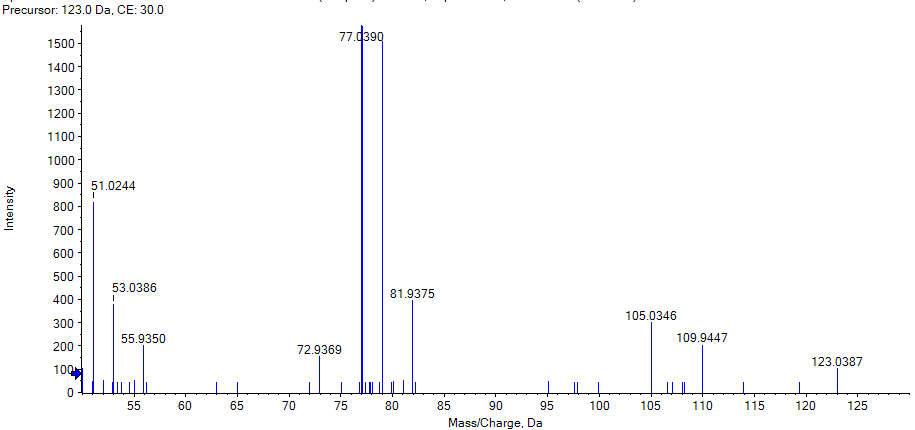 |
| 45 | Formononetin-7-O-glucoside (Ononin) | 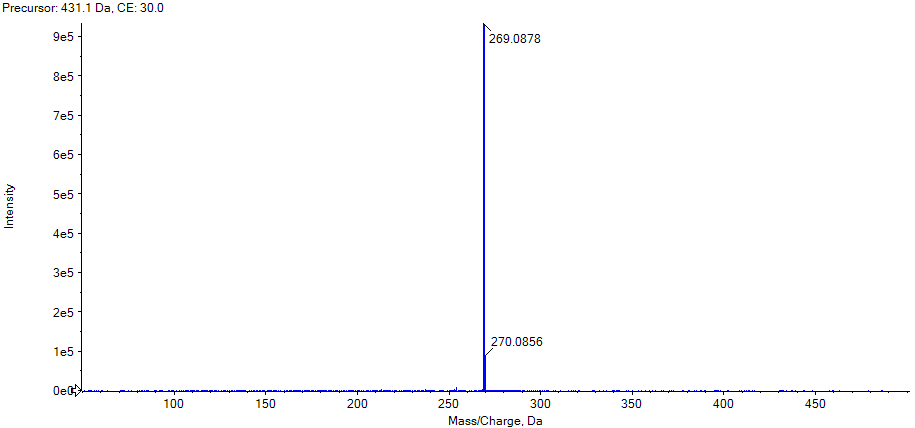 |
| 46 | Isoluteolin (Orobol)(5,7,3',4'-tetrahydroxyisoflavone) | 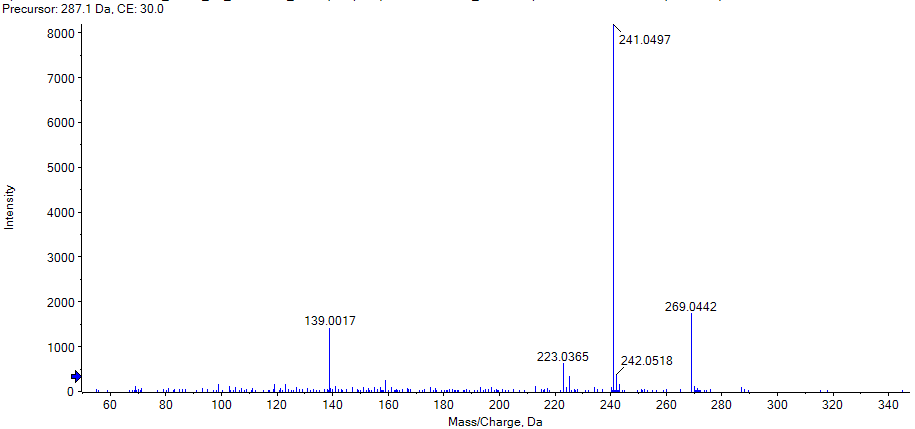 |
| 47 | Quercetin | 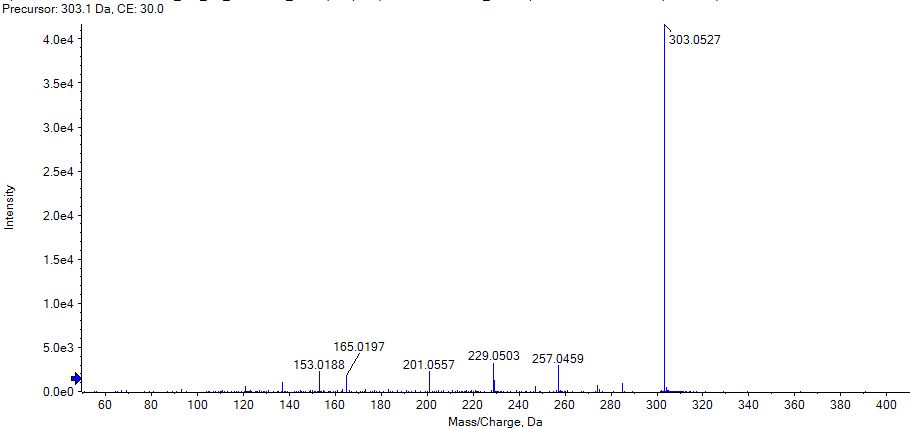 |
| 48 | Calycosin | 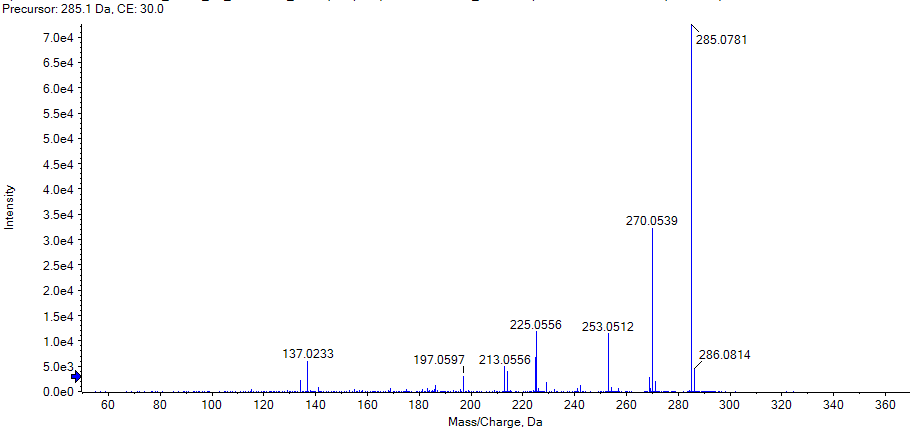 |
| 49 | Biochanin A-7-O-glucoside (Sissotrin)* | 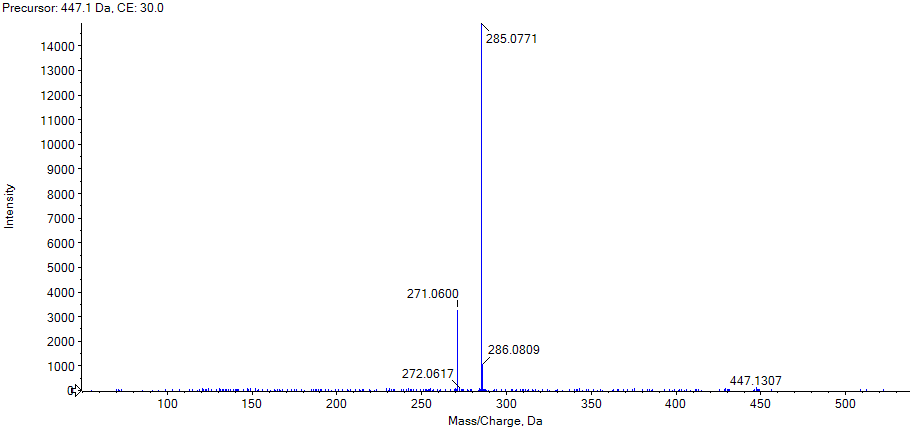 |
| 50 | Isoformononetin | 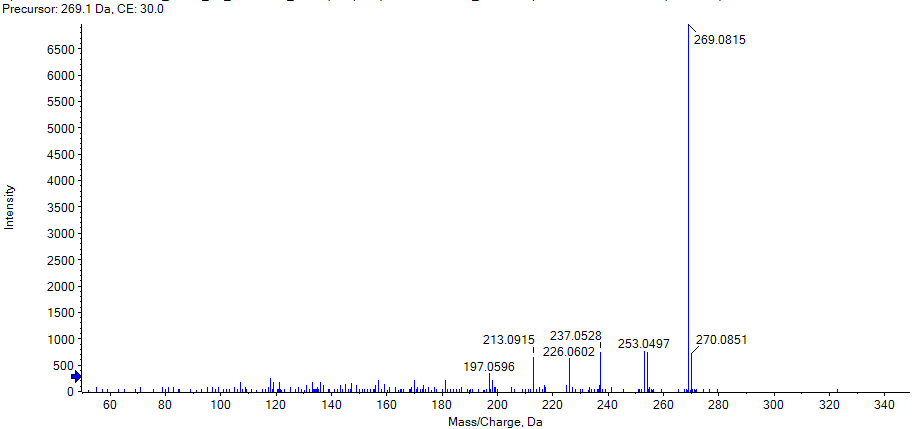 |
| 51 | Formononetin (7-Hydroxy-4'-methoxyisoflavone) | 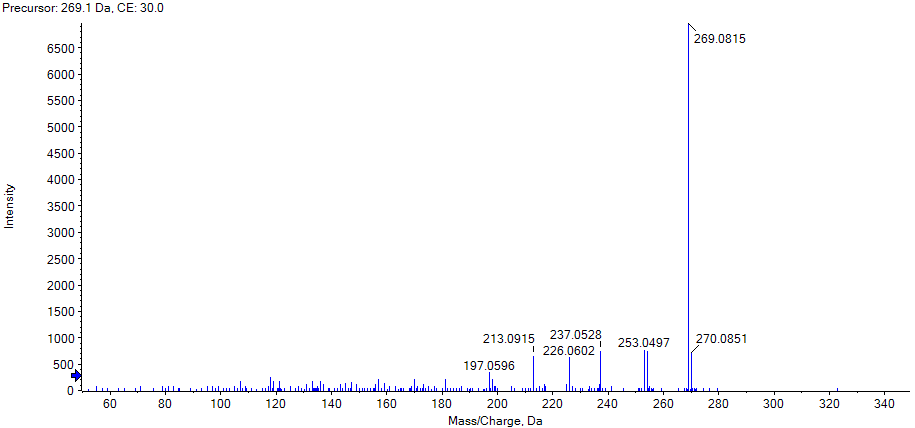 |
| 52 | Wogonin (5,7-Dihydroxy-8-Methoxyflavone) | 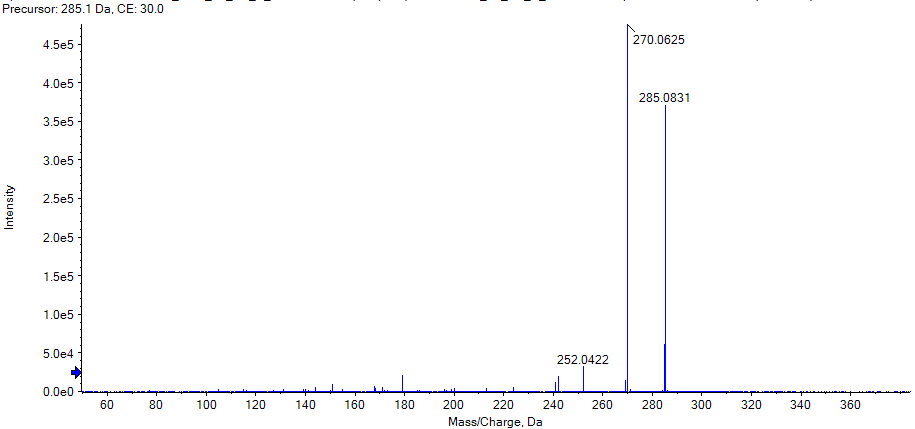 |
| 53 | Pectolinarigenin | 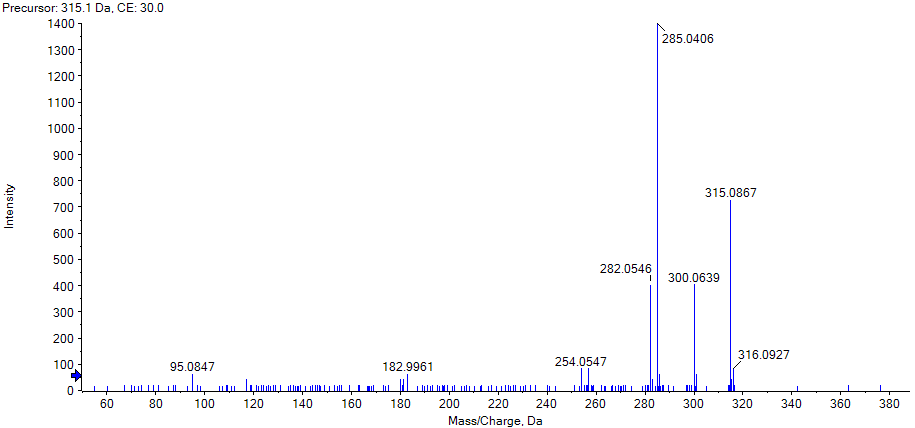 |
| 54 | Nobiletin (5,6,7,8,3',4'-Hexamethoxyflavone)* | 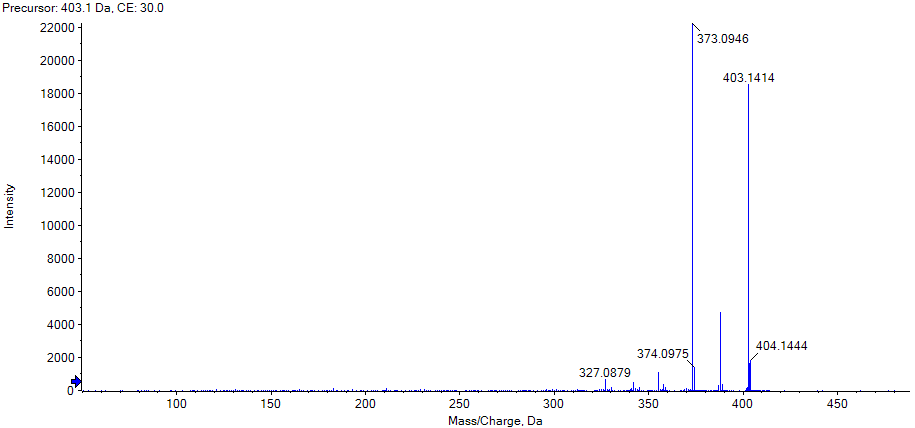 |
| 55 | 16-oxo-11-anhydroalisol A | 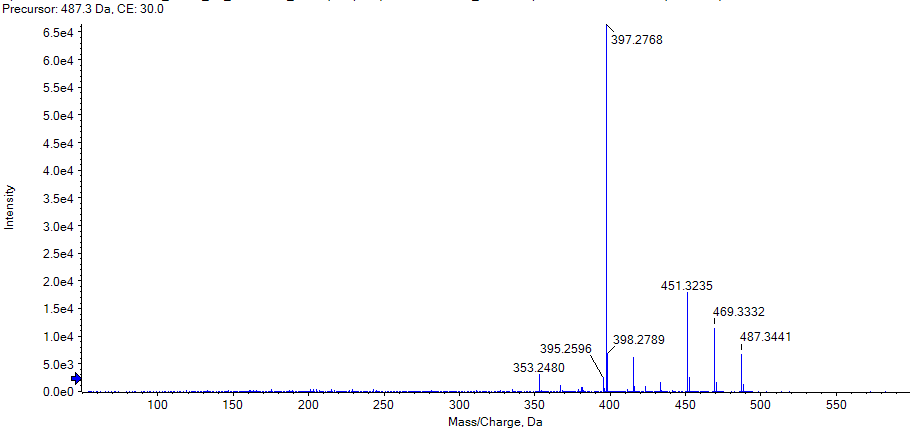 |
| 56 | alisol C,23-acetate* | 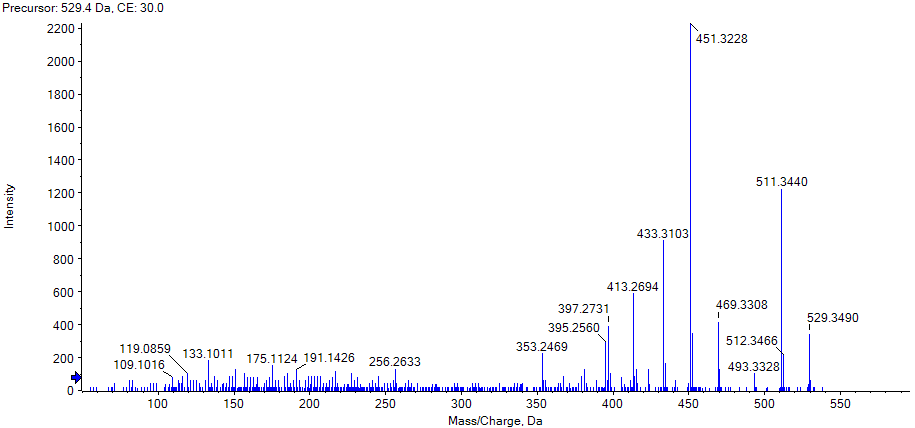 |
| 57 | Mudanpinoic acid A | 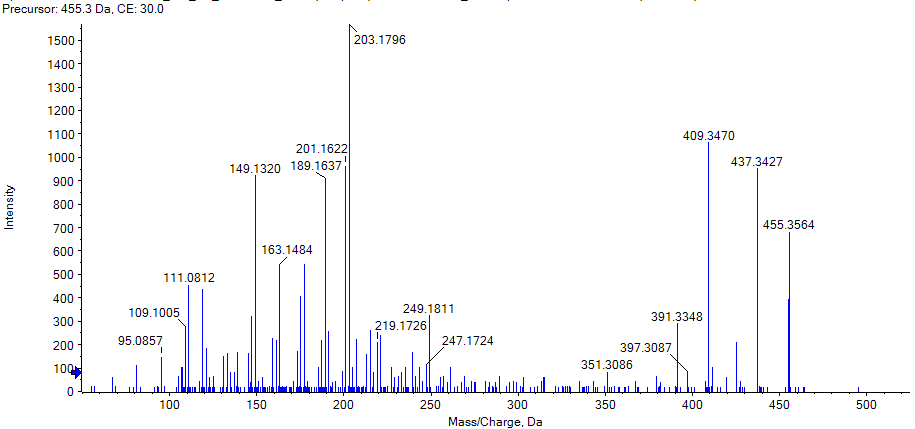 |
| 58 | 3-(Acetyloxy)-16-hydroxy-24-methylenelanost-8-en-21-oic acid (Pachymic acid) | 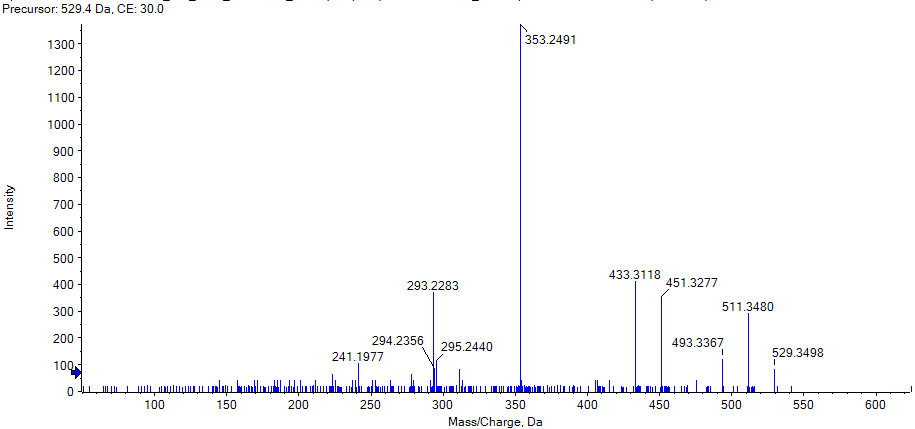 |
| 59 | 3-O-Acetyl-16-hydroxytrametenolic acid | 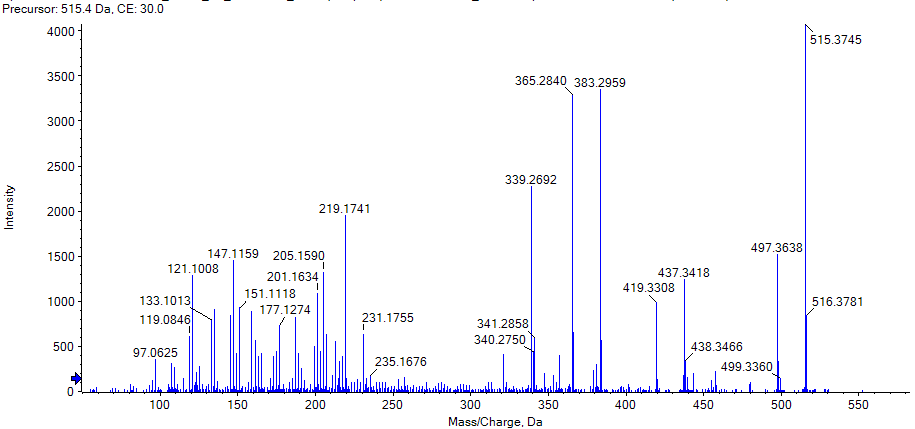 |
| 60 | Oleic acid | 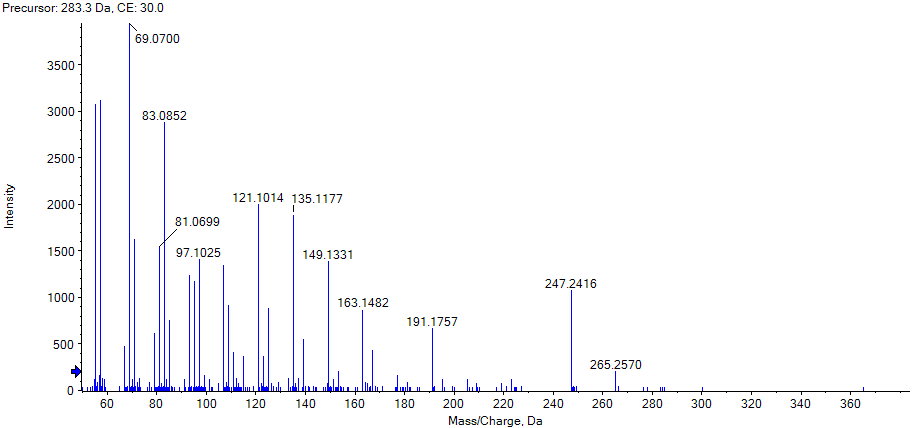 |
| 61 | Alisol B Acetate | 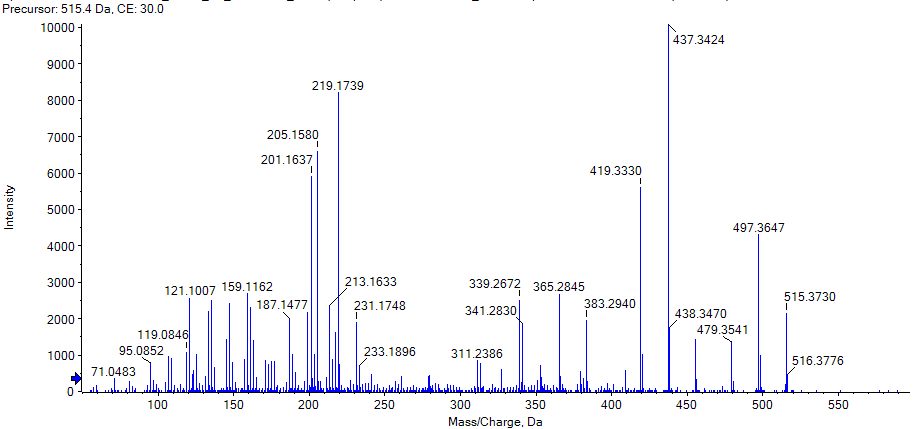 |

*calibrated with the standards.
