# Supplementary material for: ShenQi DiHuang Decoction (SQDHD) Ameliorates Neuroinflammation and Neuropsychiatric Manifestations in Pristane Induced Lupus Mice via Blocking JAK1‐STAT3 Pathway
Source: CNS Neurosci Ther. 2026 Mar 7;32(3):e70814. doi: 10.1002/cns.70814 (PMC12967629; doi:10.1002/cns.70814)
Supplement: Supplementary file 3 — Table S3: Collection of NPSLE associated targets. [file CNS-32-e70814-s004.docx]

**Table.S3 Collection of NPSLE associated targets**

|  | **DisGENT** |  |  | **GeneCards** |  |  | **CTD** |  |
| --- | --- | --- | --- | --- | --- | --- | --- | --- |
| **Gene** | **UniProt** | **Score_gda** | **Gene** | **UniProt** | **Score** | **Gene** | **UniProt** | **Inference Score** |
| ITGAM | P11215 | 0.3 | TREX1 | Q9NSU2 | 182.02 | FCGR3A | P08637 | 10 |
| FCGR3B | O75015 | 0.3 | ATRIP | Q8WXE1 | 156.58 | FCGR3B | O75015 | 10 |
| FCGR3A | P08637 | 0.3 | TNF | P01375 | 118.41 | ITGAM | P11215 | 10 |
| LAMC2 | Q13753 | 0.02 | DNASE1L3 | Q13609 | 113.33 | IL32 | P24001 | 6.09 |
| GRIN2A | Q12879 | 0.02 | C4A | P0C0L4 | 111.04 | MAGED4 | Q96JG8 | 5.51 |
| GRIN2B | Q13224 | 0.02 | HLA-DRB1 | P01911 | 107.83 | TRIML1 | Q8N9V2 | 5.47 |
| CSF2 | P04141 | 0.02 | DNASE1 | P24855 | 107.44 | G6PC2 | Q9NQR9 | 5.43 |
| AQP4 | P55087 | 0.02 | IL10 | P22301 | 107.13 | CCIN | [Q13939](https://www.uniprot.org/uniprotkb/Q13939/entry) | 5.4 |
| SBSN | Q6UWP8 | 0.02 | FCGR2A | P12318 | 98.31 | MS4A3 | Q96HJ5 | 5.37 |
|  |  |  | ITGAM | P11215 | 96.04 | CABP2 | Q9NPB3 | 5.24 |
|  |  |  | C4B | P0C0L5 | 86.76 | PTCRA | [Q6ISU1](https://www.uniprot.org/uniprotkb/Q6ISU1/entry) | 5.24 |
|  |  |  | TNFAIP3 | P21580 | 83.35 | TRIM58 | Q8NG06 | 5.22 |
|  |  |  | MECP2 | P51608 | 82.3 | FBXO40 | Q9UH90 | 5.2 |
|  |  |  | SPP1 | P10451 | 80.52 | ZNF552 | Q9H707 | 5.16 |
|  |  |  | FCGR3B | O75015 | 77.96 | MUCL1 | Q96DR8 | 5.09 |
|  |  |  | IL6 | P05231 | 77.52 | REG3A | [Q06141](https://www.uniprot.org/uniprotkb/Q06141/entry) | 5.09 |
|  |  |  | C1QA | P02745 | 76.04 | SCRT1 | [Q9BWW7](https://www.uniprot.org/uniprotkb/Q9BWW7/entry) | 5.09 |
|  |  |  | UBE2L3 | P68036 | 67.57 | UPK2 | O00526 | 5.09 |
|  |  |  | SLC22A5 | O76082 | 66.83 | ZC3H12B | Q5HYM0 | 5.09 |
|  |  |  | C1R | P00736 | 60.77 | ZNF671 | Q8TAW3 | 5.06 |
|  |  |  | ACE | P12821 | 58.52 | BPI | P17213 | 5.04 |
|  |  |  | IFNG | P01579 | 58.21 | RAPSN | [Q13702](https://www.uniprot.org/uniprotkb/Q13702/entry) | 5.04 |
|  |  |  | CP | P00450 | 57.16 | HM13 | Q8TCT9 | 5.01 |
|  |  |  | HLA-DQB1 | P01920 | 53.63 | NUP42 | O15504 | 5 |
|  |  |  | C3 | P01024 | 51 | CD8B | P10966 | 4.98 |
|  |  |  | APOH | P02749 | 50.87 | DNTT | P04053 | 4.98 |
|  |  |  | C1QC | P02747 | 50.07 | GPR20 | Q99678 | 4.98 |
|  |  |  | KIT | P10721 | 49.29 | ZBTB33 | Q86T24 | 4.97 |
|  |  |  | PRKCD | Q05655 | 49.18 | ZDHHC1 | Q8WTX9 | 4.97 |
|  |  |  | TP53 | P04637 | 47.21 | RNASE2 | P10153 | 4.96 |
|  |  |  | CRP | P02741 | 46.35 | MMEL1 | Q495T6 | 4.94 |
|  |  |  | FCGR3A | P08637 | 45.99 | MSS51 | Q4VC12 | 4.93 |
|  |  |  | FASLG | P48023 | 45.29 | PRDM13 | Q9H4Q3 | 4.93 |
|  |  |  | CCL2 | P13500 | 45.21 | RC3H2 | Q9HBD1 | 4.93 |
|  |  |  | IL1B | P01584 | 45.14 | SHARPIN | Q9H0F6 | 4.93 |
|  |  |  | TGFB1 | P01137 | 44.44 | SIT1 | Q9Y3P8 | 4.93 |
|  |  |  | FAS | P25445 | 44.22 | UPK3A | O75631 | 4.92 |
|  |  |  | IL2 | P60568 | 43.79 | USP44 | Q9H0E7 | 4.92 |
|  |  |  | F2 | P00734 | 42.43 | FKBPL | Q9UIM3 | 4.91 |
|  |  |  | IL17A | Q16552 | 42.41 | NPRL2 | Q8WTW4 | 4.9 |
|  |  |  | IFNA1 | P01562 | 41.86 | RING1 | Q06587 | 4.88 |
|  |  |  | IL4 | P05112 | 41.78 | CCR8 | P51685 | 4.87 |
|  |  |  | PRL | P01236 | 40.79 | NTF4 | P34130 | 4.87 |
|  |  |  | TLR9 | Q9NR96 | 40.76 | SLC18A3 | Q16572 | 4.87 |
|  |  |  | TNFSF13B | Q9Y275 | 40.54 | CST7 | O76096 | 4.86 |
|  |  |  | TLR4 | O00206 | 40.21 | CXCR6 | O00574 | 4.86 |
|  |  |  | IL18 | Q14116 | 39.57 | RNF216 | Q9NWF9 | 4.86 |
|  |  |  | TNFRSF1B | P20333 | 39.32 | B3GALNT2 | Q8NCR0 | 4.85 |
|  |  |  | AGTR1 | P30556 | 39.24 | IGKC | P01834 | 4.85 |
|  |  |  | PTEN | P60484 | 39.16 | SOCS7 | O14512 | 4.85 |
|  |  |  | MIF | P14174 | 38.82 | POLM | Q9NP87 | 4.84 |
|  |  |  | CXCL8 | P10145 | 38.02 | PRSS3 | P35030 | 4.84 |
|  |  |  | ALB | P02768 | 37.96 | RNF5 | Q99942 | 4.84 |
|  |  |  | IL2RA | P01589 | 37.93 | SGCE | O43556 | 4.84 |
|  |  |  | NOS3 | P29474 | 37.7 | GLIS1 | Q8NBF1 | 4.83 |
|  |  |  | PRNP | F7VJQ1 | 37.67 | HINFP | Q9BQA5 | 4.83 |
|  |  |  | STAT1 | P42224 | 37.32 | SP2 | Q02086 | 4.83 |
|  |  |  | SERPINE1 | P05121 | 37 | UBR7 | Q8N806 | 4.83 |
|  |  |  | SYK | P43405 | 36.75 | CCR9 | P51686 | 4.82 |
|  |  |  | STAT3 | P40763 | 36.06 | FAM234B | A2RU67 | 4.82 |
|  |  |  | BCL2 | P10415 | 35.9 | LIMD1 | Q9UGP4 | 4.82 |
|  |  |  | F3 | P13726 | 35.42 | LSG1 | Q9H089 | 4.82 |
|  |  |  | DNMT1 | P26358 | 35.22 | RNF180 | Q86T96 | 4.82 |
|  |  |  | CAT | P04040 | 35.01 | TAFA5 | Q7Z5A7 | 4.82 |
|  |  |  | IL1RN | P18510 | 34.8 | SLC39A13 | Q96H72 | 4.81 |
|  |  |  | INS | P01308 | 34.78 | TMEM267 | Q0VDI3 | 4.81 |
|  |  |  | MMP9 | P14780 | 34.68 | TNFRSF18 | Q9Y5U5 | 4.81 |
|  |  |  | CCL5 | P13501 | 34.57 | CHAMP1 | Q96JM3 | 4.8 |
|  |  |  | AKT1 | P31749 | 34.56 | ICAM3 | P32942 | 4.8 |
|  |  |  | IL1A | P01583 | 34 | CABP1 | Q9NZU7 | 4.79 |
|  |  |  | NOD2 | Q9HC29 | 33.7 | GPATCH8 | Q9UKJ3 | 4.79 |
|  |  |  | TNFRSF1A | P19438 | 33.63 | IL21 | Q9HBE4 | 4.79 |
|  |  |  | CALR | P27797 | 33.51 | SLC34A1 | Q06495 | 4.79 |
|  |  |  | CD4 | P01730 | 32.95 | BRPF1 | P55201 | 4.78 |
|  |  |  | CXCL10 | P02778 | 32.27 | DZIP1 | Q86YF9 | 4.78 |
|  |  |  | NFKB1 | P19838 | 31.55 | F2RL3 | Q96RI0 | 4.78 |
|  |  |  | PPARG | P37231 | 31.5 | GLRB | P48167 | 4.78 |
|  |  |  | ATG5 | Q9H1Y0 | 31.29 | KCNQ4 | P56696 | 4.78 |
|  |  |  | CYP2C19 | P33261 | 30.98 | SREK1IP1 | Q8N9Q2 | 4.78 |
|  |  |  | MTOR | P42345 | 30.84 | UMOD | P07911 | 4.78 |
|  |  |  | SAT1 | P21673 | 30.68 | UPK1B | O75841 | 4.78 |
|  |  |  | SNRNP70 | P08621 | 30.61 | ZBTB41 | Q5SVQ8 | 4.78 |
|  |  |  | VCAM1 | P19320 | 30.47 | ACOD1 | A6NK06 | 4.77 |
|  |  |  | NR3C1 | P04150 | 30.26 | ZZEF1 | O43149 | 4.77 |
|  |  |  | BDNF | P23560 | 30.23 | CHD5 | Q8TDI0 | 4.76 |
|  |  |  | TNFSF13 | O75888 | 30.14 | CCL25 | O15444 | 4.75 |
|  |  |  | AGT | P01019 | 29.94 | DEAF1 | O75398 | 4.75 |
|  |  |  | MYD88 | Q99836 | 29.4 | ISL2 | Q96A47 | 4.75 |
|  |  |  | ESR1 | P03372 | 29.36 | NRDC | O43847 | 4.75 |
|  |  |  | ADA | P00813 | 29.28 | OPN3 | Q9H1Y3 | 4.75 |
|  |  |  | APOE | P02649 | 29.09 | RRAGA | Q7L523 | 4.75 |
|  |  |  | HLA-B | P01889 | 28.92 | CHRM5 | P08912 | 4.74 |
|  |  |  | TNFSF12 | O43508 | 28.8 | LONRF2 | Q1L5Z9 | 4.74 |
|  |  |  | ADIPOQ | Q15848 | 28.67 | WAS | P42768 | 4.74 |
|  |  |  | IFNA2 | P01563 | 28.63 | CD2 | P06729 | 4.73 |
|  |  |  | CASP3 | P42574 | 28.53 | GALC | P54803 | 4.73 |
|  |  |  | SELP | P16109 | 28.43 | NKAIN1 | Q4KMZ8 | 4.73 |
|  |  |  | HMOX1 | P09601 | 28.39 | RC3H1 | Q5TC82 | 4.73 |
|  |  |  | CXCR3 | P49682 | 28.36 | ABI2 | Q9NYB9 | 4.72 |
|  |  |  | NOTCH1 | P46531 | 28.15 | GLOD4 | Q9HC38 | 4.72 |
|  |  |  | CBS | P35520 | 28.04 | HLA-F | P30511 | 4.72 |
|  |  |  | PON1 | P27169 | 27.97 | MARCHF7 | Q9H992 | 4.72 |
|  |  |  | APOA1 | P02647 | 27.75 | MUC13 | Q9H3R2 | 4.72 |
|  |  |  | SOD1 | P00441 | 27.52 | RNF44 | Q7L0R7 | 4.72 |
|  |  |  | NFE2L2 | Q16236 | 27.29 | RTEL1 | Q9NZ71 | 4.72 |
|  |  |  | PTGS2 | P35354 | 27.22 | TEX30 | Q5JUR7 | 4.72 |
|  |  |  | TUG1 | A0A6I8PU40 | 27.15 | TRIM32 | Q13049 | 4.72 |
|  |  |  | HSP90AA1 | P07900 | 27.01 | AMFR | Q9UKV5 | 4.71 |
|  |  |  | VEGFA | P15692 | 26.95 | FPR2 | P25090 | 4.71 |
|  |  |  | SNRPN | P63162 | 26.9 | GIMAP4 | Q9NUV9 | 4.71 |
|  |  |  | CD79A | P11912 | 26.87 | GPR18 | Q14330 | 4.71 |
|  |  |  | HLA-DRA | P01903 | 26.78 | HYLS1 | Q96M11 | 4.71 |
|  |  |  | EGFR | P00533 | 26.77 | ITK | Q08881 | 4.71 |
|  |  |  | CXCR4 | P61073 | 26.61 | SUN1 | O94901 | 4.71 |
|  |  |  | TFRC | P02786 | 26.26 | BBS4 | Q96RK4 | 4.7 |
|  |  |  | ANGPT2 | O15123 | 26.22 | NAIP | Q13075 | 4.7 |
|  |  |  | MAPT | P10636 | 26.22 | TMEFF1 | Q8IYR6 | 4.7 |
|  |  |  | FGF2 | P09038 | 25.97 | CD5 | P06127 | 4.69 |
|  |  |  | CTNNB1 | P35222 | 25.92 | CD6 | P30203 | 4.69 |
|  |  |  | LEP | P41159 | 25.83 | IRAG2 | Q12912 | 4.69 |
|  |  |  | GAS6 | Q14393 | 25.82 | LGALS7 | P47929 | 4.69 |
|  |  |  | IFNB1 | P01574 | 25.75 | UPK1A | O00322 | 4.69 |
|  |  |  | GFAP | P14136 | 25.67 | ZIC1 | Q15915 | 4.69 |
|  |  |  | CASP8 | Q14790 | 25.6 | FAU | P62861 | 4.68 |
|  |  |  | FLT1 | P17948 | 25.48 | GTF3C1 | Q12789 | 4.68 |
|  |  |  | ISG15 | P05161 | 25.44 | MAGED1 | Q9Y5V3 | 4.68 |
|  |  |  | IL1R1 | P14778 | 25.09 | PMS2 | P54278 | 4.68 |
|  |  |  | HSPA1B | P0DMV9 | 25.08 | SLC39A5 | Q6ZMH5 | 4.68 |
|  |  |  | LCN2 | P80188 | 25.07 | TUG1 | A0A6I8PU40 | 4.68 |
|  |  |  | SEMA3A | Q14563 | 24.73 | VIPR2 | P41587 | 4.68 |
|  |  |  | TRAF6 | Q9Y4K3 | 24.62 | CTNNBIP1 | Q9NSA3 | 4.67 |
|  |  |  | RNPC3 | Q96LT9 | 24.6 | MPC2 | O95563 | 4.67 |
|  |  |  | VDR | P11473 | 24.54 | P2RX3 | P56373 | 4.67 |
|  |  |  | JAK1 | P23458 | 24.44 | ALYREF | Q86V81 | 4.66 |
|  |  |  | MTHFR | P42898 | 24.4 | CD27 | P26842 | 4.66 |
|  |  |  | SLC45A1 | Q9Y2W3 | 24.32 | GFI1 | Q99684 | 4.66 |
|  |  |  | CXCR5 | P32302 | 24.29 | IL15RA | Q13261 | 4.66 |
|  |  |  | TBP | P20226 | 24.25 | METRN | Q9UJH8 | 4.66 |
|  |  |  | CD244 | Q9BZW8 | 24.23 | NDUFS2 | O75306 | 4.66 |
|  |  |  | MAPK8 | P45983 | 24.18 | NR2C2 | P49116 | 4.66 |
|  |  |  | HSPD1 | P10809 | 24.18 | RAG1 | P15918 | 4.66 |
|  |  |  | MYC | P01106 | 24.1 | REG3G | Q6UW15 | 4.66 |
|  |  |  | DRD4 | P21917 | 24.01 | RIPPLY3 | P57055 | 4.66 |
|  |  |  | CXCL13 | O43927 | 23.86 | CD84 | Q9UIB8 | 4.65 |
|  |  |  | IFNAR1 | P17181 | 23.75 | DPF3 | Q92784 | 4.65 |
|  |  |  | GBA1 | P04062 | 23.63 | FOXP4 | Q8IVH2 | 4.65 |
|  |  |  | IGF1 | P05019 | 23.63 | KDM2A | Q9Y2K7 | 4.65 |
|  |  |  | SLC6A4 | P31645 | 23.62 | NANOS1 | Q8WY41 | 4.65 |
|  |  |  | CX3CR1 | P49238 | 23.6 | NT5C2 | P49902 | 4.65 |
|  |  |  | CLU | P10909 | 23.49 | AKAP8 | O43823 | 4.64 |
|  |  |  | USP18 | Q9UMW8 | 23.39 | CRHR2 | Q13324 | 4.64 |
|  |  |  | CCR7 | P32248 | 23.36 | EDA2R | Q9HAV5 | 4.64 |
|  |  |  | SNRPD1 | P62314 | 23.33 | PCSK2 | P16519 | 4.64 |
|  |  |  | MBP | P02686 | 23.31 | PRTN3 | P24158 | 4.64 |
|  |  |  | CSF1 | P09603 | 23.29 | SCG3 | Q8WXD2 | 4.64 |
|  |  |  | FOXO1 | Q12778 | 23.28 | SCUBE2 | Q9NQ36 | 4.64 |
|  |  |  | SULT1A3 | P0DMM9 | 23.24 | SIRT5 | Q9NXA8 | 4.64 |
|  |  |  | CCL3 | P10147 | 23.07 | ZHX3 | Q9H4I2 | 4.64 |
|  |  |  | FGFR1 | P11362 | 23.04 | NRAP | Q86VF7 | 4.63 |
|  |  |  | MAPK1 | P28482 | 22.98 | CTNND2 | Q9UQB3 | 4.62 |
|  |  |  | NFKBIA | P25963 | 22.95 | HCRT | O43612 | 4.62 |
|  |  |  | POMC | P01189 | 22.86 | IFNA1 | P01562 | 4.62 |
|  |  |  | P2RX7 | Q99572 | 22.81 | MDFIC | Q9P1T7 | 4.62 |
|  |  |  | NOS2 | P35228 | 22.77 | STMN3 | Q9NZ72 | 4.62 |
|  |  |  | ABCB1 | P08183 | 22.76 | BSCL2 | Q96G97 | 4.61 |
|  |  |  | IL1RAPL2 | Q9NP60 | 22.7 | CD79B | P40259 | 4.61 |
|  |  |  | CST3 | P01034 | 22.68 | MAN2B2 | Q9Y2E5 | 4.61 |
|  |  |  | CD38 | P28907 | 22.48 | MSH3 | P20585 | 4.61 |
|  |  |  | CREB1 | P16220 | 22.47 | NETO2 | Q8NC67 | 4.61 |
|  |  |  | PDGFRB | P09619 | 22.46 | PAIP1 | Q9H074 | 4.61 |
|  |  |  | SNCA | P37840 | 22.41 | ADCYAP1R1 | P41586 | 4.6 |
|  |  |  | FBN1 | P35555 | 22.38 | BUB3 | O43684 | 4.6 |
|  |  |  | HAMP | P81172 | 22.37 | CD3E | P07766 | 4.6 |
|  |  |  | TPO | P07202 | 22.27 | CD3G | P09693 | 4.6 |
|  |  |  | AXL | P30530 | 22.24 | CYP2C18 | P33260 | 4.6 |
|  |  |  | SPTAN1 | Q13813 | 22.22 | ERG | P11308 | 4.6 |
|  |  |  | CCL17 | Q92583 | 22.14 | F2RL2 | O00254 | 4.6 |
|  |  |  | LRRK2 | Q5S007 | 22.07 | KBTBD11 | O94819 | 4.6 |
|  |  |  | IDO1 | P14902 | 22.04 | TM6SF1 | Q9BZW5 | 4.6 |
|  |  |  | KMT2D | O14686 | 21.98 | ABHD3 | Q8WU67 | 4.59 |
|  |  |  | CXCL9 | Q07325 | 21.69 | KIF3A | Q9Y496 | 4.59 |
|  |  |  | CDKN2A | Q8N726 | 21.6 | LAT | O43561 | 4.59 |
|  |  |  | TSC2 | P49815 | 21.53 | EIF1AX | P47813 | 4.58 |
|  |  |  | JAK2 | O60674 | 21.43 | GBP3 | Q9H0R5 | 4.58 |
|  |  |  | TH | P07101 | 21.4 | KDM4B | O94953 | 4.58 |
|  |  |  | TTR | P02766 | 21.35 | KRCC1 | Q9NPI7 | 4.58 |
|  |  |  | HSP90AB1 | P08238 | 21.34 | MAP3K12 | Q12852 | 4.58 |
|  |  |  | NRP1 | O14786 | 21.31 | THNSL2 | Q86YJ6 | 4.58 |
|  |  |  | IL12B | P29460 | 21.08 | TMED3 | Q9Y3Q3 | 4.58 |
|  |  |  | PTPN11 | Q06124 | 21.03 | TNFRSF14 | Q92956 | 4.58 |
|  |  |  | PLA2G6 | O60733 | 20.97 | GTF2B | Q00403 | 4.57 |
|  |  |  | CBL | P22681 | 20.96 | KRT80 | Q6KB66 | 4.57 |
|  |  |  | SIRT1 | Q96EB6 | 20.78 | PALM2AKAP2 | Q9Y2D5 | 4.57 |
|  |  |  | CX3CL1 | P78423 | 20.77 | PURA | Q00577 | 4.57 |
|  |  |  | HLA-DRB5 | Q30154 | 20.76 | SSRP1 | Q08945 | 4.57 |
|  |  |  | CACNA1A | O00555 | 20.62 | COX7A2 | P14406 | 4.56 |
|  |  |  | NPY | P01303 | 20.56 | DDB1 | Q16531 | 4.56 |
|  |  |  | NGF | P01138 | 20.5 | FCGRT | P55899 | 4.56 |
|  |  |  | RPLP0 | P05388 | 20.49 | GNG12 | Q9UBI6 | 4.56 |
|  |  |  | GDNF | P39905 | 20.3 | KIF21B | O75037 | 4.56 |
|  |  |  | HFE | Q30201 | 20.07 | PLOD1 | Q02809 | 4.55 |
|  |  |  | NAT2 | P11245 | 20.05 | CD3D | P04234 | 4.54 |
|  |  |  | MSN | P26038 | 20.01 | FBXW7 | Q969H0 | 4.54 |
|  |  |  | POLG | P54098 | 19.77 | MMP11 | P24347 | 4.54 |
|  |  |  | NF1 | P21359 | 19.74 | PTGES3 | Q15185 | 4.54 |
|  |  |  | SLC6A3 | Q01959 | 19.71 | SLIT1 | O75093 | 4.54 |
|  |  |  | COMT | P21964 | 19.54 | STK4 | Q13043 | 4.54 |
|  |  |  | CYP2D6 | P10635 | 19.47 | CD151 | P48509 | 4.53 |
|  |  |  | S100B | P04271 | 19.43 | EVI2A | P22794 | 4.53 |
|  |  |  | PNP | P00491 | 19.38 | FKBP1B | P68106 | 4.53 |
|  |  |  | DRD2 | P14416 | 19.25 | ICA1 | Q05084 | 4.53 |
|  |  |  | PRKN | O60260 | 19.11 | PLOD3 | O60568 | 4.53 |
|  |  |  | DNMT3A | Q9Y6K1 | 19.1 | USP36 | Q9P275 | 4.53 |
|  |  |  | CRH | P06850 | 19.01 | ARHGEF26 | Q96DR7 | 4.52 |
|  |  |  | SNRPB | P14678 | 18.94 | CD28 | P10747 | 4.52 |
|  |  |  | CYP2B6 | P20813 | 18.89 | JCHAIN | P01591 | 4.52 |
|  |  |  | ACHE | P22303 | 18.84 | MAPK7 | Q13164 | 4.52 |
|  |  |  | APP | P05067 | 18.77 | MED13L | Q71F56 | 4.52 |
|  |  |  | MTR | Q99707 | 18.77 | MPG | P29372 | 4.52 |
|  |  |  | FLNA | P21333 | 18.7 | PIGR | P01833 | 4.52 |
|  |  |  | ALYREF | Q86V81 | 18.66 | PMS1 | P54277 | 4.52 |
|  |  |  | RUNX1 | Q01196 | 18.49 | PTGER1 | P34995 | 4.52 |
|  |  |  | CAMK4 | Q16566 | 18.36 | SALL1 | Q9NSC2 | 4.52 |
|  |  |  | CREBBP | Q92793 | 18.35 | SLC2A6 | Q9UGQ3 | 4.52 |
|  |  |  | SST | P61278 | 18.22 | SLC43A1 | O75387 | 4.52 |
|  |  |  | MAOA | P21397 | 18.16 | LHX2 | P50458 | 4.51 |
|  |  |  | PPIA | P62937 | 18.08 | REV1 | Q9UBZ9 | 4.51 |
|  |  |  | COQ2 | Q96H96 | 17.8 | SEC23A | Q15436 | 4.51 |
|  |  |  | GRIN2B | Q13224 | 17.77 | SLC39A6 | Q13433 | 4.51 |
|  |  |  | FOS | P01100 | 17.71 | VIPR1 | P32241 | 4.51 |
|  |  |  | ORM1 | P02763 | 17.68 | ATG3 | Q9NT62 | 4.5 |
|  |  |  | NEU1 | Q99519 | 17.66 | EGR4 | Q05215 | 4.5 |
|  |  |  | SCN5A | Q14524 | 17.65 | KDM1A | O60341 | 4.5 |
|  |  |  | NKX2-1 | P43699 | 17.28 | VIP | P01282 | 4.5 |
|  |  |  | IGF2 | P01344 | 17.21 | AGL | P35573 | 4.49 |
|  |  |  | ACE2 | Q9BYF1 | 17.16 | BAG1 | Q99933 | 4.49 |
|  |  |  | NPPA | P01160 | 17.1 | CD4 | P01730 | 4.49 |
|  |  |  | IL12A | P29459 | 17.03 | CD8A | P01732 | 4.49 |
|  |  |  | IL34 | Q6ZMJ4 | 17 | DPYSL4 | O14531 | 4.49 |
|  |  |  | GSTP1 | P09211 | 16.98 | ERAP1 | Q9NZ08 | 4.49 |
|  |  |  | GALC | P54803 | 16.97 | GAS2 | O43903 | 4.49 |
|  |  |  | KCNQ1 | P51787 | 16.97 | GPR137B | O60478 | 4.49 |
|  |  |  | SCN8A | Q9UQD0 | 16.97 | PARD3 | Q8TEW0 | 4.49 |
|  |  |  | PAH | P00439 | 16.92 | TLR9 | Q9NR96 | 4.49 |
|  |  |  | GRN | P28799 | 16.89 | CSTA | P01040 | 4.48 |
|  |  |  | DSP | P15924 | 16.89 | EPB41L2 | O43491 | 4.48 |
|  |  |  | JUN | P05412 | 16.63 | FAM107B | Q9H098 | 4.48 |
|  |  |  | CYBB | P04839 | 16.61 | PRKCG | P05129 | 4.48 |
|  |  |  | MEN1 | O00255 | 16.59 | ZAP70 | P43403 | 4.48 |
|  |  |  | KITLG | P21583 | 16.42 | ZFR | Q96KR1 | 4.48 |
|  |  |  | CYP19A1 | P11511 | 16.41 | CCL8 | P80075 | 4.47 |
|  |  |  | GTF2I | P78347 | 16.36 | CHGA | P10645 | 4.47 |
|  |  |  | ATXN2 | Q99700 | 16.33 | EMP3 | P54852 | 4.47 |
|  |  |  | ATP7B | P35670 | 16.27 | KLF13 | Q9Y2Y9 | 4.47 |
|  |  |  | WWOX | Q9NZC7 | 16.25 | PIGA | P37287 | 4.47 |
|  |  |  | NR4A2 | P43354 | 16.23 | ST13 | P50502 | 4.47 |
|  |  |  | DYNC1H1 | Q14204 | 16.16 | TBX21 | Q9UL17 | 4.47 |
|  |  |  | MDM2 | Q00987 | 16.04 | USP5 | P45974 | 4.47 |
|  |  |  | NTRK2 | Q16620 | 15.95 | BCL2L2 | Q92843 | 4.46 |
|  |  |  | GRIN2A | Q12879 | 15.93 | MR1 | Q95460 | 4.46 |
|  |  |  | CPT2 | P23786 | 15.92 | OSM | P13725 | 4.46 |
|  |  |  | PSAP | P07602 | 15.86 | STK39 | Q9UEW8 | 4.46 |
|  |  |  | HPRT1 | P00492 | 15.86 | ADAMTS9 | Q9P2N4 | 4.45 |
|  |  |  | DBH | P09172 | 15.85 | CEL | P19835 | 4.45 |
|  |  |  | SLC7A5 | Q01650 | 15.8 | ERCC2 | P18074 | 4.45 |
|  |  |  | TERT | O14746 | 15.75 | GZMA | P12544 | 4.45 |
|  |  |  | TSC1 | Q92574 | 15.74 | MLLT11 | Q13015 | 4.45 |
|  |  |  | HTR2A | P28223 | 15.73 | NFATC3 | Q12968 | 4.45 |
|  |  |  | GJA1 | P17302 | 15.68 | NFE2L3 | Q9Y4A8 | 4.45 |
|  |  |  | BAX | Q07812 | 15.67 | RPS6KA2 | Q15349 | 4.45 |
|  |  |  | CYP21A2 | P08686 | 15.64 | ST14 | Q9Y5Y6 | 4.45 |
|  |  |  | NEFL | P07196 | 15.62 | ADCYAP1 | P18509 | 4.44 |
|  |  |  | TNFSF10 | P50591 | 15.55 | CX3CR1 | P49238 | 4.44 |
|  |  |  | ESR2 | Q92731 | 15.53 | ERBB4 | Q15303 | 4.44 |
|  |  |  | HTR1A | P08908 | 15.51 | FNDC3B | Q53EP0 | 4.44 |
|  |  |  | CSF1R | P07333 | 15.48 | IGF2BP3 | O00425 | 4.44 |
|  |  |  | SOD2 | P04179 | 15.35 | PELI1 | Q96FA3 | 4.44 |
|  |  |  | CNTF | P26441 | 15.35 | RAD54L | Q92698 | 4.44 |
|  |  |  | VIP | P01282 | 15.3 | SATB1 | Q01826 | 4.44 |
|  |  |  | PARP1 | P09874 | 15.3 | SNX10 | Q9Y5X0 | 4.44 |
|  |  |  | ATL1 | Q8WXF7 | 15.29 | TLR6 | Q9Y2C9 | 4.44 |
|  |  |  | MOG | Q16653 | 15.28 | CCT7 | Q99832 | 4.43 |
|  |  |  | MT-CYB | P00156 | 15.26 | CDKN2D | P55273 | 4.43 |
|  |  |  | PEPD | P12955 | 15.19 | COCH | O43405 | 4.43 |
|  |  |  | GHRL | Q9UBU3 | 15.19 | HOXA1 | P49639 | 4.43 |
|  |  |  | MAPK3 | P27361 | 15.07 | IL3 | P08700 | 4.43 |
|  |  |  | NTRK1 | P04629 | 14.99 | LTBR | P36941 | 4.43 |
|  |  |  | DMD | P11532 | 14.92 | MMP8 | P22894 | 4.43 |
|  |  |  | IVL | P07476 | 14.88 | MXI1 | P50539 | 4.43 |
|  |  |  | DDC | P20711 | 14.8 | RPA2 | P15927 | 4.43 |
|  |  |  | GAL | P22466 | 14.79 | RUNX3 | Q13761 | 4.43 |
|  |  |  | GLUL | P15104 | 14.75 | TYROBP | O43914 | 4.43 |
|  |  |  | SYNGAP1 | Q96PV0 | 14.73 | CA12 | O43570 | 4.42 |
|  |  |  | NOS1 | P29475 | 14.72 | CLIP1 | P30622 | 4.42 |
|  |  |  | CNR1 | P21554 | 14.68 | ILF3 | Q12906 | 4.42 |
|  |  |  | CHGA | P10645 | 14.67 | P2RY2 | P41231 | 4.42 |
|  |  |  | SLC7A7 | Q9UM01 | 14.66 | SOAT1 | P35610 | 4.42 |
|  |  |  | IL4R | P24394 | 14.64 | TCF7 | P36402 | 4.42 |
|  |  |  | TYROBP | O43914 | 14.64 | BDKRB1 | P46663 | 4.41 |
|  |  |  | HSPA4 | P34932 | 14.6 | DLC1 | Q96QB1 | 4.41 |
|  |  |  | ATRX | P46100 | 14.6 | FCER1G | P30273 | 4.41 |
|  |  |  | NAGLU | P54802 | 14.59 | RAMP1 | O60894 | 4.41 |
|  |  |  | SMARCA4 | P51532 | 14.45 | TPD52 | P55327 | 4.41 |
|  |  |  | FUS | P35637 | 14.44 | BTG3 | Q14201 | 4.4 |
|  |  |  | IGF1R | P08069 | 14.34 | CACNA1H | O95180 | 4.4 |
|  |  |  | NPPB | P16860 | 14.28 | CCT5 | P48643 | 4.4 |
|  |  |  | GRM1 | Q13255 | 14.25 | CDH3 | P22223 | 4.4 |
|  |  |  | SNRPE | P62304 | 14.23 | CFI | P05156 | 4.4 |
|  |  |  | FMR1 | Q06787 | 14.23 | CSK | P41240 | 4.4 |
|  |  |  | GABBR1 | Q9UBS5 | 14.22 | GBP1 | P32455 | 4.4 |
|  |  |  | HSPA1A | P0DMV8 | 14.2 | GZMB | P10144 | 4.4 |
|  |  |  | ARX | Q96QS3 | 14.13 | STOM | P27105 | 4.4 |
|  |  |  | FGF8 | P55075 | 13.99 | TGFBR3 | Q03167 | 4.4 |
|  |  |  | ANK3 | Q12955 | 13.95 | XAF1 | Q6GPH4 | 4.4 |
|  |  |  | SLC22A4 | Q9H015 | 13.9 | XRCC6 | P12956 | 4.4 |
|  |  |  | VPS13B | Q7Z7G8 | 13.87 | AMH | P03971 | 4.39 |
|  |  |  | GSR | P00390 | 13.85 | E2F3 | O00716 | 4.39 |
|  |  |  | LEPR | P48357 | 13.84 | HMOX2 | P30519 | 4.39 |
|  |  |  | TARDBP | Q13148 | 13.83 | MTR | Q99707 | 4.39 |
|  |  |  | SCN1A | P35498 | 13.81 | NCOA7 | Q8NI08 | 4.39 |
|  |  |  | XDH | P47989 | 13.69 | RARG | P13631 | 4.39 |
|  |  |  | ENO2 | P09104 | 13.66 | MELK | Q14680 | 4.38 |
|  |  |  | RAF1 | P04049 | 13.64 | NID1 | P14543 | 4.38 |
|  |  |  | TSPO | B1AH88 | 13.63 | RPA1 | P27694 | 4.38 |
|  |  |  | ERBB4 | Q15303 | 13.61 | CCT3 | P49368 | 4.37 |
|  |  |  | NGFR | P08138 | 13.61 | DUSP2 | Q05923 | 4.37 |
|  |  |  | CYP1A2 | P05177 | 13.58 | LMCD1 | Q9NZU5 | 4.37 |
|  |  |  | BCL6 | P41182 | 13.54 | RBPMS | Q93062 | 4.37 |
|  |  |  | RPS27A | P62979 | 13.51 | TM4SF1 | P30408 | 4.37 |
|  |  |  | CCK | P06307 | 13.49 | CRABP2 | P29373 | 4.36 |
|  |  |  | SNRPD3 | P62318 | 13.43 | DNAJB6 | O75190 | 4.36 |
|  |  |  | ERBB3 | P21860 | 13.36 | ELOVL2 | Q9NXB9 | 4.36 |
|  |  |  | NTNG2 | Q96CW9 | 13.35 | FSCN1 | Q16658 | 4.36 |
|  |  |  | AIF1 | P55008 | 13.32 | IL16 | Q14005 | 4.36 |
|  |  |  | EPO | P01588 | 13.3 | MFGE8 | Q08431 | 4.36 |
|  |  |  | RYR1 | P21817 | 13.28 | RPS3 | P23396 | 4.36 |
|  |  |  | CDH2 | P19022 | 13.28 | CXCL11 | O14625 | 4.35 |
|  |  |  | CDK5 | Q00535 | 13.26 | FOXP3 | Q9BZS1 | 4.35 |
|  |  |  | DCX | O43602 | 13.26 | G3BP1 | Q13283 | 4.35 |
|  |  |  | DNM1 | Q05193 | 13.23 | IDO1 | P14902 | 4.35 |
|  |  |  | SMPD1 | P17405 | 13.22 | IER5 | Q5VY09 | 4.35 |
|  |  |  | MAOB | P27338 | 13.2 | MLH1 | P40692 | 4.35 |
|  |  |  | NLGN3 | Q9NZ94 | 13.19 | PSMA5 | P28066 | 4.35 |
|  |  |  | HSPA8 | P11142 | 13.18 | TACR1 | P25103 | 4.35 |
|  |  |  | OPRM1 | P35372 | 13.17 | CD69 | Q07108 | 4.34 |
|  |  |  | FOXG1 | P55316 | 13.16 | FLNC | Q14315 | 4.34 |
|  |  |  | CYCS | P99999 | 13.14 | IFNGR1 | P15260 | 4.34 |
|  |  |  | ATP1A3 | P13637 | 13.13 | MYH10 | P35580 | 4.34 |
|  |  |  | MAG | P20916 | 13.09 | NASP | P49321 | 4.34 |
|  |  |  | CTSD | P07339 | 13.08 | NTF3 | P20783 | 4.34 |
|  |  |  | RELN | P78509 | 13.08 | RRAS2 | P62070 | 4.34 |
|  |  |  | EEA1 | Q15075 | 13.07 | TDO2 | P48775 | 4.34 |
|  |  |  | NCAM1 | P13591 | 13.07 | ALOX5AP | P20292 | 4.33 |
|  |  |  | XRCC1 | P18887 | 13 | GPT2 | Q8TD30 | 4.33 |
|  |  |  | ELN | P15502 | 12.96 | LASP1 | Q14847 | 4.33 |
|  |  |  | ATXN3 | P54252 | 12.9 | MX2 | P20592 | 4.33 |
|  |  |  | HTR3A | P46098 | 12.87 | NRP2 | O60462 | 4.33 |
|  |  |  | VCP | P55072 | 12.86 | PROCR | Q9UNN8 | 4.33 |
|  |  |  | PIK3C2A | O00443 | 12.85 | SRM | P19623 | 4.33 |
|  |  |  | G6PD | P11413 | 12.79 | ZFP36L1 | Q07352 | 4.33 |
|  |  |  | C9orf72 | Q96LT7 | 12.79 | ACKR3 | P25106 | 4.32 |
|  |  |  | ASS1 | P00966 | 12.76 | BIK | Q13323 | 4.32 |
|  |  |  | PTH1R | Q03431 | 12.74 | C1QA | P02745 | 4.32 |
|  |  |  | MAPK14 | Q16539 | 12.72 | FSHR | P23945 | 4.32 |
|  |  |  | HTT | P42858 | 12.66 | GFRA1 | P56159 | 4.32 |
|  |  |  | VIM | P08670 | 12.66 | LAMA5 | O15230 | 4.32 |
|  |  |  | PRKACA | P17612 | 12.66 | S100P | P25815 | 4.32 |
|  |  |  | DOCK8 | Q8NF50 | 12.65 | TNFSF9 | P41273 | 4.32 |
|  |  |  | HSPG2 | P98160 | 12.62 | TUBB2B | Q9BVA1 | 4.32 |
|  |  |  | SLC1A2 | P43004 | 12.62 | CLIC4 | Q9Y696 | 4.31 |
|  |  |  | STXBP1 | P61764 | 12.6 | IL13RA1 | P78552 | 4.31 |
|  |  |  | SLC1A1 | P43005 | 12.59 | PTPRC | P08575 | 4.31 |
|  |  |  | TRPC6 | Q9Y210 | 12.58 | TCP1 | P17987 | 4.31 |
|  |  |  | EIF2AK2 | P19525 | 12.57 | ANGPT1 | Q15389 | 4.3 |
|  |  |  | LDLR | P01130 | 12.57 | GAMT | Q14353 | 4.3 |
|  |  |  | ADRB2 | P07550 | 12.55 | HNRNPK | P61978 | 4.3 |
|  |  |  | CDKL5 | O76039 | 12.52 | RRM2B | Q7LG56 | 4.3 |
|  |  |  | KEAP1 | Q14145 | 12.49 | TNFRSF19 | Q9NS68 | 4.3 |
|  |  |  | FLT3 | P36888 | 12.43 | ARHGDIB | P52566 | 4.29 |
|  |  |  | CYP2C9 | P11712 | 12.42 | CTSH | P09668 | 4.29 |
|  |  |  | PAFAH1B1 | P43034 | 12.41 | HYOU1 | Q9Y4L1 | 4.29 |
|  |  |  | KCNH2 | Q12809 | 12.41 | LTF | P02788 | 4.29 |
|  |  |  | NOTCH3 | Q9UM47 | 12.39 | SLC39A14 | Q15043 | 4.29 |
|  |  |  | TPI1 | P60174 | 12.37 | SOCS1 | O15524 | 4.29 |
|  |  |  | UBE3A | Q05086 | 12.33 | TYMP | P19971 | 4.29 |
|  |  |  | TACR3 | P29371 | 12.3 | UBD | O15205 | 4.29 |
|  |  |  | FYN | P06241 | 12.29 | BDH1 | Q02338 | 4.28 |
|  |  |  | TUBB3 | Q13509 | 12.28 | BSG | P35613 | 4.28 |
|  |  |  | APC | P25054 | 12.28 | CD38 | P28907 | 4.28 |
|  |  |  | SLC18A2 | Q05940 | 12.27 | FHL1 | Q13642 | 4.28 |
|  |  |  | VPS13A | Q96RL7 | 12.17 | GEM | P55040 | 4.28 |
|  |  |  | GNB5 | O14775 | 12.15 | IL15 | P40933 | 4.28 |
|  |  |  | AHI1 | Q8N157 | 12.14 | LCK | P06239 | 4.28 |
|  |  |  | TSHR | P16473 | 12.05 | NTRK1 | P04629 | 4.28 |
|  |  |  | CASP1 | P29466 | 12.05 | POSTN | Q15063 | 4.28 |
|  |  |  | POU5F1 | Q01860 | 12.03 | RAD50 | Q92878 | 4.28 |
|  |  |  | LAT | O43561 | 12.03 | XPC | Q01831 | 4.28 |
|  |  |  | ATP1A2 | P50993 | 12 | CCR5 | P51681 | 4.27 |
|  |  |  | POLR1C | O15160 | 11.99 | F2RL1 | P55085 | 4.27 |
|  |  |  | FXN | Q16595 | 11.98 | RND1 | Q92730 | 4.27 |
|  |  |  | DNMT3B | Q9UBC3 | 11.94 | TNNI3 | P19429 | 4.27 |
|  |  |  | CYP2E1 | P05181 | 11.93 | BLVRB | P30043 | 4.26 |
|  |  |  | IL3 | P08700 | 11.93 | CARS1 | P49589 | 4.26 |
|  |  |  | IGFBP3 | P17936 | 11.92 | DUSP10 | Q9Y6W6 | 4.26 |
|  |  |  | SLC38A2 | Q96QD8 | 11.92 | ESRRA | P11474 | 4.26 |
|  |  |  | SYP | P08247 | 11.91 | IFIT2 | P09913 | 4.26 |
|  |  |  | POLGARF | A0A3B3IS91 | 11.9 | NANOG | Q9H9S0 | 4.26 |
|  |  |  | DGUOK | Q16854 | 11.83 | PHLDA2 | Q53GA4 | 4.26 |
|  |  |  | BCHE | P06276 | 11.82 | BDKRB2 | P30411 | 4.25 |
|  |  |  | MT-ND5 | P03915 | 11.77 | CKM | P06732 | 4.25 |
|  |  |  | NFKBIL1 | Q9UBC1 | 11.76 | CXCL3 | P19876 | 4.25 |
|  |  |  | SLC1A3 | P43003 | 11.73 | GAL | P22466 | 4.25 |
|  |  |  | GPT | P24298 | 11.73 | KLF2 | Q9Y5W3 | 4.25 |
|  |  |  | LIF | P15018 | 11.72 | PTP4A1 | Q93096 | 4.25 |
|  |  |  | STX1A | Q16623 | 11.71 | ALCAM | Q13740 | 4.24 |
|  |  |  | TAC1 | P20366 | 11.64 | GJB1 | P08034 | 4.24 |
|  |  |  | HSPB1 | P04792 | 11.62 | LDHB | P07195 | 4.24 |
|  |  |  | DRD1 | P21728 | 11.52 | ATF5 | Q9Y2D1 | 4.23 |
|  |  |  | NR3C2 | P08235 | 11.52 | ERCC1 | P07992 | 4.23 |
|  |  |  | ACP1 | P24666 | 11.49 | MSH2 | P43246 | 4.23 |
|  |  |  | CD81 | P60033 | 11.48 | NME1 | P15531 | 4.23 |
|  |  |  | BCL2L1 | Q07817 | 11.47 | THY1 | P04216 | 4.23 |
|  |  |  | TCF4 | P15884 | 11.43 | CANX | P27824 | 4.22 |
|  |  |  | OXT | P01178 | 11.42 | GUSB | P08236 | 4.22 |
|  |  |  | MAP2 | P11137 | 11.36 | IL23A | Q9NPF7 | 4.22 |
|  |  |  | DSG3 | P32926 | 11.34 | LAMC2 | Q13753 | 4.22 |
|  |  |  | CALB1 | P05937 | 11.31 | TAT | P17735 | 4.22 |
|  |  |  | GGT1 | P19440 | 11.28 | TRPA1 | O75762 | 4.22 |
|  |  |  | MB | P02144 | 11.22 | AXL | P30530 | 4.21 |
|  |  |  | GLS | O94925 | 11.2 | NFKBIZ | Q9BYH8 | 4.21 |
|  |  |  | HP | P00738 | 11.2 | PTGER4 | P35408 | 4.21 |
|  |  |  | DPP4 | P27487 | 11.16 | TUBA4A | P68366 | 4.21 |
|  |  |  | SCN2A | Q99250 | 11.13 | EMP1 | P54849 | 4.2 |
|  |  |  | PLXND1 | Q9Y4D7 | 11.11 | RORC | P51449 | 4.2 |
|  |  |  | F2RL1 | P55085 | 11.03 | RRM1 | P23921 | 4.2 |
|  |  |  | CD226 | Q15762 | 10.99 | TAP1 | Q03518 | 4.2 |
|  |  |  | TUBB | P07437 | 10.99 | UNG | P13051 | 4.2 |
|  |  |  | RPS26 | P62854 | 10.96 | ALDH3A1 | P30838 | 4.19 |
|  |  |  | MPL | P40238 | 10.96 | CLDN4 | O14493 | 4.19 |
|  |  |  | AARS1 | P49588 | 10.92 | IGFBP4 | P22692 | 4.19 |
|  |  |  | DNM1L | O00429 | 10.9 | PDIA4 | P13667 | 4.19 |
|  |  |  | HSPA5 | P11021 | 10.88 | PER1 | O15534 | 4.19 |
|  |  |  | NES | P48681 | 10.86 | STIP1 | P31948 | 4.19 |
|  |  |  | DSG1 | Q02413 | 10.83 | CAPN2 | P17655 | 4.18 |
|  |  |  | PCSK9 | Q8NBP7 | 10.76 | CFB | P00751 | 4.18 |
|  |  |  | GRIN1 | Q05586 | 10.75 | CX3CL1 | P78423 | 4.18 |
|  |  |  | SLC17A5 | Q9NRA2 | 10.73 | HSPA2 | P54652 | 4.18 |
|  |  |  | CNTNAP2 | Q9UHC6 | 10.71 | KLF5 | Q13887 | 4.18 |
|  |  |  | CDK1 | P06493 | 10.69 | MARCKS | P29966 | 4.18 |
|  |  |  | ATXN1 | P54253 | 10.69 | NGFR | P08138 | 4.18 |
|  |  |  | EIF2AK3 | Q9NZJ5 | 10.65 | SFN | P31947 | 4.18 |
|  |  |  | DHFR | P00374 | 10.62 | SLC22A3 | O75751 | 4.18 |
|  |  |  | MSX1 | P28360 | 10.6 | BTG1 | P62324 | 4.17 |
|  |  |  | STUB1 | Q9UNE7 | 10.59 | DNAJB4 | Q9UDY4 | 4.17 |
|  |  |  | NTS | P30990 | 10.58 | SORD | Q00796 | 4.17 |
|  |  |  | DYRK1A | Q13627 | 10.54 | UCP1 | P25874 | 4.17 |
|  |  |  | ARG1 | P05089 | 10.51 | XRCC1 | P18887 | 4.17 |
|  |  |  | IL17RA | Q96F46 | 10.51 | CD83 | Q01151 | 4.16 |
|  |  |  | IGFBP2 | P18065 | 10.46 | CITED2 | Q99967 | 4.16 |
|  |  |  | ATG7 | O95352 | 10.45 | FYN | P06241 | 4.16 |
|  |  |  | BIN1 | O00499 | 10.45 | GDNF | P39905 | 4.16 |
|  |  |  | NAMPT | P43490 | 10.42 | HSPE1 | P61604 | 4.16 |
|  |  |  | TG | P01266 | 10.4 | LEF1 | Q9UJU2 | 4.16 |
|  |  |  | CASP9 | P55211 | 10.31 | MTHFD2 | P13995 | 4.16 |
|  |  |  | MAF | O75444 | 10.3 | TMPRSS2 | O15393 | 4.16 |
|  |  |  | STK11 | Q15831 | 10.3 | ATF2 | P15336 | 4.15 |
|  |  |  | XRCC4 | Q13426 | 10.29 | BCL6 | P41182 | 4.15 |
|  |  |  | XK | P51811 | 10.28 | GAP43 | P17677 | 4.15 |
|  |  |  | MFGE8 | Q08431 | 10.19 | GLS | O94925 | 4.15 |
|  |  |  | SLC39A8 | Q9C0K1 | 10.18 | PDCD4 | Q53EL6 | 4.15 |
|  |  |  | AQP4 | P55087 | 10.14 | PRLR | P16471 | 4.15 |
|  |  |  | SHBG | P04278 | 10.08 | SLC22A5 | O76082 | 4.15 |
|  |  |  | FGB | P02675 | 10.07 | DCN | P07585 | 4.14 |
|  |  |  | MITF | O75030 | 10.07 | DRD1 | P21728 | 4.14 |
|  |  |  | GAD1 | Q99259 | 10.06 | GATA3 | P23771 | 4.14 |
|  |  |  | PDYN | P01213 | 10.05 | NRP1 | O14786 | 4.14 |
|  |  |  | ATP13A2 | Q9NQ11 | 10.05 | SLPI | P03973 | 4.14 |
|  |  |  | CDH23 | Q9H251 | 10.04 | STAT5B | P51692 | 4.14 |
|  |  |  | PARK7 | Q99497 | 10.01 |  |  |  |
|  |  |  | PVALB | P20472 | 9.99 |  |  |  |
|  |  |  | HADHA | P40939 | 9.93 |  |  |  |
|  |  |  | ALAD | P13716 | 9.92 |  |  |  |
|  |  |  | LAMP2 | P13473 | 9.91 |  |  |  |
|  |  |  | CAMK2G | Q13555 | 9.87 |  |  |  |
|  |  |  | MAP3K7 | O43318 | 9.84 |  |  |  |
|  |  |  | NPM1 | P06748 | 9.83 |  |  |  |
|  |  |  | ERG | P11308 | 9.82 |  |  |  |
|  |  |  | CTSB | P07858 | 9.82 |  |  |  |
|  |  |  | CLN3 | Q13286 | 9.81 |  |  |  |
|  |  |  | HMGCR | P04035 | 9.8 |  |  |  |
|  |  |  | DLG4 | P78352 | 9.72 |  |  |  |
|  |  |  | TMPO | P42166 | 9.68 |  |  |  |
|  |  |  | GABRB3 | P28472 | 9.67 |  |  |  |
|  |  |  | ACADVL | P49748 | 9.66 |  |  |  |
|  |  |  | GRIA2 | P42262 | 9.58 |  |  |  |
|  |  |  | ABCB11 | O95342 | 9.56 |  |  |  |
|  |  |  | POLR3A | O14802 | 9.54 |  |  |  |
|  |  |  | IGHM | P01871 | 9.52 |  |  |  |
|  |  |  | PAX5 | Q02548 | 9.5 |  |  |  |
|  |  |  | HLA-E | P13747 | 9.5 |  |  |  |
|  |  |  | GRM5 | P41594 | 9.5 |  |  |  |
|  |  |  | PGR | P06401 | 9.49 |  |  |  |
|  |  |  | DDAH2 | O95865 | 9.49 |  |  |  |
|  |  |  | GCH1 | P30793 | 9.46 |  |  |  |
|  |  |  | SPAST | Q9UBP0 | 9.46 |  |  |  |
|  |  |  | NR1H4 | Q96RI1 | 9.46 |  |  |  |
|  |  |  | TACR1 | P25103 | 9.44 |  |  |  |
|  |  |  | GRIA1 | P42261 | 9.44 |  |  |  |
|  |  |  | CNTN6 | Q9UQ52 | 9.43 |  |  |  |
|  |  |  | KCNT1 | Q5JUK3 | 9.41 |  |  |  |
|  |  |  | COL5A2 | P05997 | 9.39 |  |  |  |
|  |  |  | POLR3B | Q9NW08 | 9.37 |  |  |  |
|  |  |  | GSK3B | P49841 | 9.37 |  |  |  |
|  |  |  | RAI1 | Q7Z5J4 | 9.36 |  |  |  |
|  |  |  | FBXW7 | Q969H0 | 9.36 |  |  |  |
|  |  |  | ADCY5 | O95622 | 9.35 |  |  |  |
|  |  |  | GAST | P01350 | 9.35 |  |  |  |
|  |  |  | QDPR | P09417 | 9.33 |  |  |  |
|  |  |  | ALDH5A1 | P51649 | 9.31 |  |  |  |
|  |  |  | AMPH | P49418 | 9.31 |  |  |  |
|  |  |  | CSNK2A1 | P68400 | 9.29 |  |  |  |
|  |  |  | PRKG1 | Q13976 | 9.28 |  |  |  |
|  |  |  | IAPP | P10997 | 9.26 |  |  |  |
|  |  |  | FTL | P02792 | 9.23 |  |  |  |
|  |  |  | GABRA1 | P14867 | 9.18 |  |  |  |
|  |  |  | LAMP1 | P11279 | 9.18 |  |  |  |
|  |  |  | EIF2B4 | Q9UI10 | 9.17 |  |  |  |
|  |  |  | SCN11A | Q9UI33 | 9.17 |  |  |  |
|  |  |  | CCL22 | O00626 | 9.14 |  |  |  |
|  |  |  | SLC20A2 | Q08357 | 9.13 |  |  |  |
|  |  |  | PRR12 | Q9ULL5 | 9.12 |  |  |  |
|  |  |  | PINK1 | Q9BXM7 | 9.09 |  |  |  |
|  |  |  | TIMP2 | P16035 | 9.06 |  |  |  |
|  |  |  | VDAC1 | P21796 | 9.04 |  |  |  |
|  |  |  | ABAT | P80404 | 9.03 |  |  |  |
|  |  |  | ADSL | P30566 | 9.03 |  |  |  |
|  |  |  | DKK1 | O94907 | 9.02 |  |  |  |
|  |  |  | EIF4G1 | Q04637 | 9.02 |  |  |  |
|  |  |  | FLVCR1 | Q9Y5Y0 | 9.01 |  |  |  |
|  |  |  | CNBP | P62633 | 8.97 |  |  |  |
|  |  |  | HINT1 | P49773 | 8.94 |  |  |  |
|  |  |  | CCL21 | O00585 | 8.88 |  |  |  |
|  |  |  | SP1 | P08047 | 8.88 |  |  |  |
|  |  |  | RYR2 | Q92736 | 8.86 |  |  |  |
|  |  |  | HDAC9 | Q9UKV0 | 8.85 |  |  |  |
|  |  |  | NRIP1 | P48552 | 8.82 |  |  |  |
|  |  |  | DCAF17 | Q5H9S7 | 8.82 |  |  |  |
|  |  |  | SETD1A | O15047 | 8.81 |  |  |  |
|  |  |  | PLA2G2A | P14555 | 8.78 |  |  |  |
|  |  |  | TREM1 | Q9NP99 | 8.76 |  |  |  |
|  |  |  | SKP1 | P63208 | 8.76 |  |  |  |
|  |  |  | DHCR7 | Q9UBM7 | 8.71 |  |  |  |
|  |  |  | ERCC3 | P19447 | 8.7 |  |  |  |
|  |  |  | TCN2 | P20062 | 8.67 |  |  |  |
|  |  |  | PANK2 | Q9BZ23 | 8.66 |  |  |  |
|  |  |  | FBL | P22087 | 8.65 |  |  |  |
|  |  |  | SCT | P09683 | 8.62 |  |  |  |
|  |  |  | TBL1XR1 | Q9BZK7 | 8.57 |  |  |  |
|  |  |  | CHRNA4 | P43681 | 8.57 |  |  |  |
|  |  |  | RDX | P35241 | 8.57 |  |  |  |
|  |  |  | HSD3B7 | Q9H2F3 | 8.57 |  |  |  |
|  |  |  | COX5A | P20674 | 8.56 |  |  |  |
|  |  |  | PDE4A | P27815 | 8.54 |  |  |  |
|  |  |  | OGG1 | O15527 | 8.54 |  |  |  |
|  |  |  | PPOX | P50336 | 8.54 |  |  |  |
|  |  |  | VPS13C | Q709C8 | 8.54 |  |  |  |
|  |  |  | OXA1L | Q15070 | 8.48 |  |  |  |
|  |  |  | TOR1A | O14656 | 8.48 |  |  |  |
|  |  |  | XRCC3 | O43542 | 8.47 |  |  |  |
|  |  |  | KCNJ11 | Q14654 | 8.4 |  |  |  |
|  |  |  | ALDH2 | P05091 | 8.37 |  |  |  |
|  |  |  | GABRG2 | P18507 | 8.36 |  |  |  |
|  |  |  | EYS | Q5T1H1 | 8.36 |  |  |  |
|  |  |  | PIGQ | Q9BRB3 | 8.31 |  |  |  |
|  |  |  | PDE5A | O76074 | 8.3 |  |  |  |
|  |  |  | VKORC1 | Q9BQB6 | 8.29 |  |  |  |
|  |  |  | CSMD1 | Q96PZ7 | 8.26 |  |  |  |
|  |  |  | CA2 | P00918 | 8.25 |  |  |  |
|  |  |  | VPS13D | Q5THJ4 | 8.25 |  |  |  |
|  |  |  | RING1 | Q06587 | 8.24 |  |  |  |
|  |  |  | SLC40A1 | Q9NP59 | 8.22 |  |  |  |
|  |  |  | SLC12A5 | Q9H2X9 | 8.2 |  |  |  |
|  |  |  | HRH2 | P25021 | 8.18 |  |  |  |
|  |  |  | NCSTN | Q92542 | 8.17 |  |  |  |
|  |  |  | ERCC5 | P28715 | 8.14 |  |  |  |
|  |  |  | ATXN7 | O15265 | 8.14 |  |  |  |
|  |  |  | IER3 | P46695 | 8.1 |  |  |  |
|  |  |  | CRHR1 | P34998 | 8.07 |  |  |  |
|  |  |  | LAMC1 | P11047 | 8.06 |  |  |  |
|  |  |  | NR5A1 | Q13285 | 8.06 |  |  |  |
|  |  |  | TGM2 | P21980 | 8.01 |  |  |  |
|  |  |  | HNRNPDL | O14979 | 8 |  |  |  |
|  |  |  | KRT10 | P13645 | 8 |  |  |  |
|  |  |  | SLC1A4 | P43007 | 7.99 |  |  |  |
|  |  |  | DEPDC5 | O75140 | 7.98 |  |  |  |
|  |  |  | RARB | P10826 | 7.97 |  |  |  |
|  |  |  | MAP1LC3B | Q9GZQ8 | 7.95 |  |  |  |
|  |  |  | CACNA1G | O43497 | 7.94 |  |  |  |
|  |  |  | YWHAB | P31946 | 7.93 |  |  |  |
|  |  |  | SYN2 | Q92777 | 7.9 |  |  |  |
|  |  |  | FABP4 | P15090 | 7.9 |  |  |  |
|  |  |  | APAF1 | O14727 | 7.89 |  |  |  |
|  |  |  | KDM6B | O15054 | 7.86 |  |  |  |
|  |  |  | GFRA1 | P56159 | 7.84 |  |  |  |
|  |  |  | ATG16L1 | Q676U5 | 7.84 |  |  |  |
|  |  |  | PER2 | O15055 | 7.83 |  |  |  |
|  |  |  | ERVW-1 | Q9UQF0 | 7.83 |  |  |  |
|  |  |  | CD47 | Q08722 | 7.81 |  |  |  |
|  |  |  | SEPTIN2 | Q15019 | 7.78 |  |  |  |
|  |  |  | INPP5D | Q92835 | 7.77 |  |  |  |
|  |  |  | TNK2 | Q07912 | 7.76 |  |  |  |
|  |  |  | SETD1B | Q9UPS6 | 7.76 |  |  |  |
|  |  |  | SLC25A15 | Q9Y619 | 7.73 |  |  |  |
|  |  |  | ATN1 | P54259 | 7.71 |  |  |  |
|  |  |  | CNTN1 | Q12860 | 7.69 |  |  |  |
|  |  |  | HIVEP3 | Q5T1R4 | 7.68 |  |  |  |
|  |  |  | DEK | P35659 | 7.67 |  |  |  |
|  |  |  | BECN1 | Q14457 | 7.67 |  |  |  |
|  |  |  | CHD4 | Q14839 | 7.66 |  |  |  |
|  |  |  | HRH1 | P35367 | 7.66 |  |  |  |
|  |  |  | CALB2 | P22676 | 7.65 |  |  |  |
|  |  |  | HSP90B1 | P14625 | 7.64 |  |  |  |
|  |  |  | IREB2 | P48200 | 7.63 |  |  |  |
|  |  |  | KL | Q9UEF7 | 7.63 |  |  |  |
|  |  |  | KANSL1 | Q7Z3B3 | 7.62 |  |  |  |
|  |  |  | CPOX | P36551 | 7.61 |  |  |  |
|  |  |  | ATXN10 | Q9UBB4 | 7.61 |  |  |  |
|  |  |  | DDIT3 | P35638 | 7.6 |  |  |  |
|  |  |  | ADORA1 | P30542 | 7.6 |  |  |  |
|  |  |  | HRH3 | Q9Y5N1 | 7.58 |  |  |  |
|  |  |  | SLC36A1 | Q7Z2H8 | 7.55 |  |  |  |
|  |  |  | ABCA7 | Q8IZY2 | 7.53 |  |  |  |
|  |  |  | TRAP1 | Q12931 | 7.52 |  |  |  |
|  |  |  | ACADS | P16219 | 7.52 |  |  |  |
|  |  |  | EPHA3 | P29320 | 7.52 |  |  |  |
|  |  |  | TANC2 | Q9HCD6 | 7.51 |  |  |  |
|  |  |  | PRRT2 | Q7Z6L0 | 7.51 |  |  |  |
|  |  |  | MTHFD1 | P11586 | 7.5 |  |  |  |
|  |  |  | FAH | P16930 | 7.37 |  |  |  |
|  |  |  | LPP | Q93052 | 7.37 |  |  |  |
|  |  |  | JUP | P14923 | 7.36 |  |  |  |
|  |  |  | GRM7 | Q14831 | 7.35 |  |  |  |
|  |  |  | ANXA11 | P50995 | 7.33 |  |  |  |
|  |  |  | SLC26A1 | Q9H2B4 | 7.31 |  |  |  |
|  |  |  | CCL19 | Q99731 | 7.31 |  |  |  |
|  |  |  | FOXP2 | O15409 | 7.3 |  |  |  |
|  |  |  | CHKA | P35790 | 7.29 |  |  |  |
|  |  |  | RBMX | P38159 | 7.24 |  |  |  |
|  |  |  | CACNA1H | O95180 | 7.23 |  |  |  |
|  |  |  | INA | Q16352 | 7.22 |  |  |  |
|  |  |  | SUOX | P51687 | 7.22 |  |  |  |
|  |  |  | PIK3C3 | Q8NEB9 | 7.21 |  |  |  |
|  |  |  | GABBR2 | O75899 | 7.19 |  |  |  |
|  |  |  | RAPSN | Q13702 | 7.19 |  |  |  |
|  |  |  | GRIK2 | Q13002 | 7.16 |  |  |  |
|  |  |  | GAD2 | Q05329 | 7.12 |  |  |  |
|  |  |  | HMBS | P08397 | 7.12 |  |  |  |
|  |  |  | TFAM | Q00059 | 7.1 |  |  |  |
|  |  |  | CCND3 | P30281 | 7.1 |  |  |  |
|  |  |  | SMAD6 | O43541 | 7.08 |  |  |  |
|  |  |  | RPLP2 | P05387 | 7.07 |  |  |  |
|  |  |  | DEFB1 | P60022 | 7.07 |  |  |  |
|  |  |  | KCNJ3 | P48549 | 7.07 |  |  |  |
|  |  |  | PPP1R1B | Q9UD71 | 7.07 |  |  |  |
|  |  |  | FKBP5 | Q13451 | 7.05 |  |  |  |
|  |  |  | RPS24 | P62847 | 7.04 |  |  |  |
|  |  |  | NLGN4X | Q8N0W4 | 7.02 |  |  |  |
|  |  |  | TFR2 | Q9UP52 | 7.02 |  |  |  |
|  |  |  | PIM1 | P11309 | 7.01 |  |  |  |
|  |  |  | CHRM2 | P08172 | 7.01 |  |  |  |
