# Supplementary material for: ShenQi DiHuang Decoction (SQDHD) Ameliorates Neuroinflammation and Neuropsychiatric Manifestations in Pristane Induced Lupus Mice via Blocking JAK1‐STAT3 Pathway
Source: CNS Neurosci Ther. 2026 Mar 7;32(3):e70814. doi: 10.1002/cns.70814 (PMC12967629; doi:10.1002/cns.70814)
Supplement: Supplementary file 4 — Table S4: Collection of 61 active compounds associated targets. [file CNS-32-e70814-s003.docx]

**TableS4: Collection of 61 active compounds associated targets**

|  | **Swiss Target Prediction** |  |  |
| --- | --- | --- | --- |
| **Compounds** | **UniProt** | **Target genes** | **Probability** |
| 16-oxo-11-anhydroalisol A | O00748 | CES2 | 0.56 |
| 16-oxo-11-anhydroalisol A | P11511 | CYP19A1 | 0.14 |
| 16-oxo-11-anhydroalisol A | P10275 | AR | 0.18 |
| 16-oxo-11-anhydroalisol A | P04150 | NR3C1 | 0.22 |
| 3-(Acetyloxy)-16-hydroxy-24-methylenelanost-8-en-21-oic acid (Pachymic acid) | P04150 | NR3C1 | 0.12 |
| 3-(Acetyloxy)-16-hydroxy-24-methylenelanost-8-en-21-oic acid (Pachymic acid) | P08235 | NR3C2 | 0.12 |
| 3-(Acetyloxy)-16-hydroxy-24-methylenelanost-8-en-21-oic acid (Pachymic acid) | P17706 | PTPN2 | 0.12 |
| 3-(Acetyloxy)-16-hydroxy-24-methylenelanost-8-en-21-oic acid (Pachymic acid) | P28845 | HSD11B1 | 0.12 |
| 3-(Acetyloxy)-16-hydroxy-24-methylenelanost-8-en-21-oic acid (Pachymic acid) | P11511 | CYP19A1 | 0.12 |
| 3-(Acetyloxy)-16-hydroxy-24-methylenelanost-8-en-21-oic acid (Pachymic acid) | P04278 | SHBG | 0.12 |
| 3-(Acetyloxy)-16-hydroxy-24-methylenelanost-8-en-21-oic acid (Pachymic acid) | P52895 | AKR1C2 | 0.12 |
| 3-(Acetyloxy)-16-hydroxy-24-methylenelanost-8-en-21-oic acid (Pachymic acid) | Q04828 | AKR1C1 | 0.12 |
| 3-(Acetyloxy)-16-hydroxy-24-methylenelanost-8-en-21-oic acid (Pachymic acid) | P49354 P49356 | FNTA FNTB | 0.12 |
| 3-(Acetyloxy)-16-hydroxy-24-methylenelanost-8-en-21-oic acid (Pachymic acid) | Q99814 | EPAS1 | 0.12 |
| 3-(Acetyloxy)-16-hydroxy-24-methylenelanost-8-en-21-oic acid (Pachymic acid) | O00748 | CES2 | 0.12 |
| 3-(Acetyloxy)-16-hydroxy-24-methylenelanost-8-en-21-oic acid (Pachymic acid) | P35228 | NOS2 | 0.12 |
| 3-(Acetyloxy)-16-hydroxy-24-methylenelanost-8-en-21-oic acid (Pachymic acid) | P00734 | F2 | 0.12 |
| 3-(Acetyloxy)-16-hydroxy-24-methylenelanost-8-en-21-oic acid (Pachymic acid) | P08185 | SERPINA6 | 0.12 |
| 3-(Acetyloxy)-16-hydroxy-24-methylenelanost-8-en-21-oic acid (Pachymic acid) | P04035 | HMGCR | 0.12 |
| 3-(Acetyloxy)-16-hydroxy-24-methylenelanost-8-en-21-oic acid (Pachymic acid) | P80365 | HSD11B2 | 0.12 |
| 3-(Acetyloxy)-16-hydroxy-24-methylenelanost-8-en-21-oic acid (Pachymic acid) | P06401 | PGR | 0.12 |
| 3-(Acetyloxy)-16-hydroxy-24-methylenelanost-8-en-21-oic acid (Pachymic acid) | Q96P20 | NLRP3 | 0.12 |
| 3-(Acetyloxy)-16-hydroxy-24-methylenelanost-8-en-21-oic acid (Pachymic acid) | Q99895 | CTRC | 0.12 |
| 3-(Acetyloxy)-16-hydroxy-24-methylenelanost-8-en-21-oic acid (Pachymic acid) | P07477 | PRSS1 | 0.12 |
| 3-(Acetyloxy)-16-hydroxy-24-methylenelanost-8-en-21-oic acid (Pachymic acid) | P37059 | HSD17B2 | 0.12 |
| 3-(Acetyloxy)-16-hydroxy-24-methylenelanost-8-en-21-oic acid (Pachymic acid) | P42330 | AKR1C3 | 0.12 |
| 3-(Acetyloxy)-16-hydroxy-24-methylenelanost-8-en-21-oic acid (Pachymic acid) | P48147 | PREP | 0.12 |
| 3-(Acetyloxy)-16-hydroxy-24-methylenelanost-8-en-21-oic acid (Pachymic acid) | P35354 | PTGS2 | 0.12 |
| 3-(Acetyloxy)-16-hydroxy-24-methylenelanost-8-en-21-oic acid (Pachymic acid) | Q07817 | BCL2L1 | 0.12 |
| 3-(Acetyloxy)-16-hydroxy-24-methylenelanost-8-en-21-oic acid (Pachymic acid) | P21731 | TBXA2R | 0.12 |
| 3-(Acetyloxy)-16-hydroxy-24-methylenelanost-8-en-21-oic acid (Pachymic acid) | O14920 | IKBKB | 0.12 |
| 3-(Acetyloxy)-16-hydroxy-24-methylenelanost-8-en-21-oic acid (Pachymic acid) | P33261 | CYP2C19 | 0.12 |
| 3-(Acetyloxy)-16-hydroxy-24-methylenelanost-8-en-21-oic acid (Pachymic acid) | O00519 | FAAH | 0.12 |
| 3-(Acetyloxy)-16-hydroxy-24-methylenelanost-8-en-21-oic acid (Pachymic acid) | P14555 | PLA2G2A | 0.12 |
| 3-(Acetyloxy)-16-hydroxy-24-methylenelanost-8-en-21-oic acid (Pachymic acid) | P08183 | ABCB1 | 0.12 |
| 3-(Acetyloxy)-16-hydroxy-24-methylenelanost-8-en-21-oic acid (Pachymic acid) | P20292 | ALOX5AP | 0.12 |
| 3-(Acetyloxy)-16-hydroxy-24-methylenelanost-8-en-21-oic acid (Pachymic acid) | Q13133 | NR1H3 | 0.12 |
| 3-(Acetyloxy)-16-hydroxy-24-methylenelanost-8-en-21-oic acid (Pachymic acid) | P24530 | EDNRB | 0.12 |
| 3-(Acetyloxy)-16-hydroxy-24-methylenelanost-8-en-21-oic acid (Pachymic acid) | Q9Y271 | CYSLTR1 | 0.12 |
| 3-(Acetyloxy)-16-hydroxy-24-methylenelanost-8-en-21-oic acid (Pachymic acid) | O14746 | TERT | 0.12 |
| 3-(Acetyloxy)-16-hydroxy-24-methylenelanost-8-en-21-oic acid (Pachymic acid) | P09917 | ALOX5 | 0.12 |
| 3-(Acetyloxy)-16-hydroxy-24-methylenelanost-8-en-21-oic acid (Pachymic acid) | O95136 | S1PR2 | 0.12 |
| 3-(Acetyloxy)-16-hydroxy-24-methylenelanost-8-en-21-oic acid (Pachymic acid) | Q9Y5Y4 | PTGDR2 | 0.12 |
| 3-(Acetyloxy)-16-hydroxy-24-methylenelanost-8-en-21-oic acid (Pachymic acid) | P11473 | VDR | 0.12 |
| 3-(Acetyloxy)-16-hydroxy-24-methylenelanost-8-en-21-oic acid (Pachymic acid) | P18031 | PTPN1 | 0.13 |
| 3-(Acetyloxy)-16-hydroxy-24-methylenelanost-8-en-21-oic acid (Pachymic acid) | P10275 | AR | 0.13 |
| 3-(Acetyloxy)-16-hydroxy-24-methylenelanost-8-en-21-oic acid (Pachymic acid) | P05093 | CYP17A1 | 0.13 |
| 3-(Acetyloxy)-16-hydroxy-24-methylenelanost-8-en-21-oic acid (Pachymic acid) | O14684 | PTGES | 0.2 |
| 3,23-Dihydroxyolean-12-en-28-oic acid (Hederagenin)* | O14746 | TERT | 0.12 |
| 3,23-Dihydroxyolean-12-en-28-oic acid (Hederagenin)* | O00767 | SCD | 0.12 |
| 3,23-Dihydroxyolean-12-en-28-oic acid (Hederagenin)* | P48147 | PREP | 0.12 |
| 3,23-Dihydroxyolean-12-en-28-oic acid (Hederagenin)* | P37231 | PPARG | 0.12 |
| 3,23-Dihydroxyolean-12-en-28-oic acid (Hederagenin)* | Q15722 | LTB4R | 0.12 |
| 3,23-Dihydroxyolean-12-en-28-oic acid (Hederagenin)* | P30304 | CDC25A | 0.12 |
| 3,23-Dihydroxyolean-12-en-28-oic acid (Hederagenin)* | P49354 P49356 | FNTA FNTB | 0.12 |
| 3,23-Dihydroxyolean-12-en-28-oic acid (Hederagenin)* | P11511 | CYP19A1 | 0.12 |
| 3,23-Dihydroxyolean-12-en-28-oic acid (Hederagenin)* | P09917 | ALOX5 | 0.12 |
| 3,23-Dihydroxyolean-12-en-28-oic acid (Hederagenin)* | P35354 | PTGS2 | 0.12 |
| 3,23-Dihydroxyolean-12-en-28-oic acid (Hederagenin)* | O00519 | FAAH | 0.12 |
| 3,23-Dihydroxyolean-12-en-28-oic acid (Hederagenin)* | Q07869 | PPARA | 0.12 |
| 3,23-Dihydroxyolean-12-en-28-oic acid (Hederagenin)* | Q03181 | PPARD | 0.12 |
| 3,23-Dihydroxyolean-12-en-28-oic acid (Hederagenin)* | P08235 | NR3C2 | 0.12 |
| 3,23-Dihydroxyolean-12-en-28-oic acid (Hederagenin)* | P10275 | AR | 0.12 |
| 3,23-Dihydroxyolean-12-en-28-oic acid (Hederagenin)* | P80365 | HSD11B2 | 0.12 |
| 3,23-Dihydroxyolean-12-en-28-oic acid (Hederagenin)* | P08185 | SERPINA6 | 0.12 |
| 3,23-Dihydroxyolean-12-en-28-oic acid (Hederagenin)* | P04278 | SHBG | 0.12 |
| 3,23-Dihydroxyolean-12-en-28-oic acid (Hederagenin)* | P11413 | G6PD | 0.12 |
| 3,23-Dihydroxyolean-12-en-28-oic acid (Hederagenin)* | Q16850 | CYP51A1 | 0.12 |
| 3,23-Dihydroxyolean-12-en-28-oic acid (Hederagenin)* | P15090 | FABP4 | 0.12 |
| 3,23-Dihydroxyolean-12-en-28-oic acid (Hederagenin)* | P05413 | FABP3 | 0.12 |
| 3,23-Dihydroxyolean-12-en-28-oic acid (Hederagenin)* | Q01469 | FABP5 | 0.12 |
| 3,23-Dihydroxyolean-12-en-28-oic acid (Hederagenin)* | P23219 | PTGS1 | 0.12 |
| 3,23-Dihydroxyolean-12-en-28-oic acid (Hederagenin)* | Q92731 | ESR2 | 0.12 |
| 3,23-Dihydroxyolean-12-en-28-oic acid (Hederagenin)* | Q06124 | PTPN11 | 0.12 |
| 3,23-Dihydroxyolean-12-en-28-oic acid (Hederagenin)* | Q9UHC9 | NPC1L1 | 0.12 |
| 3,23-Dihydroxyolean-12-en-28-oic acid (Hederagenin)* | Q99720 | SIGMAR1 | 0.12 |
| 3,23-Dihydroxyolean-12-en-28-oic acid (Hederagenin)* | P05093 | CYP17A1 | 0.12 |
| 3,23-Dihydroxyolean-12-en-28-oic acid (Hederagenin)* | P04150 | NR3C1 | 0.12 |
| 3,23-Dihydroxyolean-12-en-28-oic acid (Hederagenin)* | P04035 | HMGCR | 0.12 |
| 3,23-Dihydroxyolean-12-en-28-oic acid (Hederagenin)* | Q01959 | SLC6A3 | 0.12 |
| 3,23-Dihydroxyolean-12-en-28-oic acid (Hederagenin)* | P0DMS8 | ADORA3 | 0.12 |
| 3,23-Dihydroxyolean-12-en-28-oic acid (Hederagenin)* | P43116 | PTGER2 | 0.12 |
| 3,23-Dihydroxyolean-12-en-28-oic acid (Hederagenin)* | P06401 | PGR | 0.12 |
| 3,23-Dihydroxyolean-12-en-28-oic acid (Hederagenin)* | P31645 | SLC6A4 | 0.12 |
| 3,23-Dihydroxyolean-12-en-28-oic acid (Hederagenin)* | P11387 | TOP1 | 0.12 |
| 3,23-Dihydroxyolean-12-en-28-oic acid (Hederagenin)* | P24723 | PRKCH | 0.12 |
| 3,23-Dihydroxyolean-12-en-28-oic acid (Hederagenin)* | Q13133 | NR1H3 | 0.12 |
| 3,23-Dihydroxyolean-12-en-28-oic acid (Hederagenin)* | P35408 | PTGER4 | 0.12 |
| 3,23-Dihydroxyolean-12-en-28-oic acid (Hederagenin)* | Q01433 | AMPD2 | 0.12 |
| 3,23-Dihydroxyolean-12-en-28-oic acid (Hederagenin)* | P39086 | GRIK1 | 0.12 |
| 3,23-Dihydroxyolean-12-en-28-oic acid (Hederagenin)* | P08172 | CHRM2 | 0.12 |
| 3,23-Dihydroxyolean-12-en-28-oic acid (Hederagenin)* | P23975 | SLC6A2 | 0.12 |
| 3,23-Dihydroxyolean-12-en-28-oic acid (Hederagenin)* | Q13002 | GRIK2 | 0.12 |
| 3,23-Dihydroxyolean-12-en-28-oic acid (Hederagenin)* | Q8TDU6 | GPBAR1 | 0.12 |
| 3,23-Dihydroxyolean-12-en-28-oic acid (Hederagenin)* | Q9Y5Y4 | PTGDR2 | 0.12 |
| 3,23-Dihydroxyolean-12-en-28-oic acid (Hederagenin)* | Q96RI1 | NR1H4 | 0.12 |
| 3,23-Dihydroxyolean-12-en-28-oic acid (Hederagenin)* | P43119 | PTGIR | 0.12 |
| 3,23-Dihydroxyolean-12-en-28-oic acid (Hederagenin)* | P05231 | IL6 | 0.12 |
| 3,23-Dihydroxyolean-12-en-28-oic acid (Hederagenin)* | P15104 | GLUL | 0.12 |
| 3,23-Dihydroxyolean-12-en-28-oic acid (Hederagenin)* | P30556 | AGTR1 | 0.12 |
| 3,23-Dihydroxyolean-12-en-28-oic acid (Hederagenin)* | P35398 | RORA | 0.12 |
| 3,23-Dihydroxyolean-12-en-28-oic acid (Hederagenin)* | P20292 | ALOX5AP | 0.12 |
| 3,23-Dihydroxyolean-12-en-28-oic acid (Hederagenin)* | P06276 | BCHE | 0.12 |
| 3,23-Dihydroxyolean-12-en-28-oic acid (Hederagenin)* | O00748 | CES2 | 0.13 |
| 3,23-Dihydroxyolean-12-en-28-oic acid (Hederagenin)* | P07148 | FABP1 | 0.13 |
| 3,23-Dihydroxyolean-12-en-28-oic acid (Hederagenin)* | P35228 | NOS2 | 0.14 |
| 3,23-Dihydroxyolean-12-en-28-oic acid (Hederagenin)* | P29350 | PTPN6 | 0.15 |
| 3,23-Dihydroxyolean-12-en-28-oic acid (Hederagenin)* | O14684 | PTGES | 0.21 |
| 3,23-Dihydroxyolean-12-en-28-oic acid (Hederagenin)* | Q08499 | PDE4D | 0.36 |
| 3,23-Dihydroxyolean-12-en-28-oic acid (Hederagenin)* | P04054 | PLA2G1B | 0.39 |
| 3,23-Dihydroxyolean-12-en-28-oic acid (Hederagenin)* | P30305 | CDC25B | 0.39 |
| 3,23-Dihydroxyolean-12-en-28-oic acid (Hederagenin)* | P51449 | RORC | 0.4 |
| 3,23-Dihydroxyolean-12-en-28-oic acid (Hederagenin)* | P10586 | PTPRF | 0.4 |
| 3,23-Dihydroxyolean-12-en-28-oic acid (Hederagenin)* | P17706 | PTPN2 | 0.4 |
| 3,23-Dihydroxyolean-12-en-28-oic acid (Hederagenin)* | P24666 | ACP1 | 0.4 |
| 3,23-Dihydroxyolean-12-en-28-oic acid (Hederagenin)* | P28845 | HSD11B1 | 0.41 |
| 3,23-Dihydroxyolean-12-en-28-oic acid (Hederagenin)* | O60218 | AKR1B10 | 0.41 |
| 3,23-Dihydroxyolean-12-en-28-oic acid (Hederagenin)* | P06746 | POLB | 0.46 |
| 3,23-Dihydroxyolean-12-en-28-oic acid (Hederagenin)* | P18031 | PTPN1 | 0.66 |
| 3,23-Dihydroxyolean-12-en-28-oic acid (Hederagenin)* | P60033 | CD81 | 0.67 |
| 3,4-Dihydroxybenzoic acid (Protocatechuic acid)* | P00918 | CA2 | 0.93 |
| 3,4-Dihydroxybenzoic acid (Protocatechuic acid)* | P43166 | CA7 | 0.93 |
| 3,4-Dihydroxybenzoic acid (Protocatechuic acid)* | P00915 | CA1 | 0.93 |
| 3,4-Dihydroxybenzoic acid (Protocatechuic acid)* | P23280 | CA6 | 0.93 |
| 3,4-Dihydroxybenzoic acid (Protocatechuic acid)* | O43570 | CA12 | 0.93 |
| 3,4-Dihydroxybenzoic acid (Protocatechuic acid)* | Q9ULX7 | CA14 | 0.93 |
| 3,4-Dihydroxybenzoic acid (Protocatechuic acid)* | Q16790 | CA9 | 0.93 |
| 3,4-Dihydroxybenzoic acid (Protocatechuic acid)* | P22748 | CA4 | 0.93 |
| 3-Hydroxybenzoic acid | P07451 | CA3 | 0.32 |
| 3-Hydroxybenzoic acid | Q9Y2D0 | CA5B | 0.32 |
| 3-Hydroxybenzoic acid | P35218 | CA5A | 0.32 |
| 3-Hydroxybenzoic acid | Q8N1Q1 | CA13 | 0.32 |
| 3-Hydroxybenzoic acid | P22748 | CA4 | 0.39 |
| 3-Hydroxybenzoic acid | P43166 | CA7 | 0.57 |
| 3-Hydroxybenzoic acid | Q9ULX7 | CA14 | 0.57 |
| 3-Hydroxybenzoic acid | P00918 | CA2 | 1 |
| 3-Hydroxybenzoic acid | P00915 | CA1 | 1 |
| 3-Hydroxybenzoic acid | P23280 | CA6 | 1 |
| 3-Hydroxybenzoic acid | O43570 | CA12 | 1 |
| 3-Hydroxybenzoic acid | Q16790 | CA9 | 1 |
| 3-O-Acetyl-16-hydroxytrametenolic acid | P18031 | PTPN1 | 0.13 |
| 3-O-Acetyl-16-hydroxytrametenolic acid | P28845 | HSD11B1 | 0.13 |
| 3-O-Acetyl-16-hydroxytrametenolic acid | P08235 | NR3C2 | 0.13 |
| 3-O-Acetyl-16-hydroxytrametenolic acid | P11511 | CYP19A1 | 0.13 |
| 3-O-Acetyl-16-hydroxytrametenolic acid | P49354 P49356 | FNTA FNTB | 0.13 |
| 3-O-Acetyl-16-hydroxytrametenolic acid | P04278 | SHBG | 0.13 |
| 3-O-Acetyl-16-hydroxytrametenolic acid | P33261 | CYP2C19 | 0.13 |
| 3-O-Acetyl-16-hydroxytrametenolic acid | P52895 | AKR1C2 | 0.13 |
| 3-O-Acetyl-16-hydroxytrametenolic acid | Q04828 | AKR1C1 | 0.13 |
| 3-O-Acetyl-16-hydroxytrametenolic acid | Q99814 | EPAS1 | 0.13 |
| 3-O-Acetyl-16-hydroxytrametenolic acid | P14555 | PLA2G2A | 0.13 |
| 3-O-Acetyl-16-hydroxytrametenolic acid | P08185 | SERPINA6 | 0.13 |
| 3-O-Acetyl-16-hydroxytrametenolic acid | P17706 | PTPN2 | 0.13 |
| 3-O-Acetyl-16-hydroxytrametenolic acid | P00734 | F2 | 0.13 |
| 3-O-Acetyl-16-hydroxytrametenolic acid | P80365 | HSD11B2 | 0.13 |
| 3-O-Acetyl-16-hydroxytrametenolic acid | P04035 | HMGCR | 0.13 |
| 3-O-Acetyl-16-hydroxytrametenolic acid | Q99895 | CTRC | 0.13 |
| 3-O-Acetyl-16-hydroxytrametenolic acid | P04150 | NR3C1 | 0.13 |
| 3-O-Acetyl-16-hydroxytrametenolic acid | Q96P20 | NLRP3 | 0.13 |
| 3-O-Acetyl-16-hydroxytrametenolic acid | Q13133 | NR1H3 | 0.13 |
| 3-O-Acetyl-16-hydroxytrametenolic acid | P35228 | NOS2 | 0.13 |
| 3-O-Acetyl-16-hydroxytrametenolic acid | P07477 | PRSS1 | 0.13 |
| 3-O-Acetyl-16-hydroxytrametenolic acid | P06401 | PGR | 0.13 |
| 3-O-Acetyl-16-hydroxytrametenolic acid | P48147 | PREP | 0.13 |
| 3-O-Acetyl-16-hydroxytrametenolic acid | P35354 | PTGS2 | 0.13 |
| 3-O-Acetyl-16-hydroxytrametenolic acid | P42330 | AKR1C3 | 0.13 |
| 3-O-Acetyl-16-hydroxytrametenolic acid | P08183 | ABCB1 | 0.13 |
| 3-O-Acetyl-16-hydroxytrametenolic acid | O00519 | FAAH | 0.13 |
| 3-O-Acetyl-16-hydroxytrametenolic acid | O14920 | IKBKB | 0.13 |
| 3-O-Acetyl-16-hydroxytrametenolic acid | Q9Y271 | CYSLTR1 | 0.13 |
| 3-O-Acetyl-16-hydroxytrametenolic acid | O14746 | TERT | 0.13 |
| 3-O-Acetyl-16-hydroxytrametenolic acid | P21731 | TBXA2R | 0.13 |
| 3-O-Acetyl-16-hydroxytrametenolic acid | P09917 | ALOX5 | 0.13 |
| 3-O-Acetyl-16-hydroxytrametenolic acid | P49841 | GSK3B | 0.13 |
| 3-O-Acetyl-16-hydroxytrametenolic acid | P37059 | HSD17B2 | 0.13 |
| 3-O-Acetyl-16-hydroxytrametenolic acid | P10275 | AR | 0.13 |
| 3-O-Acetyl-16-hydroxytrametenolic acid | P05093 | CYP17A1 | 0.13 |
| 3-O-Acetyl-16-hydroxytrametenolic acid | O00748 | CES2 | 0.16 |
| 3-O-Acetyl-16-hydroxytrametenolic acid | O14684 | PTGES | 0.27 |
| 4-Hydroxybenzoic acid | P00918 | CA2 | 1 |
| 4-Hydroxybenzoic acid | P43166 | CA7 | 1 |
| 4-Hydroxybenzoic acid | P00915 | CA1 | 1 |
| 4-Hydroxybenzoic acid | P07451 | CA3 | 1 |
| 4-Hydroxybenzoic acid | P23280 | CA6 | 1 |
| 4-Hydroxybenzoic acid | O43570 | CA12 | 1 |
| 4-Hydroxybenzoic acid | Q9ULX7 | CA14 | 1 |
| 4-Hydroxybenzoic acid | Q16790 | CA9 | 1 |
| 4-Hydroxybenzoic acid | P22748 | CA4 | 1 |
| 4-Hydroxybenzoic acid | Q9Y2D0 | CA5B | 1 |
| 4-Hydroxybenzoic acid | P35218 | CA5A | 1 |
| 4-Hydroxybenzoic acid | Q8N1Q1 | CA13 | 1 |
| 5,4'-Dihydroxy-3,7-dimethoxyflavone(Kumatakenin)* | Q96S37 | SLC22A12 | 0.13 |
| 5,4'-Dihydroxy-3,7-dimethoxyflavone(Kumatakenin)* | P41143 | OPRD1 | 0.13 |
| 5,4'-Dihydroxy-3,7-dimethoxyflavone(Kumatakenin)* | Q92731 | ESR2 | 0.13 |
| 5,4'-Dihydroxy-3,7-dimethoxyflavone(Kumatakenin)* | Q9UNQ0 | ABCG2 | 0.14 |
| 5,4'-Dihydroxy-3,7-dimethoxyflavone(Kumatakenin)* | P08183 | ABCB1 | 0.18 |
| 5,4'-Dihydroxy-3,7-dimethoxyflavone(Kumatakenin)* | Q07820 | MCL1 | 0.18 |
| 5,4'-Dihydroxy-3,7-dimethoxyflavone(Kumatakenin)* | Q9NPH5 | NOX4 | 0.2 |
| 5,4'-Dihydroxy-3,7-dimethoxyflavone(Kumatakenin)* | P29274 | ADORA2A | 0.32 |
| 5,4'-Dihydroxy-3,7-dimethoxyflavone(Kumatakenin)* | P30542 | ADORA1 | 0.42 |
| 5,4'-Dihydroxy-3,7-dimethoxyflavone(Kumatakenin)* | P15121 | AKR1B1 | 0.44 |
| 8-Debenzoylpaeoniflorin | P15692 | VEGFA | 0.13 |
| 8-Debenzoylpaeoniflorin | P05230 | FGF1 | 0.13 |
| 8-Debenzoylpaeoniflorin | P09038 | FGF2 | 0.13 |
| 8-Debenzoylpaeoniflorin | Q9Y251 | HPSE | 0.13 |
| Alisol B Acetate | Q04759 | PRKCQ | 0.13 |
| Alisol B Acetate | Q05655 | PRKCD | 0.13 |
| Alisol B Acetate | P04035 | HMGCR | 0.13 |
| Alisol B Acetate | P35228 | NOS2 | 0.13 |
| Alisol B Acetate | P28845 | HSD11B1 | 0.13 |
| Alisol B Acetate | P35354 | PTGS2 | 0.13 |
| Alisol B Acetate | P49354 P49356 | FNTA FNTB | 0.13 |
| Alisol B Acetate | P53609 P49354 | PGGT1B FNTA | 0.13 |
| Alisol B Acetate | P18031 | PTPN1 | 0.13 |
| Alisol B Acetate | P50579 | METAP2 | 0.13 |
| Alisol B Acetate | P05771 | PRKCB | 0.13 |
| Alisol B Acetate | P05129 | PRKCG | 0.13 |
| Alisol B Acetate | Q02156 | PRKCE | 0.13 |
| Alisol B Acetate | P24723 | PRKCH | 0.13 |
| Alisol B Acetate | P25103 | TACR1 | 0.13 |
| Alisol B Acetate | Q9HBA0 | TRPV4 | 0.13 |
| Alisol B Acetate | P56373 | P2RX3 | 0.13 |
| Alisol B Acetate | P52333 | JAK3 | 0.13 |
| Alisol B Acetate | P21730 | C5AR1 | 0.13 |
| Alisol B Acetate | P21554 | CNR1 | 0.13 |
| Alisol B Acetate | P11511 | CYP19A1 | 0.13 |
| Alisol B Acetate | O15111 | CHUK | 0.13 |
| Alisol B Acetate | P21917 | DRD4 | 0.13 |
| Alisol B Acetate | P35462 | DRD3 | 0.13 |
| Alisol B Acetate | P34972 | CNR2 | 0.13 |
| Alisol B Acetate | P37288 | AVPR1A | 0.13 |
| Alisol B Acetate | Q08499 | PDE4D | 0.13 |
| Alisol B Acetate | P32246 | CCR1 | 0.13 |
| Alisol B Acetate | P35968 | KDR | 0.13 |
| Alisol B Acetate | P25116 | F2R | 0.13 |
| Alisol B Acetate | Q00987 | MDM2 | 0.13 |
| Alisol B Acetate | Q9Y5Z0 | BACE2 | 0.13 |
| Alisol B Acetate | P07339 | CTSD | 0.13 |
| Alisol B Acetate | O15055 | PER2 | 0.13 |
| Alisol B Acetate | P37058 | HSD17B3 | 0.13 |
| Alisol B Acetate | Q99558 | MAP3K14 | 0.13 |
| Alisol B Acetate | Q9Y233 | PDE10A | 0.13 |
| Alisol B Acetate | Q01959 | SLC6A3 | 0.13 |
| Alisol B Acetate | P06241 | FYN | 0.13 |
| Alisol B Acetate | P00533 | EGFR | 0.13 |
| Alisol B Acetate | P16234 P09619 | PDGFRA PDGFRB | 0.13 |
| Alisol B Acetate | P08842 | STS | 0.13 |
| Alisol B Acetate | P28482 | MAPK1 | 0.13 |
| Alisol B Acetate | P22460 | KCNA5 | 0.13 |
| Alisol B Acetate | P54753 | EPHB3 | 0.13 |
| Alisol B Acetate | P33316 | DUT | 0.13 |
| Alisol B Acetate | P00734 | F2 | 0.13 |
| Alisol B Acetate | P05093 | CYP17A1 | 0.13 |
| Alisol B Acetate | P00797 | REN | 0.13 |
| Alisol B Acetate | P04150 | NR3C1 | 0.13 |
| Alisol B Acetate | O14920 | IKBKB | 0.13 |
| Alisol B Acetate | Q99835 | SMO | 0.13 |
| Alisol B Acetate | O00408 | PDE2A | 0.13 |
| Alisol B Acetate | Q16539 | MAPK14 | 0.13 |
| Alisol B Acetate | Q99705 | MCHR1 | 0.13 |
| Alisol B Acetate | O00142 | TK2 | 0.13 |
| Alisol B Acetate | P49336 | CDK8 | 0.13 |
| Alisol B Acetate | P32245 | MC4R | 0.13 |
| Alisol B Acetate | Q01726 | MC1R | 0.13 |
| Alisol B Acetate | P33032 | MC5R | 0.13 |
| Alisol B Acetate | P41968 | MC3R | 0.13 |
| Alisol B Acetate | P23458 | JAK1 | 0.13 |
| Alisol B Acetate | Q99500 | S1PR3 | 0.13 |
| Alisol B Acetate | O14684 | PTGES | 0.13 |
| Alisol B Acetate | O75469 | NR1I2 | 0.13 |
| Alisol B Acetate | P23975 | SLC6A2 | 0.13 |
| Alisol B Acetate | Q96GD4 | AURKB | 0.13 |
| Alisol B Acetate | O14649 | KCNK3 | 0.13 |
| Alisol B Acetate | Q9NWT8 | AURKAIP1 | 0.13 |
| Alisol B Acetate | P08235 | NR3C2 | 0.13 |
| Alisol B Acetate | P24530 | EDNRB | 0.13 |
| Alisol B Acetate | O75116 | ROCK2 | 0.13 |
| Alisol B Acetate | O14757 | CHEK1 | 0.13 |
| Alisol B Acetate | O14965 | AURKA | 0.13 |
| Alisol B Acetate | P0DJD9 | PGA5 | 0.13 |
| Alisol B Acetate | P01130 | LDLR | 0.13 |
| Alisol B Acetate | P03956 | MMP1 | 0.13 |
| Alisol B Acetate | P10275 | AR | 0.13 |
| Alisol B Acetate | P00740 | F9 | 0.13 |
| Alisol B Acetate | P00742 | F10 | 0.13 |
| Alisol B Acetate | P50750 | CDK9 | 0.13 |
| Alisol B Acetate | Q00975 | CACNA1B | 0.13 |
| Alisol B Acetate | O00519 | FAAH | 0.13 |
| Alisol B Acetate | P07333 | CSF1R | 0.13 |
| Alisol B Acetate | P08069 | IGF1R | 0.13 |
| Alisol B Acetate | Q96RI1 | NR1H4 | 0.13 |
| Alisol B Acetate | P21728 | DRD1 | 0.13 |
| Alisol B Acetate | P28223 | HTR2A | 0.13 |
| Alisol B Acetate | P28335 | HTR2C | 0.13 |
| Alisol B Acetate | Q9UNA0 | ADAMTS5 | 0.13 |
| Alisol B Acetate | O75173 | ADAMTS4 | 0.13 |
| Alisol B Acetate | P48067 | SLC6A9 | 0.13 |
| Alisol B Acetate | Q12908 | SLC10A2 | 0.13 |
| Alisol B Acetate | P45452 | MMP13 | 0.13 |
| Alisol B Acetate | P32239 | CCKBR | 0.13 |
| Alisol B Acetate | P08253 | MMP2 | 0.13 |
| Alisol B Acetate | P50281 | MMP14 | 0.13 |
| Alisol B Acetate | O15294 | OGT | 0.13 |
| Alisol B Acetate | P17252 | PRKCA | 0.13 |
| Alisol B Acetate | O00748 | CES2 | 0.14 |
| alisol C,23-acetate* | Q05655 | PRKCD | 0.12 |
| alisol C,23-acetate* | P05771 | PRKCB | 0.12 |
| alisol C,23-acetate* | O00748 | CES2 | 0.12 |
| alisol C,23-acetate* | P17252 | PRKCA | 0.12 |
| alisol C,23-acetate* | P05129 | PRKCG | 0.12 |
| alisol C,23-acetate* | Q02156 | PRKCE | 0.12 |
| alisol C,23-acetate* | P24723 | PRKCH | 0.12 |
| alisol C,23-acetate* | Q04759 | PRKCQ | 0.12 |
| alisol C,23-acetate* | P35228 | NOS2 | 0.12 |
| alisol C,23-acetate* | P04035 | HMGCR | 0.12 |
| alisol C,23-acetate* | P28845 | HSD11B1 | 0.12 |
| alisol C,23-acetate* | Q9HBA0 | TRPV4 | 0.12 |
| alisol C,23-acetate* | P04150 | NR3C1 | 0.12 |
| alisol C,23-acetate* | P10275 | AR | 0.12 |
| alisol C,23-acetate* | P15498 | VAV1 | 0.12 |
| alisol C,23-acetate* | P35354 | PTGS2 | 0.12 |
| alisol C,23-acetate* | P49354 P49356 | FNTA FNTB | 0.12 |
| alisol C,23-acetate* | P22001 | KCNA3 | 0.12 |
| alisol C,23-acetate* | P08235 | NR3C2 | 0.12 |
| alisol C,23-acetate* | P08185 | SERPINA6 | 0.12 |
| alisol C,23-acetate* | Q08499 | PDE4D | 0.12 |
| alisol C,23-acetate* | P56373 | P2RX3 | 0.12 |
| alisol C,23-acetate* | P32246 | CCR1 | 0.12 |
| alisol C,23-acetate* | Q00987 | MDM2 | 0.12 |
| alisol C,23-acetate* | Q99814 | EPAS1 | 0.12 |
| alisol C,23-acetate* | P09917 | ALOX5 | 0.12 |
| alisol C,23-acetate* | P42338 | PIK3CB | 0.12 |
| alisol C,23-acetate* | P55085 | F2RL1 | 0.12 |
| alisol C,23-acetate* | P33316 | DUT | 0.12 |
| alisol C,23-acetate* | P48147 | PREP | 0.12 |
| alisol C,23-acetate* | Q9BY41 | HDAC8 | 0.12 |
| alisol C,23-acetate* | O14920 | IKBKB | 0.12 |
| alisol C,23-acetate* | P08254 | MMP3 | 0.12 |
| alisol C,23-acetate* | Q02750 | MAP2K1 | 0.12 |
| alisol C,23-acetate* | P09237 | MMP7 | 0.12 |
| alisol C,23-acetate* | P39900 | MMP12 | 0.12 |
| alisol C,23-acetate* | P24530 | EDNRB | 0.12 |
| alisol C,23-acetate* | Q99558 | MAP3K14 | 0.12 |
| alisol C,23-acetate* | P25116 | F2R | 0.12 |
| alisol C,23-acetate* | P24863 P49336 | CCNC CDK8 | 0.12 |
| alisol C,23-acetate* | O15111 | CHUK | 0.12 |
| alisol C,23-acetate* | P49336 | CDK8 | 0.12 |
| alisol C,23-acetate* | P08183 | ABCB1 | 0.12 |
| alisol C,23-acetate* | P52333 | JAK3 | 0.12 |
| alisol C,23-acetate* | P35968 | KDR | 0.12 |
| alisol C,23-acetate* | O75469 | NR1I2 | 0.12 |
| alisol C,23-acetate* | P37288 | AVPR1A | 0.12 |
| alisol C,23-acetate* | Q96RI1 | NR1H4 | 0.12 |
| alisol C,23-acetate* | O15055 | PER2 | 0.12 |
| alisol C,23-acetate* | Q99835 | SMO | 0.12 |
| alisol C,23-acetate* | Q9UNA0 | ADAMTS5 | 0.12 |
| alisol C,23-acetate* | O75173 | ADAMTS4 | 0.12 |
| alisol C,23-acetate* | P35462 | DRD3 | 0.12 |
| alisol C,23-acetate* | P28482 | MAPK1 | 0.12 |
| alisol C,23-acetate* | P53609 P49354 | PGGT1B FNTA | 0.12 |
| alisol C,23-acetate* | P25103 | TACR1 | 0.12 |
| alisol C,23-acetate* | O75116 | ROCK2 | 0.12 |
| alisol C,23-acetate* | O00329 | PIK3CD | 0.12 |
| alisol C,23-acetate* | O15294 | OGT | 0.12 |
| alisol C,23-acetate* | P18031 | PTPN1 | 0.12 |
| alisol C,23-acetate* | O14965 | AURKA | 0.12 |
| Apigenin-7-O-neohesperidoside (Rhoifolin)* | P47989 | XDH | 0.14 |
| Apigenin-7-O-neohesperidoside (Rhoifolin)* | P30542 | ADORA1 | 0.14 |
| Apigenin-7-O-neohesperidoside (Rhoifolin)* | P15121 | AKR1B1 | 0.18 |
| Apigenin-7-O-neohesperidoside (Rhoifolin)* | P60568 | IL2 | 0.2 |
| Apigenin-7-O-neohesperidoside (Rhoifolin)* | P01375 | TNF | 0.3 |
| Azelaic acid | Q4U2R8 | SLC22A6 | 0.14 |
| Azelaic acid | P10275 | AR | 0.15 |
| Azelaic acid | P11473 | VDR | 0.15 |
| Azelaic acid | Q96RI1 | NR1H4 | 0.15 |
| Azelaic acid | P06746 | POLB | 0.15 |
| Azelaic acid | P30304 | CDC25A | 0.15 |
| Azelaic acid | Q8TDU6 | GPBAR1 | 0.15 |
| Azelaic acid | P28845 | HSD11B1 | 0.2 |
| Azelaic acid | Q07869 | PPARA | 0.25 |
| Azelaic acid | P15090 | FABP4 | 0.29 |
| Azelaic acid | P05413 | FABP3 | 0.29 |
| Azelaic acid | Q01469 | FABP5 | 0.29 |
| Azelaic acid | Q03181 | PPARD | 0.29 |
| Azelaic acid | O14842 | FFAR1 | 0.29 |
| Azelaic acid | P12104 | FABP2 | 0.29 |
| Benzamide | P09874 | PARP1 | 1 |
| Benzoic acid | P00918 | CA2 | 0.17 |
| Benzoic acid | P00915 | CA1 | 0.17 |
| Benzoic acid | P14920 | DAO | 1 |
| Biochanin A-7-O-glucoside (Sissotrin)* | P01375 | TNF | 0.17 |
| Biochanin A-7-O-glucoside (Sissotrin)* | P05091 | ALDH2 | 0.26 |
| Biochanin A-7-O-glucoside (Sissotrin)* | P60568 | IL2 | 0.28 |
| Caffeic acid | P15121 | AKR1B1 | 0.15 |
| Caffeic acid | Q92731 | ESR2 | 0.15 |
| Caffeic acid | P22748 | CA4 | 0.15 |
| Caffeic acid | P07451 | CA3 | 0.21 |
| Caffeic acid | P00918 | CA2 | 0.73 |
| Caffeic acid | P09917 | ALOX5 | 0.73 |
| Caffeic acid | P43166 | CA7 | 0.73 |
| Caffeic acid | P00915 | CA1 | 0.73 |
| Caffeic acid | P23280 | CA6 | 0.73 |
| Caffeic acid | P14780 | MMP9 | 0.73 |
| Caffeic acid | O43570 | CA12 | 0.73 |
| Caffeic acid | P03956 | MMP1 | 0.73 |
| Caffeic acid | P08253 | MMP2 | 0.73 |
| Caffeic acid | P18031 | PTPN1 | 0.73 |
| Caffeic acid | Q9ULX7 | CA14 | 0.73 |
| Caffeic acid | Q16790 | CA9 | 0.73 |
| Caffeic acid | Q9Y2D0 | CA5B | 0.73 |
| Caffeic acid | P35218 | CA5A | 0.73 |
| Calycosin | P14061 | HSD17B1 | 0.12 |
| Calycosin | P03372 | ESR1 | 0.12 |
| Calycosin | Q92731 | ESR2 | 0.12 |
| Calycosin | Q07869 | PPARA | 0.13 |
| Calycosin | P14679 | TYR | 0.16 |
| Calycosin | P14174 | MIF | 0.16 |
| Calycosin | P08183 | ABCB1 | 0.18 |
| Calycosin | P11511 | CYP19A1 | 0.31 |
| Calycosin | P43166 | CA7 | 0.31 |
| Calycosin | P37059 | HSD17B2 | 0.31 |
| Calycosin | O43570 | CA12 | 0.31 |
| Calycosin | P22748 | CA4 | 0.31 |
| Calycosin | P16152 | CBR1 | 0.31 |
| Calycosin-7-O-glucoside | P05091 | ALDH2 | 0.15 |
| Calycosin-7-O-glucoside | P01375 | TNF | 1 |
| Calycosin-7-O-glucoside | P60568 | IL2 | 1 |
| Cornuside | P07900 | HSP90AA1 | 0.12 |
| Cornuside | P29274 | ADORA2A | 0.12 |
| Cornuside | P0DMS8 | ADORA3 | 0.12 |
| Cornuside | P13866 | SLC5A1 | 0.12 |
| Cornuside | P31639 | SLC5A2 | 0.12 |
| Cornuside | P45452 | MMP13 | 0.12 |
| Cornuside | P03956 | MMP1 | 0.12 |
| Cornuside | P09237 | MMP7 | 0.12 |
| Cornuside | P22894 | MMP8 | 0.12 |
| Cornuside | P00918 | CA2 | 0.12 |
| Cornuside | P00915 | CA1 | 0.12 |
| Cornuside | O43570 | CA12 | 0.12 |
| Cornuside | Q9ULX7 | CA14 | 0.12 |
| Cornuside | Q16790 | CA9 | 0.12 |
| Cornuside | P29275 | ADORA2B | 0.12 |
| Cornuside | Q99808 | SLC29A1 | 0.12 |
| Cornuside | P11387 | TOP1 | 0.12 |
| Cornuside | P11511 | CYP19A1 | 0.12 |
| Cornuside | P56470 | LGALS4 | 0.12 |
| Cornuside | O00214 | LGALS8 | 0.12 |
| Cornuside | P18405 | SRD5A1 | 0.12 |
| Cornuside | P20839 | IMPDH1 | 0.12 |
| Cornuside | P05091 | ALDH2 | 0.12 |
| Cornuside | Q8TDS4 | HCAR2 | 0.12 |
| Cornuside | O43868 | SLC28A2 | 0.12 |
| Cornuside | P01375 | TNF | 0.12 |
| Cornuside | P16455 | MGMT | 0.12 |
| Cornuside | P14679 | TYR | 0.12 |
| Cornuside | Q9HAS3 | SLC28A3 | 0.12 |
| D-Fructose* | P06493 | CDK1 | 0.13 |
| D-Fructose* | P56470 | LGALS4 | 0.13 |
| D-Fructose* | P17931 | LGALS3 | 0.13 |
| D-Fructose* | O00214 | LGALS8 | 0.13 |
| D-Fructose* | P07900 | HSP90AA1 | 0.13 |
| D-Fructose* | P15692 | VEGFA | 0.13 |
| D-Fructose* | P49810 Q9NZ42 Q92542 Q96BI3 P49768 Q8WW43 | PSEN2 PSENEN NCSTN APH1A PSEN1 APH1B | 0.13 |
| D-Fructose* | P05230 | FGF1 | 0.13 |
| D-Fructose* | P09038 | FGF2 | 0.13 |
| D-Fructose* | Q9Y251 | HPSE | 0.13 |
| D-Fructose* | P04062 | GBA1 | 0.14 |
| D-Galactose* | P06493 | CDK1 | 0.13 |
| D-Galactose* | P56470 | LGALS4 | 0.13 |
| D-Galactose* | P17931 | LGALS3 | 0.13 |
| D-Galactose* | O00214 | LGALS8 | 0.13 |
| D-Galactose* | P07900 | HSP90AA1 | 0.13 |
| D-Galactose* | P04062 | GBA | 0.13 |
| D-Galactose* | P15692 | VEGFA | 0.13 |
| D-Galactose* | P49810 Q9NZ42 Q92542 Q96BI3 P49768 Q8WW43 | PSEN2 PSENEN NCSTN APH1A PSEN1 APH1B | 0.13 |
| D-Galactose* | P05230 | FGF1 | 0.13 |
| D-Galactose* | P09038 | FGF2 | 0.13 |
| D-Galactose* | Q9Y251 | HPSE | 0.13 |
| D-Maltose* | P09038 | FGF2 | 0.13 |
| D-Maltose* | P06493 | CDK1 | 0.19 |
| D-Maltose* | P15692 | VEGFA | 0.22 |
| D-Maltose* | P05230 | FGF1 | 0.22 |
| D-Maltose* | Q9Y251 | HPSE | 0.22 |
| Ellagic acid | P00390 | GSR | 0.17 |
| Ellagic acid | Q9HC97 | GPR35 | 1 |
| Ellagic acid | P04626 | ERBB2 | 1 |
| Ellagic acid | P15121 | AKR1B1 | 1 |
| Ellagic acid | P24385 P11802 | CCND1 CDK4 | 1 |
| Ellagic acid | P09619 | PDGFRB | 1 |
| Ellagic acid | P35916 | FLT4 | 1 |
| Ellagic acid | P08069 | IGF1R | 1 |
| Ellagic acid | P06213 | INSR | 1 |
| Ellagic acid | P00533 | EGFR | 1 |
| Ellagic acid | P00918 | CA2 | 1 |
| Ellagic acid | P24941 P78396 P20248 | CDK2 CCNA1 CCNA2 | 1 |
| Ellagic acid | Q96GD4 | AURKB | 1 |
| Ellagic acid | P43166 | CA7 | 1 |
| Ellagic acid | P00915 | CA1 | 1 |
| Ellagic acid | P49841 | GSK3B | 1 |
| Ellagic acid | P12931 | SRC | 1 |
| Ellagic acid | Q05397 | PTK2 | 1 |
| Ellagic acid | P35968 | KDR | 1 |
| Ellagic acid | P53350 | PLK1 | 1 |
| Ellagic acid | P23280 | CA6 | 1 |
| Ellagic acid | O43570 | CA12 | 1 |
| Ellagic acid | Q9ULX7 | CA14 | 1 |
| Ellagic acid | Q16790 | CA9 | 1 |
| Ellagic acid | P68400 | CSNK2A1 | 1 |
| Ellagic acid | P08581 | MET | 1 |
| Ellagic acid | P22748 | CA4 | 1 |
| Ellagic acid | O00444 | PLK4 | 1 |
| Ellagic acid | Q8N1Q1 | CA13 | 1 |
| Ellagic acid | Q02763 | TEK | 1 |
| Ellagic acid | P31749 | AKT1 | 1 |
| Ellagic acid | O14965 | AURKA | 1 |
| Ellagic acid | P35218 | CA5A | 1 |
| Ellagic acid | P56817 | BACE1 | 1 |
| Ellagic acid | P41279 | MAP3K8 | 1 |
| Ellagic acid | P15056 | BRAF | 1 |
| Ellagic acid | P54760 | EPHB4 | 1 |
| Ellagic acid | P0DMV8 | HSPA1A | 1 |
| Ellagic acid | O60285 | NUAK1 | 1 |
| Ellagic acid | Q14534 | SQLE | 1 |
| Ellagic acid | P09769 | FGR | 1 |
| Ellagic acid | P07948 | LYN | 1 |
| Formononetin (7-Hydroxy-4'-methoxyisoflavone) | P00533 | EGFR | 0.13 |
| Formononetin (7-Hydroxy-4'-methoxyisoflavone) | P23975 | SLC6A2 | 0.13 |
| Formononetin (7-Hydroxy-4'-methoxyisoflavone) | P18054 | ALOX12 | 0.13 |
| Formononetin (7-Hydroxy-4'-methoxyisoflavone) | P43166 | CA7 | 0.14 |
| Formononetin (7-Hydroxy-4'-methoxyisoflavone) | P37059 | HSD17B2 | 0.14 |
| Formononetin (7-Hydroxy-4'-methoxyisoflavone) | P22748 | CA4 | 0.14 |
| Formononetin (7-Hydroxy-4'-methoxyisoflavone) | P16152 | CBR1 | 0.14 |
| Formononetin (7-Hydroxy-4'-methoxyisoflavone) | P24557 | TBXAS1 | 0.15 |
| Formononetin (7-Hydroxy-4'-methoxyisoflavone) | P21397 | MAOA | 0.15 |
| Formononetin (7-Hydroxy-4'-methoxyisoflavone) | O43451 | MGAM | 0.15 |
| Formononetin (7-Hydroxy-4'-methoxyisoflavone) | P28223 | HTR2A | 0.15 |
| Formononetin (7-Hydroxy-4'-methoxyisoflavone) | P28335 | HTR2C | 0.15 |
| Formononetin (7-Hydroxy-4'-methoxyisoflavone) | P11474 | ESRRA | 0.15 |
| Formononetin (7-Hydroxy-4'-methoxyisoflavone) | O95718 | ESRRB | 0.15 |
| Formononetin (7-Hydroxy-4'-methoxyisoflavone) | Q9UNQ0 | ABCG2 | 0.15 |
| Formononetin (7-Hydroxy-4'-methoxyisoflavone) | P30542 | ADORA1 | 0.16 |
| Formononetin (7-Hydroxy-4'-methoxyisoflavone) | P29274 | ADORA2A | 0.16 |
| Formononetin (7-Hydroxy-4'-methoxyisoflavone) | P05091 | ALDH2 | 0.18 |
| Formononetin (7-Hydroxy-4'-methoxyisoflavone) | O43570 | CA12 | 0.18 |
| Formononetin (7-Hydroxy-4'-methoxyisoflavone) | P11511 | CYP19A1 | 0.29 |
| Formononetin (7-Hydroxy-4'-methoxyisoflavone) | P60568 | IL2 | 1 |
| Formononetin-7-O-glucoside (Ononin) | P05091 | ALDH2 | 0.39 |
| Formononetin-7-O-glucoside (Ononin) | P01375 | TNF | 0.5 |
| Formononetin-7-O-glucoside (Ononin) | P60568 | IL2 | 0.99 |
| Gallic Acid Ethyl Ester; Ethyl gallate | Q11130 | FUT7 | 0.15 |
| Gallic Acid Ethyl Ester; Ethyl gallate | P00918 | CA2 | 0.33 |
| Gallic Acid Ethyl Ester; Ethyl gallate | P43166 | CA7 | 0.33 |
| Gallic Acid Ethyl Ester; Ethyl gallate | P00915 | CA1 | 0.33 |
| Gallic Acid Ethyl Ester; Ethyl gallate | O43570 | CA12 | 0.33 |
| Gallic Acid Ethyl Ester; Ethyl gallate | Q9ULX7 | CA14 | 0.33 |
| Gallic Acid Ethyl Ester; Ethyl gallate | Q16790 | CA9 | 0.33 |
| Gallic Acid Ethyl Ester; Ethyl gallate | Q14534 | SQLE | 0.91 |
| Genistein-7-O-Glucoside (Genistin) | P01375 | TNF | 0.18 |
| Genistein-7-O-Glucoside (Genistin) | P05091 | ALDH2 | 0.29 |
| Genistein-7-O-Glucoside (Genistin) | P60568 | IL2 | 0.29 |
| Glycitein | Q13332 | PTPRS | 0.12 |
| Glycitein | P22748 | CA4 | 0.12 |
| Glycitein | P16152 | CBR1 | 0.12 |
| Glycitein | P14061 | HSD17B1 | 0.13 |
| Glycitein | P43166 | CA7 | 0.13 |
| Glycitein | O43570 | CA12 | 0.13 |
| Glycitein | P11511 | CYP19A1 | 0.13 |
| Glycitein | P60568 | IL2 | 0.15 |
| Glycitein | P30542 | ADORA1 | 0.15 |
| Glycitein | P29274 | ADORA2A | 0.15 |
| Glycitein | P18054 | ALOX12 | 0.16 |
| Glycitein | P03372 | ESR1 | 0.17 |
| Glycitein | Q92731 | ESR2 | 0.17 |
| Glycitein | P37059 | HSD17B2 | 0.19 |
| Glycitein | P00533 | EGFR | 0.62 |
| Hesperetin | P33527 | ABCC1 | 0.13 |
| Hesperetin | P04278 | SHBG | 0.13 |
| Hesperetin | P16152 | CBR1 | 0.13 |
| Hesperetin | P45452 | MMP13 | 0.13 |
| Hesperetin | P03372 | ESR1 | 0.14 |
| Hesperetin | P27338 | MAOB | 0.14 |
| Hesperetin | Q92731 | ESR2 | 0.15 |
| Hesperetin | Q9UNQ0 | ABCG2 | 0.16 |
| Hesperetin | P14061 | HSD17B1 | 0.16 |
| Hesperetin | P59538 | TAS2R31 | 0.17 |
| Hesperetin | P30542 | ADORA1 | 0.17 |
| Hesperetin | P0DMS8 | ADORA3 | 0.17 |
| Hesperetin | P11511 | CYP19A1 | 0.21 |
| Hesperetin | P43166 | CA7 | 1 |
| Hesperetin | O43570 | CA12 | 1 |
| Hesperetin | P22748 | CA4 | 1 |
| Hesperetin | Q16678 | CYP1B1 | 1 |
| Hydroquinone | P22748 | CA4 | 0.16 |
| Hydroquinone | P35218 | CA5A | 0.21 |
| Hydroquinone | Q9Y2D0 | CA5B | 0.28 |
| Hydroquinone | P00918 | CA2 | 1 |
| Hydroquinone | P07451 | CA3 | 1 |
| Hydroquinone | O43570 | CA12 | 1 |
| Isoformononetin | P23975 | SLC6A2 | 0.13 |
| Isoformononetin | P21397 | MAOA | 0.14 |
| Isoformononetin | P27338 | MAOB | 0.14 |
| Isoformononetin | P30542 | ADORA1 | 0.15 |
| Isoformononetin | P29274 | ADORA2A | 0.15 |
| Isoformononetin | Q16875 | PFKFB3 | 0.17 |
| Isoformononetin | P14174 | MIF | 0.17 |
| Isoformononetin | P28223 | HTR2A | 0.17 |
| Isoformononetin | Q9UNQ0 | ABCG2 | 0.17 |
| Isoformononetin | P22748 | CA4 | 0.17 |
| Isoformononetin | P23219 | PTGS1 | 0.19 |
| Isoformononetin | P14061 | HSD17B1 | 0.2 |
| Isoformononetin | Q92731 | ESR2 | 0.21 |
| Isoformononetin | P43166 | CA7 | 0.22 |
| Isoformononetin | P11511 | CYP19A1 | 0.22 |
| Isoformononetin | P60568 | IL2 | 0.24 |
| Isoformononetin | P00533 | EGFR | 0.24 |
| Isoformononetin | O43570 | CA12 | 0.26 |
| Isoformononetin | P05091 | ALDH2 | 0.26 |
| Isoformononetin | P37059 | HSD17B2 | 0.31 |
| Isoformononetin | P03372 | ESR1 | 0.32 |
| Isoluteolin (Orobol)(5,7,3',4'-tetrahydroxyisoflavone) | P05091 | ALDH2 | 0.13 |
| Isoluteolin (Orobol)(5,7,3',4'-tetrahydroxyisoflavone) | Q16875 | PFKFB3 | 0.15 |
| Isoluteolin (Orobol)(5,7,3',4'-tetrahydroxyisoflavone) | P08183 | ABCB1 | 0.21 |
| Isoluteolin (Orobol)(5,7,3',4'-tetrahydroxyisoflavone) | P11511 | CYP19A1 | 0.21 |
| Isoluteolin (Orobol)(5,7,3',4'-tetrahydroxyisoflavone) | P16050 | ALOX15 | 0.23 |
| Isoluteolin (Orobol)(5,7,3',4'-tetrahydroxyisoflavone) | P37059 | HSD17B2 | 0.25 |
| Isoluteolin (Orobol)(5,7,3',4'-tetrahydroxyisoflavone) | P24557 | TBXAS1 | 0.33 |
| Isoluteolin (Orobol)(5,7,3',4'-tetrahydroxyisoflavone) | P03372 | ESR1 | 0.33 |
| Isoluteolin (Orobol)(5,7,3',4'-tetrahydroxyisoflavone) | O43451 | MGAM | 0.33 |
| Isoluteolin (Orobol)(5,7,3',4'-tetrahydroxyisoflavone) | P28223 | HTR2A | 0.33 |
| Isoluteolin (Orobol)(5,7,3',4'-tetrahydroxyisoflavone) | P28335 | HTR2C | 0.33 |
| Isoluteolin (Orobol)(5,7,3',4'-tetrahydroxyisoflavone) | Q92731 | ESR2 | 0.33 |
| Isoluteolin (Orobol)(5,7,3',4'-tetrahydroxyisoflavone) | O95718 | ESRRB | 0.33 |
| Isoluteolin (Orobol)(5,7,3',4'-tetrahydroxyisoflavone) | Q9NPH5 | NOX4 | 0.33 |
| Isoluteolin (Orobol)(5,7,3',4'-tetrahydroxyisoflavone) | P47989 | XDH | 0.34 |
| Isoluteolin (Orobol)(5,7,3',4'-tetrahydroxyisoflavone) | P00533 | EGFR | 0.39 |
| Isoluteolin (Orobol)(5,7,3',4'-tetrahydroxyisoflavone) | P29274 | ADORA2A | 0.39 |
| Isoluteolin (Orobol)(5,7,3',4'-tetrahydroxyisoflavone) | P43166 | CA7 | 0.43 |
| Isoluteolin (Orobol)(5,7,3',4'-tetrahydroxyisoflavone) | O43570 | CA12 | 0.43 |
| Isoluteolin (Orobol)(5,7,3',4'-tetrahydroxyisoflavone) | P22748 | CA4 | 0.43 |
| Isoluteolin (Orobol)(5,7,3',4'-tetrahydroxyisoflavone) | P14061 | HSD17B1 | 0.46 |
| Isoluteolin (Orobol)(5,7,3',4'-tetrahydroxyisoflavone) | P11474 | ESRRA | 0.46 |
| Isoluteolin (Orobol)(5,7,3',4'-tetrahydroxyisoflavone) | P18054 | ALOX12 | 0.49 |
| Isoluteolin (Orobol)(5,7,3',4'-tetrahydroxyisoflavone) | P21397 | MAOA | 0.52 |
| Isoluteolin (Orobol)(5,7,3',4'-tetrahydroxyisoflavone) | P30542 | ADORA1 | 0.52 |
| Isoluteolin (Orobol)(5,7,3',4'-tetrahydroxyisoflavone) | Q9UNQ0 | ABCG2 | 0.52 |
| Isoluteolin (Orobol)(5,7,3',4'-tetrahydroxyisoflavone) | P14174 | MIF | 0.55 |
| Isoluteolin (Orobol)(5,7,3',4'-tetrahydroxyisoflavone) | P14679 | TYR | 0.68 |
| Isoluteolin (Orobol)(5,7,3',4'-tetrahydroxyisoflavone) | Q13332 | PTPRS | 0.79 |
| Kaempferol-3-O-galactoside (Trifolin)* | P28907 | CD38 | 0.12 |
| Kaempferol-3-O-galactoside (Trifolin)* | O76074 | PDE5A | 0.12 |
| Kaempferol-3-O-galactoside (Trifolin)* | Q9GZQ4 | NMUR2 | 0.18 |
| Kaempferol-3-O-galactoside (Trifolin)* | P08913 | ADRA2A | 0.18 |
| Kaempferol-3-O-galactoside (Trifolin)* | P51812 | RPS6KA3 | 0.65 |
| Kaempferol-3-O-galactoside (Trifolin)* | Q9NPH5 | NOX4 | 0.92 |
| Kaempferol-3-O-galactoside (Trifolin)* | P18825 | ADRA2C | 0.92 |
| Kaempferol-3-O-galactoside (Trifolin)* | P16083 | NQO2 | 0.92 |
| Kaempferol-3-O-galactoside (Trifolin)* | P15121 | AKR1B1 | 1 |
| Kaempferol-3-O-galactoside (Trifolin)* | P00918 | CA2 | 1 |
| Kaempferol-3-O-galactoside (Trifolin)* | P43166 | CA7 | 1 |
| Kaempferol-3-O-galactoside (Trifolin)* | O43570 | CA12 | 1 |
| Kaempferol-3-O-galactoside (Trifolin)* | P22748 | CA4 | 1 |
| Kaempferol-3-O-galactoside (Trifolin)* | P22303 | ACHE | 1 |
| Kaempferol-3-O-glucoside (Astragalin)* | P28907 | CD38 | 0.12 |
| Kaempferol-3-O-glucoside (Astragalin)* | O76074 | PDE5A | 0.12 |
| Kaempferol-3-O-glucoside (Astragalin)* | Q9GZQ4 | NMUR2 | 0.18 |
| Kaempferol-3-O-glucoside (Astragalin)* | P08913 | ADRA2A | 0.18 |
| Kaempferol-3-O-glucoside (Astragalin)* | P51812 | RPS6KA3 | 0.65 |
| Kaempferol-3-O-glucoside (Astragalin)* | Q9NPH5 | NOX4 | 0.92 |
| Kaempferol-3-O-glucoside (Astragalin)* | P18825 | ADRA2C | 0.92 |
| Kaempferol-3-O-glucoside (Astragalin)* | P16083 | NQO2 | 0.92 |
| Kaempferol-3-O-glucoside (Astragalin)* | P15121 | AKR1B1 | 1 |
| Kaempferol-3-O-glucoside (Astragalin)* | P00918 | CA2 | 1 |
| Kaempferol-3-O-glucoside (Astragalin)* | P43166 | CA7 | 1 |
| Kaempferol-3-O-glucoside (Astragalin)* | O43570 | CA12 | 1 |
| Kaempferol-3-O-glucoside (Astragalin)* | P22748 | CA4 | 1 |
| Kaempferol-3-O-glucoside (Astragalin)* | P22303 | ACHE | 1 |
| Kaempferol-3-O-neohesperidoside* | P16083 | NQO2 | 0.15 |
| Kaempferol-3-O-neohesperidoside* | Q9NPH5 | NOX4 | 0.21 |
| Kaempferol-3-O-neohesperidoside* | P51812 | RPS6KA3 | 0.22 |
| Kaempferol-3-O-neohesperidoside* | P00918 | CA2 | 0.24 |
| Kaempferol-3-O-neohesperidoside* | P43166 | CA7 | 0.29 |
| Kaempferol-3-O-neohesperidoside* | O43570 | CA12 | 0.29 |
| Kaempferol-3-O-neohesperidoside* | P22748 | CA4 | 0.29 |
| Kaempferol-3-O-neohesperidoside* | P15121 | AKR1B1 | 0.38 |
| Kaempferol-3-O-neohesperidoside* | Q9GZQ4 | NMUR2 | 0.81 |
| Kaempferol-3-O-neohesperidoside* | P08913 | ADRA2A | 0.81 |
| Kaempferol-3-O-neohesperidoside* | P18825 | ADRA2C | 0.81 |
| Kaempferol-3-O-neohesperidoside* | P22303 | ACHE | 0.81 |
| L-Tryptophan | P14902 | IDO1 | 0.17 |
| L-Tryptophan | P41595 | HTR2B | 0.18 |
| L-Tryptophan | P08908 | HTR1A | 0.18 |
| L-Tryptophan | P28223 | HTR2A | 0.18 |
| L-Tryptophan | P28335 | HTR2C | 0.18 |
| L-Tryptophan | P34969 | HTR7 | 0.18 |
| Manninotriose | P15692 | VEGFA | 0.12 |
| Manninotriose | P05230 | FGF1 | 0.12 |
| Manninotriose | Q9Y251 | HPSE | 0.12 |
| Manninotriose | P40763 | STAT3 | 0.12 |
| Manninotriose | P23415 | GLRA1 | 0.12 |
| Manninotriose | P23416 | GLRA2 | 0.12 |
| Manninotriose | P49810 Q9NZ42 Q92542 Q96BI3 P49768 Q8WW43 | PSEN2 PSENEN NCSTN APH1A PSEN1 APH1B | 0.12 |
| Manninotriose | P35813 | PPM1A | 0.12 |
| Manninotriose | P56470 | LGALS4 | 0.12 |
| Manninotriose | O00214 | LGALS8 | 0.12 |
| Manninotriose | P09038 | FGF2 | 0.12 |
| Manninotriose | P11473 | VDR | 0.12 |
| Manninotriose | P25105 | PTAFR | 0.12 |
| Manninotriose | P80365 | HSD11B2 | 0.12 |
| Manninotriose | P28845 | HSD11B1 | 0.12 |
| Manninotriose | O43451 | MGAM | 0.12 |
| Manninotriose | P17931 | LGALS3 | 0.12 |
| Manninotriose | P04746 | AMY2A | 0.12 |
| Manninotriose | P41145 | OPRK1 | 0.12 |
| Manninotriose | P04745 | AMY1A | 0.12 |
| Manninotriose | P37268 | FDFT1 | 0.12 |
| Manninotriose | P06493 | CDK1 | 0.14 |
| Manninotriose | P41595 | HTR2B | 0.15 |
| Manninotriose | P08913 | ADRA2A | 0.15 |
| Manninotriose | P18825 | ADRA2C | 0.15 |
| Manninotriose | P18089 | ADRA2B | 0.15 |
| Manninotriose | P21728 | DRD1 | 0.15 |
| Manninotriose | P14416 | DRD2 | 0.15 |
| Manninotriose | P25100 | ADRA1D | 0.15 |
| Manninotriose | P28223 | HTR2A | 0.15 |
| Manninotriose | P28335 | HTR2C | 0.15 |
| Manninotriose | P35462 | DRD3 | 0.15 |
| Manninotriose | P10635 | CYP2D6 | 0.15 |
| Manninotriose | P50406 | HTR6 | 0.15 |
| Manninotriose | P35348 | ADRA1A | 0.15 |
| Manninotriose | P28222 | HTR1B | 0.15 |
| Manninotriose | P51449 | RORC | 0.15 |
| Melibiose | P09038 | FGF2 | 0.13 |
| Melibiose | P15692 | VEGFA | 0.22 |
| Melibiose | P05230 | FGF1 | 0.22 |
| Melibiose | Q9Y251 | HPSE | 0.22 |
| Melibiose | P06493 | CDK1 | 0.23 |
| Methyl 4-hydroxybenzoate | P07451 | CA3 | 0.13 |
| Methyl 4-hydroxybenzoate | Q92731 | ESR2 | 0.25 |
| Methyl 4-hydroxybenzoate | P00918 | CA2 | 0.86 |
| Methyl 4-hydroxybenzoate | P43166 | CA7 | 0.86 |
| Methyl 4-hydroxybenzoate | P00915 | CA1 | 0.86 |
| Methyl 4-hydroxybenzoate | O43570 | CA12 | 0.86 |
| Methyl 4-hydroxybenzoate | Q9ULX7 | CA14 | 0.86 |
| Methyl 4-hydroxybenzoate | Q16790 | CA9 | 0.86 |
| Methyl gallate* | P07451 | CA3 | 0.12 |
| Methyl gallate* | P23280 | CA6 | 0.12 |
| Methyl gallate* | P22748 | CA4 | 0.12 |
| Methyl gallate* | Q9Y2D0 | CA5B | 0.12 |
| Methyl gallate* | P35218 | CA5A | 0.12 |
| Methyl gallate* | Q8N1Q1 | CA13 | 0.12 |
| Methyl gallate* | P00918 | CA2 | 0.21 |
| Methyl gallate* | P43166 | CA7 | 0.21 |
| Methyl gallate* | P00915 | CA1 | 0.21 |
| Methyl gallate* | O43570 | CA12 | 0.21 |
| Methyl gallate* | Q9ULX7 | CA14 | 0.21 |
| Methyl gallate* | Q16790 | CA9 | 0.21 |
| Methyl gallate* | Q11130 | FUT7 | 0.73 |
| Mudanpinoic acid A | O14684 | PTGES | 0.14 |
| Mudanpinoic acid A | P60033 | CD81 | 0.17 |
| Mudanpinoic acid A | P04054 | PLA2G1B | 0.44 |
| Mudanpinoic acid A | P30305 | CDC25B | 0.44 |
| Mudanpinoic acid A | Q08499 | PDE4D | 0.48 |
| Mudanpinoic acid A | P51449 | RORC | 0.56 |
| Mudanpinoic acid A | P06746 | POLB | 0.56 |
| Mudanpinoic acid A | P18031 | PTPN1 | 0.56 |
| Mudanpinoic acid A | P10586 | PTPRF | 0.56 |
| Mudanpinoic acid A | P17706 | PTPN2 | 0.56 |
| Mudanpinoic acid A | P28845 | HSD11B1 | 0.56 |
| Mudanpinoic acid A | P24666 | ACP1 | 0.56 |
| Mudanpinoic acid A | O60218 | AKR1B10 | 0.56 |
| Nobiletin (5,6,7,8,3',4'-Hexamethoxyflavone)* | P35372 | OPRM1 | 0.12 |
| Nobiletin (5,6,7,8,3',4'-Hexamethoxyflavone)* | P10721 | KIT | 0.16 |
| Nobiletin (5,6,7,8,3',4'-Hexamethoxyflavone)* | Q16678 | CYP1B1 | 0.25 |
| Nobiletin (5,6,7,8,3',4'-Hexamethoxyflavone)* | P41143 | OPRD1 | 0.26 |
| Nobiletin (5,6,7,8,3',4'-Hexamethoxyflavone)* | P15121 | AKR1B1 | 0.42 |
| Nobiletin (5,6,7,8,3',4'-Hexamethoxyflavone)* | Q9UNQ0 | ABCG2 | 0.94 |
| Oleic acid | P48147 | PREP | 0.13 |
| Oleic acid | P29350 | PTPN6 | 0.13 |
| Oleic acid | P09917 | ALOX5 | 0.13 |
| Oleic acid | O14684 | PTGES | 0.13 |
| Oleic acid | P11387 | TOP1 | 0.14 |
| Oleic acid | P04035 | HMGCR | 0.14 |
| Oleic acid | P23219 | PTGS1 | 0.15 |
| Oleic acid | O14842 | FFAR1 | 0.15 |
| Oleic acid | P17706 | PTPN2 | 0.16 |
| Oleic acid | P18031 | PTPN1 | 0.17 |
| Oleic acid | P05413 | FABP3 | 0.57 |
| Oleic acid | O00767 | SCD | 0.59 |
| Oleic acid | P15090 | FABP4 | 1 |
| Oleic acid | O00519 | FAAH | 1 |
| Oleic acid | P37231 | PPARG | 1 |
| Oleic acid | Q07869 | PPARA | 1 |
| Oleic acid | O14746 | TERT | 1 |
| Oleic acid | Q01469 | FABP5 | 1 |
| Oleic acid | Q03181 | PPARD | 1 |
| Oleic acid | P07148 | FABP1 | 1 |
| Pectolinarigenin | P03372 | ESR1 | 0.13 |
| Pectolinarigenin | Q92731 | ESR2 | 0.13 |
| Pectolinarigenin | Q9NPH5 | NOX4 | 0.13 |
| Pectolinarigenin | P14061 | HSD17B1 | 0.13 |
| Pectolinarigenin | P35228 | NOS2 | 0.15 |
| Pectolinarigenin | Q16678 | CYP1B1 | 0.17 |
| Pectolinarigenin | P36888 | FLT3 | 0.18 |
| Pectolinarigenin | P0DMS8 | ADORA3 | 0.2 |
| Pectolinarigenin | P11309 | PIM1 | 0.23 |
| Pectolinarigenin | P30542 | ADORA1 | 0.23 |
| Pectolinarigenin | P29274 | ADORA2A | 0.23 |
| Pectolinarigenin | P15121 | AKR1B1 | 0.34 |
| Pectolinarigenin | P10721 | KIT | 0.97 |
| Pectolinarigenin | P41143 | OPRD1 | 0.97 |
| Procyanidin B1/B2 | P14780 | MMP9 | 0.21 |
| Procyanidin B1/B2 | P49763 | PGF | 0.35 |
| Procyanidin B1/B2 | P15692 | VEGFA | 0.35 |
| Procyanidin B1/B2 | P08253 | MMP2 | 0.58 |
| Quercetin | O14746 | TERT | 0.2 |
| Quercetin | P05067 | APP | 0.26 |
| Quercetin | P09874 | PARP1 | 0.26 |
| Quercetin | P02766 | TTR | 0.26 |
| Quercetin | P39900 | MMP12 | 0.26 |
| Quercetin | P28907 | CD38 | 0.26 |
| Quercetin | O60218 | AKR1B10 | 0.26 |
| Quercetin | Q9H2K2 | TNKS2 | 0.26 |
| Quercetin | O95271 | TNKS | 0.26 |
| Quercetin | P11387 | TOP1 | 0.26 |
| Quercetin | P14679 | TYR | 0.4 |
| Quercetin | P14061 | HSD17B1 | 0.4 |
| Quercetin | P35869 | AHR | 0.4 |
| Quercetin | P11474 | ESRRA | 0.4 |
| Quercetin | Q00534 | CDK6 | 0.5 |
| Quercetin | P24941 | CDK2 | 0.5 |
| Quercetin | Q15078 Q00535 | CDK5R1 CDK5 | 0.54 |
| Quercetin | Q8WWL7 P06493 P14635 O95067 | CCNB3 CDK1 CCNB1 CCNB2 | 0.54 |
| Quercetin | P05089 | ARG1 | 0.54 |
| Quercetin | Q13332 | PTPRS | 0.55 |
| Quercetin | Q92731 | ESR2 | 0.55 |
| Quercetin | P29372 | MPG | 0.55 |
| Quercetin | Q96S37 | SLC22A12 | 0.55 |
| Quercetin | P10636 | MAPT | 0.68 |
| Quercetin | B2RXH2 | KDM4E | 0.68 |
| Quercetin | P11388 | TOP2A | 0.68 |
| Quercetin | P06213 | INSR | 0.68 |
| Quercetin | P22303 | ACHE | 0.68 |
| Quercetin | Q15746 | MYLK | 0.68 |
| Quercetin | P43405 | SYK | 0.68 |
| Quercetin | P48736 | PIK3CG | 0.68 |
| Quercetin | P27695 | APEX1 | 0.68 |
| Quercetin | Q9NPH5 | NOX4 | 1 |
| Quercetin | P30518 | AVPR2 | 1 |
| Quercetin | P15121 | AKR1B1 | 1 |
| Quercetin | P47989 | XDH | 1 |
| Quercetin | P21397 | MAOA | 1 |
| Quercetin | P08069 | IGF1R | 1 |
| Quercetin | P36888 | FLT3 | 1 |
| Quercetin | P11511 | CYP19A1 | 1 |
| Quercetin | P00533 | EGFR | 1 |
| Quercetin | P00734 | F2 | 1 |
| Quercetin | P00918 | CA2 | 1 |
| Quercetin | P11309 | PIM1 | 1 |
| Quercetin | P09917 | ALOX5 | 1 |
| Quercetin | Q96GD4 | AURKB | 1 |
| Quercetin | P21917 | DRD4 | 1 |
| Quercetin | P30542 | ADORA1 | 1 |
| Quercetin | P43166 | CA7 | 1 |
| Quercetin | Q04760 | GLO1 | 1 |
| Quercetin | P05164 | MPO | 1 |
| Quercetin | P27986 | PIK3R1 | 1 |
| Quercetin | P29274 | ADORA2A | 1 |
| Quercetin | P53355 | DAPK1 | 1 |
| Quercetin | P06737 | PYGL | 1 |
| Quercetin | P00915 | CA1 | 1 |
| Quercetin | P49841 | GSK3B | 1 |
| Quercetin | P12931 | SRC | 1 |
| Quercetin | Q05397 | PTK2 | 1 |
| Quercetin | P37059 | HSD17B2 | 1 |
| Quercetin | P35968 | KDR | 1 |
| Quercetin | P45452 | MMP13 | 1 |
| Quercetin | P08254 | MMP3 | 1 |
| Quercetin | P07451 | CA3 | 1 |
| Quercetin | P16050 | ALOX15 | 1 |
| Quercetin | P33527 | ABCC1 | 1 |
| Quercetin | P53350 | PLK1 | 1 |
| Quercetin | P23280 | CA6 | 1 |
| Quercetin | P06493 | CDK1 | 1 |
| Quercetin | P14780 | MMP9 | 1 |
| Quercetin | O43570 | CA12 | 1 |
| Quercetin | P08253 | MMP2 | 1 |
| Quercetin | Q16512 | PKN1 | 1 |
| Quercetin | Q9ULX7 | CA14 | 1 |
| Quercetin | Q16790 | CA9 | 1 |
| Quercetin | P68400 | CSNK2A1 | 1 |
| Quercetin | P18054 | ALOX12 | 1 |
| Quercetin | P08581 | MET | 1 |
| Quercetin | P22748 | CA4 | 1 |
| Quercetin | P51955 | NEK2 | 1 |
| Quercetin | P25024 | CXCR1 | 1 |
| Quercetin | Q13554 | CAMK2B | 1 |
| Quercetin | Q9UM73 | ALK | 1 |
| Quercetin | P31749 | AKT1 | 1 |
| Quercetin | P08183 | ABCB1 | 1 |
| Quercetin | Q9HC98 | NEK6 | 1 |
| Quercetin | P04054 | PLA2G1B | 1 |
| Quercetin | P35218 | CA5A | 1 |
| Quercetin | P56817 | BACE1 | 1 |
| Quercetin | Q16678 | CYP1B1 | 1 |
| Quercetin | P30530 | AXL | 1 |
| Quercetin | Q9UNQ0 | ABCG2 | 1 |
| Quercetin | O60285 | NUAK1 | 1 |
| Quercetin | P52895 | AKR1C2 | 1 |
| Quercetin | Q04828 | AKR1C1 | 1 |
| Quercetin | P42330 | AKR1C3 | 1 |
| Quercetin | P17516 | AKR1C4 | 1 |
| Quercetin | Q8N1Q1 | CA13 | 1 |
| Quercetin | P14550 | AKR1A1 | 1 |
| Quercetin | Q9HC97 | GPR35 | 1 |
| Quercetin-3-O-glucoside (Isoquercitrin)* | P28907 | CD38 | 0.13 |
| Quercetin-3-O-glucoside (Isoquercitrin)* | P35354 | PTGS2 | 0.16 |
| Quercetin-3-O-glucoside (Isoquercitrin)* | Q9GZQ4 | NMUR2 | 0.37 |
| Quercetin-3-O-glucoside (Isoquercitrin)* | P08913 | ADRA2A | 0.37 |
| Quercetin-3-O-glucoside (Isoquercitrin)* | P51812 | RPS6KA3 | 0.43 |
| Quercetin-3-O-glucoside (Isoquercitrin)* | P16083 | NQO2 | 0.58 |
| Quercetin-3-O-glucoside (Isoquercitrin)* | P22303 | ACHE | 0.8 |
| Quercetin-3-O-glucoside (Isoquercitrin)* | Q9NPH5 | NOX4 | 1 |
| Quercetin-3-O-glucoside (Isoquercitrin)* | P18825 | ADRA2C | 1 |
| Quercetin-3-O-glucoside (Isoquercitrin)* | P15121 | AKR1B1 | 1 |
| Quercetin-3-O-glucoside (Isoquercitrin)* | P00918 | CA2 | 1 |
| Quercetin-3-O-glucoside (Isoquercitrin)* | P43166 | CA7 | 1 |
| Quercetin-3-O-glucoside (Isoquercitrin)* | O43570 | CA12 | 1 |
| Quercetin-3-O-glucoside (Isoquercitrin)* | P22748 | CA4 | 1 |
| Quercetin-3-O-rhamnoside(Quercitrin) | P28907 | CD38 | 0.13 |
| Quercetin-3-O-rhamnoside(Quercitrin) | P35354 | PTGS2 | 0.13 |
| Quercetin-3-O-rhamnoside(Quercitrin) | Q9GZQ4 | NMUR2 | 0.27 |
| Quercetin-3-O-rhamnoside(Quercitrin) | P08913 | ADRA2A | 0.27 |
| Quercetin-3-O-rhamnoside(Quercitrin) | P51812 | RPS6KA3 | 0.76 |
| Quercetin-3-O-rhamnoside(Quercitrin) | Q9NPH5 | NOX4 | 0.91 |
| Quercetin-3-O-rhamnoside(Quercitrin) | P18825 | ADRA2C | 0.91 |
| Quercetin-3-O-rhamnoside(Quercitrin) | P16083 | NQO2 | 0.92 |
| Quercetin-3-O-rhamnoside(Quercitrin) | P22303 | ACHE | 0.93 |
| Quercetin-3-O-rhamnoside(Quercitrin) | P15121 | AKR1B1 | 1 |
| Quercetin-3-O-rhamnoside(Quercitrin) | P00918 | CA2 | 1 |
| Quercetin-3-O-rhamnoside(Quercitrin) | P43166 | CA7 | 1 |
| Quercetin-3-O-rhamnoside(Quercitrin) | O43570 | CA12 | 1 |
| Quercetin-3-O-rhamnoside(Quercitrin) | P22748 | CA4 | 1 |
| Quercetin-3-O-rutinoside (Rutin) | P51812 | RPS6KA3 | 0.23 |
| Quercetin-3-O-rutinoside (Rutin) | P16083 | NQO2 | 0.24 |
| Quercetin-3-O-rutinoside (Rutin) | P00918 | CA2 | 0.4 |
| Quercetin-3-O-rutinoside (Rutin) | Q9NPH5 | NOX4 | 0.44 |
| Quercetin-3-O-rutinoside (Rutin) | P43166 | CA7 | 0.48 |
| Quercetin-3-O-rutinoside (Rutin) | O43570 | CA12 | 0.48 |
| Quercetin-3-O-rutinoside (Rutin) | P22748 | CA4 | 0.48 |
| Quercetin-3-O-rutinoside (Rutin) | P15121 | AKR1B1 | 0.6 |
| Quercetin-3-O-rutinoside (Rutin) | Q9GZQ4 | NMUR2 | 1 |
| Quercetin-3-O-rutinoside (Rutin) | P08913 | ADRA2A | 1 |
| Quercetin-3-O-rutinoside (Rutin) | P18825 | ADRA2C | 1 |
| Quercetin-3-O-rutinoside (Rutin) | P22303 | ACHE | 1 |
| Raffinose* | P56470 | LGALS4 | 0.12 |
| Raffinose* | O00214 | LGALS8 | 0.12 |
| Raffinose* | P09038 | FGF2 | 0.12 |
| Raffinose* | P49810 Q9NZ42 Q92542 Q96BI3 P49768 Q8WW43 | PSEN2 PSENEN NCSTN APH1A PSEN1 APH1B | 0.12 |
| Raffinose* | P41595 | HTR2B | 0.12 |
| Raffinose* | P08913 | ADRA2A | 0.12 |
| Raffinose* | P18825 | ADRA2C | 0.12 |
| Raffinose* | P18089 | ADRA2B | 0.12 |
| Raffinose* | P21728 | DRD1 | 0.12 |
| Raffinose* | P14416 | DRD2 | 0.12 |
| Raffinose* | P25100 | ADRA1D | 0.12 |
| Raffinose* | P28223 | HTR2A | 0.12 |
| Raffinose* | P28335 | HTR2C | 0.12 |
| Raffinose* | P35462 | DRD3 | 0.12 |
| Raffinose* | P10635 | CYP2D6 | 0.12 |
| Raffinose* | P50406 | HTR6 | 0.12 |
| Raffinose* | P35348 | ADRA1A | 0.12 |
| Raffinose* | P28222 | HTR1B | 0.12 |
| Raffinose* | P07900 | HSP90AA1 | 0.12 |
| Raffinose* | P17931 | LGALS3 | 0.12 |
| Raffinose* | P51449 | RORC | 0.12 |
| Raffinose* | Q8NER1 | TRPV1 | 0.12 |
| Raffinose* | P04746 | AMY2A | 0.12 |
| Raffinose* | P04745 | AMY1A | 0.12 |
| Raffinose* | P40763 | STAT3 | 0.12 |
| Raffinose* | P15692 | VEGFA | 0.15 |
| Raffinose* | P05230 | FGF1 | 0.15 |
| Raffinose* | Q9Y251 | HPSE | 0.15 |
| Raffinose* | P06493 | CDK1 | 0.83 |
| Sinapic acid | P43166 | CA7 | 0.27 |
| Sinapic acid | P23280 | CA6 | 0.27 |
| Sinapic acid | O43570 | CA12 | 0.27 |
| Sinapic acid | Q9ULX7 | CA14 | 0.27 |
| Sinapic acid | Q16790 | CA9 | 0.27 |
| Sinapic acid | P35218 | CA5A | 0.27 |
| Sinapic acid | P00918 | CA2 | 0.29 |
| Sinapic acid | P00915 | CA1 | 0.29 |
| Syringic acid | P00918 | CA2 | 1 |
| Syringic acid | P43166 | CA7 | 1 |
| Syringic acid | P00915 | CA1 | 1 |
| Syringic acid | P07451 | CA3 | 1 |
| Syringic acid | P23280 | CA6 | 1 |
| Syringic acid | O43570 | CA12 | 1 |
| Syringic acid | Q9ULX7 | CA14 | 1 |
| Syringic acid | Q16790 | CA9 | 1 |
| Syringic acid | P35218 | CA5A | 1 |
| Uridine | P11217 | PYGM | 0.15 |
| Uridine | P00813 | ADA | 0.26 |
| Uridine | P32320 | CDA | 0.87 |
| Vanillic acid | P06241 | FYN | 0.13 |
| Vanillic acid | P06239 | LCK | 0.13 |
| Vanillic acid | P09467 | FBP1 | 0.13 |
| Vanillic acid | P42330 | AKR1C3 | 0.13 |
| Vanillic acid | Q9Y2K7 | KDM2A | 0.13 |
| Vanillic acid | P14780 | MMP9 | 0.13 |
| Vanillic acid | P03956 | MMP1 | 0.13 |
| Vanillic acid | P08253 | MMP2 | 0.13 |
| Vanillic acid | P22894 | MMP8 | 0.13 |
| Vanillic acid | Q14534 | SQLE | 0.13 |
| Vanillic acid | Q11130 | FUT7 | 0.13 |
| Vanillic acid | B2RXH2 | KDM4E | 0.13 |
| Vanillic acid | O75164 | KDM4A | 0.13 |
| Vanillic acid | Q9Y4C1 | KDM3A | 0.13 |
| Vanillic acid | O15054 | KDM6B | 0.13 |
| Vanillic acid | Q9C0B1 | FTO | 0.13 |
| Vanillic acid | Q9H3R0 | KDM4C | 0.13 |
| Vanillic acid | Q9Y2D0 | CA5B | 0.14 |
| Vanillic acid | Q8N1Q1 | CA13 | 0.14 |
| Vanillic acid | P02766 | TTR | 0.15 |
| Vanillic acid | P51580 | TPMT | 0.18 |
| Vanillic acid | P22748 | CA4 | 0.21 |
| Vanillic acid | P07451 | CA3 | 0.27 |
| Vanillic acid | P23280 | CA6 | 0.27 |
| Vanillic acid | P35218 | CA5A | 0.27 |
| Vanillic acid | P00918 | CA2 | 0.32 |
| Vanillic acid | P43166 | CA7 | 0.32 |
| Vanillic acid | P00915 | CA1 | 0.32 |
| Vanillic acid | O43570 | CA12 | 0.32 |
| Vanillic acid | Q9ULX7 | CA14 | 0.32 |
| Vanillic acid | Q16790 | CA9 | 0.32 |
| Vanillin; 4-Hydroxy-3-Methoxybenzaldehyde | P00918 | CA2 | 0.17 |
| Vanillin; 4-Hydroxy-3-Methoxybenzaldehyde | O75460 | ERN1 | 0.26 |
| Wogonin (5,7-Dihydroxy-8-Methoxyflavone) | O14920 | IKBKB | 0.12 |
| Wogonin (5,7-Dihydroxy-8-Methoxyflavone) | Q16620 | NTRK2 | 0.12 |
| Wogonin (5,7-Dihydroxy-8-Methoxyflavone) | P10721 | KIT | 0.13 |
| Wogonin (5,7-Dihydroxy-8-Methoxyflavone) | P08183 | ABCB1 | 0.13 |
| Wogonin (5,7-Dihydroxy-8-Methoxyflavone) | P41143 | OPRD1 | 0.14 |
| Wogonin (5,7-Dihydroxy-8-Methoxyflavone) | P15121 | AKR1B1 | 0.23 |
| Wogonin (5,7-Dihydroxy-8-Methoxyflavone) | P36888 | FLT3 | 0.25 |
| Wogonin (5,7-Dihydroxy-8-Methoxyflavone) | P35354 | PTGS2 | 1 |
| Wogonin (5,7-Dihydroxy-8-Methoxyflavone) | P35228 | NOS2 | 1 |
| Oleic acid | Q07869 | PPARA | 1 |
| Oleic acid | O14746 | TERT | 1 |
| Oleic acid | Q01469 | FABP5 | 1 |
| Oleic acid | Q03181 | PPARD | 1 |
| Oleic acid | P07148 | FABP1 | 1 |
| Oleic acid | O00767 | SCD | 0.59 |
| Oleic acid | P05413 | FABP3 | 0.57 |
| Oleic acid | P18031 | PTPN1 | 0.17 |
| Oleic acid | P17706 | PTPN2 | 0.16 |
| Oleic acid | P23219 | PTGS1 | 0.15 |
| Oleic acid | O14842 | FFAR1 | 0.15 |
| Oleic acid | P11387 | TOP1 | 0.14 |
| Oleic acid | P04035 | HMGCR | 0.14 |
| Oleic acid | P48147 | PREP | 0.13 |
| Oleic acid | P29350 | PTPN6 | 0.13 |
| Oleic acid | P09917 | ALOX5 | 0.13 |
| Oleic acid | O14684 | PTGES | 0.13 |
| Procyanidin B1/B2 | P08253 | MMP2 | 0.58 |
| Procyanidin B1/B2 | P49763 | PGF | 0.35 |
| Procyanidin B1/B2 | P15692 | VEGFA | 0.35 |
| Procyanidin B1/B2 | P14780 | MMP9 | 0.21 |
| Procyanidin B1/B2 | NLR family, pyrin domain containing 3 | NLRP3 |  |
| Alisol B Acetate | O00748 | CES2 | 0.14 |
| Alisol B Acetate | P17252 | PRKCA | 0.13 |
| Alisol B Acetate | Q04759 | PRKCQ | 0.13 |
| Alisol B Acetate | Q05655 | PRKCD | 0.13 |
| Alisol B Acetate | P04035 | HMGCR | 0.13 |
| Alisol B Acetate | P35228 | NOS2 | 0.13 |
| Alisol B Acetate | P28845 | HSD11B1 | 0.13 |
| Alisol B Acetate | P35354 | PTGS2 | 0.13 |
| Alisol B Acetate | P49354 P49356 | FNTA FNTB | 0.13 |
| Alisol B Acetate | P53609 P49354 | PGGT1B FNTA | 0.13 |
| Alisol B Acetate | P18031 | PTPN1 | 0.13 |
| Alisol B Acetate | P50579 | METAP2 | 0.13 |
| Alisol B Acetate | P05771 | PRKCB | 0.13 |
| Alisol B Acetate | P05129 | PRKCG | 0.13 |
| Alisol B Acetate | Q02156 | PRKCE | 0.13 |
| Alisol B Acetate | P24723 | PRKCH | 0.13 |
| Alisol B Acetate | P25103 | TACR1 | 0.13 |
| Alisol B Acetate | Q9HBA0 | TRPV4 | 0.13 |
| Alisol B Acetate | P56373 | P2RX3 | 0.13 |
| Alisol B Acetate | P52333 | JAK3 | 0.13 |
| Alisol B Acetate | P21730 | C5AR1 | 0.13 |
| Alisol B Acetate | P21554 | CNR1 | 0.13 |
| Alisol B Acetate | P11511 | CYP19A1 | 0.13 |
| Alisol B Acetate | O15111 | CHUK | 0.13 |
| Alisol B Acetate | P21917 | DRD4 | 0.13 |
| Alisol B Acetate | P35462 | DRD3 | 0.13 |
| Alisol B Acetate | P34972 | CNR2 | 0.13 |
| Alisol B Acetate | P37288 | AVPR1A | 0.13 |
| Alisol B Acetate | Q08499 | PDE4D | 0.13 |
| Alisol B Acetate | P32246 | CCR1 | 0.13 |
| Alisol B Acetate | P35968 | KDR | 0.13 |
| Alisol B Acetate | P25116 | F2R | 0.13 |
| Alisol B Acetate | Q00987 | MDM2 | 0.13 |
| Alisol B Acetate | Q9Y5Z0 | BACE2 | 0.13 |
| Alisol B Acetate | P07339 | CTSD | 0.13 |
| Alisol B Acetate | O15055 | PER2 | 0.13 |
| Alisol B Acetate | P37058 | HSD17B3 | 0.13 |
| Alisol B Acetate | Q99558 | MAP3K14 | 0.13 |
| Alisol B Acetate | Q9Y233 | PDE10A | 0.13 |
| Alisol B Acetate | Q01959 | SLC6A3 | 0.13 |
| Alisol B Acetate | P06241 | FYN | 0.13 |
| Alisol B Acetate | P00533 | EGFR | 0.13 |
| Alisol B Acetate | P16234 P09619 | PDGFRA PDGFRB | 0.13 |
| Alisol B Acetate | P08842 | STS | 0.13 |
| Alisol B Acetate | P28482 | MAPK1 | 0.13 |
| Alisol B Acetate | P22460 | KCNA5 | 0.13 |
| Alisol B Acetate | P54753 | EPHB3 | 0.13 |
| Alisol B Acetate | P33316 | DUT | 0.13 |
| Alisol B Acetate | P00734 | F2 | 0.13 |
| Alisol B Acetate | P05093 | CYP17A1 | 0.13 |
| Alisol B Acetate | P00797 | REN | 0.13 |
| Alisol B Acetate | P04150 | NR3C1 | 0.13 |
| Alisol B Acetate | O14920 | IKBKB | 0.13 |
| Alisol B Acetate | Q99835 | SMO | 0.13 |
| Alisol B Acetate | O00408 | PDE2A | 0.13 |
| Alisol B Acetate | Q16539 | MAPK14 | 0.13 |
| Alisol B Acetate | Q99705 | MCHR1 | 0.13 |
| Alisol B Acetate | O00142 | TK2 | 0.13 |
| Alisol B Acetate | P49336 | CDK8 | 0.13 |
| Alisol B Acetate | P32245 | MC4R | 0.13 |
| Alisol B Acetate | Q01726 | MC1R | 0.13 |
| Alisol B Acetate | P33032 | MC5R | 0.13 |
| Alisol B Acetate | P41968 | MC3R | 0.13 |
| Alisol B Acetate | P23458 | JAK1 | 0.13 |
| Alisol B Acetate | Q99500 | S1PR3 | 0.13 |
| Alisol B Acetate | O14684 | PTGES | 0.13 |
| Alisol B Acetate | O75469 | NR1I2 | 0.13 |
| Alisol B Acetate | P23975 | SLC6A2 | 0.13 |
| Alisol B Acetate | Q96GD4 | AURKB | 0.13 |
| Alisol B Acetate | O14649 | KCNK3 | 0.13 |
| Alisol B Acetate | Q9NWT8 | AURKAIP1 | 0.13 |
| Alisol B Acetate | P08235 | NR3C2 | 0.13 |
| Alisol B Acetate | P24530 | EDNRB | 0.13 |
| Alisol B Acetate | O75116 | ROCK2 | 0.13 |
| Alisol B Acetate | O14757 | CHEK1 | 0.13 |
| Alisol B Acetate | O14965 | AURKA | 0.13 |
| Alisol B Acetate | P0DJD9 | PGA5 | 0.13 |
| Alisol B Acetate | P01130 | LDLR | 0.13 |
| Alisol B Acetate | P03956 | MMP1 | 0.13 |
| Alisol B Acetate | P10275 | AR | 0.13 |
| Alisol B Acetate | P00740 | F9 | 0.13 |
| Alisol B Acetate | P00742 | F10 | 0.13 |
| Alisol B Acetate | P50750 | CDK9 | 0.13 |
| Alisol B Acetate | Q00975 | CACNA1B | 0.13 |
| Alisol B Acetate | O00519 | FAAH | 0.13 |
| Alisol B Acetate | P07333 | CSF1R | 0.13 |
| Alisol B Acetate | P08069 | IGF1R | 0.13 |
| Alisol B Acetate | Q96RI1 | NR1H4 | 0.13 |
| Alisol B Acetate | P21728 | DRD1 | 0.13 |
| Alisol B Acetate | P28223 | HTR2A | 0.13 |
| Alisol B Acetate | P28335 | HTR2C | 0.13 |
| Alisol B Acetate | Q9UNA0 | ADAMTS5 | 0.13 |
| Alisol B Acetate | O75173 | ADAMTS4 | 0.13 |
| Alisol B Acetate | P48067 | SLC6A9 | 0.13 |
| Alisol B Acetate | Q12908 | SLC10A2 | 0.13 |
| Alisol B Acetate | P45452 | MMP13 | 0.13 |
| Alisol B Acetate | P32239 | CCKBR | 0.13 |
| Alisol B Acetate | P08253 | MMP2 | 0.13 |
| Alisol B Acetate | P50281 | MMP14 | 0.13 |
| Alisol B Acetate | O15294 | OGT | 0.13 |

|  | **STITCH** |  |  |
| --- | --- | --- | --- |
| **Compounds** | **UniProt** | **Target genes** | **Score** |
| 3,4-Dihydroxybenzoic acid (Protocatechuic acid)* | [Q13285](https://www.uniprot.org/uniprotkb/Q13285/entry) | NR5A1 | 0.8 |
| 3,4-Dihydroxybenzoic acid (Protocatechuic acid)* | [P23526](https://www.uniprot.org/uniprotkb/P23526/entry) | AHCY | 0.9 |
| 3,4-Dihydroxybenzoic acid (Protocatechuic acid)* | [P21964](https://www.uniprot.org/uniprotkb/P21964/entry) | COMT | 0.914 |
| 3-Hydroxybenzoic acid | [P46952](https://www.uniprot.org/uniprotkb/P46952/entry) | HAAO | 0.9 |
| 4-Hydroxybenzoic acid | [P00918](https://www.uniprot.org/uniprotkb/P00918/entry) | CA2 | 0.8 |
| 4-Hydroxybenzoic acid | [Q86YH6](https://www.uniprot.org/uniprotkb/Q86YH6/entry) | PDSS2 | 0.927 |
| 4-Hydroxybenzoic acid | [Q5T2R2](https://www.uniprot.org/uniprotkb/Q5T2R2/entry) | PDSS1 | 0.942 |
| 4-Hydroxybenzoic acid | [Q9Y2Z9](https://www.uniprot.org/uniprotkb/Q9Y2Z9/entry) | COQ6 | 0.983 |
| 4-Hydroxybenzoic acid | [Q96H96](https://www.uniprot.org/uniprotkb/Q96H96/entry) | COQ2 | 0.998 |
| Benzamide | [P17931](https://www.uniprot.org/uniprotkb/P17931/entry) | LGALS3 | 0.8 |
| Benzamide | [P43490](https://www.uniprot.org/uniprotkb/P43490/entry) | NAMPT | 0.8 |
| Benzamide | [P22748](https://www.uniprot.org/uniprotkb/P22748/entry) | CA4 | 0.845 |
| Benzoic acid | [Q9UN36](https://www.uniprot.org/uniprotkb/Q9UN36/entry) | NDRG2 | 0.812 |
| Benzoic acid | [P00918](https://www.uniprot.org/uniprotkb/P00918/entry) | CA2 | 0.815 |
| Benzoic acid | [Q6IB77](https://www.uniprot.org/uniprotkb/Q6IB77/entry) | GLYAT | 0.919 |
| Benzoic acid | [P51151](https://www.uniprot.org/uniprotkb/P51151/entry) | RAB9A | 0.946 |
| Benzoic acid | [Q08AH1](https://www.uniprot.org/uniprotkb/Q08AH1/entry) | ACSM1 | 0.952 |
| Benzoic acid | [Q68CK6](https://www.uniprot.org/uniprotkb/Q68CK6/entry) | ACSM2B | 0.958 |
| Benzoic acid | [P23141](https://www.uniprot.org/uniprotkb/P23141/entry) | CES1 | 0.958 |
| Benzoic acid | [P52758](https://www.uniprot.org/uniprotkb/P52758/entry) | RIDA | 0.958 |
| Benzoic acid | [P30044](https://www.uniprot.org/uniprotkb/P30044/entry) | PRDX5 | 0.97 |
| Benzoic acid | [P14920](https://www.uniprot.org/uniprotkb/P14920/entry) | DAO | 0.993 |
| Calycosin | [P09429](https://www.uniprot.org/uniprotkb/P09429/entry) | HMGB1 | 0.824 |
| Calycosin | [P30043](https://www.uniprot.org/uniprotkb/P30043/entry) | BLVRB | 0.845 |
| Catechin | [P56817](https://www.uniprot.org/uniprotkb/P56817/entry) | BACE1 | 0.8 |
| Catechin | [P04141](https://www.uniprot.org/uniprotkb/P04141/entry) | CSF2 | 0.8 |
| Catechin | [Q9NSA0](https://www.uniprot.org/uniprotkb/Q9NSA0/entry) | SLC22A11 | 0.8 |
| Catechin | [P27169](https://www.uniprot.org/uniprotkb/P27169/entry) | PON1 | 0.814 |
| Catechin | [P04114](https://www.uniprot.org/uniprotkb/P04114/entry) | APOB | 0.822 |
| Catechin | [Q96FL8](https://www.uniprot.org/uniprotkb/Q96FL8/entry) | SLC47A1 | 0.824 |
| Catechin | [P35354](https://www.uniprot.org/uniprotkb/P35354/entry) | PTGS2 | 0.833 |
| Catechin | [P26358](https://www.uniprot.org/uniprotkb/P26358/entry) | DNMT1 | 0.837 |
| Catechin | [P09601](https://www.uniprot.org/uniprotkb/P09601/entry) | HMOX1 | 0.855 |
| D-Galactose* | [P01236](https://www.uniprot.org/uniprotkb/P01236/entry) | PRL | 0.8 |
| D-Galactose* | [Q2TB90](https://www.uniprot.org/uniprotkb/Q2TB90/entry) | HKDC1 | 0.815 |
| D-Galactose* | [P24385](https://www.uniprot.org/uniprotkb/P24385/entry) | CCND1 | 0.818 |
| D-Galactose* | [P52790](https://www.uniprot.org/uniprotkb/P52790/entry) | HK3 | 0.831 |
| D-Galactose* | [P19367](https://www.uniprot.org/uniprotkb/P19367/entry) | HK1 | 0.836 |
| D-Galactose* | [P52789](https://www.uniprot.org/uniprotkb/P52789/entry) | HK2 | 0.836 |
| D-Galactose* | [P01275](https://www.uniprot.org/uniprotkb/P01275/entry) | GCG | 0.886 |
| Ellagic acid | [P43405](https://www.uniprot.org/uniprotkb/P43405/entry) | SYK | 0.816 |
| Ellagic acid | [P00918](https://www.uniprot.org/uniprotkb/P00918/entry) | CA2 | 0.817 |
| Ellagic acid | [P15531 P22392](https://www.uniprot.org/uniprotkb/P15531/entry) | NME1-NME2 | 0.82 |
| Ellagic acid | P22392 | NME2 | 0.82 |
| Ellagic acid | [Q14534](https://www.uniprot.org/uniprotkb/Q14534/entry) | SQLE | 0.827 |
| Ellagic acid | [P29474](https://www.uniprot.org/uniprotkb/P29474/entry) | NOS3 | 0.828 |
| Ellagic acid | [P05112](https://www.uniprot.org/uniprotkb/P05112/entry) | IL4 | 0.834 |
| Ellagic acid | [P14679](https://www.uniprot.org/uniprotkb/P14679/entry) | TYR | 0.847 |
| Ellagic acid | [P68400](https://www.uniprot.org/uniprotkb/P68400/entry) | CSNK2A1 | 0.958 |
| Ellagic acid | [P00748](https://www.uniprot.org/uniprotkb/P00748/entry) | F12 | 0.966 |
| Formononetin (7-Hydroxy-4'-methoxyisoflavone) | [P27815](https://www.uniprot.org/uniprotkb/P27815/entry) | PDE4A | 0.8 |
| Glycitein | [P45452](https://www.uniprot.org/uniprotkb/P45452/entry) | MMP13 | 0.8 |
| Homovanillic acid; 4-Hydroxy-3-methoxyphenylacetic acid | [P07101](https://www.uniprot.org/uniprotkb/P07101/entry) | TH | 0.847 |
| Homovanillic acid; 4-Hydroxy-3-methoxyphenylacetic acid | [P28223](https://www.uniprot.org/uniprotkb/P28223/entry) | HTR2A | 0.862 |
| Homovanillic acid; 4-Hydroxy-3-methoxyphenylacetic acid | [P47895](https://www.uniprot.org/uniprotkb/P47895/entry) | ALDH1A3 | 0.9 |
| Homovanillic acid; 4-Hydroxy-3-methoxyphenylacetic acid | [P30838](https://www.uniprot.org/uniprotkb/P30838/entry) | ALDH3A1 | 0.907 |
| Homovanillic acid; 4-Hydroxy-3-methoxyphenylacetic acid | [P48448](https://www.uniprot.org/uniprotkb/P48448/entry) | ALDH3B2 | 0.908 |
| Homovanillic acid; 4-Hydroxy-3-methoxyphenylacetic acid | [P21964](https://www.uniprot.org/uniprotkb/P21964/entry) | COMT | 0.947 |
| Hydroquinone | [P00918](https://www.uniprot.org/uniprotkb/P00918/entry) | CA2 | 0.8 |
| Hydroquinone | [P27169](https://www.uniprot.org/uniprotkb/P27169/entry) | PON1 | 0.8 |
| Hydroquinone | [Q15165](https://www.uniprot.org/uniprotkb/Q15165/entry) | PON2 | 0.8 |
| Hydroquinone | [Q15166](https://www.uniprot.org/uniprotkb/Q15166/entry) | PON3 | 0.8 |
| Hydroquinone | [P04040](https://www.uniprot.org/uniprotkb/P04040/entry) | CAT | 0.824 |
| Hydroquinone | [P14679](https://www.uniprot.org/uniprotkb/P14679/entry) | TYR | 0.904 |
| Hydroquinone | [P15559](https://www.uniprot.org/uniprotkb/P15559/entry) | NQO1 | 0.914 |
| Hydroquinone | [P00156](https://www.uniprot.org/uniprotkb/P00156/entry) | MT-CYB | 0.922 |
| Kaempferol-3-O-galactoside (Trifolin)* | [P35354](https://www.uniprot.org/uniprotkb/P35354/entry) | PTGS2 | 0.8 |
| Kaempferol-3-O-glucoside (Astragalin)* | [P35354](https://www.uniprot.org/uniprotkb/P35354/entry) | PTGS2 | 0.8 |
| Methyl 4-hydroxybenzoate | [F8WCM5](https://www.uniprot.org/uniprotkb/F8WCM5/entry) | INS-IGF2 | 0.8 |
| Nobiletin (5,6,7,8,3',4'-Hexamethoxyflavone)* | [P33527](https://www.uniprot.org/uniprotkb/P33527/entry) | ABCC1 | 0.8 |
| Nobiletin (5,6,7,8,3',4'-Hexamethoxyflavone)* | [P13569](https://www.uniprot.org/uniprotkb/P13569/entry) | CFTR | 0.8 |
| Nobiletin (5,6,7,8,3',4'-Hexamethoxyflavone)* | [O75907](https://www.uniprot.org/uniprotkb/O75907/entry) | DGAT1 | 0.8 |
| Nobiletin (5,6,7,8,3',4'-Hexamethoxyflavone)* | [P08473](https://www.uniprot.org/uniprotkb/P08473/entry) | MME | 0.8 |
| Nobiletin (5,6,7,8,3',4'-Hexamethoxyflavone)* | [P08253](https://www.uniprot.org/uniprotkb/P08253/entry) | MMP2 | 0.8 |
| Nobiletin (5,6,7,8,3',4'-Hexamethoxyflavone)* | [P01106](https://www.uniprot.org/uniprotkb/P01106/entry) | MYC | 0.8 |
| Nobiletin (5,6,7,8,3',4'-Hexamethoxyflavone)* | [P15692](https://www.uniprot.org/uniprotkb/P15692/entry) | VEGFA | 0.8 |
| Nobiletin (5,6,7,8,3',4'-Hexamethoxyflavone)* | [P14780](https://www.uniprot.org/uniprotkb/P14780/entry) | MMP9 | 0.818 |
| Nobiletin (5,6,7,8,3',4'-Hexamethoxyflavone)* | [P11511](https://www.uniprot.org/uniprotkb/P11511/entry) | CYP1A1 | 0.943 |
| Procyanidin B1/B2 | [Q96P20](https://www.uniprot.org/uniprotkb/Q96P20/entry) | NLRP3 | 0.8 |
| Quercetin | [Q6NVY1](https://www.uniprot.org/uniprotkb/Q6NVY1/entry) | HIBCH | 0.958 |
| Quercetin | [O94768](https://www.uniprot.org/uniprotkb/O94768/entry) | STK17B | 0.958 |
| Quercetin | [P06576](https://www.uniprot.org/uniprotkb/P06576/entry) | ATP5B | 0.961 |
| Quercetin | [P11511](https://www.uniprot.org/uniprotkb/P11511/entry) | CYP1A1 | 0.963 |
| Quercetin | [P10632](https://www.uniprot.org/uniprotkb/P10632/entry) | CYP2C8 | 0.964 |
| Quercetin | [P11168](https://www.uniprot.org/uniprotkb/P11168/entry) | SLC2A2 | 0.965 |
| Quercetin | [P08631](https://www.uniprot.org/uniprotkb/P08631/entry) | HCK | 0.969 |
| Quercetin | [P11309](https://www.uniprot.org/uniprotkb/P11309/entry) | PIM1 | 0.969 |
| Quercetin | [Q16678](https://www.uniprot.org/uniprotkb/Q16678/entry) | CYP1B1 | 0.975 |
| Quercetin | [Q07820](https://www.uniprot.org/uniprotkb/Q07820/entry) | MCL1 | 0.987 |
| Quercetin-3-O-glucoside (Isoquercitrin)* | [P42574](https://www.uniprot.org/uniprotkb/P42574/entry) | CASP3 | 0.8 |
| Quercetin-3-O-rhamnoside(Quercitrin) | [P08684](https://www.uniprot.org/uniprotkb/P08684/entry) | CYP3A4 | 0.8 |
| Quercetin-3-O-rutinoside (Rutin) | [P05091](https://www.uniprot.org/uniprotkb/P05091/entry) | ALDH2 | 0.8 |
| Quercetin-3-O-rutinoside (Rutin) | [P29279](https://www.uniprot.org/uniprotkb/P29279/entry) | CTGF | 0.8 |
| Quercetin-3-O-rutinoside (Rutin) | [P00533](https://www.uniprot.org/uniprotkb/P00533/entry) | EGFR | 0.8 |
| Quercetin-3-O-rutinoside (Rutin) | [P04156](https://www.uniprot.org/uniprotkb/P04156/entry) | PRNP | 0.8 |
| Quercetin-3-O-rutinoside (Rutin) | [P36956](https://www.uniprot.org/uniprotkb/P36956/entry) | SREBF1 | 0.8 |
| Quercetin-3-O-rutinoside (Rutin) | [P07237](https://www.uniprot.org/uniprotkb/P07237/entry) | P4HB | 0.841 |
| Quercetin-3-O-rutinoside (Rutin) | [P42330](https://www.uniprot.org/uniprotkb/P42330/entry) | AKR1C3 | 0.962 |
| Riboflavin (Vitamin B2) | [P00390](https://www.uniprot.org/uniprotkb/P00390/entry) | GSR | 0.893 |
| Riboflavin (Vitamin B2) | [P30043](https://www.uniprot.org/uniprotkb/P30043/entry) | BLVRB | 0.9 |
| Riboflavin (Vitamin B2) | [Q969G6](https://www.uniprot.org/uniprotkb/Q969G6/entry) | RFK | 0.9 |
| Salidroside | [Q16665](https://www.uniprot.org/uniprotkb/Q16665/entry) | HIF1A | 0.8 |
| Salidroside | [P22301](https://www.uniprot.org/uniprotkb/P22301/entry) | IL10 | 0.8 |
| Salidroside | [P31749](https://www.uniprot.org/uniprotkb/P31749/entry) | AKT1 | 0.815 |
| Salidroside | [P42574](https://www.uniprot.org/uniprotkb/P42574/entry) | CASP3 | 0.822 |
| Syringic acid | [O15467](https://www.uniprot.org/uniprotkb/O15467/entry) | CCL16 | 0.8 |
| Uridine | [P08908](https://www.uniprot.org/uniprotkb/P08908/entry) | HTR1A | 0.8 |
| Uridine | [P28223](https://www.uniprot.org/uniprotkb/P28223/entry) | HTR2A | 0.8 |
| Vanillin; 4-Hydroxy-3-Methoxybenzaldehyde | [O14649](https://www.uniprot.org/uniprotkb/O14649/entry) | KCNK3 | 0.8 |
| Vanillin; 4-Hydroxy-3-Methoxybenzaldehyde | [P14780](https://www.uniprot.org/uniprotkb/P14780/entry) | MMP9 | 0.8 |
| Vanillin; 4-Hydroxy-3-Methoxybenzaldehyde | [Q8NET8](https://www.uniprot.org/uniprotkb/Q8NET8/entry) | TRPV3 | 0.818 |
| Wogonin (5,7-Dihydroxy-8-Methoxyflavone) | [P13500](https://www.uniprot.org/uniprotkb/P13500/entry) | CCL2 | 0.8 |
| Wogonin (5,7-Dihydroxy-8-Methoxyflavone) | [P15976](https://www.uniprot.org/uniprotkb/P15976/entry) | GATA1 | 0.8 |
| Wogonin (5,7-Dihydroxy-8-Methoxyflavone) | [P09429](https://www.uniprot.org/uniprotkb/P09429/entry) | HMGB1 | 0.8 |
| Wogonin (5,7-Dihydroxy-8-Methoxyflavone) | [P57789](https://www.uniprot.org/uniprotkb/P57789/entry) | KCNK10 | 0.8 |
| Wogonin (5,7-Dihydroxy-8-Methoxyflavone) | [P01106](https://www.uniprot.org/uniprotkb/P01106/entry) | MYC | 0.8 |
| Wogonin (5,7-Dihydroxy-8-Methoxyflavone) | [O15162](https://www.uniprot.org/uniprotkb/O15162/entry) | PLSCR1 | 0.8 |
| Wogonin (5,7-Dihydroxy-8-Methoxyflavone) | [Q07820](https://www.uniprot.org/uniprotkb/Q07820/entry) | MCL1 | 0.803 |
| Wogonin (5,7-Dihydroxy-8-Methoxyflavone) | [P50750](https://www.uniprot.org/uniprotkb/P50750/entry) | CDK9 | 0.82 |
| Wogonin (5,7-Dihydroxy-8-Methoxyflavone) | [P14780](https://www.uniprot.org/uniprotkb/P14780/entry) | MMP9 | 0.84 |
| Wogonin (5,7-Dihydroxy-8-Methoxyflavone) | [P35354](https://www.uniprot.org/uniprotkb/P35354/entry) | PTGS2 | 0.861 |
