# Supplementary material for: ShenQi DiHuang Decoction (SQDHD) Ameliorates Neuroinflammation and Neuropsychiatric Manifestations in Pristane Induced Lupus Mice via Blocking JAK1‐STAT3 Pathway
Source: CNS Neurosci Ther. 2026 Mar 7;32(3):e70814. doi: 10.1002/cns.70814 (PMC12967629; doi:10.1002/cns.70814)
Supplement: Supplementary file 5 — Table S5: Collection of overlapping target genes between SQDHD and NPSLE. [file CNS-32-e70814-s005.docx]

**TableS5：Collection of overlapping target genes between SQDHD and NPSLE**

| **NPSLE associated target genes** | | | **SQDHD associated target genes** | | | **Overlapping target genes** | | |
| --- | --- | --- | --- | --- | --- | --- | --- | --- |
|  | AARS1 |  |  | ABCB1 |  |  | ABCB1 |  |
|  | ABAT |  |  | ABCC1 |  |  | ACHE |  |
|  | ABCA7 |  |  | ABCG2 |  |  | ACP1 |  |
|  | ABCB1 |  |  | ACHE |  |  | ADA |  |
|  | ABCB11 |  |  | ACP1 |  |  | ADORA1 |  |
|  | ABHD3 |  |  | ACSM1 |  |  | AGTR1 |  |
|  | ABI2 |  |  | ACSM2B |  |  | AKT1 |  |
|  | ACADS |  |  | ADA |  |  | ALDH2 |  |
|  | ACADVL |  |  | ADAMTS4 |  |  | ALDH3A1 |  |
|  | ACE |  |  | ADAMTS5 |  |  | ALOX5AP |  |
|  | ACE2 |  |  | ADORA1 |  |  | APP |  |
|  | ACHE |  |  | ADORA2A |  |  | ARG1 |  |
|  | ACKR3 |  |  | ADORA2B |  |  | AXL |  |
|  | ACOD1 |  |  | ADORA3 |  |  | BCHE |  |
|  | ACP1 |  |  | ADRA1A |  |  | BCL2L1 |  |
|  | ADA |  |  | ADRA1D |  |  | BLVRB |  |
|  | ADAMTS9 |  |  | ADRA2A |  |  | CA12 |  |
|  | ADCY5 |  |  | ADRA2B |  |  | CA2 |  |
|  | ADCYAP1 |  |  | ADRA2C |  |  | CASP3 |  |
|  | ADCYAP1R1 |  |  | AGTR1 |  |  | CAT |  |
|  | ADIPOQ |  |  | AHCY |  |  | CCL2 |  |
|  | ADORA1 |  |  | AHR |  |  | CD38 |  |
|  | ADRB2 |  |  | AKR1A1 |  |  | CD81 |  |
|  | ADSL |  |  | AKR1B1 |  |  | CDK1 |  |
|  | AGL |  |  | AKR1B10 |  |  | CDK5 |  |
|  | AGT |  |  | AKR1C1 |  |  | CHRM2 |  |
|  | AGTR1 |  |  | AKR1C2 |  |  | CNR1 |  |
|  | AHI1 |  |  | AKR1C3 |  |  | COMT |  |
|  | AIF1 |  |  | AKR1C4 |  |  | COQ2 |  |
|  | AKAP8 |  |  | AKT1 |  |  | CSF1R |  |
|  | AKT1 |  |  | ALDH1A3 |  |  | CSF2 |  |
|  | ALAD |  |  | ALDH2 |  |  | CSNK2A1 |  |
|  | ALB |  |  | ALDH3A1 |  |  | CTSD |  |
|  | ALCAM |  |  | ALDH3B2 |  |  | CYP19A1 |  |
|  | ALDH2 |  |  | ALK |  |  | CYP2C19 |  |
|  | ALDH3A1 |  |  | ALOX12 |  |  | CYP2D6 |  |
|  | ALDH5A1 |  |  | ALOX15 |  |  | DNMT1 |  |
|  | ALOX5AP |  |  | ALOX5 |  |  | DRD1 |  |
|  | ALYREF |  |  | ALOX5AP |  |  | DRD2 |  |
|  | AMFR |  |  | AMPD2 |  |  | DRD4 |  |
|  | AMH |  |  | AMY1A |  |  | EGFR |  |
|  | AMPH |  |  | AMY2A |  |  | ESR1 |  |
|  | ANGPT1 |  |  | APEX1 |  |  | ESR2 |  |
|  | ANGPT2 |  |  | APH1A |  |  | ESRRA |  |
|  | ANK3 |  |  | APH1B |  |  | F2 |  |
|  | ANXA11 |  |  | APOB |  |  | F2RL1 |  |
|  | APAF1 |  |  | APP |  |  | FABP4 |  |
|  | APC |  |  | AR |  |  | FGF2 |  |
|  | APOA1 |  |  | ARG1 |  |  | FLT3 |  |
|  | APOE |  |  | ATP5B |  |  | FYN |  |
|  | APOH |  |  | AURKA |  |  | G6PD |  |
|  | APP |  |  | AURKAIP1 |  |  | GBA1 |  |
|  | AQP4 |  |  | AURKB |  |  | GLUL |  |
|  | ARG1 |  |  | AVPR1A |  |  | GRIK2 |  |
|  | ARHGDIB |  |  | AVPR2 |  |  | GSK3B |  |
|  | ARHGEF26 |  |  | AXL |  |  | GSR |  |
|  | ARX |  |  | BACE1 |  |  | HMGCR |  |
|  | ASS1 |  |  | BACE2 |  |  | HMOX1 |  |
|  | ATF2 |  |  | BCHE |  |  | HSP90AA1 |  |
|  | ATF5 |  |  | BCL2L1 |  |  | HSPA1A |  |
|  | ATG16L1 |  |  | BLVRB |  |  | HTR1A |  |
|  | ATG3 |  |  | BRAF |  |  | HTR2A |  |
|  | ATG5 |  |  | C5AR1 |  |  | IDO1 |  |
|  | ATG7 |  |  | CA1 |  |  | IGF1R |  |
|  | ATL1 |  |  | CA12 |  |  | IL10 |  |
|  | ATN1 |  |  | CA13 |  |  | IL2 |  |
|  | ATP13A2 |  |  | CA14 |  |  | IL4 |  |
|  | ATP1A2 |  |  | CA2 |  |  | IL6 |  |
|  | ATP1A3 |  |  | CA3 |  |  | JAK1 |  |
|  | ATP7B |  |  | CA4 |  |  | KDM2A |  |
|  | ATRIP |  |  | CA5A |  |  | KDM6B |  |
|  | ATRX |  |  | CA5B |  |  | KIT |  |
|  | ATXN1 |  |  | CA6 |  |  | LCK |  |
|  | ATXN10 |  |  | CA7 |  |  | LDLR |  |
|  | ATXN2 |  |  | CA9 |  |  | MAOA |  |
|  | ATXN3 |  |  | CACNA1B |  |  | MAOB |  |
|  | ATXN7 |  |  | CAMK2B |  |  | MAPK1 |  |
|  | AXL |  |  | CASP3 |  |  | MAPK14 |  |
|  | B3GALNT2 |  |  | CAT |  |  | MAPT |  |
|  | BAG1 |  |  | CBR1 |  |  | MDM2 |  |
|  | BAX |  |  | CCKBR |  |  | MIF |  |
|  | BBS4 |  |  | CCL16 |  |  | MMP8 |  |
|  | BCHE |  |  | CCL2 |  |  | MMP9 |  |
|  | BCL2 |  |  | CCNA1 |  |  | MPG |  |
|  | BCL2L1 |  |  | CCNA2 |  |  | MT-CYB |  |
|  | BCL2L2 |  |  | CCNB1 |  |  | MYC |  |
|  | BCL6 |  |  | CCNB2 |  |  | NAMPT |  |
|  | BDH1 |  |  | CCNB3 |  |  | NCSTN |  |
|  | BDKRB1 |  |  | CCNC |  |  | NME1 |  |
|  | BDKRB2 |  |  | CCND1 |  |  | NOS2 |  |
|  | BDNF |  |  | CCR1 |  |  | NOS3 |  |
|  | BECN1 |  |  | CD38 |  |  | NR1H4 |  |
|  | BIK |  |  | CD81 |  |  | NR3C1 |  |
|  | BIN1 |  |  | CDA |  |  | NR3C2 |  |
|  | BLVRB |  |  | CDC25A |  |  | NR5A1 |  |
|  | BPI |  |  | CDC25B |  |  | NTRK2 |  |
|  | BRPF1 |  |  | CDK1 |  |  | OPRM1 |  |
|  | BSCL2 |  |  | CDK2 |  |  | P2RX3 |  |
|  | BSG |  |  | CDK4 |  |  | PARP1 |  |
|  | BTG1 |  |  | CDK5 |  |  | PDE4A |  |
|  | BTG3 |  |  | CDK5R1 |  |  | PDE5A |  |
|  | BUB3 |  |  | CDK6 |  |  | PDGFRB |  |
|  | C1QA |  |  | CDK8 |  |  | PER2 |  |
|  | C1QC |  |  | CDK9 |  |  | PGR |  |
|  | C1R |  |  | CES1 |  |  | PIM1 |  |
|  | C3 |  |  | CES2 |  |  | PLA2G2A |  |
|  | C4A |  |  | CFTR |  |  | PON1 |  |
|  | C4B |  |  | CHEK1 |  |  | PPARG |  |
|  | C9orf72 |  |  | CHRM2 |  |  | PRKCD |  |
|  | CA12 |  |  | CHUK |  |  | PRKCG |  |
|  | CA2 |  |  | CNR1 |  |  | PRL |  |
|  | CABP1 |  |  | CNR2 |  |  | PRNP |  |
|  | CABP2 |  |  | COMT |  |  | PTGER4 |  |
|  | CACNA1A |  |  | COQ2 |  |  | PTGS2 |  |
|  | CACNA1G |  |  | COQ6 |  |  | PTPN11 |  |
|  | CACNA1H |  |  | CSF1R |  |  | RORC |  |
|  | CALB1 |  |  | CSF2 |  |  | SHBG |  |
|  | CALB2 |  |  | CSNK2A1 |  |  | SLC6A3 |  |
|  | CALR |  |  | CTGF |  |  | SLC6A4 |  |
|  | CAMK2G |  |  | CTRC |  |  | STAT3 |  |
|  | CAMK4 |  |  | CTSD |  |  | SYK |  |
|  | CANX |  |  | CXCR1 |  |  | TACR1 |  |
|  | CAPN2 |  |  | CYP17A1 |  |  | TERT |  |
|  | CARS1 |  |  | CYP19A1 |  |  | TH |  |
|  | CASP1 |  |  | CYP1A1 |  |  | TNF |  |
|  | CASP3 |  |  | CYP1B1 |  |  | TTR |  |
|  | CASP8 |  |  | CYP2C19 |  |  | VDR |  |
|  | CASP9 |  |  | CYP2C8 |  |  | VEGFA |  |
|  | CAT |  |  | CYP2D6 |  |  | XDH |  |
|  | CBL |  |  | CYP3A4 |  |  |  |  |
|  | CBS |  |  | CYP51A1 |  |  |  |  |
|  | CCIN |  |  | CYSLTR1 |  |  |  |  |
|  | CCK |  |  | DAO |  |  |  |  |
|  | CCL17 |  |  | DAPK1 |  |  |  |  |
|  | CCL19 |  |  | DGAT1 |  |  |  |  |
|  | CCL2 |  |  | DNMT1 |  |  |  |  |
|  | CCL21 |  |  | DRD1 |  |  |  |  |
|  | CCL22 |  |  | DRD2 |  |  |  |  |
|  | CCL25 |  |  | DRD3 |  |  |  |  |
|  | CCL3 |  |  | DRD4 |  |  |  |  |
|  | CCL5 |  |  | DUT |  |  |  |  |
|  | CCL8 |  |  | EDNRB |  |  |  |  |
|  | CCND3 |  |  | EGFR |  |  |  |  |
|  | CCR5 |  |  | EPAS1 |  |  |  |  |
|  | CCR7 |  |  | EPHB3 |  |  |  |  |
|  | CCR8 |  |  | EPHB4 |  |  |  |  |
|  | CCR9 |  |  | ERBB2 |  |  |  |  |
|  | CCT3 |  |  | ERN1 |  |  |  |  |
|  | CCT5 |  |  | ESR1 |  |  |  |  |
|  | CCT7 |  |  | ESR2 |  |  |  |  |
|  | CD151 |  |  | ESRRA |  |  |  |  |
|  | CD2 |  |  | ESRRB |  |  |  |  |
|  | CD226 |  |  | F10 |  |  |  |  |
|  | CD244 |  |  | F12 |  |  |  |  |
|  | CD27 |  |  | F2 |  |  |  |  |
|  | CD28 |  |  | F2R |  |  |  |  |
|  | CD38 |  |  | F2RL1 |  |  |  |  |
|  | CD3D |  |  | F9 |  |  |  |  |
|  | CD3E |  |  | FAAH |  |  |  |  |
|  | CD3G |  |  | FABP1 |  |  |  |  |
|  | CD4 |  |  | FABP2 |  |  |  |  |
|  | CD47 |  |  | FABP3 |  |  |  |  |
|  | CD5 |  |  | FABP4 |  |  |  |  |
|  | CD6 |  |  | FABP5 |  |  |  |  |
|  | CD69 |  |  | FBP1 |  |  |  |  |
|  | CD79A |  |  | FDFT1 |  |  |  |  |
|  | CD79B |  |  | FFAR1 |  |  |  |  |
|  | CD81 |  |  | FGF1 |  |  |  |  |
|  | CD83 |  |  | FGF2 |  |  |  |  |
|  | CD84 |  |  | FGR |  |  |  |  |
|  | CD8A |  |  | FLT3 |  |  |  |  |
|  | CD8B |  |  | FLT4 |  |  |  |  |
|  | CDH2 |  |  | FNTA |  |  |  |  |
|  | CDH23 |  |  | FNTB |  |  |  |  |
|  | CDH3 |  |  | FTO |  |  |  |  |
|  | CDK1 |  |  | FUT7 |  |  |  |  |
|  | CDK5 |  |  | FYN |  |  |  |  |
|  | CDKL5 |  |  | G6PD |  |  |  |  |
|  | CDKN2A |  |  | GATA1 |  |  |  |  |
|  | CDKN2D |  |  | GBA1 |  |  |  |  |
|  | CEL |  |  | GCG |  |  |  |  |
|  | CFB |  |  | GLO1 |  |  |  |  |
|  | CFI |  |  | GLRA1 |  |  |  |  |
|  | CHAMP1 |  |  | GLRA2 |  |  |  |  |
|  | CHD4 |  |  | GLUL |  |  |  |  |
|  | CHD5 |  |  | GLYAT |  |  |  |  |
|  | CHGA |  |  | GPBAR1 |  |  |  |  |
|  | CHKA |  |  | GPR35 |  |  |  |  |
|  | CHRM2 |  |  | GRIK1 |  |  |  |  |
|  | CHRM5 |  |  | GRIK2 |  |  |  |  |
|  | CHRNA4 |  |  | GSK3B |  |  |  |  |
|  | CITED2 |  |  | GSR |  |  |  |  |
|  | CKM |  |  | HAAO |  |  |  |  |
|  | CLDN4 |  |  | HCAR2 |  |  |  |  |
|  | CLIC4 |  |  | HCK |  |  |  |  |
|  | CLIP1 |  |  | HDAC8 |  |  |  |  |
|  | CLN3 |  |  | HIBCH |  |  |  |  |
|  | CLU |  |  | HIF1A |  |  |  |  |
|  | CNBP |  |  | HK1 |  |  |  |  |
|  | CNR1 |  |  | HK2 |  |  |  |  |
|  | CNTF |  |  | HK3 |  |  |  |  |
|  | CNTN1 |  |  | HKDC1 |  |  |  |  |
|  | CNTN6 |  |  | HMGB1 |  |  |  |  |
|  | CNTNAP2 |  |  | HMGCR |  |  |  |  |
|  | COCH |  |  | HMOX1 |  |  |  |  |
|  | COL5A2 |  |  | HPSE |  |  |  |  |
|  | COMT |  |  | RIDA |  |  |  |  |
|  | COQ2 |  |  | HSD11B1 |  |  |  |  |
|  | COX5A |  |  | HSD11B2 |  |  |  |  |
|  | COX7A2 |  |  | HSD17B1 |  |  |  |  |
|  | CP |  |  | HSD17B2 |  |  |  |  |
|  | CPOX |  |  | HSD17B3 |  |  |  |  |
|  | CPT2 |  |  | HSP90AA1 |  |  |  |  |
|  | CRABP2 |  |  | HSPA1A |  |  |  |  |
|  | CREB1 |  |  | HTR1A |  |  |  |  |
|  | CREBBP |  |  | HTR1B |  |  |  |  |
|  | CRH |  |  | HTR2A |  |  |  |  |
|  | CRHR1 |  |  | HTR2B |  |  |  |  |
|  | CRHR2 |  |  | HTR2C |  |  |  |  |
|  | CRP |  |  | HTR6 |  |  |  |  |
|  | CSF1 |  |  | HTR7 |  |  |  |  |
|  | CSF1R |  |  | IDO1 |  |  |  |  |
|  | CSF2 |  |  | IGF1R |  |  |  |  |
|  | CSK |  |  | IKBKB |  |  |  |  |
|  | CSMD1 |  |  | IL10 |  |  |  |  |
|  | CSNK2A1 |  |  | IL2 |  |  |  |  |
|  | CST3 |  |  | IL4 |  |  |  |  |
|  | CST7 |  |  | IL6 |  |  |  |  |
|  | CSTA |  |  | IMPDH1 |  |  |  |  |
|  | CTNNB1 |  |  | INS-IGF2 |  |  |  |  |
|  | CTNNBIP1 |  |  | INSR |  |  |  |  |
|  | CTNND2 |  |  | JAK1 |  |  |  |  |
|  | CTSB |  |  | JAK3 |  |  |  |  |
|  | CTSD |  |  | KCNA3 |  |  |  |  |
|  | CTSH |  |  | KCNA5 |  |  |  |  |
|  | CX3CL1 |  |  | KCNK10 |  |  |  |  |
|  | CX3CR1 |  |  | KCNK3 |  |  |  |  |
|  | CXCL10 |  |  | KDM2A |  |  |  |  |
|  | CXCL11 |  |  | KDM3A |  |  |  |  |
|  | CXCL13 |  |  | KDM4A |  |  |  |  |
|  | CXCL3 |  |  | KDM4C |  |  |  |  |
|  | CXCL8 |  |  | KDM4E |  |  |  |  |
|  | CXCL9 |  |  | KDM6B |  |  |  |  |
|  | CXCR3 |  |  | KDR |  |  |  |  |
|  | CXCR4 |  |  | KIT |  |  |  |  |
|  | CXCR5 |  |  | LCK |  |  |  |  |
|  | CXCR6 |  |  | LDLR |  |  |  |  |
|  | CYBB |  |  | LGALS3 |  |  |  |  |
|  | CYCS |  |  | LGALS4 |  |  |  |  |
|  | CYP19A1 |  |  | LGALS8 |  |  |  |  |
|  | CYP1A2 |  |  | LTB4R |  |  |  |  |
|  | CYP21A2 |  |  | LYN |  |  |  |  |
|  | CYP2B6 |  |  | MAOA |  |  |  |  |
|  | CYP2C18 |  |  | MAOB |  |  |  |  |
|  | CYP2C19 |  |  | MAP2K1 |  |  |  |  |
|  | CYP2C9 |  |  | MAP3K14 |  |  |  |  |
|  | CYP2D6 |  |  | MAP3K8 |  |  |  |  |
|  | CYP2E1 |  |  | MAPK1 |  |  |  |  |
|  | DBH |  |  | MAPK14 |  |  |  |  |
|  | DCAF17 |  |  | MAPT |  |  |  |  |
|  | DCN |  |  | MC1R |  |  |  |  |
|  | DCX |  |  | MC3R |  |  |  |  |
|  | DDAH2 |  |  | MC4R |  |  |  |  |
|  | DDB1 |  |  | MC5R |  |  |  |  |
|  | DDC |  |  | MCHR1 |  |  |  |  |
|  | DDIT3 |  |  | MCL1 |  |  |  |  |
|  | DEAF1 |  |  | MDM2 |  |  |  |  |
|  | DEFB1 |  |  | MET |  |  |  |  |
|  | DEK |  |  | METAP2 |  |  |  |  |
|  | DEPDC5 |  |  | MGAM |  |  |  |  |
|  | DGUOK |  |  | MGMT |  |  |  |  |
|  | DHCR7 |  |  | MIF |  |  |  |  |
|  | DHFR |  |  | MME |  |  |  |  |
|  | DKK1 |  |  | MMP1 |  |  |  |  |
|  | DLC1 |  |  | MMP12 |  |  |  |  |
|  | DLG4 |  |  | MMP13 |  |  |  |  |
|  | DMD |  |  | MMP14 |  |  |  |  |
|  | DNAJB4 |  |  | MMP2 |  |  |  |  |
|  | DNAJB6 |  |  | MMP3 |  |  |  |  |
|  | DNASE1 |  |  | MMP7 |  |  |  |  |
|  | DNASE1L3 |  |  | MMP8 |  |  |  |  |
|  | DNM1 |  |  | MMP9 |  |  |  |  |
|  | DNM1L |  |  | MPG |  |  |  |  |
|  | DNMT1 |  |  | MPO |  |  |  |  |
|  | DNMT3A |  |  | MT-CYB |  |  |  |  |
|  | DNMT3B |  |  | MYC |  |  |  |  |
|  | DNTT |  |  | MYLK |  |  |  |  |
|  | DOCK8 |  |  | NAMPT |  |  |  |  |
|  | DPF3 |  |  | NCSTN |  |  |  |  |
|  | DPP4 |  |  | NDRG2 |  |  |  |  |
|  | DPYSL4 |  |  | NEK2 |  |  |  |  |
|  | DRD1 |  |  | NEK6 |  |  |  |  |
|  | DRD2 |  |  | NLRP3 |  |  |  |  |
|  | DRD4 |  |  | NME1 |  |  |  |  |
|  | DSG1 |  |  | NME2 |  |  |  |  |
|  | DSG3 |  |  | NMUR2 |  |  |  |  |
|  | DSP |  |  | NOS2 |  |  |  |  |
|  | DUSP10 |  |  | NOS3 |  |  |  |  |
|  | DUSP2 |  |  | NOX4 |  |  |  |  |
|  | DYNC1H1 |  |  | NPC1L1 |  |  |  |  |
|  | DYRK1A |  |  | NQO1 |  |  |  |  |
|  | DZIP1 |  |  | NQO2 |  |  |  |  |
|  | E2F3 |  |  | NR1H3 |  |  |  |  |
|  | EDA2R |  |  | NR1H4 |  |  |  |  |
|  | EEA1 |  |  | NR1I2 |  |  |  |  |
|  | EGFR |  |  | NR3C1 |  |  |  |  |
|  | EGR4 |  |  | NR3C2 |  |  |  |  |
|  | EIF1AX |  |  | NR5A1 |  |  |  |  |
|  | EIF2AK2 |  |  | NTRK2 |  |  |  |  |
|  | EIF2AK3 |  |  | NUAK1 |  |  |  |  |
|  | EIF2B4 |  |  | OGT |  |  |  |  |
|  | EIF4G1 |  |  | OPRD1 |  |  |  |  |
|  | ELN |  |  | OPRK1 |  |  |  |  |
|  | ELOVL2 |  |  | OPRM1 |  |  |  |  |
|  | EMP1 |  |  | P2RX3 |  |  |  |  |
|  | EMP3 |  |  | P4HB |  |  |  |  |
|  | ENO2 |  |  | PARP1 |  |  |  |  |
|  | EPB41L2 |  |  | PDE10A |  |  |  |  |
|  | EPHA3 |  |  | PDE2A |  |  |  |  |
|  | EPO |  |  | PDE4A |  |  |  |  |
|  | ERAP1 |  |  | PDE4D |  |  |  |  |
|  | ERBB3 |  |  | PDE5A |  |  |  |  |
|  | ERBB4 |  |  | PDGFRA |  |  |  |  |
|  | ERCC1 |  |  | PDGFRB |  |  |  |  |
|  | ERCC2 |  |  | PDSS1 |  |  |  |  |
|  | ERCC3 |  |  | PDSS2 |  |  |  |  |
|  | ERCC5 |  |  | PER2 |  |  |  |  |
|  | ERG |  |  | PFKFB3 |  |  |  |  |
|  | ERVW-1 |  |  | PGA5 |  |  |  |  |
|  | ESR1 |  |  | PGF |  |  |  |  |
|  | ESR2 |  |  | PGGT1B |  |  |  |  |
|  | ESRRA |  |  | PGR |  |  |  |  |
|  | EVI2A |  |  | PIK3CB |  |  |  |  |
|  | EYS |  |  | PIK3CD |  |  |  |  |
|  | F2 |  |  | PIK3CG |  |  |  |  |
|  | F2RL1 |  |  | PIK3R1 |  |  |  |  |
|  | F2RL2 |  |  | PIM1 |  |  |  |  |
|  | F2RL3 |  |  | PKN1 |  |  |  |  |
|  | F3 |  |  | PLA2G1B |  |  |  |  |
|  | FABP4 |  |  | PLA2G2A |  |  |  |  |
|  | FAH |  |  | PLK1 |  |  |  |  |
|  | FAM107B |  |  | PLK4 |  |  |  |  |
|  | FAM234B |  |  | PLSCR1 |  |  |  |  |
|  | FAS |  |  | POLB |  |  |  |  |
|  | FASLG |  |  | PON1 |  |  |  |  |
|  | FAU |  |  | PON2 |  |  |  |  |
|  | FBL |  |  | PON3 |  |  |  |  |
|  | FBN1 |  |  | PPARA |  |  |  |  |
|  | FBXO40 |  |  | PPARD |  |  |  |  |
|  | FBXW7 |  |  | PPARG |  |  |  |  |
|  | FCER1G |  |  | PPM1A |  |  |  |  |
|  | FCGR2A |  |  | PRDX5 |  |  |  |  |
|  | FCGR3A |  |  | PREP |  |  |  |  |
|  | FCGR3B |  |  | PRKCA |  |  |  |  |
|  | FCGRT |  |  | PRKCB |  |  |  |  |
|  | FGB |  |  | PRKCD |  |  |  |  |
|  | FGF2 |  |  | PRKCE |  |  |  |  |
|  | FGF8 |  |  | PRKCG |  |  |  |  |
|  | FGFR1 |  |  | PRKCH |  |  |  |  |
|  | FHL1 |  |  | PRKCQ |  |  |  |  |
|  | FKBP1B |  |  | PRL |  |  |  |  |
|  | FKBP5 |  |  | PRNP |  |  |  |  |
|  | FKBPL |  |  | PRSS1 |  |  |  |  |
|  | FLNA |  |  | PSEN1 |  |  |  |  |
|  | FLNC |  |  | PSEN2 |  |  |  |  |
|  | FLT1 |  |  | PSENEN |  |  |  |  |
|  | FLT3 |  |  | PTAFR |  |  |  |  |
|  | FLVCR1 |  |  | PTGDR2 |  |  |  |  |
|  | FMR1 |  |  | PTGER2 |  |  |  |  |
|  | FNDC3B |  |  | PTGER4 |  |  |  |  |
|  | FOS |  |  | PTGES |  |  |  |  |
|  | FOXG1 |  |  | PTGIR |  |  |  |  |
|  | FOXO1 |  |  | PTGS1 |  |  |  |  |
|  | FOXP2 |  |  | PTGS2 |  |  |  |  |
|  | FOXP3 |  |  | PTK2 |  |  |  |  |
|  | FOXP4 |  |  | PTPN1 |  |  |  |  |
|  | FPR2 |  |  | PTPN11 |  |  |  |  |
|  | FSCN1 |  |  | PTPN2 |  |  |  |  |
|  | FSHR |  |  | PTPN6 |  |  |  |  |
|  | FTL |  |  | PTPRF |  |  |  |  |
|  | FUS |  |  | PTPRS |  |  |  |  |
|  | FXN |  |  | PYGL |  |  |  |  |
|  | FYN |  |  | PYGM |  |  |  |  |
|  | G3BP1 |  |  | RAB9A |  |  |  |  |
|  | G6PC2 |  |  | REN |  |  |  |  |
|  | G6PD |  |  | RFK |  |  |  |  |
|  | GABBR1 |  |  | ROCK2 |  |  |  |  |
|  | GABBR2 |  |  | RORA |  |  |  |  |
|  | GABRA1 |  |  | RORC |  |  |  |  |
|  | GABRB3 |  |  | RPS6KA3 |  |  |  |  |
|  | GABRG2 |  |  | S1PR2 |  |  |  |  |
|  | GAD1 |  |  | S1PR3 |  |  |  |  |
|  | GAD2 |  |  | SCD |  |  |  |  |
|  | GAL |  |  | SERPINA6 |  |  |  |  |
|  | GALC |  |  | SHBG |  |  |  |  |
|  | GAMT |  |  | SIGMAR1 |  |  |  |  |
|  | GAP43 |  |  | SLC10A2 |  |  |  |  |
|  | GAS2 |  |  | SLC22A11 |  |  |  |  |
|  | GAS6 |  |  | SLC22A12 |  |  |  |  |
|  | GAST |  |  | SLC22A6 |  |  |  |  |
|  | GATA3 |  |  | SLC28A2 |  |  |  |  |
|  | GBA1 |  |  | SLC28A3 |  |  |  |  |
|  | GBP1 |  |  | SLC29A1 |  |  |  |  |
|  | GBP3 |  |  | SLC2A2 |  |  |  |  |
|  | GCH1 |  |  | SLC47A1 |  |  |  |  |
|  | GDNF |  |  | SLC5A1 |  |  |  |  |
|  | GEM |  |  | SLC5A2 |  |  |  |  |
|  | GFAP |  |  | SLC6A2 |  |  |  |  |
|  | GFI1 |  |  | SLC6A3 |  |  |  |  |
|  | GFRA1 |  |  | SLC6A4 |  |  |  |  |
|  | GGT1 |  |  | SLC6A9 |  |  |  |  |
|  | GHRL |  |  | SMO |  |  |  |  |
|  | GIMAP4 |  |  | SQLE |  |  |  |  |
|  | GJA1 |  |  | SRC |  |  |  |  |
|  | GJB1 |  |  | SRD5A1 |  |  |  |  |
|  | GLIS1 |  |  | SREBF1 |  |  |  |  |
|  | GLOD4 |  |  | STAT3 |  |  |  |  |
|  | GLRB |  |  | STK17B |  |  |  |  |
|  | GLS |  |  | STS |  |  |  |  |
|  | GLUL |  |  | SYK |  |  |  |  |
|  | GNB5 |  |  | TACR1 |  |  |  |  |
|  | GNG12 |  |  | TAS2R31 |  |  |  |  |
|  | GPATCH8 |  |  | TBXA2R |  |  |  |  |
|  | GPR137B |  |  | TBXAS1 |  |  |  |  |
|  | GPR18 |  |  | TEK |  |  |  |  |
|  | GPR20 |  |  | TERT |  |  |  |  |
|  | GPT |  |  | TH |  |  |  |  |
|  | GPT2 |  |  | TK2 |  |  |  |  |
|  | GRIA1 |  |  | TNF |  |  |  |  |
|  | GRIA2 |  |  | TNKS |  |  |  |  |
|  | GRIK2 |  |  | TNKS2 |  |  |  |  |
|  | GRIN1 |  |  | TOP1 |  |  |  |  |
|  | GRIN2A |  |  | TOP2A |  |  |  |  |
|  | GRIN2B |  |  | TPMT |  |  |  |  |
|  | GRM1 |  |  | TRPV1 |  |  |  |  |
|  | GRM5 |  |  | TRPV3 |  |  |  |  |
|  | GRM7 |  |  | TRPV4 |  |  |  |  |
|  | GRN |  |  | TTR |  |  |  |  |
|  | GSK3B |  |  | TYR |  |  |  |  |
|  | GSR |  |  | VAV1 |  |  |  |  |
|  | GSTP1 |  |  | VDR |  |  |  |  |
|  | GTF2B |  |  | VEGFA |  |  |  |  |
|  | GTF2I |  |  | XDH |  |  |  |  |
|  | GTF3C1 |  |  |  |  |  |  |  |
|  | GUSB |  |  |  |  |  |  |  |
|  | GZMA |  |  |  |  |  |  |  |
|  | GZMB |  |  |  |  |  |  |  |
|  | HADHA |  |  |  |  |  |  |  |
|  | HAMP |  |  |  |  |  |  |  |
|  | HCRT |  |  |  |  |  |  |  |
|  | HDAC9 |  |  |  |  |  |  |  |
|  | HFE |  |  |  |  |  |  |  |
|  | HINFP |  |  |  |  |  |  |  |
|  | HINT1 |  |  |  |  |  |  |  |
|  | HIVEP3 |  |  |  |  |  |  |  |
|  | HLA-B |  |  |  |  |  |  |  |
|  | HLA-DQB1 |  |  |  |  |  |  |  |
|  | HLA-DRA |  |  |  |  |  |  |  |
|  | HLA-DRB1 |  |  |  |  |  |  |  |
|  | HLA-DRB5 |  |  |  |  |  |  |  |
|  | HLA-E |  |  |  |  |  |  |  |
|  | HLA-F |  |  |  |  |  |  |  |
|  | HM13 |  |  |  |  |  |  |  |
|  | HMBS |  |  |  |  |  |  |  |
|  | HMGCR |  |  |  |  |  |  |  |
|  | HMOX1 |  |  |  |  |  |  |  |
|  | HMOX2 |  |  |  |  |  |  |  |
|  | HNRNPDL |  |  |  |  |  |  |  |
|  | HNRNPK |  |  |  |  |  |  |  |
|  | HOXA1 |  |  |  |  |  |  |  |
|  | HP |  |  |  |  |  |  |  |
|  | HPRT1 |  |  |  |  |  |  |  |
|  | HRH1 |  |  |  |  |  |  |  |
|  | HRH2 |  |  |  |  |  |  |  |
|  | HRH3 |  |  |  |  |  |  |  |
|  | HSD3B7 |  |  |  |  |  |  |  |
|  | HSP90AA1 |  |  |  |  |  |  |  |
|  | HSP90AB1 |  |  |  |  |  |  |  |
|  | HSP90B1 |  |  |  |  |  |  |  |
|  | HSPA1A |  |  |  |  |  |  |  |
|  | HSPA1B |  |  |  |  |  |  |  |
|  | HSPA2 |  |  |  |  |  |  |  |
|  | HSPA4 |  |  |  |  |  |  |  |
|  | HSPA5 |  |  |  |  |  |  |  |
|  | HSPA8 |  |  |  |  |  |  |  |
|  | HSPB1 |  |  |  |  |  |  |  |
|  | HSPD1 |  |  |  |  |  |  |  |
|  | HSPE1 |  |  |  |  |  |  |  |
|  | HSPG2 |  |  |  |  |  |  |  |
|  | HTR1A |  |  |  |  |  |  |  |
|  | HTR2A |  |  |  |  |  |  |  |
|  | HTR3A |  |  |  |  |  |  |  |
|  | HTT |  |  |  |  |  |  |  |
|  | HYLS1 |  |  |  |  |  |  |  |
|  | HYOU1 |  |  |  |  |  |  |  |
|  | IAPP |  |  |  |  |  |  |  |
|  | ICA1 |  |  |  |  |  |  |  |
|  | ICAM3 |  |  |  |  |  |  |  |
|  | IDO1 |  |  |  |  |  |  |  |
|  | IER3 |  |  |  |  |  |  |  |
|  | IER5 |  |  |  |  |  |  |  |
|  | IFIT2 |  |  |  |  |  |  |  |
|  | IFNA1 |  |  |  |  |  |  |  |
|  | IFNA2 |  |  |  |  |  |  |  |
|  | IFNAR1 |  |  |  |  |  |  |  |
|  | IFNB1 |  |  |  |  |  |  |  |
|  | IFNG |  |  |  |  |  |  |  |
|  | IFNGR1 |  |  |  |  |  |  |  |
|  | IGF1 |  |  |  |  |  |  |  |
|  | IGF1R |  |  |  |  |  |  |  |
|  | IGF2 |  |  |  |  |  |  |  |
|  | IGF2BP3 |  |  |  |  |  |  |  |
|  | IGFBP2 |  |  |  |  |  |  |  |
|  | IGFBP3 |  |  |  |  |  |  |  |
|  | IGFBP4 |  |  |  |  |  |  |  |
|  | IGHM |  |  |  |  |  |  |  |
|  | IGKC |  |  |  |  |  |  |  |
|  | IL10 |  |  |  |  |  |  |  |
|  | IL12A |  |  |  |  |  |  |  |
|  | IL12B |  |  |  |  |  |  |  |
|  | IL13RA1 |  |  |  |  |  |  |  |
|  | IL15 |  |  |  |  |  |  |  |
|  | IL15RA |  |  |  |  |  |  |  |
|  | IL16 |  |  |  |  |  |  |  |
|  | IL17A |  |  |  |  |  |  |  |
|  | IL17RA |  |  |  |  |  |  |  |
|  | IL18 |  |  |  |  |  |  |  |
|  | IL1A |  |  |  |  |  |  |  |
|  | IL1B |  |  |  |  |  |  |  |
|  | IL1R1 |  |  |  |  |  |  |  |
|  | IL1RAPL2 |  |  |  |  |  |  |  |
|  | IL1RN |  |  |  |  |  |  |  |
|  | IL2 |  |  |  |  |  |  |  |
|  | IL21 |  |  |  |  |  |  |  |
|  | IL23A |  |  |  |  |  |  |  |
|  | IL2RA |  |  |  |  |  |  |  |
|  | IL3 |  |  |  |  |  |  |  |
|  | IL32 |  |  |  |  |  |  |  |
|  | IL34 |  |  |  |  |  |  |  |
|  | IL4 |  |  |  |  |  |  |  |
|  | IL4R |  |  |  |  |  |  |  |
|  | IL6 |  |  |  |  |  |  |  |
|  | ILF3 |  |  |  |  |  |  |  |
|  | INA |  |  |  |  |  |  |  |
|  | INPP5D |  |  |  |  |  |  |  |
|  | INS |  |  |  |  |  |  |  |
|  | IRAG2 |  |  |  |  |  |  |  |
|  | IREB2 |  |  |  |  |  |  |  |
|  | ISG15 |  |  |  |  |  |  |  |
|  | ISL2 |  |  |  |  |  |  |  |
|  | ITGAM |  |  |  |  |  |  |  |
|  | ITK |  |  |  |  |  |  |  |
|  | IVL |  |  |  |  |  |  |  |
|  | JAK1 |  |  |  |  |  |  |  |
|  | JAK2 |  |  |  |  |  |  |  |
|  | JCHAIN |  |  |  |  |  |  |  |
|  | JUN |  |  |  |  |  |  |  |
|  | JUP |  |  |  |  |  |  |  |
|  | KANSL1 |  |  |  |  |  |  |  |
|  | KBTBD11 |  |  |  |  |  |  |  |
|  | KCNH2 |  |  |  |  |  |  |  |
|  | KCNJ11 |  |  |  |  |  |  |  |
|  | KCNJ3 |  |  |  |  |  |  |  |
|  | KCNQ1 |  |  |  |  |  |  |  |
|  | KCNQ4 |  |  |  |  |  |  |  |
|  | KCNT1 |  |  |  |  |  |  |  |
|  | KDM1A |  |  |  |  |  |  |  |
|  | KDM2A |  |  |  |  |  |  |  |
|  | KDM4B |  |  |  |  |  |  |  |
|  | KDM6B |  |  |  |  |  |  |  |
|  | KEAP1 |  |  |  |  |  |  |  |
|  | KIF21B |  |  |  |  |  |  |  |
|  | KIF3A |  |  |  |  |  |  |  |
|  | KIT |  |  |  |  |  |  |  |
|  | KITLG |  |  |  |  |  |  |  |
|  | KL |  |  |  |  |  |  |  |
|  | KLF13 |  |  |  |  |  |  |  |
|  | KLF2 |  |  |  |  |  |  |  |
|  | KLF5 |  |  |  |  |  |  |  |
|  | KMT2D |  |  |  |  |  |  |  |
|  | KRCC1 |  |  |  |  |  |  |  |
|  | KRT10 |  |  |  |  |  |  |  |
|  | KRT80 |  |  |  |  |  |  |  |
|  | LAMA5 |  |  |  |  |  |  |  |
|  | LAMC1 |  |  |  |  |  |  |  |
|  | LAMC2 |  |  |  |  |  |  |  |
|  | LAMP1 |  |  |  |  |  |  |  |
|  | LAMP2 |  |  |  |  |  |  |  |
|  | LASP1 |  |  |  |  |  |  |  |
|  | LAT |  |  |  |  |  |  |  |
|  | LCK |  |  |  |  |  |  |  |
|  | LCN2 |  |  |  |  |  |  |  |
|  | LDHB |  |  |  |  |  |  |  |
|  | LDLR |  |  |  |  |  |  |  |
|  | LEF1 |  |  |  |  |  |  |  |
|  | LEP |  |  |  |  |  |  |  |
|  | LEPR |  |  |  |  |  |  |  |
|  | LGALS7 |  |  |  |  |  |  |  |
|  | LHX2 |  |  |  |  |  |  |  |
|  | LIF |  |  |  |  |  |  |  |
|  | LIMD1 |  |  |  |  |  |  |  |
|  | LMCD1 |  |  |  |  |  |  |  |
|  | LONRF2 |  |  |  |  |  |  |  |
|  | LPP |  |  |  |  |  |  |  |
|  | LRRK2 |  |  |  |  |  |  |  |
|  | LSG1 |  |  |  |  |  |  |  |
|  | LTBR |  |  |  |  |  |  |  |
|  | LTF |  |  |  |  |  |  |  |
|  | MAF |  |  |  |  |  |  |  |
|  | MAG |  |  |  |  |  |  |  |
|  | MAGED1 |  |  |  |  |  |  |  |
|  | MAGED4 |  |  |  |  |  |  |  |
|  | MAN2B2 |  |  |  |  |  |  |  |
|  | MAOA |  |  |  |  |  |  |  |
|  | MAOB |  |  |  |  |  |  |  |
|  | MAP1LC3B |  |  |  |  |  |  |  |
|  | MAP2 |  |  |  |  |  |  |  |
|  | MAP3K12 |  |  |  |  |  |  |  |
|  | MAP3K7 |  |  |  |  |  |  |  |
|  | MAPK1 |  |  |  |  |  |  |  |
|  | MAPK14 |  |  |  |  |  |  |  |
|  | MAPK3 |  |  |  |  |  |  |  |
|  | MAPK7 |  |  |  |  |  |  |  |
|  | MAPK8 |  |  |  |  |  |  |  |
|  | MAPT |  |  |  |  |  |  |  |
|  | MARCHF7 |  |  |  |  |  |  |  |
|  | MARCKS |  |  |  |  |  |  |  |
|  | MB |  |  |  |  |  |  |  |
|  | MBP |  |  |  |  |  |  |  |
|  | MDFIC |  |  |  |  |  |  |  |
|  | MDM2 |  |  |  |  |  |  |  |
|  | MECP2 |  |  |  |  |  |  |  |
|  | MED13L |  |  |  |  |  |  |  |
|  | MELK |  |  |  |  |  |  |  |
|  | MEN1 |  |  |  |  |  |  |  |
|  | METRN |  |  |  |  |  |  |  |
|  | MFGE8 |  |  |  |  |  |  |  |
|  | MIF |  |  |  |  |  |  |  |
|  | MITF |  |  |  |  |  |  |  |
|  | MLH1 |  |  |  |  |  |  |  |
|  | MLLT11 |  |  |  |  |  |  |  |
|  | MMEL1 |  |  |  |  |  |  |  |
|  | MMP11 |  |  |  |  |  |  |  |
|  | MMP8 |  |  |  |  |  |  |  |
|  | MMP9 |  |  |  |  |  |  |  |
|  | MOG |  |  |  |  |  |  |  |
|  | MPC2 |  |  |  |  |  |  |  |
|  | MPG |  |  |  |  |  |  |  |
|  | MPL |  |  |  |  |  |  |  |
|  | MR1 |  |  |  |  |  |  |  |
|  | MS4A3 |  |  |  |  |  |  |  |
|  | MSH2 |  |  |  |  |  |  |  |
|  | MSH3 |  |  |  |  |  |  |  |
|  | MSN |  |  |  |  |  |  |  |
|  | MSS51 |  |  |  |  |  |  |  |
|  | MSX1 |  |  |  |  |  |  |  |
|  | MT-CYB |  |  |  |  |  |  |  |
|  | MTHFD1 |  |  |  |  |  |  |  |
|  | MTHFD2 |  |  |  |  |  |  |  |
|  | MTHFR |  |  |  |  |  |  |  |
|  | MT-ND5 |  |  |  |  |  |  |  |
|  | MTOR |  |  |  |  |  |  |  |
|  | MTR |  |  |  |  |  |  |  |
|  | MUC13 |  |  |  |  |  |  |  |
|  | MUCL1 |  |  |  |  |  |  |  |
|  | MX2 |  |  |  |  |  |  |  |
|  | MXI1 |  |  |  |  |  |  |  |
|  | MYC |  |  |  |  |  |  |  |
|  | MYD88 |  |  |  |  |  |  |  |
|  | MYH10 |  |  |  |  |  |  |  |
|  | NAGLU |  |  |  |  |  |  |  |
|  | NAIP |  |  |  |  |  |  |  |
|  | NAMPT |  |  |  |  |  |  |  |
|  | NANOG |  |  |  |  |  |  |  |
|  | NANOS1 |  |  |  |  |  |  |  |
|  | NASP |  |  |  |  |  |  |  |
|  | NAT2 |  |  |  |  |  |  |  |
|  | NCAM1 |  |  |  |  |  |  |  |
|  | NCOA7 |  |  |  |  |  |  |  |
|  | NCSTN |  |  |  |  |  |  |  |
|  | NDUFS2 |  |  |  |  |  |  |  |
|  | NEFL |  |  |  |  |  |  |  |
|  | NES |  |  |  |  |  |  |  |
|  | NETO2 |  |  |  |  |  |  |  |
|  | NEU1 |  |  |  |  |  |  |  |
|  | NF1 |  |  |  |  |  |  |  |
|  | NFATC3 |  |  |  |  |  |  |  |
|  | NFE2L2 |  |  |  |  |  |  |  |
|  | NFE2L3 |  |  |  |  |  |  |  |
|  | NFKB1 |  |  |  |  |  |  |  |
|  | NFKBIA |  |  |  |  |  |  |  |
|  | NFKBIL1 |  |  |  |  |  |  |  |
|  | NFKBIZ |  |  |  |  |  |  |  |
|  | NGF |  |  |  |  |  |  |  |
|  | NGFR |  |  |  |  |  |  |  |
|  | NID1 |  |  |  |  |  |  |  |
|  | NKAIN1 |  |  |  |  |  |  |  |
|  | NKX2-1 |  |  |  |  |  |  |  |
|  | NLGN3 |  |  |  |  |  |  |  |
|  | NLGN4X |  |  |  |  |  |  |  |
|  | NME1 |  |  |  |  |  |  |  |
|  | NOD2 |  |  |  |  |  |  |  |
|  | NOS1 |  |  |  |  |  |  |  |
|  | NOS2 |  |  |  |  |  |  |  |
|  | NOS3 |  |  |  |  |  |  |  |
|  | NOTCH1 |  |  |  |  |  |  |  |
|  | NOTCH3 |  |  |  |  |  |  |  |
|  | NPM1 |  |  |  |  |  |  |  |
|  | NPPA |  |  |  |  |  |  |  |
|  | NPPB |  |  |  |  |  |  |  |
|  | NPRL2 |  |  |  |  |  |  |  |
|  | NPY |  |  |  |  |  |  |  |
|  | NR1H4 |  |  |  |  |  |  |  |
|  | NR2C2 |  |  |  |  |  |  |  |
|  | NR3C1 |  |  |  |  |  |  |  |
|  | NR3C2 |  |  |  |  |  |  |  |
|  | NR4A2 |  |  |  |  |  |  |  |
|  | NR5A1 |  |  |  |  |  |  |  |
|  | NRAP |  |  |  |  |  |  |  |
|  | NRDC |  |  |  |  |  |  |  |
|  | NRIP1 |  |  |  |  |  |  |  |
|  | NRP1 |  |  |  |  |  |  |  |
|  | NRP2 |  |  |  |  |  |  |  |
|  | NT5C2 |  |  |  |  |  |  |  |
|  | NTF3 |  |  |  |  |  |  |  |
|  | NTF4 |  |  |  |  |  |  |  |
|  | NTNG2 |  |  |  |  |  |  |  |
|  | NTRK1 |  |  |  |  |  |  |  |
|  | NTRK2 |  |  |  |  |  |  |  |
|  | NTS |  |  |  |  |  |  |  |
|  | NUP42 |  |  |  |  |  |  |  |
|  | OGG1 |  |  |  |  |  |  |  |
|  | OPN3 |  |  |  |  |  |  |  |
|  | OPRM1 |  |  |  |  |  |  |  |
|  | ORM1 |  |  |  |  |  |  |  |
|  | OSM |  |  |  |  |  |  |  |
|  | OXA1L |  |  |  |  |  |  |  |
|  | OXT |  |  |  |  |  |  |  |
|  | P2RX3 |  |  |  |  |  |  |  |
|  | P2RX7 |  |  |  |  |  |  |  |
|  | P2RY2 |  |  |  |  |  |  |  |
|  | PAFAH1B1 |  |  |  |  |  |  |  |
|  | PAH |  |  |  |  |  |  |  |
|  | PAIP1 |  |  |  |  |  |  |  |
|  | PALM2AKAP2 |  |  |  |  |  |  |  |
|  | PANK2 |  |  |  |  |  |  |  |
|  | PARD3 |  |  |  |  |  |  |  |
|  | PARK7 |  |  |  |  |  |  |  |
|  | PARP1 |  |  |  |  |  |  |  |
|  | PAX5 |  |  |  |  |  |  |  |
|  | PCSK2 |  |  |  |  |  |  |  |
|  | PCSK9 |  |  |  |  |  |  |  |
|  | PDCD4 |  |  |  |  |  |  |  |
|  | PDE4A |  |  |  |  |  |  |  |
|  | PDE5A |  |  |  |  |  |  |  |
|  | PDGFRB |  |  |  |  |  |  |  |
|  | PDIA4 |  |  |  |  |  |  |  |
|  | PDYN |  |  |  |  |  |  |  |
|  | PELI1 |  |  |  |  |  |  |  |
|  | PEPD |  |  |  |  |  |  |  |
|  | PER1 |  |  |  |  |  |  |  |
|  | PER2 |  |  |  |  |  |  |  |
|  | PGR |  |  |  |  |  |  |  |
|  | PHLDA2 |  |  |  |  |  |  |  |
|  | PIGA |  |  |  |  |  |  |  |
|  | PIGQ |  |  |  |  |  |  |  |
|  | PIGR |  |  |  |  |  |  |  |
|  | PIK3C2A |  |  |  |  |  |  |  |
|  | PIK3C3 |  |  |  |  |  |  |  |
|  | PIM1 |  |  |  |  |  |  |  |
|  | PINK1 |  |  |  |  |  |  |  |
|  | PLA2G2A |  |  |  |  |  |  |  |
|  | PLA2G6 |  |  |  |  |  |  |  |
|  | PLOD1 |  |  |  |  |  |  |  |
|  | PLOD3 |  |  |  |  |  |  |  |
|  | PLXND1 |  |  |  |  |  |  |  |
|  | PMS1 |  |  |  |  |  |  |  |
|  | PMS2 |  |  |  |  |  |  |  |
|  | PNP |  |  |  |  |  |  |  |
|  | POLG |  |  |  |  |  |  |  |
|  | POLGARF |  |  |  |  |  |  |  |
|  | POLM |  |  |  |  |  |  |  |
|  | POLR1C |  |  |  |  |  |  |  |
|  | POLR3A |  |  |  |  |  |  |  |
|  | POLR3B |  |  |  |  |  |  |  |
|  | POMC |  |  |  |  |  |  |  |
|  | PON1 |  |  |  |  |  |  |  |
|  | POSTN |  |  |  |  |  |  |  |
|  | POU5F1 |  |  |  |  |  |  |  |
|  | PPARG |  |  |  |  |  |  |  |
|  | PPIA |  |  |  |  |  |  |  |
|  | PPOX |  |  |  |  |  |  |  |
|  | PPP1R1B |  |  |  |  |  |  |  |
|  | PRDM13 |  |  |  |  |  |  |  |
|  | PRKACA |  |  |  |  |  |  |  |
|  | PRKCD |  |  |  |  |  |  |  |
|  | PRKCG |  |  |  |  |  |  |  |
|  | PRKG1 |  |  |  |  |  |  |  |
|  | PRKN |  |  |  |  |  |  |  |
|  | PRL |  |  |  |  |  |  |  |
|  | PRLR |  |  |  |  |  |  |  |
|  | PRNP |  |  |  |  |  |  |  |
|  | PROCR |  |  |  |  |  |  |  |
|  | PRR12 |  |  |  |  |  |  |  |
|  | PRRT2 |  |  |  |  |  |  |  |
|  | PRSS3 |  |  |  |  |  |  |  |
|  | PRTN3 |  |  |  |  |  |  |  |
|  | PSAP |  |  |  |  |  |  |  |
|  | PSMA5 |  |  |  |  |  |  |  |
|  | PTCRA |  |  |  |  |  |  |  |
|  | PTEN |  |  |  |  |  |  |  |
|  | PTGER1 |  |  |  |  |  |  |  |
|  | PTGER4 |  |  |  |  |  |  |  |
|  | PTGES3 |  |  |  |  |  |  |  |
|  | PTGS2 |  |  |  |  |  |  |  |
|  | PTH1R |  |  |  |  |  |  |  |
|  | PTP4A1 |  |  |  |  |  |  |  |
|  | PTPN11 |  |  |  |  |  |  |  |
|  | PTPRC |  |  |  |  |  |  |  |
|  | PURA |  |  |  |  |  |  |  |
|  | PVALB |  |  |  |  |  |  |  |
|  | QDPR |  |  |  |  |  |  |  |
|  | RAD50 |  |  |  |  |  |  |  |
|  | RAD54L |  |  |  |  |  |  |  |
|  | RAF1 |  |  |  |  |  |  |  |
|  | RAG1 |  |  |  |  |  |  |  |
|  | RAI1 |  |  |  |  |  |  |  |
|  | RAMP1 |  |  |  |  |  |  |  |
|  | RAPSN |  |  |  |  |  |  |  |
|  | RARB |  |  |  |  |  |  |  |
|  | RARG |  |  |  |  |  |  |  |
|  | RBMX |  |  |  |  |  |  |  |
|  | RBPMS |  |  |  |  |  |  |  |
|  | RC3H1 |  |  |  |  |  |  |  |
|  | RC3H2 |  |  |  |  |  |  |  |
|  | RDX |  |  |  |  |  |  |  |
|  | REG3A |  |  |  |  |  |  |  |
|  | REG3G |  |  |  |  |  |  |  |
|  | RELN |  |  |  |  |  |  |  |
|  | REV1 |  |  |  |  |  |  |  |
|  | RING1 |  |  |  |  |  |  |  |
|  | RIPPLY3 |  |  |  |  |  |  |  |
|  | RNASE2 |  |  |  |  |  |  |  |
|  | RND1 |  |  |  |  |  |  |  |
|  | RNF180 |  |  |  |  |  |  |  |
|  | RNF216 |  |  |  |  |  |  |  |
|  | RNF44 |  |  |  |  |  |  |  |
|  | RNF5 |  |  |  |  |  |  |  |
|  | RNPC3 |  |  |  |  |  |  |  |
|  | RORC |  |  |  |  |  |  |  |
|  | RPA1 |  |  |  |  |  |  |  |
|  | RPA2 |  |  |  |  |  |  |  |
|  | RPLP0 |  |  |  |  |  |  |  |
|  | RPLP2 |  |  |  |  |  |  |  |
|  | RPS24 |  |  |  |  |  |  |  |
|  | RPS26 |  |  |  |  |  |  |  |
|  | RPS27A |  |  |  |  |  |  |  |
|  | RPS3 |  |  |  |  |  |  |  |
|  | RPS6KA2 |  |  |  |  |  |  |  |
|  | RRAGA |  |  |  |  |  |  |  |
|  | RRAS2 |  |  |  |  |  |  |  |
|  | RRM1 |  |  |  |  |  |  |  |
|  | RRM2B |  |  |  |  |  |  |  |
|  | RTEL1 |  |  |  |  |  |  |  |
|  | RUNX1 |  |  |  |  |  |  |  |
|  | RUNX3 |  |  |  |  |  |  |  |
|  | RYR1 |  |  |  |  |  |  |  |
|  | RYR2 |  |  |  |  |  |  |  |
|  | S100B |  |  |  |  |  |  |  |
|  | S100P |  |  |  |  |  |  |  |
|  | SALL1 |  |  |  |  |  |  |  |
|  | SAT1 |  |  |  |  |  |  |  |
|  | SATB1 |  |  |  |  |  |  |  |
|  | SBSN |  |  |  |  |  |  |  |
|  | SCG3 |  |  |  |  |  |  |  |
|  | SCN11A |  |  |  |  |  |  |  |
|  | SCN1A |  |  |  |  |  |  |  |
|  | SCN2A |  |  |  |  |  |  |  |
|  | SCN5A |  |  |  |  |  |  |  |
|  | SCN8A |  |  |  |  |  |  |  |
|  | SCRT1 |  |  |  |  |  |  |  |
|  | SCT |  |  |  |  |  |  |  |
|  | SCUBE2 |  |  |  |  |  |  |  |
|  | SEC23A |  |  |  |  |  |  |  |
|  | SELP |  |  |  |  |  |  |  |
|  | SEMA3A |  |  |  |  |  |  |  |
|  | SEPTIN2 |  |  |  |  |  |  |  |
|  | SERPINE1 |  |  |  |  |  |  |  |
|  | SETD1A |  |  |  |  |  |  |  |
|  | SETD1B |  |  |  |  |  |  |  |
|  | SFN |  |  |  |  |  |  |  |
|  | SGCE |  |  |  |  |  |  |  |
|  | SHARPIN |  |  |  |  |  |  |  |
|  | SHBG |  |  |  |  |  |  |  |
|  | SIRT1 |  |  |  |  |  |  |  |
|  | SIRT5 |  |  |  |  |  |  |  |
|  | SIT1 |  |  |  |  |  |  |  |
|  | SKP1 |  |  |  |  |  |  |  |
|  | SLC12A5 |  |  |  |  |  |  |  |
|  | SLC17A5 |  |  |  |  |  |  |  |
|  | SLC18A2 |  |  |  |  |  |  |  |
|  | SLC18A3 |  |  |  |  |  |  |  |
|  | SLC1A1 |  |  |  |  |  |  |  |
|  | SLC1A2 |  |  |  |  |  |  |  |
|  | SLC1A3 |  |  |  |  |  |  |  |
|  | SLC1A4 |  |  |  |  |  |  |  |
|  | SLC20A2 |  |  |  |  |  |  |  |
|  | SLC22A3 |  |  |  |  |  |  |  |
|  | SLC22A4 |  |  |  |  |  |  |  |
|  | SLC22A5 |  |  |  |  |  |  |  |
|  | SLC25A15 |  |  |  |  |  |  |  |
|  | SLC26A1 |  |  |  |  |  |  |  |
|  | SLC2A6 |  |  |  |  |  |  |  |
|  | SLC34A1 |  |  |  |  |  |  |  |
|  | SLC36A1 |  |  |  |  |  |  |  |
|  | SLC38A2 |  |  |  |  |  |  |  |
|  | SLC39A13 |  |  |  |  |  |  |  |
|  | SLC39A14 |  |  |  |  |  |  |  |
|  | SLC39A5 |  |  |  |  |  |  |  |
|  | SLC39A6 |  |  |  |  |  |  |  |
|  | SLC39A8 |  |  |  |  |  |  |  |
|  | SLC40A1 |  |  |  |  |  |  |  |
|  | SLC43A1 |  |  |  |  |  |  |  |
|  | SLC45A1 |  |  |  |  |  |  |  |
|  | SLC6A3 |  |  |  |  |  |  |  |
|  | SLC6A4 |  |  |  |  |  |  |  |
|  | SLC7A5 |  |  |  |  |  |  |  |
|  | SLC7A7 |  |  |  |  |  |  |  |
|  | SLIT1 |  |  |  |  |  |  |  |
|  | SLPI |  |  |  |  |  |  |  |
|  | SMAD6 |  |  |  |  |  |  |  |
|  | SMARCA4 |  |  |  |  |  |  |  |
|  | SMPD1 |  |  |  |  |  |  |  |
|  | SNCA |  |  |  |  |  |  |  |
|  | SNRNP70 |  |  |  |  |  |  |  |
|  | SNRPB |  |  |  |  |  |  |  |
|  | SNRPD1 |  |  |  |  |  |  |  |
|  | SNRPD3 |  |  |  |  |  |  |  |
|  | SNRPE |  |  |  |  |  |  |  |
|  | SNRPN |  |  |  |  |  |  |  |
|  | SNX10 |  |  |  |  |  |  |  |
|  | SOAT1 |  |  |  |  |  |  |  |
|  | SOCS1 |  |  |  |  |  |  |  |
|  | SOCS7 |  |  |  |  |  |  |  |
|  | SOD1 |  |  |  |  |  |  |  |
|  | SOD2 |  |  |  |  |  |  |  |
|  | SORD |  |  |  |  |  |  |  |
|  | SP1 |  |  |  |  |  |  |  |
|  | SP2 |  |  |  |  |  |  |  |
|  | SPAST |  |  |  |  |  |  |  |
|  | SPP1 |  |  |  |  |  |  |  |
|  | SPTAN1 |  |  |  |  |  |  |  |
|  | SREK1IP1 |  |  |  |  |  |  |  |
|  | SRM |  |  |  |  |  |  |  |
|  | SSRP1 |  |  |  |  |  |  |  |
|  | SST |  |  |  |  |  |  |  |
|  | ST13 |  |  |  |  |  |  |  |
|  | ST14 |  |  |  |  |  |  |  |
|  | STAT1 |  |  |  |  |  |  |  |
|  | STAT3 |  |  |  |  |  |  |  |
|  | STAT5B |  |  |  |  |  |  |  |
|  | STIP1 |  |  |  |  |  |  |  |
|  | STK11 |  |  |  |  |  |  |  |
|  | STK39 |  |  |  |  |  |  |  |
|  | STK4 |  |  |  |  |  |  |  |
|  | STMN3 |  |  |  |  |  |  |  |
|  | STOM |  |  |  |  |  |  |  |
|  | STUB1 |  |  |  |  |  |  |  |
|  | STX1A |  |  |  |  |  |  |  |
|  | STXBP1 |  |  |  |  |  |  |  |
|  | SULT1A3 |  |  |  |  |  |  |  |
|  | SUN1 |  |  |  |  |  |  |  |
|  | SUOX |  |  |  |  |  |  |  |
|  | SYK |  |  |  |  |  |  |  |
|  | SYN2 |  |  |  |  |  |  |  |
|  | SYNGAP1 |  |  |  |  |  |  |  |
|  | SYP |  |  |  |  |  |  |  |
|  | TAC1 |  |  |  |  |  |  |  |
|  | TACR1 |  |  |  |  |  |  |  |
|  | TACR3 |  |  |  |  |  |  |  |
|  | TAFA5 |  |  |  |  |  |  |  |
|  | TANC2 |  |  |  |  |  |  |  |
|  | TAP1 |  |  |  |  |  |  |  |
|  | TARDBP |  |  |  |  |  |  |  |
|  | TAT |  |  |  |  |  |  |  |
|  | TBL1XR1 |  |  |  |  |  |  |  |
|  | TBP |  |  |  |  |  |  |  |
|  | TBX21 |  |  |  |  |  |  |  |
|  | TCF4 |  |  |  |  |  |  |  |
|  | TCF7 |  |  |  |  |  |  |  |
|  | TCN2 |  |  |  |  |  |  |  |
|  | TCP1 |  |  |  |  |  |  |  |
|  | TDO2 |  |  |  |  |  |  |  |
|  | TERT |  |  |  |  |  |  |  |
|  | TEX30 |  |  |  |  |  |  |  |
|  | TFAM |  |  |  |  |  |  |  |
|  | TFR2 |  |  |  |  |  |  |  |
|  | TFRC |  |  |  |  |  |  |  |
|  | TG |  |  |  |  |  |  |  |
|  | TGFB1 |  |  |  |  |  |  |  |
|  | TGFBR3 |  |  |  |  |  |  |  |
|  | TGM2 |  |  |  |  |  |  |  |
|  | TH |  |  |  |  |  |  |  |
|  | THNSL2 |  |  |  |  |  |  |  |
|  | THY1 |  |  |  |  |  |  |  |
|  | TIMP2 |  |  |  |  |  |  |  |
|  | TLR4 |  |  |  |  |  |  |  |
|  | TLR6 |  |  |  |  |  |  |  |
|  | TLR9 |  |  |  |  |  |  |  |
|  | TM4SF1 |  |  |  |  |  |  |  |
|  | TM6SF1 |  |  |  |  |  |  |  |
|  | TMED3 |  |  |  |  |  |  |  |
|  | TMEFF1 |  |  |  |  |  |  |  |
|  | TMEM267 |  |  |  |  |  |  |  |
|  | TMPO |  |  |  |  |  |  |  |
|  | TMPRSS2 |  |  |  |  |  |  |  |
|  | TNF |  |  |  |  |  |  |  |
|  | TNFAIP3 |  |  |  |  |  |  |  |
|  | TNFRSF14 |  |  |  |  |  |  |  |
|  | TNFRSF18 |  |  |  |  |  |  |  |
|  | TNFRSF19 |  |  |  |  |  |  |  |
|  | TNFRSF1A |  |  |  |  |  |  |  |
|  | TNFRSF1B |  |  |  |  |  |  |  |
|  | TNFSF10 |  |  |  |  |  |  |  |
|  | TNFSF12 |  |  |  |  |  |  |  |
|  | TNFSF13 |  |  |  |  |  |  |  |
|  | TNFSF13B |  |  |  |  |  |  |  |
|  | TNFSF9 |  |  |  |  |  |  |  |
|  | TNK2 |  |  |  |  |  |  |  |
|  | TNNI3 |  |  |  |  |  |  |  |
|  | TOR1A |  |  |  |  |  |  |  |
|  | TP53 |  |  |  |  |  |  |  |
|  | TPD52 |  |  |  |  |  |  |  |
|  | TPI1 |  |  |  |  |  |  |  |
|  | TPO |  |  |  |  |  |  |  |
|  | TRAF6 |  |  |  |  |  |  |  |
|  | TRAP1 |  |  |  |  |  |  |  |
|  | TREM1 |  |  |  |  |  |  |  |
|  | TREX1 |  |  |  |  |  |  |  |
|  | TRIM32 |  |  |  |  |  |  |  |
|  | TRIM58 |  |  |  |  |  |  |  |
|  | TRIML1 |  |  |  |  |  |  |  |
|  | TRPA1 |  |  |  |  |  |  |  |
|  | TRPC6 |  |  |  |  |  |  |  |
|  | TSC1 |  |  |  |  |  |  |  |
|  | TSC2 |  |  |  |  |  |  |  |
|  | TSHR |  |  |  |  |  |  |  |
|  | TSPO |  |  |  |  |  |  |  |
|  | TTR |  |  |  |  |  |  |  |
|  | TUBA4A |  |  |  |  |  |  |  |
|  | TUBB |  |  |  |  |  |  |  |
|  | TUBB2B |  |  |  |  |  |  |  |
|  | TUBB3 |  |  |  |  |  |  |  |
|  | TUG1 |  |  |  |  |  |  |  |
|  | TYMP |  |  |  |  |  |  |  |
|  | TYROBP |  |  |  |  |  |  |  |
|  | UBD |  |  |  |  |  |  |  |
|  | UBE2L3 |  |  |  |  |  |  |  |
|  | UBE3A |  |  |  |  |  |  |  |
|  | UBR7 |  |  |  |  |  |  |  |
|  | UCP1 |  |  |  |  |  |  |  |
|  | UMOD |  |  |  |  |  |  |  |
|  | UNG |  |  |  |  |  |  |  |
|  | UPK1A |  |  |  |  |  |  |  |
|  | UPK1B |  |  |  |  |  |  |  |
|  | UPK2 |  |  |  |  |  |  |  |
|  | UPK3A |  |  |  |  |  |  |  |
|  | USP18 |  |  |  |  |  |  |  |
|  | USP36 |  |  |  |  |  |  |  |
|  | USP44 |  |  |  |  |  |  |  |
|  | USP5 |  |  |  |  |  |  |  |
|  | VCAM1 |  |  |  |  |  |  |  |
|  | VCP |  |  |  |  |  |  |  |
|  | VDAC1 |  |  |  |  |  |  |  |
|  | VDR |  |  |  |  |  |  |  |
|  | VEGFA |  |  |  |  |  |  |  |
|  | VIM |  |  |  |  |  |  |  |
|  | VIP |  |  |  |  |  |  |  |
|  | VIPR1 |  |  |  |  |  |  |  |
|  | VIPR2 |  |  |  |  |  |  |  |
|  | VKORC1 |  |  |  |  |  |  |  |
|  | VPS13A |  |  |  |  |  |  |  |
|  | VPS13B |  |  |  |  |  |  |  |
|  | VPS13C |  |  |  |  |  |  |  |
|  | VPS13D |  |  |  |  |  |  |  |
|  | WAS |  |  |  |  |  |  |  |
|  | WWOX |  |  |  |  |  |  |  |
|  | XAF1 |  |  |  |  |  |  |  |
|  | XDH |  |  |  |  |  |  |  |
|  | XK |  |  |  |  |  |  |  |
|  | XPC |  |  |  |  |  |  |  |
|  | XRCC1 |  |  |  |  |  |  |  |
|  | XRCC3 |  |  |  |  |  |  |  |
|  | XRCC4 |  |  |  |  |  |  |  |
|  | XRCC6 |  |  |  |  |  |  |  |
|  | YWHAB |  |  |  |  |  |  |  |
|  | ZAP70 |  |  |  |  |  |  |  |
|  | ZBTB33 |  |  |  |  |  |  |  |
|  | ZBTB41 |  |  |  |  |  |  |  |
|  | ZC3H12B |  |  |  |  |  |  |  |
|  | ZDHHC1 |  |  |  |  |  |  |  |
|  | ZFP36L1 |  |  |  |  |  |  |  |
|  | ZFR |  |  |  |  |  |  |  |
|  | ZHX3 |  |  |  |  |  |  |  |
|  | ZIC1 |  |  |  |  |  |  |  |
|  | ZNF552 |  |  |  |  |  |  |  |
|  | ZNF671 |  |  |  |  |  |  |  |
|  | ZZEF1 |  |  |  |  |  |  |  |
