# Supplementary material for: ShenQi DiHuang Decoction (SQDHD) Ameliorates Neuroinflammation and Neuropsychiatric Manifestations in Pristane Induced Lupus Mice via Blocking JAK1‐STAT3 Pathway
Source: CNS Neurosci Ther. 2026 Mar 7;32(3):e70814. doi: 10.1002/cns.70814 (PMC12967629; doi:10.1002/cns.70814)
Supplement: Supplementary file 6 — Table S6: GO and KEGG analyses of the top 15 targets. [file CNS-32-e70814-s006.docx]

TableS6: GO and KEGG analyses of the 15 top targets

| **Go analysis** | |  |  |  |
| --- | --- | --- | --- | --- |
| **GO term** | **Subgroup** | **Count** | **P-value** | **Genes** |
| cell surface receptor signaling pathway via JAK-STAT | Biological process | 7 | ##### | IL4, IL6, CSF2, STAT3, CCL2, TNF, JAK1 |
| positive regulation of miRNA transcription | Biological process | 7 | ##### | IL10, IL6, STAT3, PPARG, TNF, FGF2, EGFR |
| positive regulation of smooth muscle cell proliferation | Biological process | 6 | ##### | IL6, AKT1, PTGS2, TNF, FGF2, EGFR |
| cellular response to lipopolysaccharide | Biological process | 7 | ##### | IL10, IL6, CSF2, CCL2, PTGS2, TNF, MMP9 |
| extracellular space | Cellular component | 10 | ##### | IL10, IL4, IL6, CSF2, CCL2, TNF, FGF2, MMP9, EGFR, IL2 |
| extracellular region | Cellular component | 9 | ##### | IL10, IL4, IL6, CSF2, CCL2, TNF, FGF2, MMP9, IL2 |
| protein-containing complex | Cellular component | 4 | 0.01 | AKT1, PTGS2, TNF, EGFR |
| RNA polymerase II transcription regulator complex | Cellular component | 2 | 0.082 | STAT3, PPARG |
| cytokine activity | Molecular function | 7 | ##### | IL10, IL4, IL6, CSF2, TNF, FGF2, IL2 |
| growth factor activity | Molecular function | 6 | ##### | IL10, IL4, IL6, CSF2, FGF2, IL2 |
| protein phosphatase binding | Molecular function | 4 | ##### | STAT3, PPARG, EGFR, JAK1 |
| identical protein binding | Molecular function | 8 | ##### | IL6, STAT3, AKT1, PPARG, TNF, FGF2, MMP9, EGFR |
|  |  |  |  |  |
| **KEGG analysis** | |  |  |  |
| **KEGG term** | **Enrichment score** | **Count** | **P-value** | **Genes** |
| JAK-STAT signaling pathway | 10.40599 | 9 | ##### | IL10, IL4, IL6, CSF2, STAT3, AKT1, EGFR, IL2, JAK1 |
| Pathway in cancer | 9.327587 | 11 | ##### | IL4, IL6, STAT3, AKT1, PPARG, PTGS2, FGF2, MMP9, EGFR, IL2, JAK1 |
| IL-17 signaling pathway | 8.434139 | 7 | ##### | IL4, IL6, CSF2, CCL2, PTGS2, TNF, MMP9 |
| TNF signaling pathway | 7.841031 | 7 | ##### | IL6, CSF2, CCL2, AKT1, PTGS2, TNF, MMP9 |
| Human cytomegalovirus infection | 7.717201 | 8 | ##### | IL6, STAT3, CCL2, AKT1, PTGS2, TNF, EGFR, JAK1 |
| Coronavirus disease - COVID-19 | 7.561537 | 8 | ##### | IL6, CSF2, STAT3, CCL2, TNF, EGFR, IL2, JAK1 |
| Inflammatory bowel disease | 7.456328 | 6 | ##### | IL10, IL4, IL6, STAT3, TNF, IL2 |
| EGFR tyrosine kinase inhibitor resistanc | 6.997926 | 6 | ##### | IL6, STAT3, AKT1, FGF2, EGFR, JAK1 |
| Kaposi sarcoma-associated herpesvirus infection | 6.545018 | 7 | ##### | IL6, CSF2, STAT3, AKT1, PTGS2, FGF2, JAK1 |
| Chagas disease | 6.445185 | 6 | ##### | IL10, IL6, CCL2, AKT1, TNF, IL2 |
