# Supplementary material for: ShenQi DiHuang Decoction (SQDHD) Ameliorates Neuroinflammation and Neuropsychiatric Manifestations in Pristane Induced Lupus Mice via Blocking JAK1‐STAT3 Pathway
Source: CNS Neurosci Ther. 2026 Mar 7;32(3):e70814. doi: 10.1002/cns.70814 (PMC12967629; doi:10.1002/cns.70814)
Supplement: Supplementary file 7 — Table S7: Degree values between the active compounds and the top 15 targets. [file CNS-32-e70814-s007.docx]

Table.S7: Degree values between the active compounds and the top 15 targets

| Compounds | Degree values | Genes |
| --- | --- | --- |
| Hederagenin | 3 | IL6,PTGS2,PPARG |
| Ellagic acid | 3 | IL4,AKT1,EGFR |
| Quercetin | 3 | AKT1,EGFR,MMP9 |
| Wogonin | 3 | CCL2,PTGS2,MMP9 |
| Alisol B Acetate | 3 | JAK1,EGFR,PTGS2 |
| Salidroside | 2 | IL10,AKT1 |
| Glycitein | 2 | IL2,EGFR |
| Formononetin | 2 | IL2,EGFR |
| Ononin | 2 | IL2,TNF |
| Calycosin-7-O-glucoside | 2 | IL2,TNF |
| Rhoifolin | 2 | IL2,TNF |
| Genistin | 2 | IL2,TNF |
| Isoformononetin | 2 | IL2,EGFR |
| Sissotrin | 2 | IL2,TNF |
| Catechin | 2 | CSF2,PTGS2 |
| Manninotriose | 2 | STAT3,FGF2 |
| Raffinose | 2 | STAT3,FGF2 |
| Rutin | 1 | EGFR |
| Isoluteolin | 1 | EGFR |
| alisol C,23-acetate | 1 | PTGS2 |
| Pachymic acid | 1 | PTGS2 |
| 3-O-Acetyl-16-hydroxytrametenolic acid | 1 | PTGS2 |
| Isoquercitrin | 1 | PTGS2 |
| Quercitrin | 1 | PTGS2 |
| Trifolin | 1 | PTGS2 |
| Astragalin | 1 | PTGS2 |
| Nobiletin | 1 | MMP9 |
| Caffeic acid | 1 | MMP9 |
| Vanillin | 1 | MMP9 |
| Vanillic acid | 1 | MMP9 |
| Procyanidin B1/B2 | 1 | MMP9 |
| 8-Debenzoylpaeoniflorin | 1 | FGF2 |
| D-Galactose | 1 | FGF2 |
| D-Fructose | 1 | FGF2 |
| D-Maltose | 1 | FGF2 |
| Melibiose | 1 | FGF2 |
| Oleic acid | 1 | PPARG |
| Cornuside | 1 | TNF |
